# Total Syntheses of Agelamadin F and (±)‐Tauroacidin A, Enabled by NaClO_2_‐Mediated Coupling Reactions of Oroidin and Amines

**DOI:** 10.1002/chem.71055

**Published:** 2026-04-30

**Authors:** Ryosuke Hirozumi, Yuta Kudo, Yuko Cho, Mari Yotsu‐Yamashita

**Affiliations:** ^1^ Graduate School of Agricultural Science Tohoku University Sendai Miyagi Japan; ^2^ Frontier Research Institute for Interdisciplinary Sciences Tohoku University Sendai Miyagi Japan

**Keywords:** amino acids, cross‐coupling, oxidation, total synthesis, zwitterions

## Abstract

Agelamadin F and tauroacidin A are pyrrole–imidazole alkaloids isolated from marine sponges. To the best of our knowledge, no methodology has been established for the oxidative installation of amine or pyridine moieties at the C15 position of oroidin, except for our previous total synthesis of mauritamide B, in which C15 was modified with taurine under an oxidative condition using activated carbon and molecular oxygen. In this study, we report a more effective condition for oxidative C─N coupling; sodium chlorite enables the incorporation of a broader range of amines at the C15 position of oroidin. Using this oxidant, we achieved the first total syntheses of agelamadin F and tauroacidin A, even though in low yields. In addition, nine C15–*N*–amino acid‐substituted oroidin derivatives were successfully prepared to demonstrate the applicability and versatility of this oxidative coupling reaction. Based on the structures of the products, we propose that a key structural requirement for the nucleophilic amines to react in this coupling reaction is the ability to form zwitterionic species after reaction with oroidin.

## Graphical abstract

Total syntheses of agelamadin F and (±)‐tauroacidin A were achieved by sodium chlorite (NaClO_2_)‐mediated oxidative C─N coupling. Nine C15–*N*–amino acid‐substituted oroidin derivatives were successfully prepared to demonstrate the applicability and versatility of this oxidative coupling reaction. [Correction added on May 8 2026, after first online publication: the graphical abstract has been updated.]

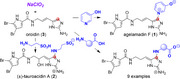

## 1 Introduction

**FIGURE 1:**
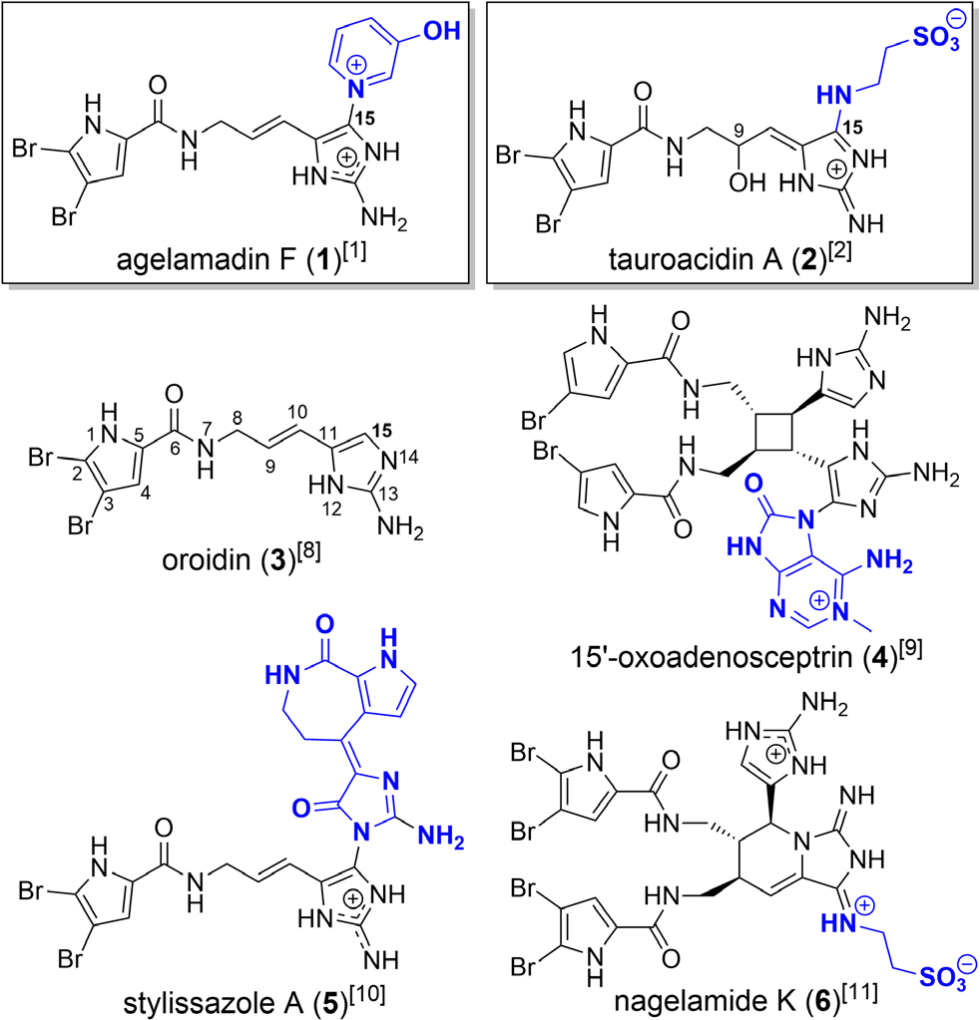
Representative bromopyrrole oroidin‐derived natural products bearing nitrogen‐containing moieties at C15 of oroidin. The charge of each compound is shown as reported.

Agelamadin F (**1**) [1] and tauroacidin A (**2**) [2] are marine natural products that belong to the dibromopyrrole alkaloid family (Figure 1). This family comprises more than 220 structural analogues, and extensive synthetic studies have been conducted on these compounds [3, 4, 5, 6]. Compounds **1** and **2** were isolated from Okinawan marine sponges of the genera *Agelas* spp. [1] and *Hymeniacidon* sp. [2], respectively, and were presumed to be biosynthesized from oroidin (**3**) [7]. Oroidin (**3**), which contains a 2‐aminoimidazole moiety (C11–C15), is a marine natural product exhibiting antimalarial activity (Figure 1) [8]. Within this compound family, there are marine natural products in which oroidin‐derived units are dimerized or a 2‐aminoimidazole structure is further substituted by a nitrogen‐bearing moiety. For example, 15'‐oxoadenosceptrin (**4**) [9], stylissazole A (**5**) [10], and nagelamide K (**6**) [11] (Figure 1) were reported from marine sponges. The biological activity of agelamadin F (**1**) has not been fully explored; however, its cationic pyridinium motif may contribute to cytotoxicity [12, 13, 14, 15]. Tauroacidin A (**2**) inhibits epidermal growth factor receptor kinase and *c‐erbB*‐2 kinase [2]. Additionally, nagelamide K (**6**) exhibited moderate antimicrobial activity [11].

**SCHEME 1:**
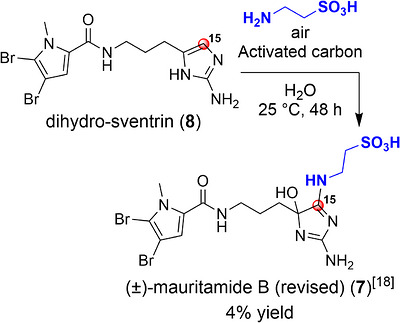
Previously reported total synthesis of (±)‐mauritamide B (**7**) from dihydro‐sventrin (**8**) developed by our group [18].

Despite the biological potency of C15–*N*‐type oroidin derivatives, detailed biological evaluations of these compounds remain limited, primarily because synthetic methods for C15–*N*‐type oroidin derivatives have not been established, except for mauritamide B (**7**) [16], which was synthesized from dihydro‐sventrin (**8**) [17] by our group (Scheme 1) [18]. In this case, we introduced taurine to the 2‐aminoimidazole moiety of dihydro‐sventrin (**8**) via oxidative modification. However, activated‐carbon‐mediated air oxidation exhibits a limited substrate scope and low yields. Consequently, structure–activity relationship (SAR) studies of substituents at the C15 position of oroidin, where the amine moiety is introduced, have not yet been conducted.

In this study, we explored more useful oxidants capable for modification of oroidin C15 with amines to convert it into agelamadin F (**1**) and (±)‐tauroacidin A (**2**), and found sodium chlorite (NaClO_2_) can be used as an oxidant for this reaction. In addition, the investigation of the reactant scope of several amines and pyridine derivatives for this reaction under optimized oxidative conditions resulted in the preparation of nine amino acid–modified oroidin derivatives at C15.

## 2 Results and Discussion

### 2.1 Synthesis of Agelamadin F (**1**)

Oroidin (**3**) was prepared following previously reported procedures [17, 19, 20, 21, 22, 23, 24, 25]. First, we selected H_2_O as the reaction solvent to convert oroidin (**3**) with 3‐hydroxypyridine into agelamadin F (**1**), because of the poor solubility of oroidin (**3**) in organic solvents. We then screened several oxidants, including ceric ammonium nitrate (CAN), 2‐iodoxybenzoic acid (IBX), Dess–Martin periodinane (DMP), N‐chlorosuccinimide (NCS), N‐bromosuccinimide (NBS), 2,2,6,6‐tetramethylpiperidine 1‐oxyl (TEMPO), oxone, sodium hypochlorite (NaClO), sodium chlorite (NaClO_2_), sodium chlorate (NaClO_3_), magnesium perchlorate (Mg(ClO_4_)_2_), and sodium periodate (NaIO_4_).

**SCHEME 2:**
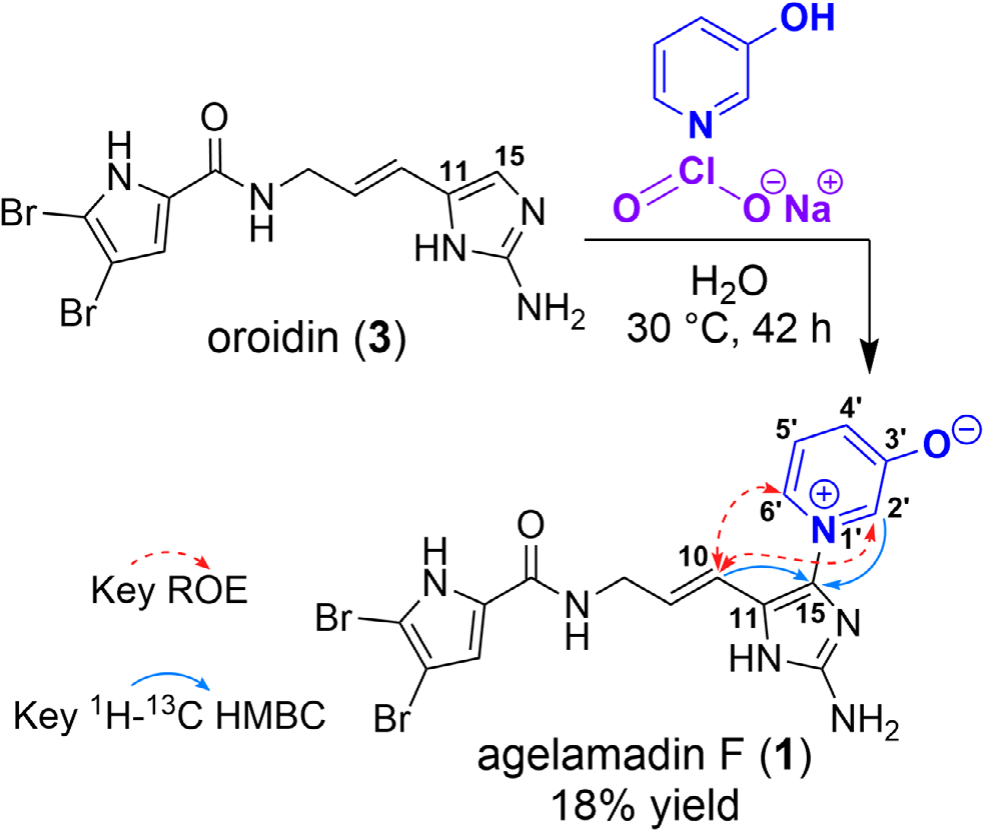
Cross‐coupling between C15 of oroidin (**3**) and N of 3‐hydroxypyridine for formation of agelamadin F (**1**) with NaClO_2_.

With oxone and Mg(ClO_4_)_2_, oroidin (**3**) was simply recovered. IBX, DMP, NCS, NBS, TEMPO, and NaIO_4_ caused oroidin (**3**) to decompose. With NaClO and NaClO_3_, trace amounts of agelamadin F (**1**) were obtained, but oroidin (**3**) was mainly recovered. In contrast, NaClO_2_ led to complete consumption of oroidin (**3**) and reproducible formation of agelamadin F (**1**). Finally, a solution of oroidin (**3**) in H_2_O was treated with excess 3‐hydroxypyridine and NaClO_2_ (46 and 48 equiv., respectively), and the mixture was stirred at 30°C for 42 h. The reaction afforded a bright yellow solid whose exact mass matched that of agelamadin F (**1**) in 18% yield (Scheme 2).

We also examined the effect of additives when NaClO_2_ was used as the oxidant. When 2‐methyl‐2‐butene was added, agelamadin F (**1**) was formed, but a portion of oroidin (**3**) was recovered, and the yield of **1** was not improved. Using trifluoroacetic acid (TFA) resulted only in the decomposition of oroidin (**3**). Adding Na_2_CO_3_ produced a small amount of agelamadin F (**1**), but in a lower yield than NaClO_2_ alone. These results show NaClO_2_ alone is the best for cross‐coupling reaction between C15 position of oroidin (**3**) and the nitrogen atom of 3‐hydroxypyridine.

Compound **1** was analyzed by ^1^H and ^13^C NMR spectroscopy in DMSO‐*d*_6_, both with and without the addition of TFA, because the chemical shifts of pyrrole–imidazole alkaloids (PIAs) might change upon the addition of TFA [26]. Upon the addition of TFA (2.0 µL, 2.6 equiv.) to a DMSO‐*d*_6_ solution of **1** (500 µL), the ^1^H and ^13^C NMR signals of the synthetic material closely matched those reported for natural **1** [1] (Table S2).

The structure of synthetic **1** was confirmed based on the NMR data (DMSO‐*d*_6_ containing TFA, COSY, TOCSY, ^1^H‐^13^C HSQC, and ^1^H‐^13^C and ^1^H‐^15^N HMBC, Figures S9–S15); the connectivity between C15 and N1' was supported by ^1^H‐^13^C HMBC correlations H10 (6.41 ppm)/C15 (134.2 ppm) and H2' (8.52 ppm)/ C15 (Figure S14). This structure was further supported by ROEs between H10 (6.41 ppm) and both H2' (8.52 ppm) and H6' (8.56 ppm) (Figure S23), probably due to the rotation of the bond between C15 and N1'.

**SCHEME 3:**
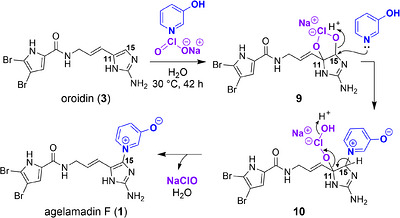
Proposed mechanism for the formation of agelamadin F (**1**) from oroidin (**3**) with NaClO_2_ and 3‐hydroxypyridine.

The proposed mechanism of the formation of agelamadin F (**1**) from oroidin (**3**) is shown in Scheme 3. In the first step, the chlorite ion (ClO_2_^–^) undergoes cycloaddition with oroidin (**3**) at the C11 and C15 positions to form bicyclic compound **9**. Subsequently, the lone‐pair electron of the N atom of 3‐hydroxypyridine nucleophilically attacks the C15 position of **9**, generating zwitterion **10**, which is finally converted to agelamadin F (**1**) via elimination of NaClO and H_2_O.

Without oroidin (**3**), when either NaClO_2_ or 3‐hydroxypyridine was separately dissolved in H_2_O and stirred at 30°C, the solution remained clear. In contrast, dissolving both NaClO_2_ and 3‐hydroxypyridine together in H_2_O and stirring at 30°C resulted in a pale‐yellow solution, whose odor and color suggested the formation of chlorine dioxide (ClO_2_) according to a literature [27].

In addition, when oroidin (**3**) was treated with NaClO_2_ alone (in the absence of 3‐hydroxypyridine), it decomposed, and no oxidized products were detected, in contrast to the activated carbon and O_2_ oxidation used for synthesis of mauritamide B (**7**), in which producing the oxygenated byproducts of dihydro‐sventrin (**8**) [18]. Notably, in the oxidative coupling reaction using NaClO_2_, the reaction proceeded even under an argon atmosphere after degassing the dissolved oxygen in H_2_O by argon bubbling, indicating that the reaction is not influenced by molecular oxygen from air.

### 2.2 Synthesis of Tauroacidin A (**2**)

**SCHEME 4:**
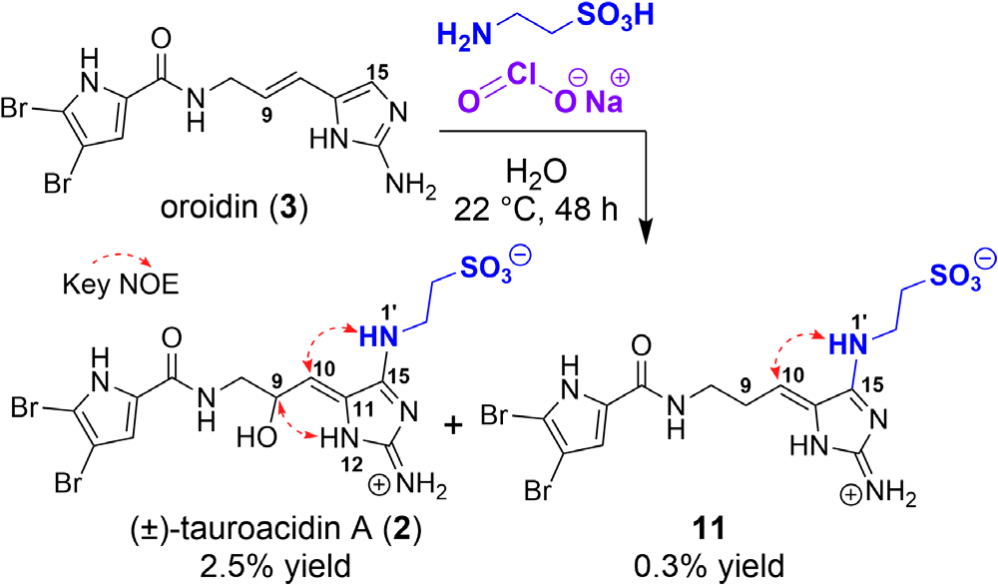
Cross‐coupling between C15 of oroidin (**3**) and N atom of taurine for the synthesis of (±)‐tauroacidin A (**2**) with NaClO_2_.

Next, we applied a NaClO_2_‐mediated oxidative coupling reaction to the transformation of **3** with taurine. The taurine‐insertion reaction was carried out using taurine and NaClO_2_ (522 and 192 equiv., respectively) in water at 22°C for 48 h. Consequently, a trace amount of (±)‐tauroacidin A (**2**) was obtained in 2.5% yield (Scheme 4). The reaction temperature was also critical; unlike the synthesis of agelamadin F (**1**), raising the temperature to 30°C resulted in no product formation, and oroidin (**3**) decomposed instead.

The structure of the synthetic **2** was confirmed based on NMR data (^1^H NMR, ^13^C NMR, COSY, TOCSY, ^1^H‐^13^C and ^1^H‐^15^N HSQC, and ^1^H‐^13^C HMBC, Figures S26–S32) obtained in DMSO‐*d*_6_ containing TFA. The ^1^H and ^13^C data for **2** were in good agreement with those reported for tauroacidin A (**2**) [2] (Table S3). The differences of the chemical shifts between synthetic **2** and those reported were within 0.04 ppm for ^1^H NMR signals, and within 0.2 ppm for ^13^C NMR. Furthermore, analysis of the NOESY spectrum of synthetic **2** showed an NOE correlation between H9 (4.60 ppm) and H12 (10.55 ppm), and between H10 (6.16 ppm) and H1' (9.66 ppm), supporting that **2** exists as the *Z*‐isomer (Figure S33). Taken together, these results demonstrate that this study achieved the first total synthesis of (±)‐tauroacidin A (**2**), although the yield was very low. In addition, compound **11** was isolated as a byproduct in 0.3% yield (Figure S36−S46). In this reaction, (±)‐tauroacidin A (**2**) is presumed to be formed from oroidin (**3**) through a pathway nearly identical to that shown in Scheme 3 (Scheme S10). Hydroxylation at C9 position of intermediate **11** probably occurred during the formation of tauroacidin A (**2**). In this reaction, the starting material oroidin (**3**) was barely recovered.

### 2.3 Substrate Scope of Nucleophiles

Next, we investigated the substrate scope of amines as nucleophiles in the NaClO_2_ oxidative coupling of **3**. As described above, both 3‐hydroxypyridine and taurine successfully participate in this reaction and form zwitterions. Therefore, 22 amino acids were tested as representative nucleophilic amines to determine the substrate scope for the oxidative coupling reaction. As a result, glycine (Gly), β‐alanine (β‐Ala), γ‐aminobutyric acid (GABA), l‐alanine (Ala), l‐valine (Val), l‐isoleucine (Ile), l‐proline (Pro), and l‐phenylalanine (Phe) were reproducibly introduced at the C15 position of **3** via oxidative coupling. l‐Leucine (Leu) was also introduced at the C15 position of **3**; however, the reaction showed low reproducibility and yielded nearly 1%. The structures of all the compounds synthesized from amino acids and **3** were determined to possess the C10/C11 *Z*‐configuration by NOESY or NOESY‐1D spectra (Figures S57, S68, S79, S90, S101, S106, S117, S126, S127, S138), similar to **2** and **11**. The yields were generally low and ranged from 1% to 17% (Table 1). In these reactions, a large excess (552–1449 equiv.) of the corresponding amino acid was required to react with **3**, and C9–OH species were detected only in trace amounts (Scheme S14).

**TABLE 1:** Reactant scope of amino acids for cross‐coupling of C15 of oroidine (**3**) with NaClO_2_.

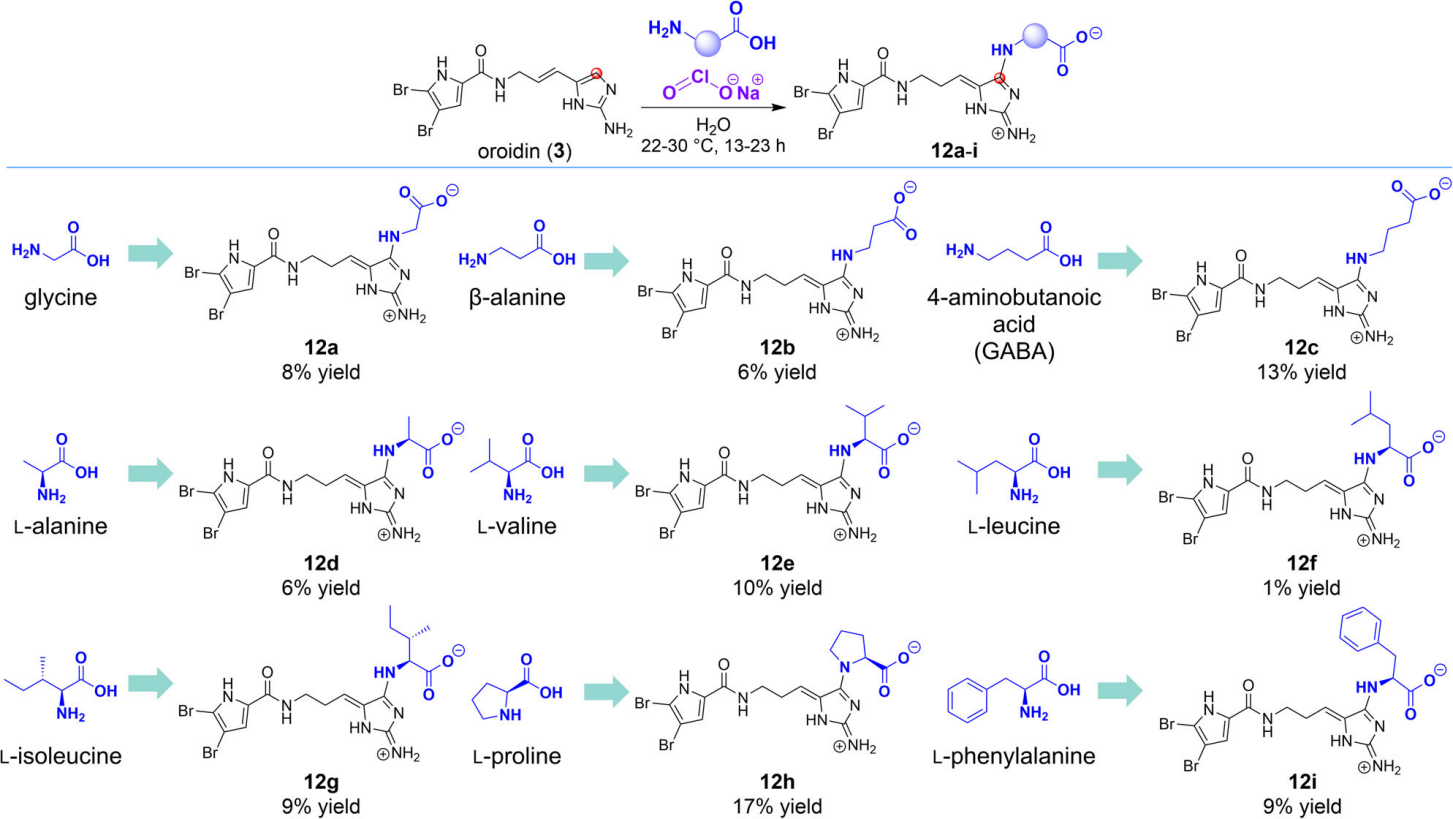

Similar reactions with other amino acids did not yield the expected products, or the yield was less than 1%. Details are presented in Table S4. Based on this result, the ability to form a zwitterionic species without other functional groups is suggested to be necessary for reactions in which amino acids are introduced at the C15 position of oroidin (**3**).

We examined the syntheses of **2** and **12a** using NaClO, NaClO_3_, and Mg(ClO_4_)_2_ as alternative oxidants. Only NaClO_2_ produced the desired products. We anticipate that NaClO_2_ would likewise be optimal for reactions involving other amino acids.

We also investigated the scope of pyridine‐type nucleophiles in the cross‐coupling by testing 2‐hydroxypyridine and 4‐hydroxypyridine in addition to 3‐hydroxypyridine in the presence of oroidin (**3**) and NaClO_2_. No coupling products were detected in these reactions, and **3** underwent decomposition (Scheme S11). We assume that 2‐hydroxypyridine and 4‐hydroxypyridine existed in equilibrium with 2‐pyridone and 4‐pyridone tautomers, respectively, in solution [28], whereas 3‐hydroxypyridine adopts four distinct forms—neutral, dipolar, cationic, and anionic—depending on the solvent (Scheme S12) [29]. The formation of this zwitterionic species may also enhance the stability of the resulting product by increasing intramolecular electronic delocalization.

### 2.4 Analyses of By‐Products

During the syntheses of agelamadin F (**1**), tauroacidin A (**2**), and compounds **12a**–**i**, we obtained large amounts of brown by‐products that strongly adsorbed onto Silica Gel 60 (spherical) NH_2_. In the crude reaction mixture for agelamadin F (**1**), neither oroidin (**3**) nor other by‐products were detected by MS spectrum, suggesting that approximately 80% of the material converted into these brown by‐products under the NaClO_2_/nucleophile reactions (Scheme S13). During the synthesis of tauroacidin A (**2**), we could not determine structures of by‐products by NMR due to difficulty of the purification, we assumed their structures based on MS spectra (Scheme S14). The predicted structure of the by‐products detected by MS spectra during the syntheses of compounds **12a**–**i** are also summarized in Scheme S15. Formation of unidentified by‐products and decreased yields has often been reported in transformations of compounds containing 2‐aminoimidazole moieties [30, 31, 32, 33].

The reaction with taurine yielded more of the C9–OH product, tauroacidin A (**2**), than the C9–H analogue, compound **11**. In contrast, reactions with amino acids mainly gave C9–H products (compounds **12a–i**). Only trace amounts of C9–OH species were detected by MS. We assumed that larger bulkiness of amino acids compared with that of taurine may cause this difference.

## 3 Conclusion

We achieved the first total syntheses of agelamadin F (**1**) and (±)‐tauroacidin A (**2**) by promoting a cross‐coupling reaction between the C15 position of the 2‐aminoimidazole moiety in oroidin (**3**) and the N atom of 3‐hydroxypyridine or taurine, using NaClO_2_ as the oxidant, although the yields were low. In addition, the investigation of the reactant amine scope as nucleophiles in the NaClO_2_ oxidation coupling reaction revealed that neutral L‐type amino acids commonly found in proteins, as well as β‐Ala and GABA, could be introduced at the C15 position of oroidin (**3**) in 1%–17% yields. All the obtained products possessed the C10‐C11 *Z*‐configuration. Notably, this reaction is a rare example of direct cross‐coupling between the C(*sp* ^2^) of 2‐aminoimidazole and the N atom of a compound containing an acidic functional group.

## 4 Experimental Section

### 4.1 Materials and Methods

Dry solvents for organic syntheses were purchased from FUJIFILM Wako Pure Chemical Corporation (Osaka, Japan). Other reagents were purchased from Sigma‐Aldrich (St. Louis, MO, USA), Tokyo Chemical Industry Co., Ltd. (Tokyo, Japan), Nacalai Tesque, Inc. (Kyoto, Japan), and Ambeed Inc. (Arlington, Texas, USA). LC/MS‐grade methanol (Kanto Chemical, Tokyo, Japan) was used for HR‐ESI‐MS. Distilled and purified water (MilliQ) by Simplicity UV (Merck Millipore Corporation, Billerica, MA, USA) was used for all experiments. Compounds were monitored by TLC using Silica gel 60 F_254_ plates (Merck & Co., Inc., New Jersey, US) for R*_f_* values and spots were visualized under UV (254 nm). FT‐IR spectra (film: ATR, Zn‐Se) were measured using a JASCO FT/IR‐4600 spectrometer (JASCO, Tokyo, Japan). UV absorption spectra were measured by UV‐1800 (Shimadzu Corporation, Kyoto, Japan). Optical rotation values were measured with a Jasco P‐2200 polarimeter. NMR spectra were recorded with an Agilent 600 MHz NMR spectrometer (Agilent Technologies, Inc., Santa Clara, CA, USA) with 5 mm ID Probe and a Bruker AVANCE NEO 600 (Bruker, Billerica, MA, USA) with 5 mm Probe. CD_3_OD or DMSO‐*d*_6_ were used as solvents. Chemical shifts were referenced to residual solvent signals at *δ*_H/C_ 3.30/49.8 ppm (CD_3_OD) and 2.50/39.5 ppm (DMSO‐*d*_6_). ^1^H, ^13^C, and ^15^N NMR were measured at 600, 151, and 60.8 MHz, respectively. High‐resolution electrospray ionization mass spectrometry (HR‐ESI‐MS) spectra were obtained using a micrOTOF‐Q II (Bruker Daltonics Inc., Billerica, MA, USA) equipped with an ESI ion source. Mass spectrometer API2000 (Sciex, Framingham, MA, USA) was also used for the identification of the synthetic products.

Sodium chlorite used in this study was purchased from Sigma‐Aldrich (product Nos. 244155‐5G and 244155‐100G). This reagent was technical grade 80% (purity). Although the impurities in this material were not completely specified, technical support indicated that the product contains sodium carbonate (1%–5%, most likely around 4%) as well as NaClO_3_, NaCl, Na_2_CO_3_, Na_2_SO_4_, H_2_O, and trace amounts of H_2_O_2_.

Oroidin (**3**) was prepared following previously reported procedures [17, 19–25]. The crude oroidin (**3**) was filtered through a Cosmospin Filter H (0.45 µm; Nacalai Tesque, Inc., Kyoto, Japan) and purified by reversed phase (RP)‐HPLC (InertSustain AQ‐C18, 5 µm, 10 mm i.d. × 250 mm; GL Sciences) using gradient elution (0–4 min, MeOH/H_2_O = 50:50, v/v; 4–40 min, 50:50 to 90:10) at a flow rate of 2.0 mL/min. Pure oroidin (**3**) was obtained at 21–29 min as a white to slightly yellow solid.

### 4.2 Synthesis of Agelamadin F (**1**)

Ten batches of oroidin (**3**) (HCOOH salt, 2.0 mg, 0.0046 mmol each; 20 mg, 0.046 mmol in total) were placed in 1 mL Reacti‐Vials (Thermo Fisher Scientific K.K.), and H_2_O (300 µL) was added to each vial. Subsequently, 3‐hydroxypyridine (20.0 mg, 0.21 mmol, 46 equiv.) and NaClO_2_ (20.0 mg, 0.22 mmol, 48 equiv.) were added. The vials were tightly sealed, vortexed until the stir bars rotated smoothly, and then heated at 30°C. After stirring for 42 h, all reaction mixtures were combined and directly purified by RP column chromatography using Cosmosil 140C_18_–OPN (2.0 g) and MeOH/H_2_O/TFA (0:100:0.1 to 75:25:0.1, v/v/v; 50 mL per fraction). The crude agelamadin F (**1**) was further purified by silica gel column chromatography (Silica Gel 60, spherical, NH_2_, 40–50 µm, equilibrated with EtOAc) using MeOH (30 mL) as the eluent. The obtained agelamadin F (**1**) was filtered through a Cosmospin Filter H (0.45 µm; Nacalai Tesque, Inc., Kyoto, Japan) and purified by RP‐HPLC (InertSustain AQ‐C18, 5 µm, 10 mm i.d. × 250 mm; GL Sciences) using gradient elution (0–4 min, MeOH/H_2_O = 50:50, v/v; 4–40 min, 50:50 to 100:0) at a flow rate of 2.0 mL/min. Pure agelamadin F (**1**) was obtained at 19–25 min (4.91 mg, 0.00824 mmol, 18% yield) as a yellow to orange solid. HRESIMS [M+H]^+^ (*m/z*) calcd for C_16_H_15_^79^Br_2_N_6_O_2_^+^ 480.9618, found 480.9608. Properties: see page S1 in SI. NMR, UV, and IR spectra: see Tables S1–S2 and Figures S1–S25.

### 4.3 Synthesis of Tauroacidin A (**2**) and Compound **11**

Fifteen batches of oroidin (**3**) (HCOOH salt, 1.0 mg, 0.0023 mmol each; 15 mg, 0.034 mmol in total) were placed in 4 mL vials. Taurine (150.0 mg, 1.20 mmol, 522 equiv.) and H_2_O (4.0 mL) were added to each vial, and the mixtures were stirred until taurine was completely dissolved. NaClO_2_ (40.0 mg, 0.44 mmol, 192 equiv.) was then added, and the reaction mixtures were stirred at 22°C for 48 h. After completion, all reaction mixtures were combined and directly purified by RP column chromatography using Cosmosil 140C_18_–OPN (3.0 g) and MeOH/H_2_O (0:100 to 100:0, v/v). The MeOH fraction was concentrated under reduced pressure, and one drop of TFA was added. The crude material was filtered through a Cosmospin Filter H (0.45 µm) and further purified by RP‐HPLC (InertSustain AQ‐C18, 5 µm, 10 mm i.d. × 250 mm; GL Sciences) using isocratic elution (MeOH/H_2_O/HCOOH = 35:65:0.1, v/v/v) at a flow rate of 2.0 mL/min. Pure (±)‐tauroacidin A (**2**) was obtained as HCOOH salt at 76–86 min (0.48 mg, 0.00084 mmol, 2.5% yield) as a white to slightly orange film. HRESIMS [M+Na]^+^ (*m/z*) calcd for C_13_H_16_^79^Br^81^BrN_6_NaO_5_S^+^: 550.9142, found 550.9147. Properties: see page S29. NMR, UV, and IR spectra: see Table S3 and Figures S26–S35.

For the purification of compound **11**, a semi‐purified mixture of (±)‐tauroacidin A (**2**) and **11** (obtained after RP‐HPLC purification from seventy batches of oroidin (**3**), 0.161 mmol in total, without the addition of TFA) was filtered through a Cosmospin Filter H (0.45 µm) and further purified by RP‐HPLC (InertSustain AQ‐C18, 5 µm, 10 mm i.d. × 250 mm; GL Sciences) using isocratic elution with MeOH/H_2_O/HCOOH = 35:65:0.1 (v/v/v) at a flow rate of 2.0 mL/min. Pure **11** was obtained as the HCOOH salt at 90–102 min (0.30 mg, 0.000538 mmol, 0.3% yield) as a white film. HRESIMS [M+H]^+^ (*m/z*) calcd for C_13_H_17_^79^Br_2_N_6_O_5_S^+^ [M+H]^+^: 510.9393, found 510.9369. Properties: see page S41. NMR, UV, and IR spectra: see Figures S36–S48.

### 4.4 Synthesis of **12a–i**

Oroidin (**3**) (HCOOH salt, 3.0 mg, 0.0069 mmol) was placed in a 20 mL round‐bottomed flask, and H_2_O (4.0 mL) was added with stirring. An amino acid was then added to the mixture, followed by addition of NaClO_2_ (120 mg, 1.33 mmol, 193 equiv.). The flask was sealed with a septa cap, and the reaction mixture was stirred. After completion, the reaction mixture was filtered through a small pad of Celite, rinsing the flask and filter cake with H_2_O. The filtrate was directly purified by 140C_18_–OPN (MeOH/H_2_O). The eluate was concentrated under reduced pressure, and the crude material was filtered through a Cosmospin filter H (0.45 µm). Further purification was performed by RP‐HPLC (InertSustain AQ‐C18, 5 µm, 10 mm i.d. × 250 mm; GL Science) using MeOH/H_2_O/HCOOH as the eluent. Detailed synthetic procedures: see Scheme S1–S9. Properties: see pages S56, S69, S82, S95, S108, S121, S128, S141, and S153. NMR, UV, and IR spectra: see Figures S49–140.

## Conflicts of Interest

The authors declare no conflicts of interest.

## Supporting information




**Supporting file**: The data that support the findings of this study are available in the Supporting information of this article. The supporting information includes properties of compounds **1**, **2**, **11**, and **12a–i**; ^1^H NMR spectra of compounds **1**, **2**, **11**, and **12a–i**; ^13^C NMR spectra of compounds **1**, **2**, **12a–e**, and **12g–i**; COSY spectra of compounds **1**, **2**, **11**, and **12a–i**; TOCSY spectra of compounds **1**, **2**, **11**, and **12a–i**; ^1^H‐^13^C HSQC spectra of compounds **1**, **2**, **11**, **12a–e**, and **12g–i**; ^1^H‐^13^C HMBC spectra of compounds **1**, **2**, **11**, **12a–e**, and **12g–i**; ^1^H‐^15^N HSQC spectrum of compounds **1**, **2**; ^1^H‐^15^N HMBC spectrum of compound **1**; NOESY spectra of compounds **2**, **11**, **12a–g**, and **12i**; NOESY‐1D spectra of compounds **12h**; ROESY‐1D spectra of compounds **1**; UV absorption spectra of compounds **1**, **2**, **11**, and **12a–i**; IR spectra of compounds of **1**, **2**, **11**, and **12a–i**; Synthetic procedures **12a–i**; Comparison of synthetic compound **1** and natural agelamadin F; Comparison of synthetic compound **2** and natural tauroacidin A.

## Data Availability

The data that support the findings of this study are available on request from the corresponding author. The data are not publicly available due to privacy or ethical restrictions.
