# Supplementary material for: Total Syntheses of Agelamadin F and (±)‐Tauroacidin A, Enabled by NaClO2‐Mediated Coupling Reactions of Oroidin and Amines
Source: Chemistry. 2026 Apr 30;32(27):e71055. doi: 10.1002/chem.71055 (PMC13380358; doi:10.1002/chem.71055)
Supplement: Supplementary file 1 — Supporting file: The data that support the findings of this study are available in the Supporting information of this article. The supporting information includes properties of compounds 1, 2, 11, and 12a–i; 1H NMR spectra of compounds 1, 2, 11, and 12a–i; 13C NMR spectra of compounds 1, 2, 12a–e, and 12g–i; COSY spectra of compounds 1, 2, 11, and 12a–i; TOCSY spectra of compounds 1, 2, 11, and 12a–i; 1H‐13C HSQC spectra of compounds 1, 2, 11, 12a–e, and 12g–i; 1H‐13C HMBC spectra of compounds 1, 2, 11, 12a–e, and 12g–i; 1H‐15N HSQC spectrum of compounds 1, 2; 1H‐15N HMBC spectrum of compound 1; NOESY spectra of compounds 2, 11, 12a–g, and 12i; NOESY‐1D spectra of compounds 12h; ROESY‐1D spectra of compounds 1; UV absorption spectra of compounds 1, 2, 11, and 12a–i; IR spectra of compounds of 1, 2, 11, and 12a–i; Synthetic procedures 12a–i; Comparison of synthetic compound 1 and natural agelamadin F; Comparison of synthetic compound 2 and natural tauroacidin A. [file CHEM-32-e71055-s001.pdf]

Supporting Information

Total Syntheses of Agelamadin F and (±)-Tauroacidin A, Enabled by $NaClO_2$-Mediated Coupling Reactions of Oroidin and Amines

Ryosuke Hirozumi,[1] Yuta Kudo,[2,1] Yuko Cho,[1] Mari Yotsu-Yamashita[1,*]

[1] Graduate School of Agricultural Science, Tohoku University, 468-1 Aramaki-Aza-Aoba, Aoba-ku, Sendai, Miyagi 980-8572, Japan

[2] Frontier Research Institute for Interdisciplinary Sciences, Tohoku University, 6-3 AramakiAza-Aoba, Aoba-ku, Sendai, Miyagi 980-8578, Japan

	Contents	Page
	Properties of agelamadin F (**1**).	S1
**Table S1**	^{1}H (600 MHz), ^{13}C (151 MHz), and ^{15}N (60.8 MHz) NMR data for **1**, and reported data for **1** in DMSO-d_6 (δ in ppm).	S2
**Table S2**	^{1}H (600 MHz), ^{13}C (151 MHz), and ^{15}N (60.8 MHz) NMR data for **1** (DMSO-d_6: 500 μL; TFA: 2.0 μL), and reported data for **1** (DMSO-d_6) (δ in ppm).	S3
**Figure S1**	Comparison of ^{1}H and ^{13}C NMR chemical shift values of agelamadin F (**1**) before and after the addition of TFA. Chemical shifts of ^{1}H NMR (black), ^{13}C NMR (red), ^{15}N NMR (blue) are shown.	S4
**Figure S2**	^{1}H NMR spectrum of **1** (4.91 mg) (600 MHz, DMSO-d_6: 550 μL).	S5
**Figure S3**	^{13}C NMR spectrum of **1** (4.25 mg) (151 MHz, DMSO-d_6: 500 μL).	S6
**Figure S4**	COSY spectrum of **1** (4.25 mg) (600 MHz, DMSO-d_6: 500 μL).	S7
**Figure S5**	TOCSY spectrum of **1** (4.25 mg) (600 MHz, DMSO-d_6: 500 μL).	S8
**Figure S6**	^{1}H-^{13}C HSQC spectrum of **1** (4.25 mg) (600 MHz / 151 MHz, DMSO-d_6: 500 μL).	S9
**Figure S7**	^{1}H-^{13}C HMBC spectrum of **1** (4.25 mg) (600 MHz / 151 MHz, DMSO-d_6: 500 μL).	S10
**Figure S8**	^{1}H-^{15}N HSQC spectrum of **1** (4.91 mg) (600 MHz / 60.8 MHz, DMSO-d_6: 550 μL).	S11
**Figure S9**	^{1}H NMR spectrum of **1** (4.91 mg) (600 MHz, DMSO-d_6: 500 μL; TFA: 2.0 μL).	S12
**Figure S10**	^{13}C NMR spectrum of **1** (4.91 mg) (151 MHz, DMSO-d_6: 500 μL; TFA: 2.0 μL).	S13
**Figure S11**	COSY spectrum of **1** (4.91 mg) (600 MHz, DMSO-d_6: 500 μL; TFA: 2.0 μL).	S14
**Figure S12**	TOCSY spectrum of **1** (4.91 mg) (600 MHz, DMSO-d_6: 500 μL; TFA: 2.0 μL).	S15
**Figure S13**	^{1}H-^{13}C HSQC spectrum of **1** (4.91 mg) (600 MHz / 151 MHz, DMSO-d_6: 500 μL; TFA: 2.0 μL).	S16
**Figure S14**	^{1}H-^{13}C HMBC spectrum of **1** (4.91 mg) (600 MHz / 151 MHz, DMSO-d_6: 500 μL; TFA: 2.0 μL).	S17
**Figure S15**	^{1}H-^{15}N HMBC spectrum of **1** (4.91 mg) (600 MHz / 60.8 MHz, DMSO-d_6: 500 μL; TFA: 2.0 μL).	S18
**Figure S16**	Comparison of ^{1}H NMR spectra showing stepwise addition of TFA (0, 0.25 μL, 0.50 μL, 0.75 μL, 1.0 μL) to agelamadin F (4.25 mg) in DMSO-d_6 (500 μL) (600 MHz).	S19
**Figure S17**	ROESY-1D spectrum of **1** (4.91 mg); irradiation at 11.79 ppm (600 MHz, DMSO-d_6: 500 μL; TFA: 2.0 μL).	S20
**Figure S18**	ROESY-1D spectrum of **1** (4.91 mg); irradiation at 8.56 ppm (600 MHz, DMSO-d_6: 500 μL; TFA: 2.0 μL).	S21
**Figure S19**	ROESY-1D spectrum of **1** (4.91 mg); irradiation at 8.52 ppm (600 MHz, DMSO-d_6: 500 μL; TFA: 2.0 μL).	S22
**Figure S20**	ROESY-1D spectrum of **1** (4.91 mg); irradiation at 8.40 ppm (600 MHz, DMSO-d_6: 500 μL; TFA: 2.0 μL).	S23
**Figure S21**	ROESY-1D spectrum of **1** (4.91 mg); irradiation at 7.98 ppm (600 MHz, DMSO-d_6: 500 μL; TFA: 2.0 μL).	S24
**Figure S22**	ROESY-1D spectrum of **1** (4.91 mg); irradiation at 6.94 ppm (600 MHz, DMSO-d_6: 500 μL; TFA: 2.0 μL).	S25

**Figure S23**	ROESY-1D spectrum of **1** (6.35 mg); irradiation at 6.41 ppm (600 MHz, DMSO-d_6: 500 µL; TFA: 2.5 µL).	S26
**Figure S24**	UV absorption spectrum of agelamadin F (**1**) (MeOH). c = 1.1 x 10^{-4} (M)	S27
**Figure S25**	IR spectrum of **1** (ATR).	S28
	Properties of (±)-tauroacidin A (**2**).	S29
**Table S3**	^{1}H (600 MHz), ^{13}C (151 MHz) and ^{15}N (60.8 MHz) NMR data for **2** containing TFA, and reported data for **2** (*δ* in ppm).	S30
**Figure S26**	^{1}H NMR spectrum of **2** (1.09 mg) (600 MHz, DMSO-d_6: 190 µL - 0.1% TFA).	S31
**Figure S27**	^{13}C NMR spectrum of **2** (2.61 mg) (151 MHz, DMSO-d_6: 550 µL - 0.5% TFA).	S32
**Figure S28**	COSY spectrum of **2** (1.09 mg) (600 MHz, DMSO-d_6: 190 µL - 0.1% TFA).	S33
**Figure S29**	TOCSY spectrum of **2** (1.09 mg) (600 MHz, DMSO-d_6: 190 µL - 0.1% TFA).	S34
**Figure S30**	^{1}H-^{13}C HSQC spectrum of **2** (1.09 mg) (600 MHz / 151 MHz, DMSO-d_6: 190 µL - 0.1% TFA).	S35
**Figure S31**	^{1}H-^{13}C HMBC spectrum of **2** (1.09 mg) (600 MHz / 151 MHz, DMSO-d_6: 190 µL - 0.1% TFA).	S36
**Figure S32**	^{1}H-^{15}N HMBC spectrum of **2** (1.09 mg) (600 MHz / 60.8 MHz, DMSO-d_6: 190 µL - 0.1% TFA).	S37
**Figure S33**	NOESY spectrum of **2** (1.09 mg) (600 MHz, DMSO-d_6: 190 µL - 0.1% TFA).	S38
**Figure S34**	UV absorption spectrum of tauroacidin A (**2**) (MeOH). c = 9.5 x 10^{-5} (M)	S39
**Figure S35**	IR spectrum of **2** (ATR).	S40
	Properties of **11**.	S41
**Figure S36**	^{1}H NMR spectrum of **11** (0.30 mg) (600 MHz, CD_3OD: 190 µL).	S42
**Figure S37**	COSY spectrum of **11** (0.30 mg) (600 MHz, CD_3OD: 190 µL).	S43
**Figure S38**	TOCSY spectrum of **11** (0.30 mg) (600 MHz, CD_3OD: 190 µL).	S44
**Figure S39**	^{1}H-^{13}C HSQC spectrum of **11** (0.30 mg) (600 MHz /151 MHz, CD_3OD: 190 µL).	S45
**Figure S40**	^{1}H-^{13}C HMBC spectrum of **11** (0.30 mg) (600 MHz /151 MHz, CD_3OD: 190 µL).	S46
**Figure S41**	^{1}H NMR spectrum of **11** (0.30 mg) (600 MHz, DMSO-d_6: 180 µL - 0.1% TFA).	S47
**Figure S42**	COSY spectrum of **11** (0.30 mg) (600 MHz, DMSO-d_6: 180 µL - 0.1% TFA).	S48
**Figure S43**	TOCSY spectrum of **11** (0.30 mg) (600 MHz, DMSO-d_6: 180 µL - 0.1% TFA).	S49
**Figure S44**	^{1}H-^{13}C HSQC spectrum of **11** (0.30 mg) (600 MHz / 151 MHz, DMSO-d_6: 180 µL - 0.1% TFA).	S50

Figure S45 ^{1}H-^{13}C HMBC spectrum of **11** (0.30 mg) (600 MHz / 151 MHz, DMSO-d_6: 180 μL - 0.1% TFA). S51

Figure S46 NOESY spectrum of **11** (0.30 mg) (600 MHz, DMSO-d_6: 180 μL - 0.1% TFA). S52

Figure S47 UV absorption spectrum of **11** (MeOH). c = 1.35 x 10^{-4} (M) S53

Figure S48 IR spectrum of **11** (ATR). S54

Scheme S1 Synthesis of **12a**. S55

Properties of **12a**. S56

Figure S49 ^{1}H NMR spectrum of **12a** (1.03 mg) (600 MHz, CD_3OD: 500 μL - 0.1% TFA). S57

Figure S50 ^{13}C NMR spectrum of **12a** (1.73 mg) (151 MHz, CD_3OD: 550 μL - 0.1% TFA). S58

Figure S51 COSY spectrum of **12a** (1.03 mg) (600 MHz, CD_3OD: 500 μL - 0.1% TFA). S59

Figure S52 TOCSY spectrum of **12a** (1.03 mg) (600 MHz, CD_3OD: 500 μL - 0.1% TFA). S60

Figure S53 ^{1}H-^{13}C HSQC spectrum of **12a** (1.03 mg) (600 MHz/151 MHz, CD_3OD: 500 μL - 0.1% TFA). S61

Figure S54 ^{1}H-^{13}C HMBC spectrum of **12a** (1.03 mg) (600 MHz/151 MHz, CD_3OD: 500 μL - 0.1% TFA). S62

Figure S55 ^{1}H NMR spectrum of **12a** (0.98 mg) (600 MHz, DMSO-d_6: 500 μL - 0.1% TFA). S63

Figure S56 COSY spectrum of **12a** (0.98 mg) (600 MHz, DMSO-d_6: 500 μL - 0.1% TFA). S64

Figure S57 NOESY spectrum of **12a** (0.98 mg) (600 MHz, DMSO-d_6: 500 μL - 0.1% TFA). S65

Figure S58 UV absorption spectrum of **12a** (MeOH). c = 1.36 x 10^{-4} (M) S66

Figure S59 IR spectrum of **12a** (ATR). S67

Scheme S2 Synthesis of **12b**. S68

Properties of **12b**. S69

Figure S60 ^{1}H NMR spectrum of **12b** (1.03 mg) (600 MHz, CD_3OD: 500 μL - 0.1% TFA). S70

Figure S61 ^{13}C NMR spectrum of **12b** (2.44 mg) (151 MHz, CD_3OD: 500 μL - 0.1% TFA). S71

Figure S62 COSY spectrum of **12b** (1.03 mg) (600 MHz, CD_3OD: 500 μL - 0.1% TFA). S72

Figure S63 TOCSY spectrum of **12b** (1.03 mg) (600 MHz, CD_3OD: 500 μL - 0.1% TFA). S73

Figure S64 ^{1}H-^{13}C HSQC spectrum of **12b** (1.03 mg) (600 MHz/151 MHz, CD_3OD: 500 μL - 0.1% TFA). S74

Figure S65 ^{1}H-^{13}C HMBC spectrum of **12b** (1.03 mg) (600 MHz/151 MHz, CD_3OD: 500 μL - 0.1% TFA). S75

**Figure S66**	^{1}H NMR spectrum of **12b** (1.08 mg) (600 MHz, DMSO-d_6: 500 μL - 0.1% TFA).	S76
**Figure S67**	COSY spectrum of **12b** (1.08 mg) (600 MHz, DMSO-d_6: 500 μL - 0.1% TFA).	S77
**Figure S68**	NOESY spectrum of **12b** (1.08 mg) (600 MHz, DMSO-d_6: 500 μL - 0.1% TFA).	S78
**Figure S69**	UV absorption spectrum of **12b** (MeOH). c = 1.32 x 10^{-4} (M)	S79
**Figure S70**	IR spectrum of **12b** (ATR).	S80
**Scheme S3**	Synthesis of **12c**.	S81
	Properties of **12c**.	S82
**Figure S71**	^{1}H NMR spectrum of **12c** (2.14 mg) (600 MHz, CD_3OD: 500 μL - 0.1% TFA).	S83
**Figure S72**	^{13}C NMR spectrum of **12c** (2.14 mg) (151 MHz, CD_3OD: 500 μL - 0.1% TFA).	S84
**Figure S73**	COSY spectrum of **12c** (2.14 mg) (600 MHz, CD_3OD: 500 μL - 0.1% TFA).	S85
**Figure S74**	TOCSY spectrum of **12c** (2.14 mg) (600 MHz, CD_3OD: 500 μL - 0.1% TFA).	S86
**Figure S75**	^{1}H-^{13}C HSQC spectrum of **12c** (2.14 mg) (600 MHz /151 MHz, CD_3OD: 500 μL - 0.1% TFA).	S87
**Figure S76**	^{1}H-^{13}C HMBC spectrum of **12c** (2.14 mg) (600 MHz /151 MHz, CD_3OD: 500 μL - 0.1% TFA).	S88
**Figure S77**	^{1}H NMR spectrum of **12c** (2.08 mg) (600 MHz, DMSO-d_6: 500 μL - 0.1% TFA).	S89
**Figure S78**	COSY spectrum of **12c** (2.08 mg) (600 MHz, DMSO-d_6: 500 μL - 0.1% TFA).	S90
**Figure S79**	NOESY spectrum of **12c** (2.08 mg) (600 MHz, DMSO-d_6: 500 μL - 0.1% TFA).	S91
**Figure S80**	UV absorption spectrum of **12c** (MeOH). c = 1.28 x 10^{-4} (M)	S92
**Figure S81**	IR spectrum of **12c** (ATR).	S93
**Scheme S4**	Synthesis of **12d**.	S94
	Properties of **12d**.	S95
**Figure S82**	^{1}H NMR spectrum of **12d** (1.52 mg) (600 MHz, CD_3OD: 500 μL - 0.1% TFA).	S96
**Figure S83**	^{13}C NMR spectrum of **12d** (1.52 mg) (151 MHz, CD_3OD: 500 μL - 0.1% TFA).	S97
**Figure S84**	COSY spectrum of **12d** (1.52 mg) (600 MHz, CD_3OD: 500 μL - 0.1% TFA).	S98
**Figure S85**	TOCSY spectrum of **12d** (1.52 mg) (600 MHz, CD_3OD: 500 μL - 0.1% TFA).	S99
**Figure S86**	^{1}H-^{13}C HSQC spectrum of **12d** (1.52 mg) (600 MHz / 151 MHz, CD_3OD: 500 μL - 0.1% TFA).	S100

**Figure S87**	^{1}H-^{13}C HMBC spectrum of **12d** (1.52 mg) (600 MHz / 151 MHz, CD_3OD: 500 µL - 0.1% TFA).	S101
**Figure S88**	^{1}H NMR spectrum of **12d** (1.52 mg) (600 MHz, DMSO-d_6: 500 µL - 0.1% TFA).	S102
**Figure S89**	COSY spectrum of **12d** (1.52 mg) (600 MHz, DMSO-d_6: 500 µL - 0.1% TFA).	S103
**Figure S90**	NOESY spectrum of **12d** (1.52 mg) (600 MHz, DMSO-d_6: 500 µL - 0.1% TFA).	S104
**Figure S91**	UV absorption spectrum of **12d** (MeOH). c = 1.05 x 10^{-4} (M)	S105
**Figure S92**	IR spectrum of **12d** (ATR).	S106
**Scheme S5**	Synthesis of **12e**.	S107
	Properties of **12e**.	S108
**Figure S93**	^{1}H NMR spectrum of **12e** (1.80 mg) (600 MHz, CD_3OD: 550 µL - 0.1% TFA).	S109
**Figure S94**	^{13}C NMR spectrum of **12e** (1.80 mg) (151 MHz, CD_3OD: 550 µL - 0.1% TFA).	S110
**Figure S95**	COSY spectrum of **12e** (1.80 mg) (600 MHz, CD_3OD: 550 µL - 0.1% TFA).	S111
**Figure S96**	TOCSY spectrum of **12e** (1.80 mg) (600 MHz, CD_3OD: 550 µL - 0.1% TFA).	S112
**Figure S97**	^{1}H-^{13}C HSQC spectrum of **12e** (1.80 mg) (600 MHz/151 MHz, CD_3OD: 550 µL - 0.1% TFA).	S113
**Figure S98**	^{1}H-^{13}C HMBC spectrum of **12e** (1.80 mg) (600 MHz/151 MHz, CD_3OD: 550 µL - 0.1% TFA).	S114
**Figure S99**	^{1}H NMR spectrum of **12e** (1.74 mg) (600 MHz, DMSO-d_6: 500 µL - 0.1% TFA).	S115
**Figure S100**	COSY spectrum of **12e** (1.74 mg) (600 MHz, DMSO-d_6: 500 µL - 0.1% TFA).	S116
**Figure S101**	NOESY spectrum of **12e** (1.74 mg) (600 MHz, DMSO-d_6: 500 µL - 0.1% TFA).	S117
**Figure S102**	UV absorption spectrum of **12e** (MeOH). c = 1.24 x 10^{-4} (M)	S118
**Figure S103**	IR spectrum of **12e** (ATR).	S119
**Scheme S6**	Synthesis of **12f**.	S120
	Properties of **12f**.	S121
**Figure S104**	^{1}H NMR spectrum of **12f** (0.53 mg) (600 MHz, DMSO-d_6: 180 µL - 0.1% TFA).	S122
**Figure S105**	COSY spectrum of **12f** (0.53 mg) (600 MHz, DMSO-d_6: 180 µL - 0.1% TFA).	S123
**Figure S106**	NOESY spectrum of **12f** (0.53 mg) (600 MHz, DMSO-d_6: 180 µL - 0.1% TFA).	S124
**Figure S107**	UV absorption spectrum of **12f** (MeOH). c = 9.65 x 10^{-5} (M)	S125

Figure S108 IR spectrum of **12f** (ATR). S126

Scheme S7 Synthesis of **12g**. S127

Properties of **12g**. S128

Figure S109 ^{1}H NMR spectrum of **12g** (1.61 mg) (600 MHz, CD_3OD: 550 μL - 0.1% TFA). S129

Figure S110 ^{13}C NMR spectrum of **12g** (1.61 mg) (151 MHz, CD_3OD: 550 μL - 0.1% TFA). S130

Figure S111 COSY spectrum of **12g** (1.61 mg) (600 MHz, CD_3OD: 550 μL - 0.1% TFA). S131

Figure S112 TOCSY spectrum of **12g** (1.61 mg) (600 MHz, CD_3OD: 550 μL - 0.1% TFA). S132

Figure S113 ^{1}H-^{13}C HSQC spectrum of **12g** (1.61 mg) (600 MHz/151 MHz, CD_3OD: 550 μL - 0.1% TFA). S133

Figure S114 ^{1}H-^{13}C HMBC spectrum of **12g** (1.61 mg) (600 MHz/151 MHz, CD_3OD: 550 μL - 0.1% TFA). S134

Figure S115 ^{1}H NMR spectrum of **12g** (1.55 mg) (600 MHz, DMSO-d_6: 500 μL - 0.1% TFA). S135

Figure S116 COSY spectrum of **12g** (1.55 mg) (600 MHz, DMSO-d_6: 500 μL - 0.1% TFA). S136

Figure S117 NOESY spectrum of **12g** (1.55 mg) (600 MHz, DMSO-d_6: 500 μL - 0.1% TFA). S137

Figure S118 UV absorption spectrum of **12g** (MeOH). c = 6.05 x 10^{-5} (M) S138

Figure S119 IR spectrum of **12g** (ATR). S139

Scheme S8 Synthesis of **12h**. S140

Properties of **12h**. S141

Figure S120 ^{1}H NMR spectrum of **12h** (3.43 mg) (600 MHz, CD_3OD: 500 μL - 0.4% TFA). S142

Figure S121 ^{13}C NMR spectrum of **12h** (3.43 mg) (151 MHz, CD_3OD: 500 μL - 0.4% TFA). S143

Figure S122 COSY spectrum of **12h** (3.43 mg) (600 MHz, CD_3OD: 500 μL - 0.4% TFA). S144

Figure S123 TOCSY spectrum of **12h** (3.43 mg) (600 MHz, CD_3OD: 500 μL - 0.4% TFA). S145

Figure S124 ^{1}H-^{13}C HSQC spectrum of **12h** (3.43 mg) (600 MHz, CD_3OD: 500 μL - 0.4% TFA). S146

Figure S125 ^{1}H-^{13}C HMBC spectrum of **12h** (3.43 mg) (600 MHz, CD_3OD: 500 μL - 0.4% TFA). S147

Figure S126 NOESY-1D spectrum of **12h** (3.43 mg); irradiation at 6.26 ppm (600 MHz, CD_3OD: 500 μL - 0.4% TFA). S148

Figure S127 NOESY-1D spectrum of **12h** (3.43 mg); irradiation at 2.67 ppm (600 MHz, CD_3OD: 500 μL - 0.4% TFA). S149

Figure S128 UV absorption spectrum of **12h** (MeOH). c = 3.12 x 10^{-5} (M) S150

**Figure S129**	IR spectrum of **12h** (ATR).	S151
**Scheme S9**	Synthesis of **12i**.	S152
	Properties of **12i**.	S153
**Figure S130**	^{1}H NMR spectrum of **12i** (1.77 mg) (600 MHz, CD_3OD: 500 μL - 0.1% TFA).	S154
**Figure S131**	^{13}C NMR spectrum of **12i** (1.77 mg) (151 MHz, CD_3OD: 500 μL - 0.1% TFA).	S155
**Figure S132**	COSY spectrum of **12i** (1.77 mg) (600 MHz, CD_3OD: 500 μL - 0.1% TFA).	S156
**Figure S133**	TOCSY spectrum of **12i** (1.77 mg) (600 MHz, CD_3OD: 500 μL - 0.1% TFA).	S157
**Figure S134**	^{1}H-^{13}C HSQC spectrum of **12i** (1.77 mg) (600 MHz/151 MHz, CD_3OD: 500 μL - 0.1% TFA).	S158
**Figure S135**	^{1}H-^{13}C HMBC spectrum of **12i** (1.77 mg) (600 MHz/151 MHz, CD_3OD: 500 μL - 0.1% TFA).	S159
**Figure S136**	^{1}H NMR spectrum of **12i** (1.70 mg) (600 MHz, DMSO-d_6: 500 μL - 0.1% TFA).	S160
**Figure S137**	COSY spectrum of **12i** (1.70 mg) (600 MHz, DMSO-d_6: 500 μL - 0.1% TFA).	S161
**Figure S138**	NOESY spectrum of **12i** (1.70 mg) (600 MHz, DMSO-d_6: 500 μL - 0.1% TFA).	S162
**Figure S139**	UV absorption spectrum of **12i** (MeOH). c = 1.13×10^{-4} (M)	S163
**Figure S140**	IR spectrum of **12i** (ATR).	S164
**Scheme S10**	Possible mechanism for the formation of (±)-tauroacidin A (**2**) from oroidin (**3**) with $NaClO_2$ and taurine.	S165
**Table S4**	Amino acids that could not be introduced into oroidin (**3**).	S166
**Scheme S11**	Reaction of oroidin (**3**) with 2-hydroxypyridine or 4-hydroxypyridine using sodium chlorite.	S167
**Scheme S12**	Isomerization of hydroxypyridine isomers.	S167
**Scheme S13**	Product and by-products during the synthesis of agelamadin F (**1**).	S168
**Scheme S14**	Product and putative by-products during the synthesis of tauroacidin A (**2**) based on MS spectra.	S168
**Scheme S15**	Product and putative by-products during the synthesis of compounds **12a–i** based on MS spectra.	S168

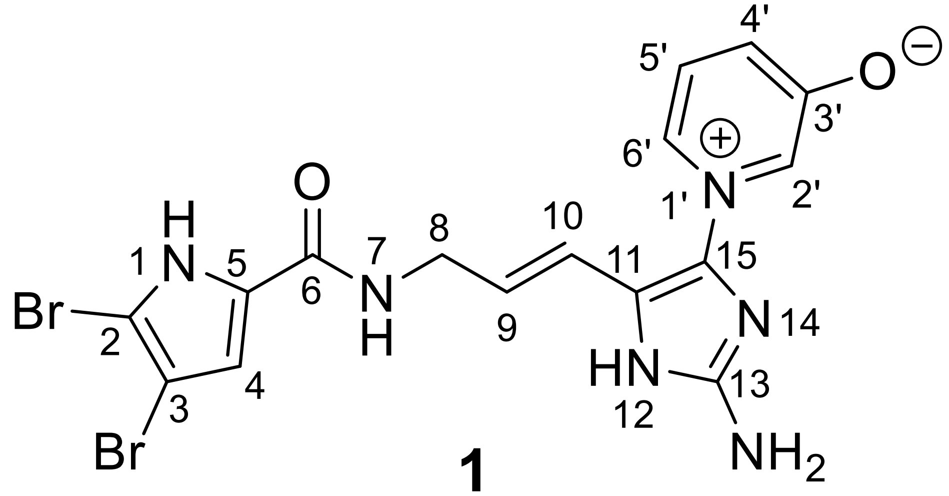

1

Agelamadin F (1):

R_f = 0.54 ($CHCl_3$/MeOH/28% NH_3 aq. = 70:30:2, v/v/v; UV).

UV/vis λ_{max} (MeOH) nm (log ε): 413.0 (3.40), 277.0 (4.30), 203.0 (4.50).

^{1}H and ^{13}C NMR: See Table S1–2

IR v_{max}: 3121 (br), 2934 (w), 1681 (s), 1640 (s), 1564 (m), 1525 (w), 1488 (w), 1425 (w), 1324 (w), 1203 (s), 1136 (m).

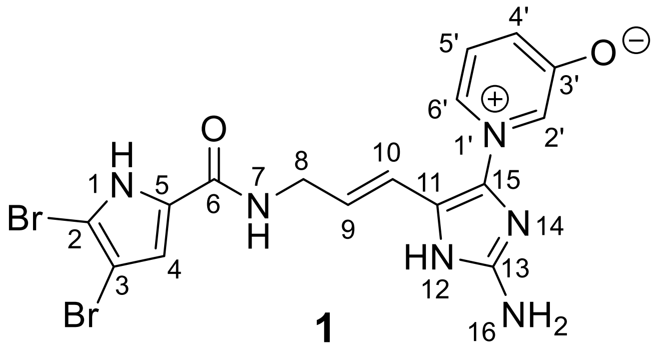

1

Table S1. ^{1}H (600 MHz), ^{13}C (151 MHz), and ^{15}N (60.8 MHz) NMR data for **1**, and reported data for **1** in DMSO-d_6 (δ in ppm).

	Synthetic agelamadin F (**1**)				Natural **1** (reported)[1]			Δδ (Synthetic **1** - Natural **1**)	
Position	δ_N[a]	δ_C, type	δ_H (J in Hz)	^{13}C HMBC	δ_C, type	δ_H (J in Hz)	^{13}C HMBC	δ_C	δ_H
1			12.70, br			12.70, br s	2, 3, 4, 5		0.00
2		105.0, C	-		104.7, C	-		0.3	
3		97.7, C	-		97.9, C	-		-0.2	
4		112.7, CH	6.90, s[b]	2, 5, 6	112.7, CH	6.93, d (1.7)	2, 3, 5, 6	0.0	-0.03
5		128.4, C	-		128.0, C	-		0.4	
6		159.0, C	-		158.7, C	-		0.3	
7	107.2		8.40, t (5.7)	6		8.39, t (5.8)			0.01
8		40.5, CH_2	3.93, t (5.8)	6, 9, 10	40.5, CH_2	3.96, t (5.8)	6	0.0	-0.03
9		124.2, CH	5.95, dt (15.7, 6.3)	8, 11	126.4, CH	6.07, dt (15.8, 5.8)	11	-2.2	-0.12
10		115.8, CH	6.26, d (15.8)	8, 9, 11, 15	115.4, CH	6.41, d (15.8)	15	0.4	-0.15
11		114.4, C	-		115.8, C	-		-1.4	
12	135.3		11.43, s		-	11.75, br s			-0.32
13		149.5, C	-		150.0, C	-		-0.5	
14			Nd		-	Nd			
15		135.6, C	-		134.2, C	-		1.4	
16	99.8		7.62, s<br>7.19, s			Nd			
1'			-		-	-			
2'		132.5, CH	7.44, s	15	130.6, CH	8.51, br s	15, 4', 6'	1.9	-1.07
3'		168.3, C	-		157.1, C	-		11.2	
4'		133.7, CH	7.10, dd (9.0, 2.1)	2', 6'	131.5, CH	7.98, br d (8.8)		2.2	-0.88
5'		126.8, CH	7.39, dd (8.4, 5.7)	6'	128.6, CH	7.96, dd (8.8, 5.6)	3'	-1.8	-0.57
6'		121.8, CH	7.50, d (5.5)	4', 5'	134.1, CH	8.55, br d (5.6)	15	-12.3	-1.05
(3'-OH)			5.90, s[c]		-	6.10, br s			-0.20

[a]Determined by ^{1}H-^{15}N HSQC.

[b]Two signals were observed for C4–H: a major peak at 6.90 ppm and a minor peak at 6.92 ppm.

[c]The signal at 5.90 ppm was assigned to 3'-OH according to the isolation paper.[1] The integral value of this signal was 2.

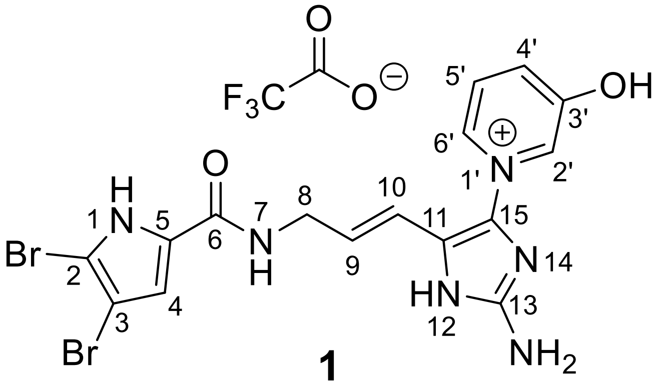

1

Table S2. ^{1}H (600 MHz), ^{13}C (151 MHz), and ^{15}N (60.8 MHz) NMR data for **1** (DMSO-d_6: 500 μL; TFA: 2.0 μL), and reported data for **1** (DMSO-d_6) (δ in ppm).

	Synthetic agelamadin F (**1**) (TFA added)				Natural **1** (reported)[1]			Δδ (Synthetic **1** - Natural **1**)	
Position	δ_N	δ_C, type	δ_H (J in Hz)	^{13}C HMBC	δ_C, type	δ_H (J in Hz)	^{13}C HMBC	δ_C	δ_H
1	165.9		12.71, d (2.4)	3, 4, 5		12.70, br s	2, 3, 4, 5		0.01
2		104.8, C	-		104.7, C	-		0.1	
3		97.9, C	-		97.9, C	-		0.0	
4		112.7, CH	6.94, d (3.0)	2, 5	112.7, CH	6.93, d (1.7)	2, 3, 5, 6	0.0	0.01
5		128.0, C	-		128.0, C	-		0.0	
6		158.8, C	-		158.7, C	-		0.0	
7	106.6		8.40, t (5.4)	6, 8		8.39, t (5.8)			0.01
8		40.5, CH_2	3.97, t (5.7)	6, 9, 10	40.5, CH_2	3.96, t (5.8)	6	0.0	0.01
9		126.5, CH	6.09, dt (15.6, 6.0)	8, 10, 11	126.4, CH	6.07, dt (15.8, 5.8)	11	0.1	0.02
10		115.4, CH	6.41, d (15.6)	8, 9, 11, 15	115.4, CH	6.41, d (15.8)	15	0.0	0.00
11		115.9, C	-		115.8, C	-		0.1	
12	136.8		11.75, br s		-	11.75, br s			0.00
13		150.0, C	-		150.0, C	-		0.0	
14			Nd		-	Nd			
15		134.2, C	-		134.2, C	-		0.0	
1'	209.0		-		-	-			
2'		130.6, CH	8.52, s	15, 6'	130.6, CH	8.51, br s	15, 4', 6'	0.0	0.01
3'		157.1, C	-		157.1, C	-		0.0	
4'		131.6, CH	7.99, dd (9.6)	2', 6'	131.5, CH	7.98, br d (8.8)		0.1	0.01
5'		128.6, CH	7.98, dd (8.0)	3', 6'	128.6, CH	7.96, dd (8.8, 5.6)	3'	0.0	0.02
6'		134.1, CH	8.56, d (6.0)	2', 4'	134.1, CH	8.55, br d (5.6)	15	0.0	0.01
(3'-OH)			6.10, br s[a]		-	6.10, br s			0.00

[a]The signal at 6.10 ppm (assigned to 3'-OH) observed after addition of TFA (0.75 μL, 1.1 equiv.) was completely absent after further addition of TFA (1.0 μL, 1.5 equiv.) (see Figure S16).

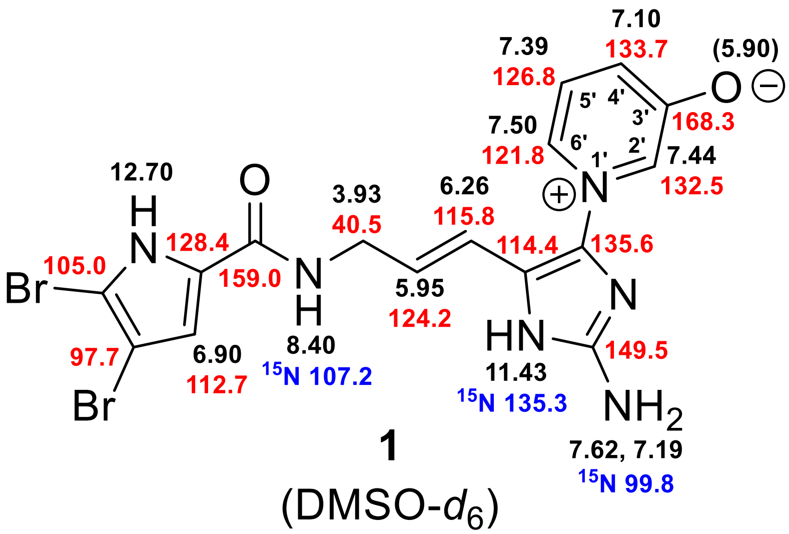

1

(DMSO-d_6)

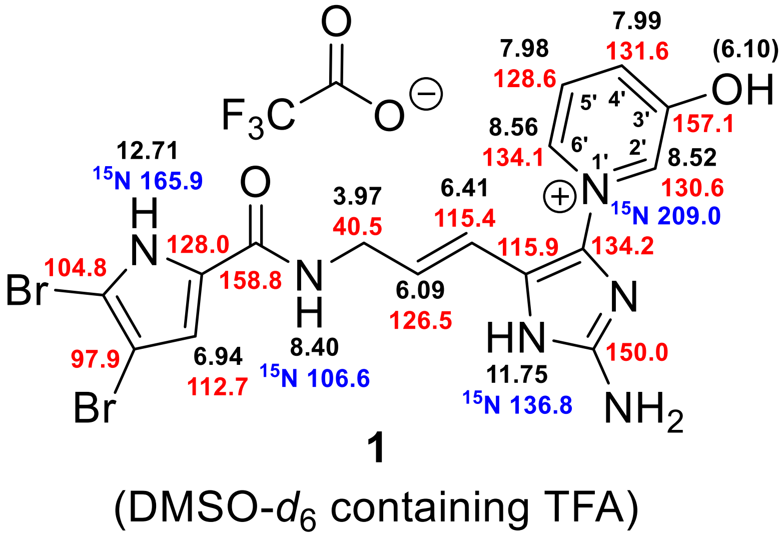

1

(DMSO-d_6 containing TFA)

Comparison of ^{1}H and ^{13}C NMR chemical shifts of 3-hydroxypyridine moiety.

	**1** (DMSO-d_6)		**1** (DMSO-d_6 containing TFA)
H2':	7.44 ppm	→	8.52 ppm (Δ +1.08 ppm)
(3'-OH):	5.90 ppm	→	6.10 ppm (Δ +0.20 ppm)
H4':	7.10 ppm	→	7.99 ppm (Δ +0.89 ppm)
H5':	7.39 ppm	→	7.98 ppm (Δ +0.59 ppm)
H6':	7.50 ppm	→	8.56 ppm (Δ +1.06 ppm)
C2':	132.5 ppm	→	130.6 ppm (Δ -1.9 ppm)
C3':	168.3 ppm	→	157.1 ppm (Δ -11.2 ppm)
C4':	133.7 ppm	→	131.6 ppm (Δ -2.1 ppm)
C5':	126.8 ppm	→	128.6 ppm (Δ+1.8 ppm)
C6':	121.8 ppm	→	134.1 ppm (Δ+12.3 ppm)

Figure S1. Comparison of ^{1}H and ^{13}C NMR chemical shift values of agelamadin F (**1**) before and after the addition of TFA. Chemical shifts of ^{1}H NMR (black), ^{13}C NMR (red), ^{15}N NMR (blue) are shown.

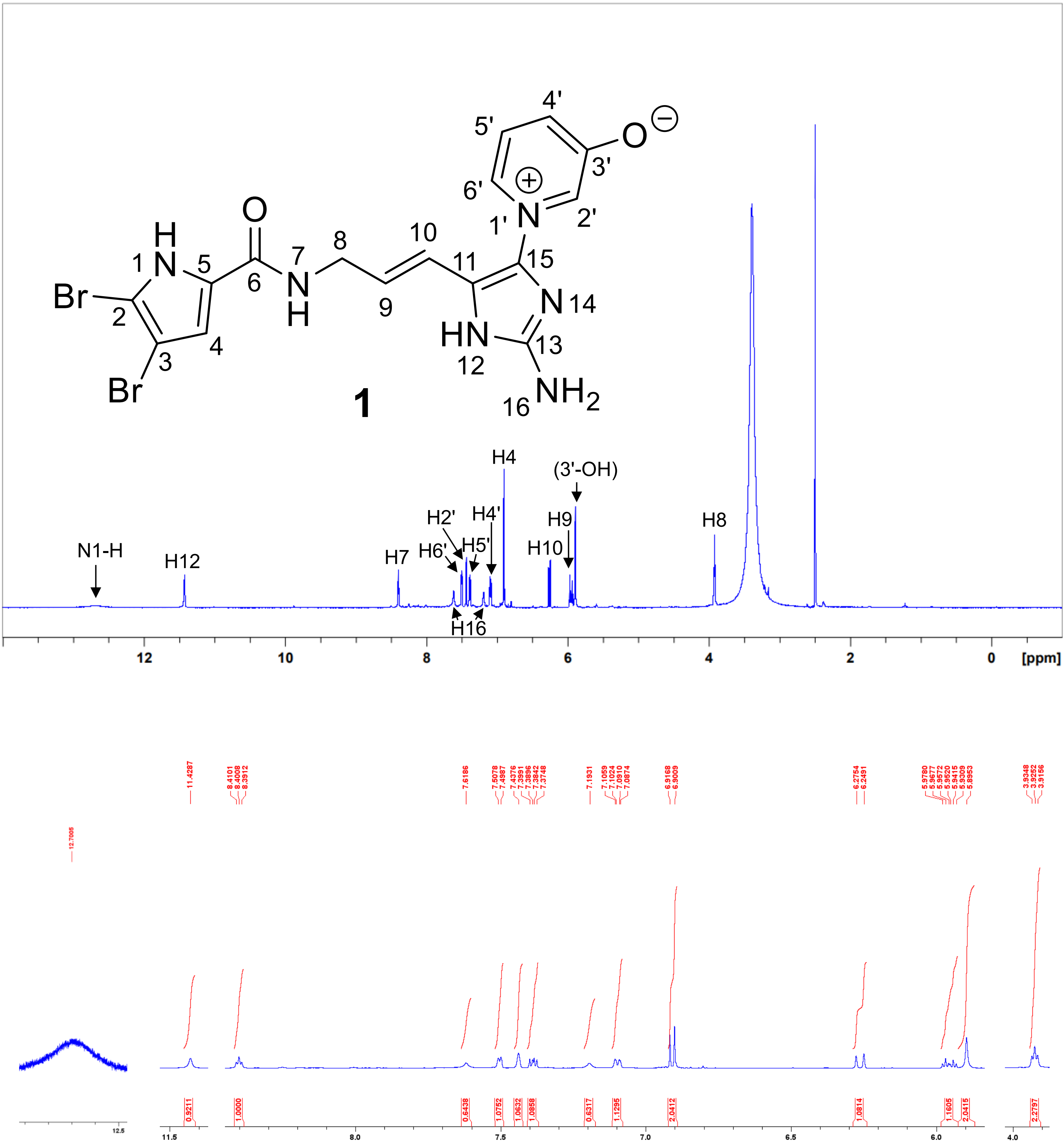

Figure S2. ^{1}H NMR spectrum of **1** (4.91 mg) (600 MHz, DMSO-d_6: 550 µL). The 16N-H_2 signals (7.62 and 7.19 ppm) appeared only after the sample had been dried in a vacuum desiccator for 42 days, whereas these signals were not observed when the ^{1}H NMR spectrum was recorded immediately after the synthesis of agelamadin F (**1**).

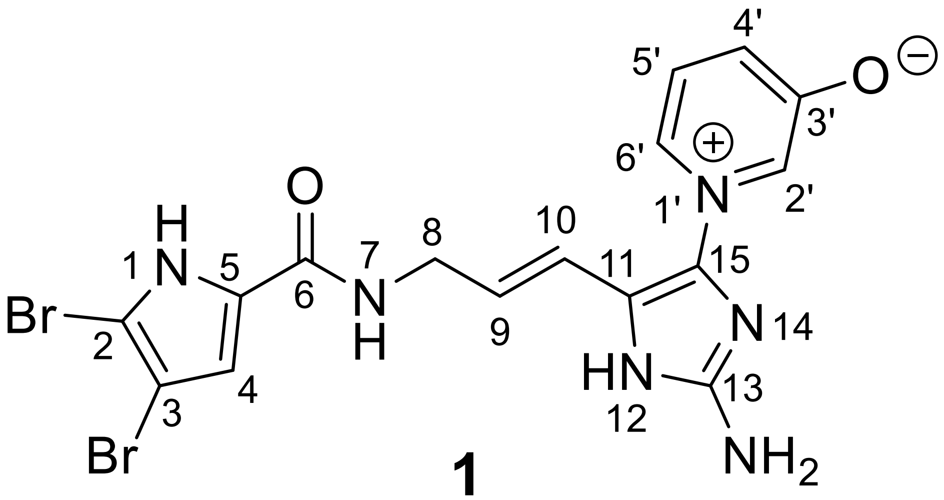

1

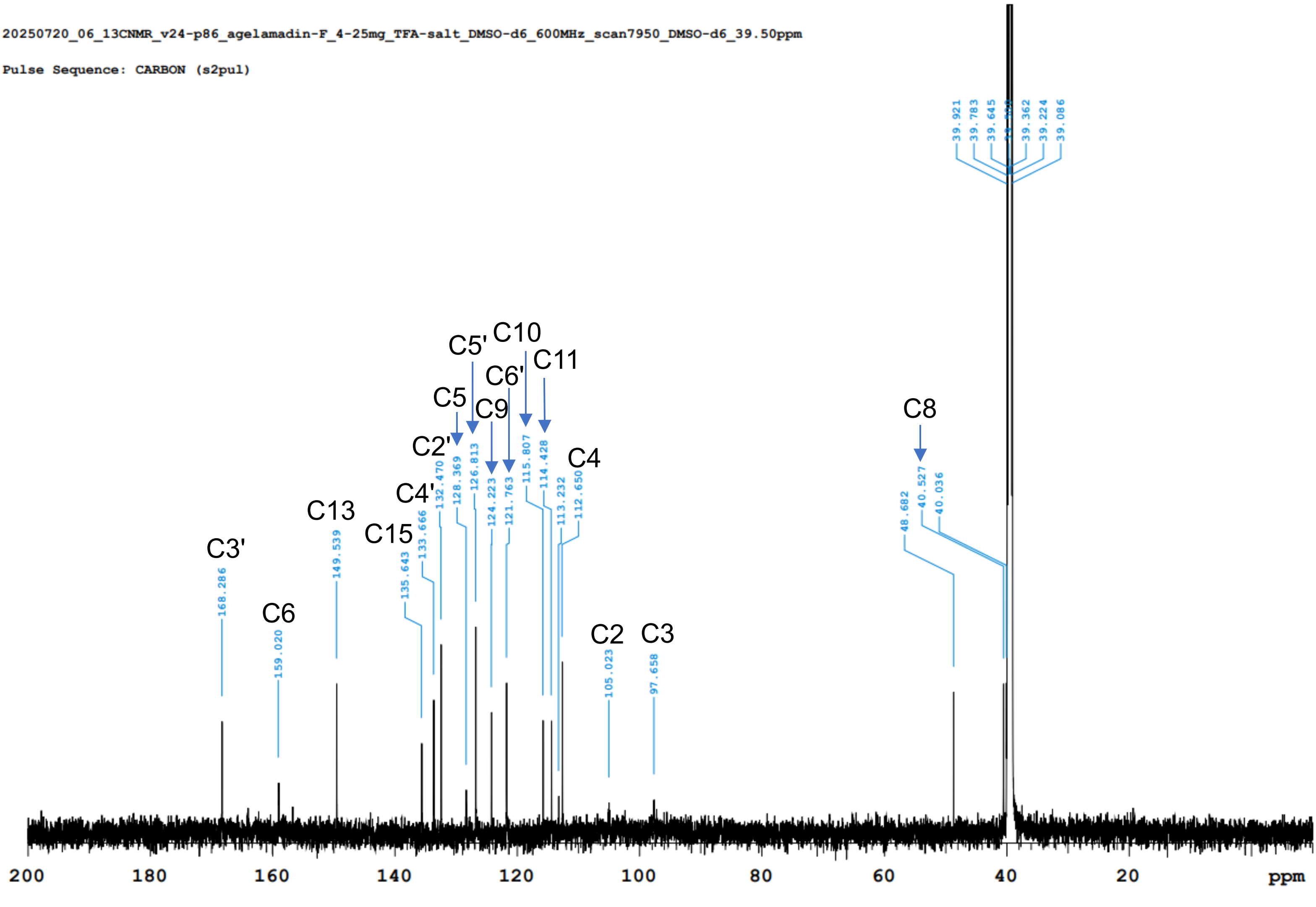

Figure S3. ^{13}C NMR spectrum of **1** (4.25 mg) (151 MHz, DMSO-d_6: 500 μL).

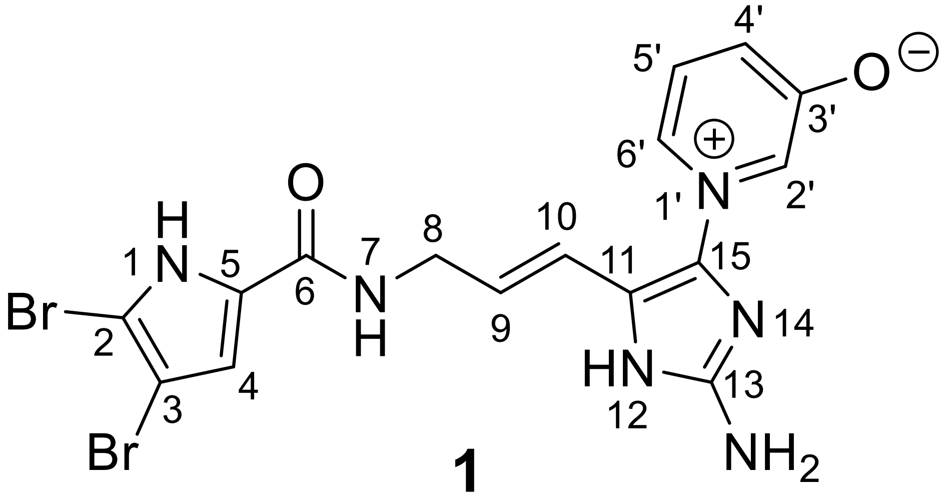

1

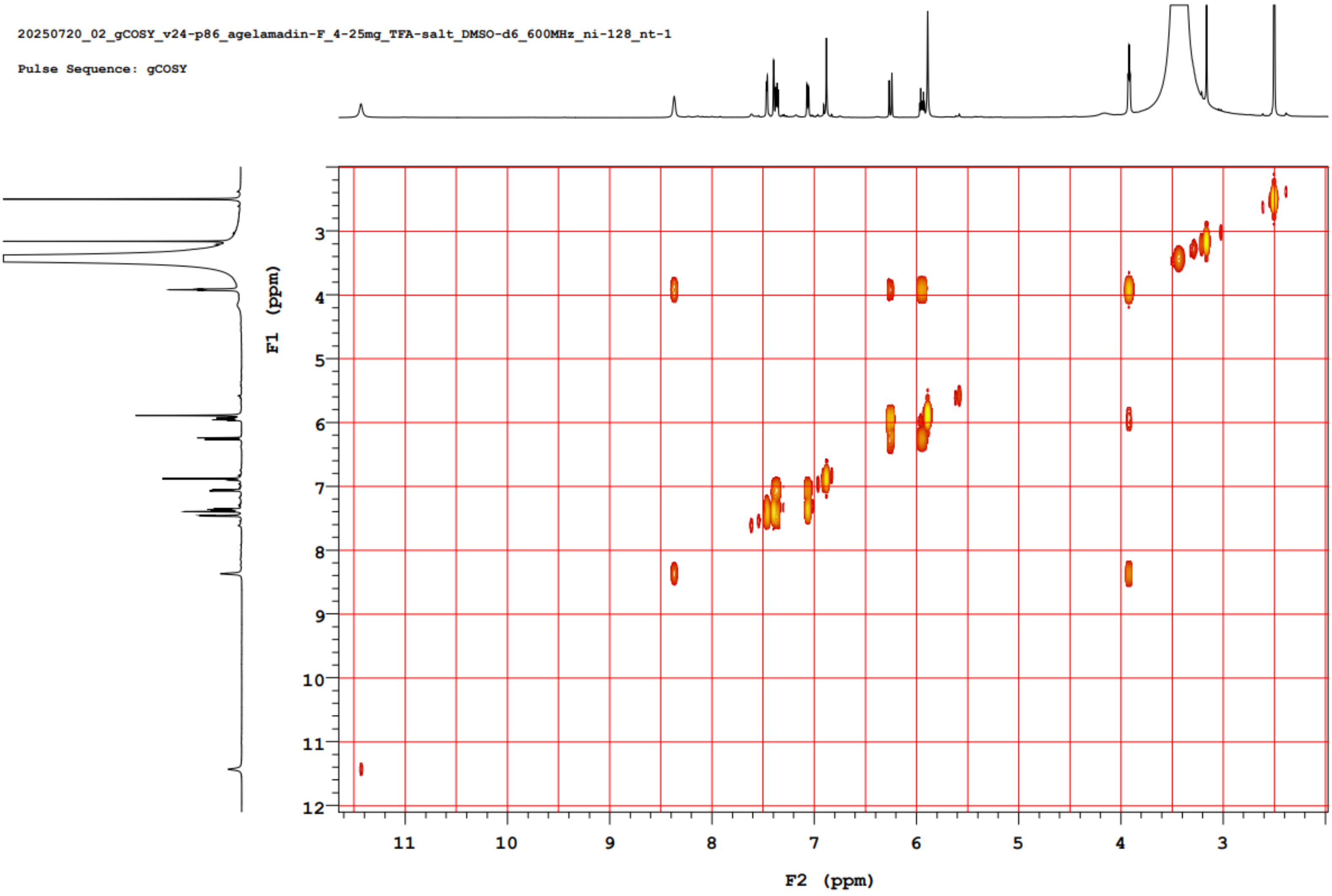

Figure S4. COSY spectrum of **1** (4.25 mg) (600 MHz, DMSO-d_6: 500 µL).

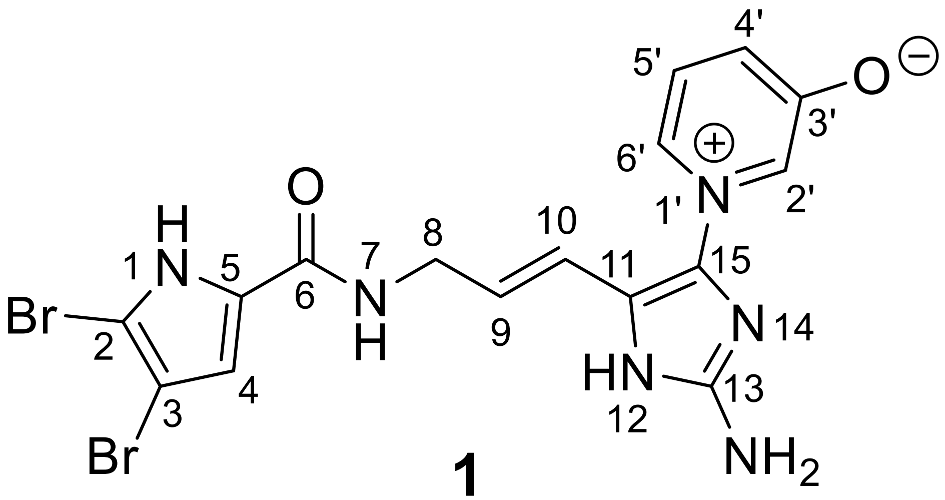

1

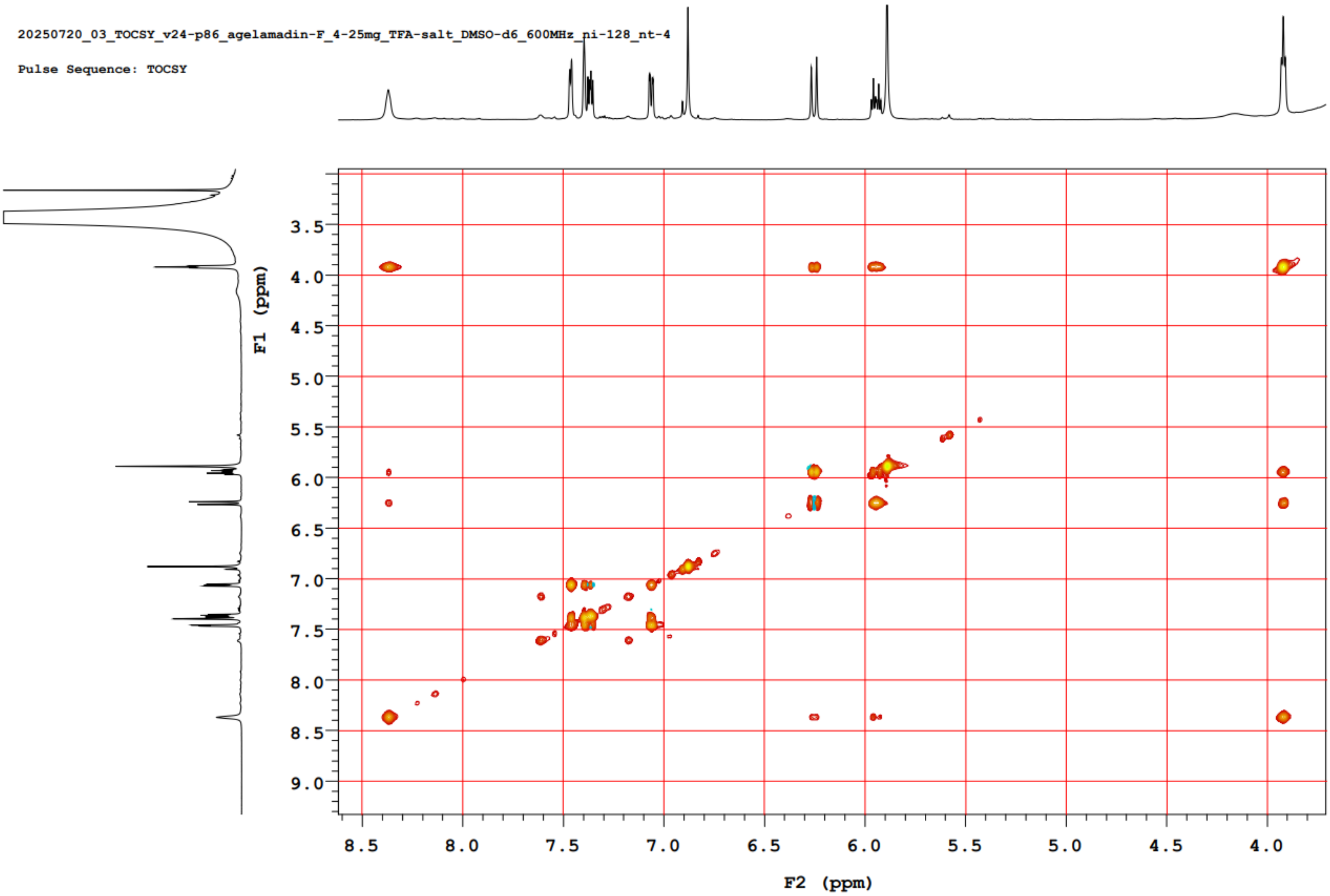

Figure S5. TOCSY spectrum of **1** (4.25 mg) (600 MHz, DMSO-d_6: 500 μL).

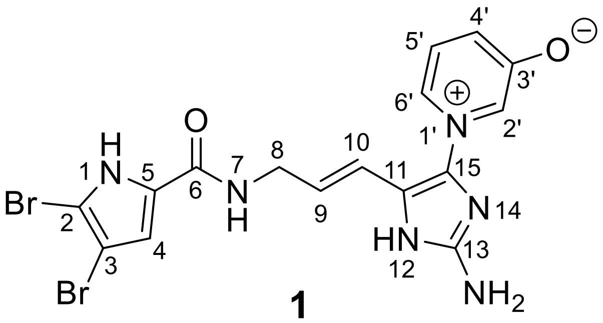

1

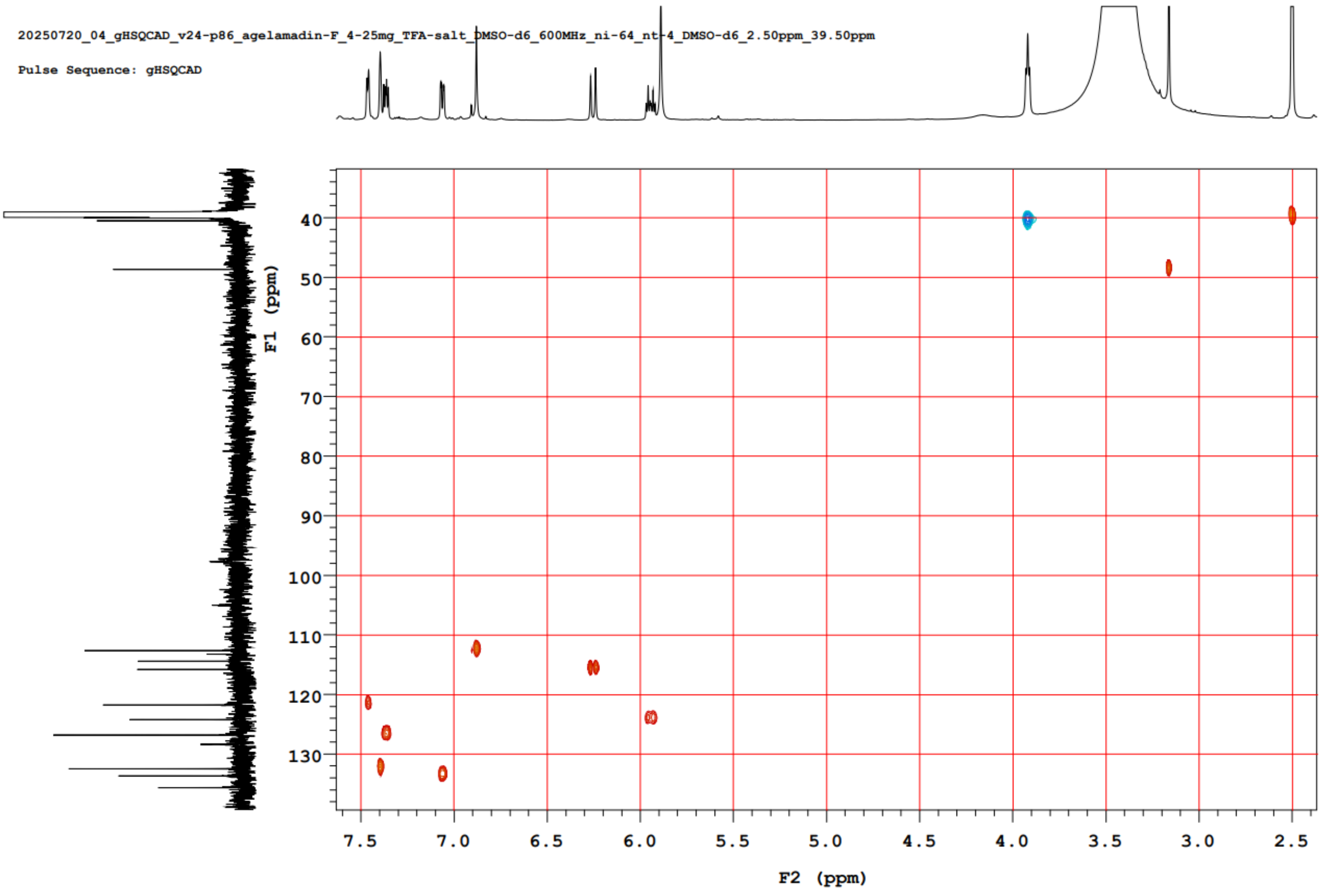

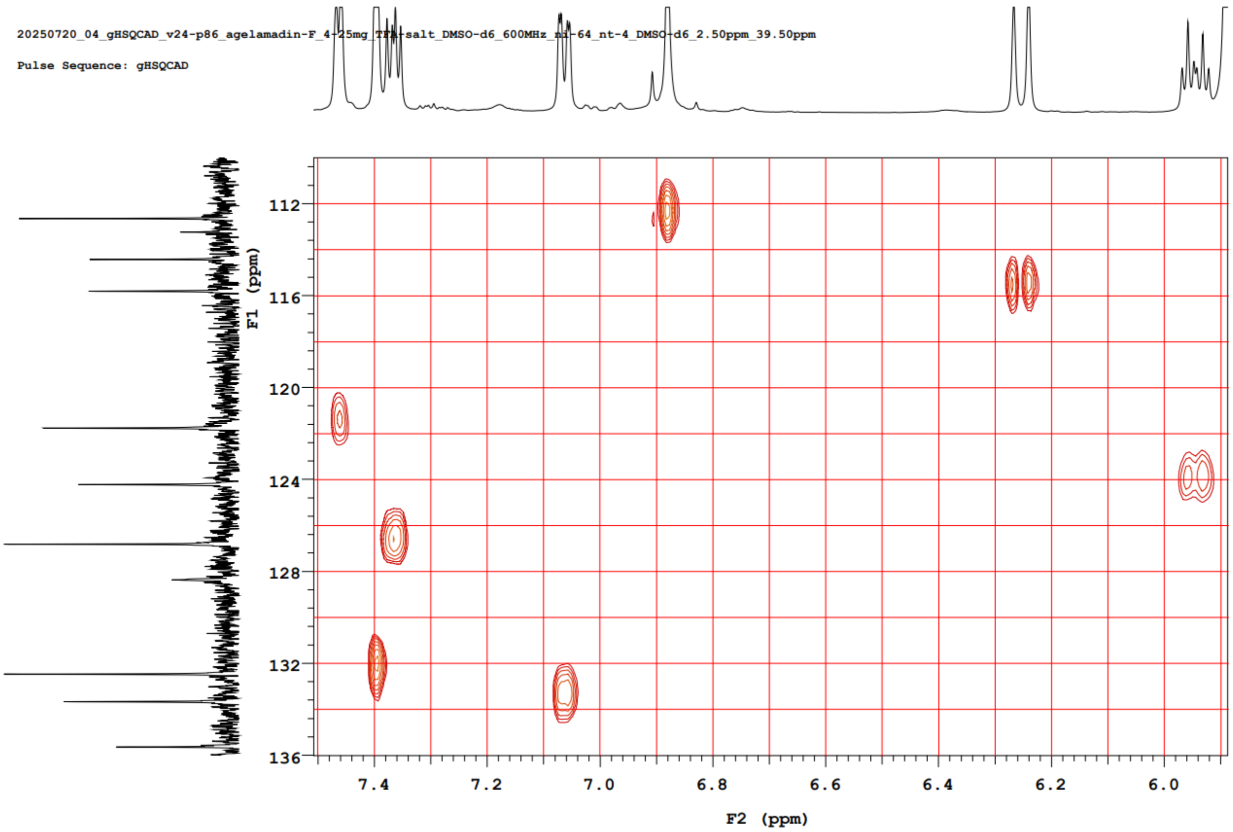

Figure S6. ^{1}H-^{13}C HSQC spectrum of **1** (4.25 mg) (600 MHz / 151 MHz, DMSO-d_6: 500 μL).

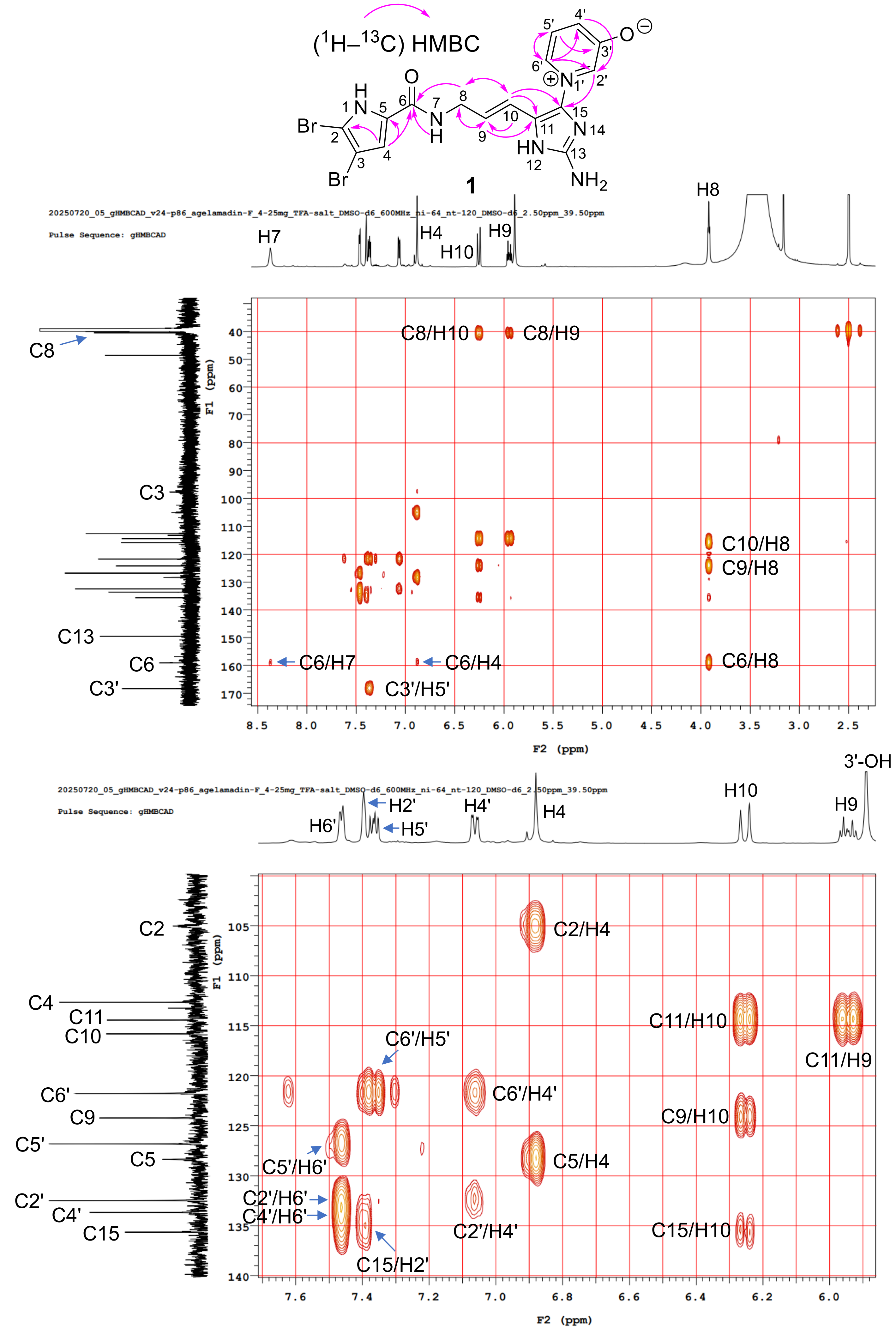

Figure S7. ^{1}H-^{13}C HMBC spectrum of **1** (4.25 mg) (600 MHz / 151 MHz, DMSO-d_6: 500 µL).

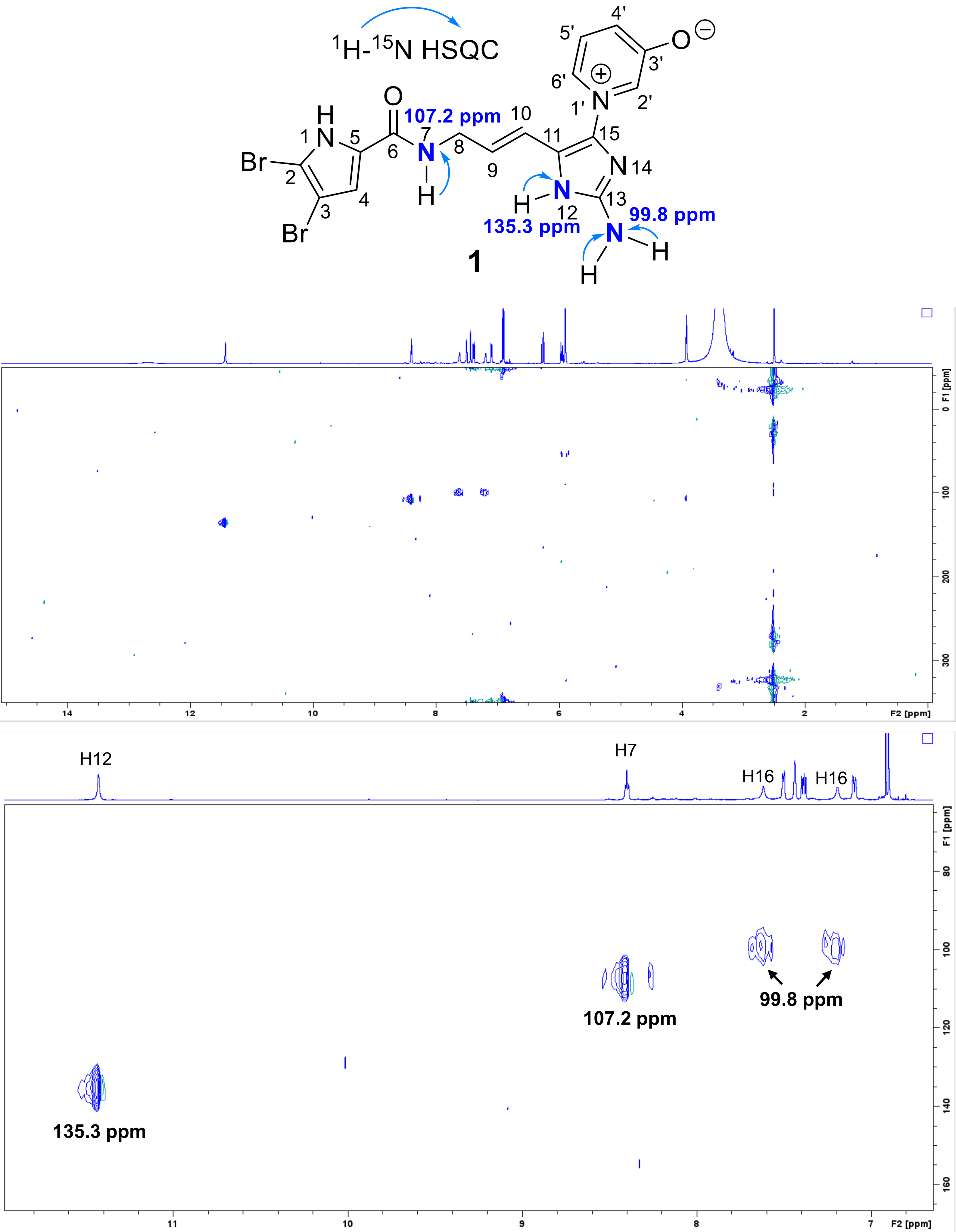

Figure S8. ^{1}H-^{15}N HSQC spectrum of **1** (4.91 mg) (600 MHz / 60.8 MHz, DMSO-d_6: 550 μL).

20250621_01_1HNMR_v23-p202_Agelamadin-F-TFA_4-91mg_DMSO-d6-500uL_TFA-2-0uL_600MHz_scan60_DMSO-d6_2.50ppm

Pulse Sequence: PROTON (s2pul)

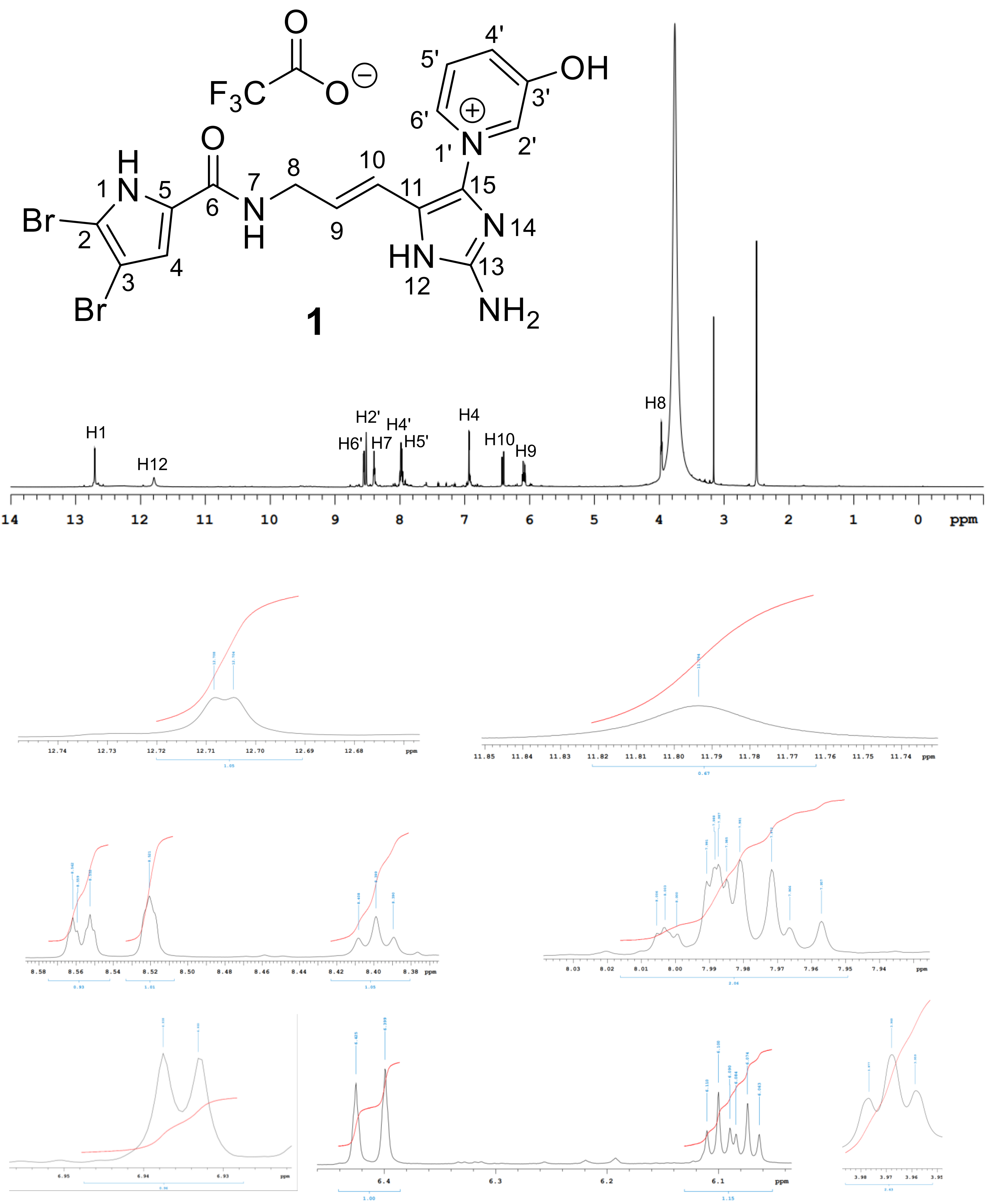

Figure S9. ^{1}H NMR spectrum of **1** (4.91 mg) (600 MHz, DMSO-d_6: 500 μL; TFA: 2.0 μL).

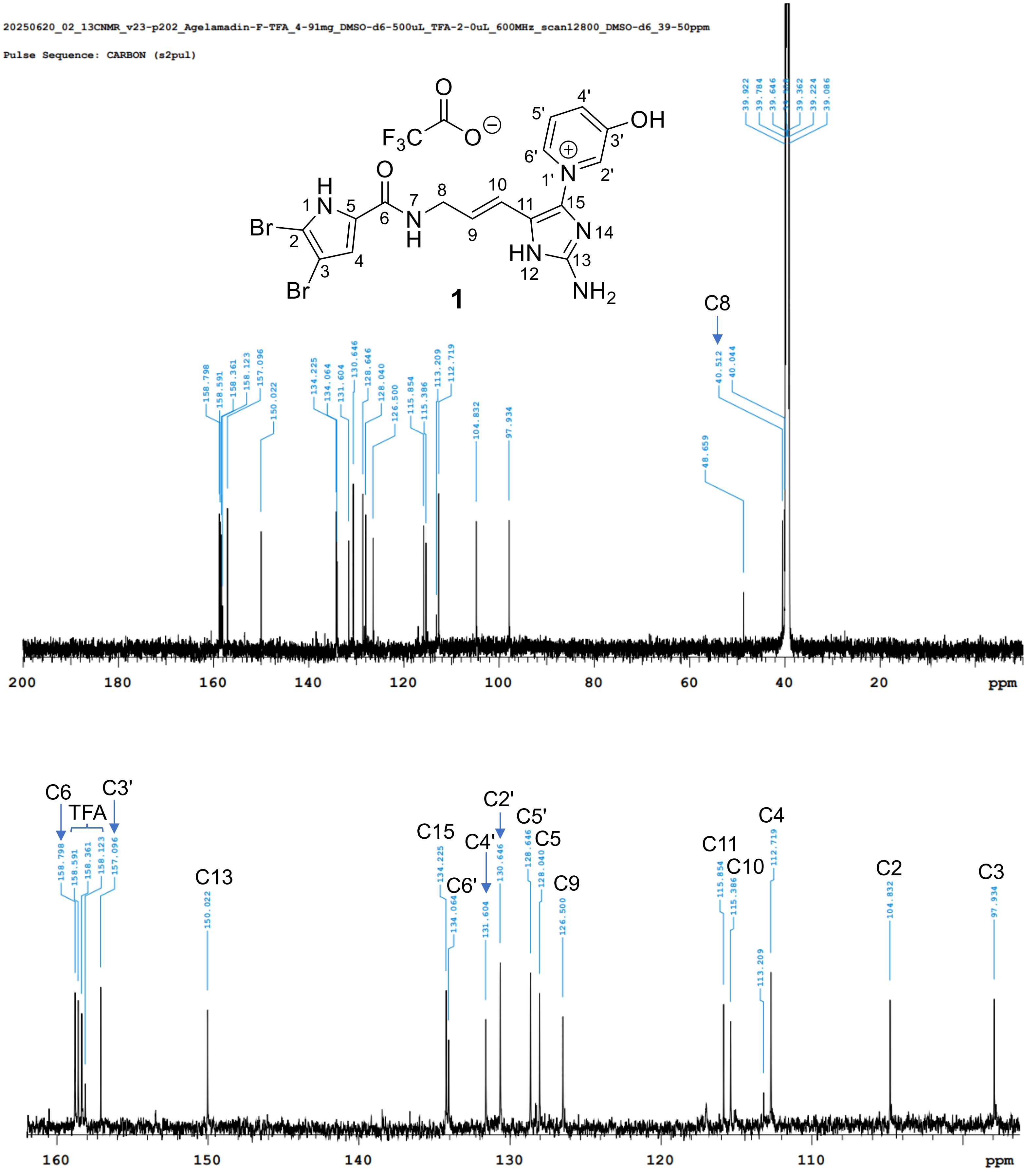

Figure S10. ^{13}C NMR spectrum of **1** (4.91 mg) (151 MHz, DMSO-d_6: 500 µL; TFA: 2.0 µL).

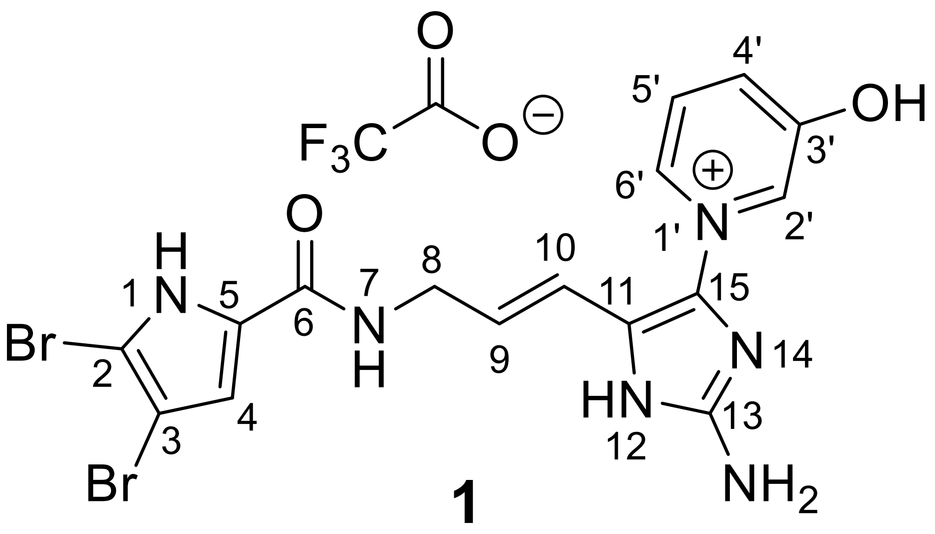

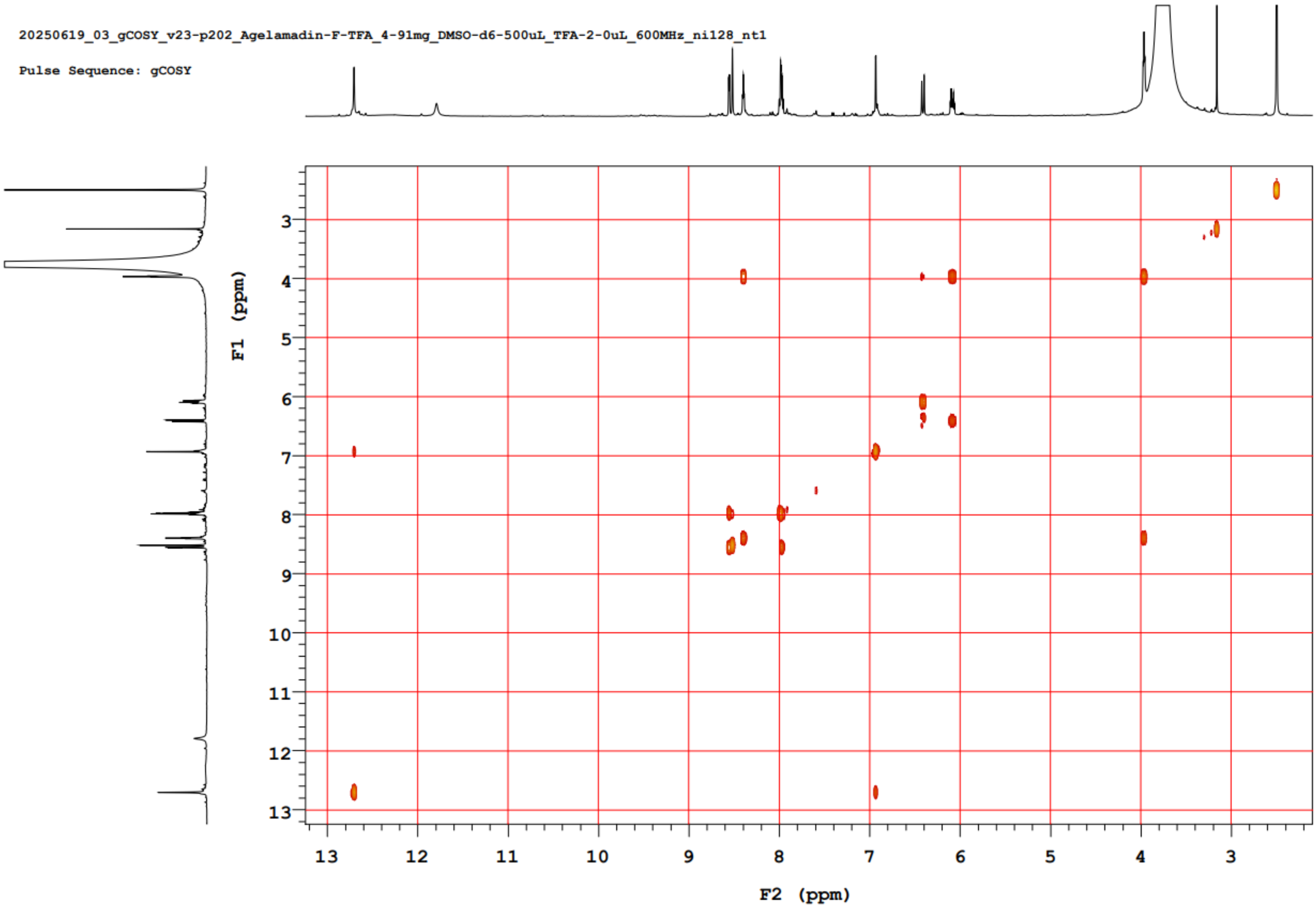

Figure S11. COSY spectrum of **1** (4.91 mg) (600 MHz, DMSO-d_6: 500 µL; TFA: 2.0 µL).

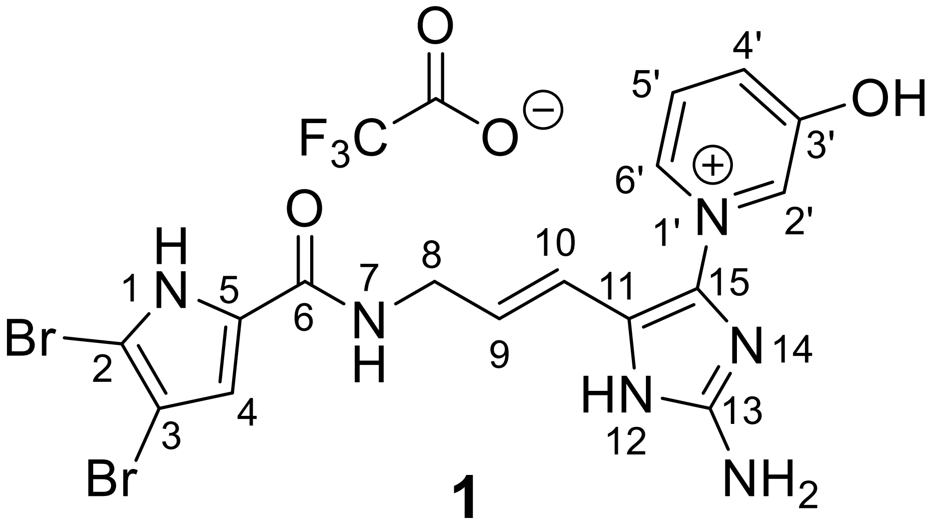

1

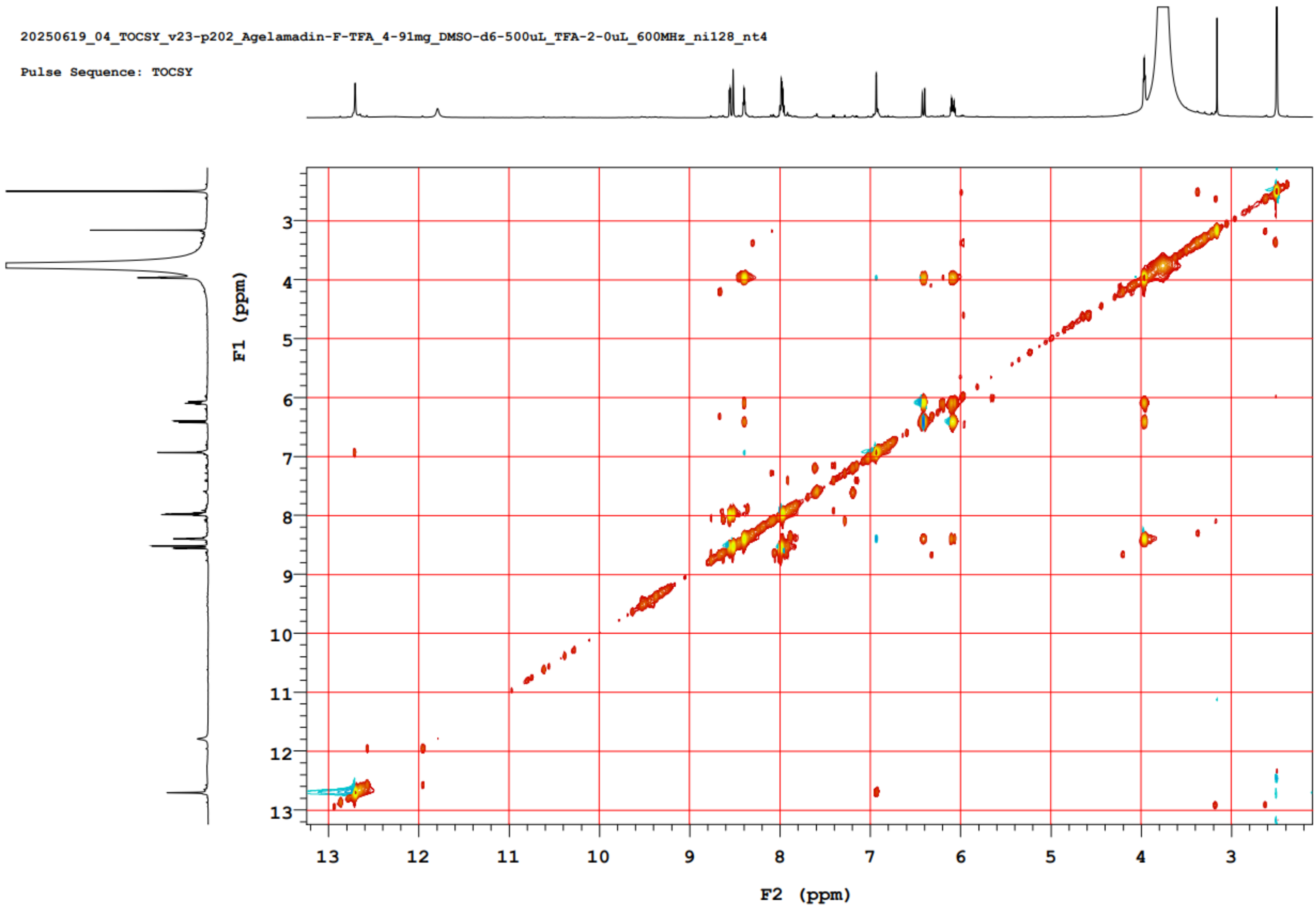

Figure S12. TOCSY spectrum of **1** (4.91 mg) (600 MHz, DMSO-d_6: 500 μL; TFA: 2.0 μL).

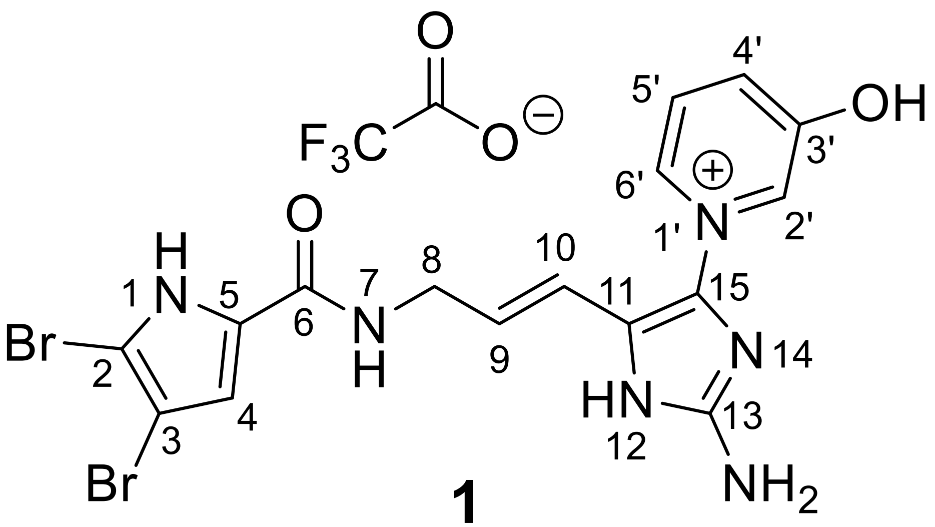

1

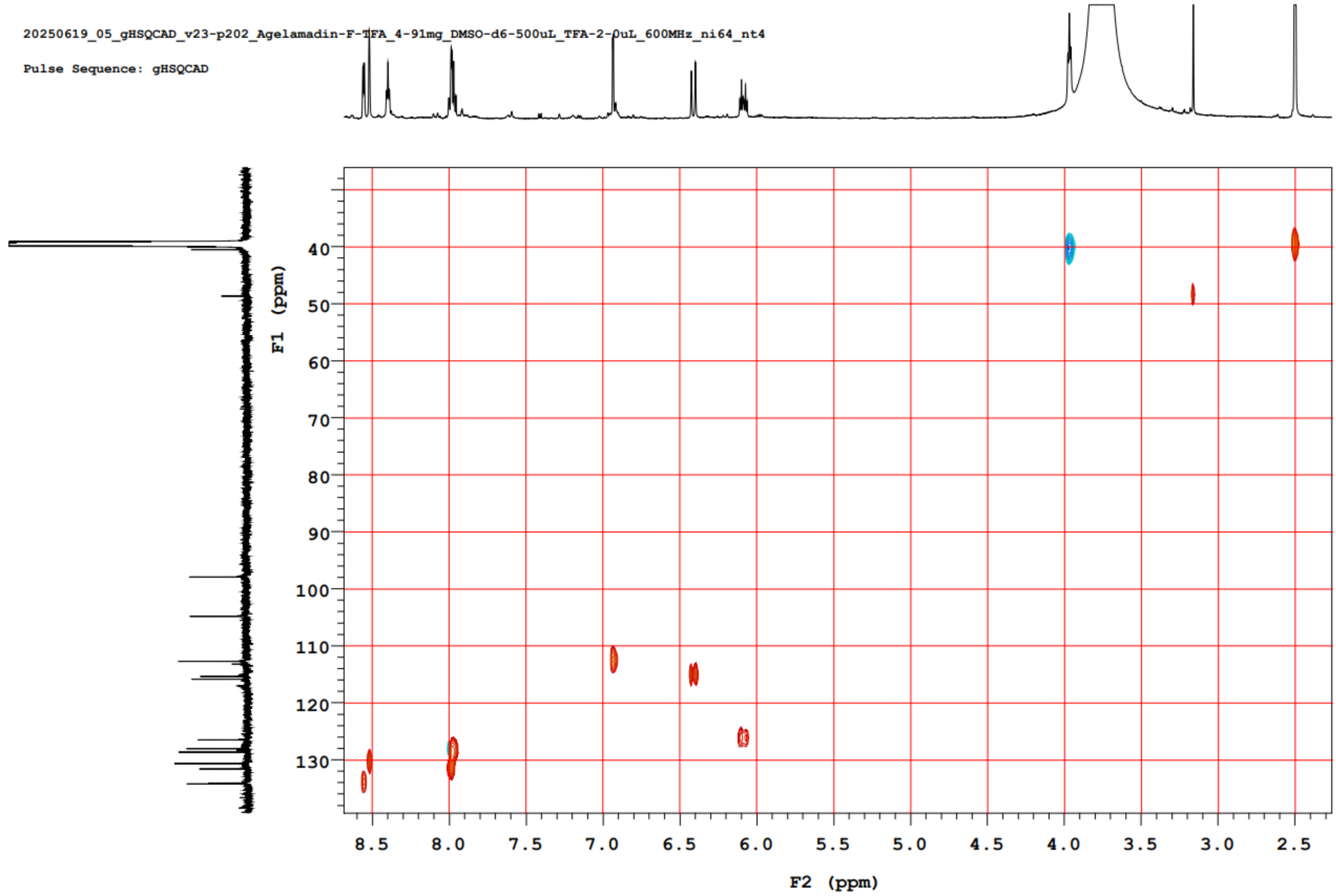

Figure S13. ^{1}H-^{13}C HSQC spectrum of **1** (4.91 mg) (600 MHz / 151 MHz, DMSO-d_6: 500 μL; TFA: 2.0 μL).

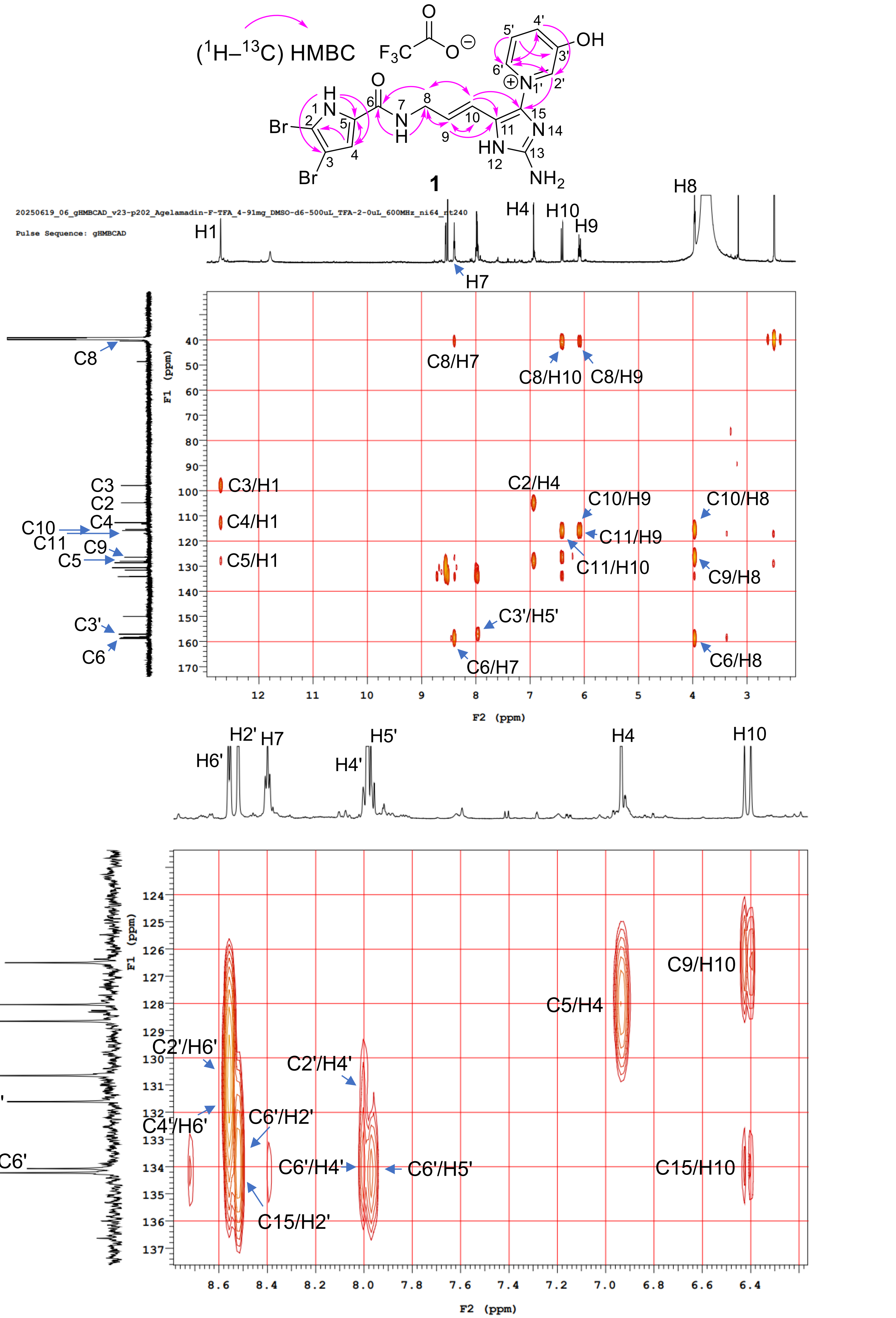

Figure S14. ^{1}H-^{13}C HMBC spectrum of **1** (4.91 mg) (600 MHz / 151 MHz, DMSO-d_6: 500 μL; TFA: 2.0 μL).

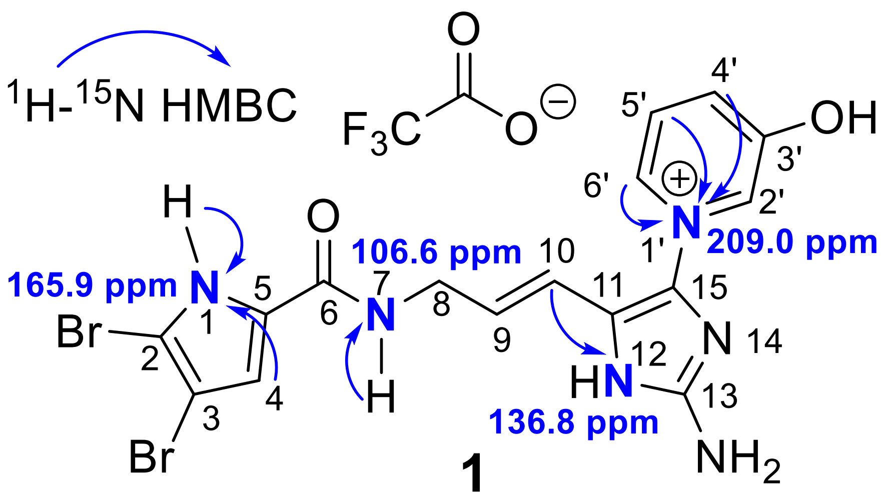

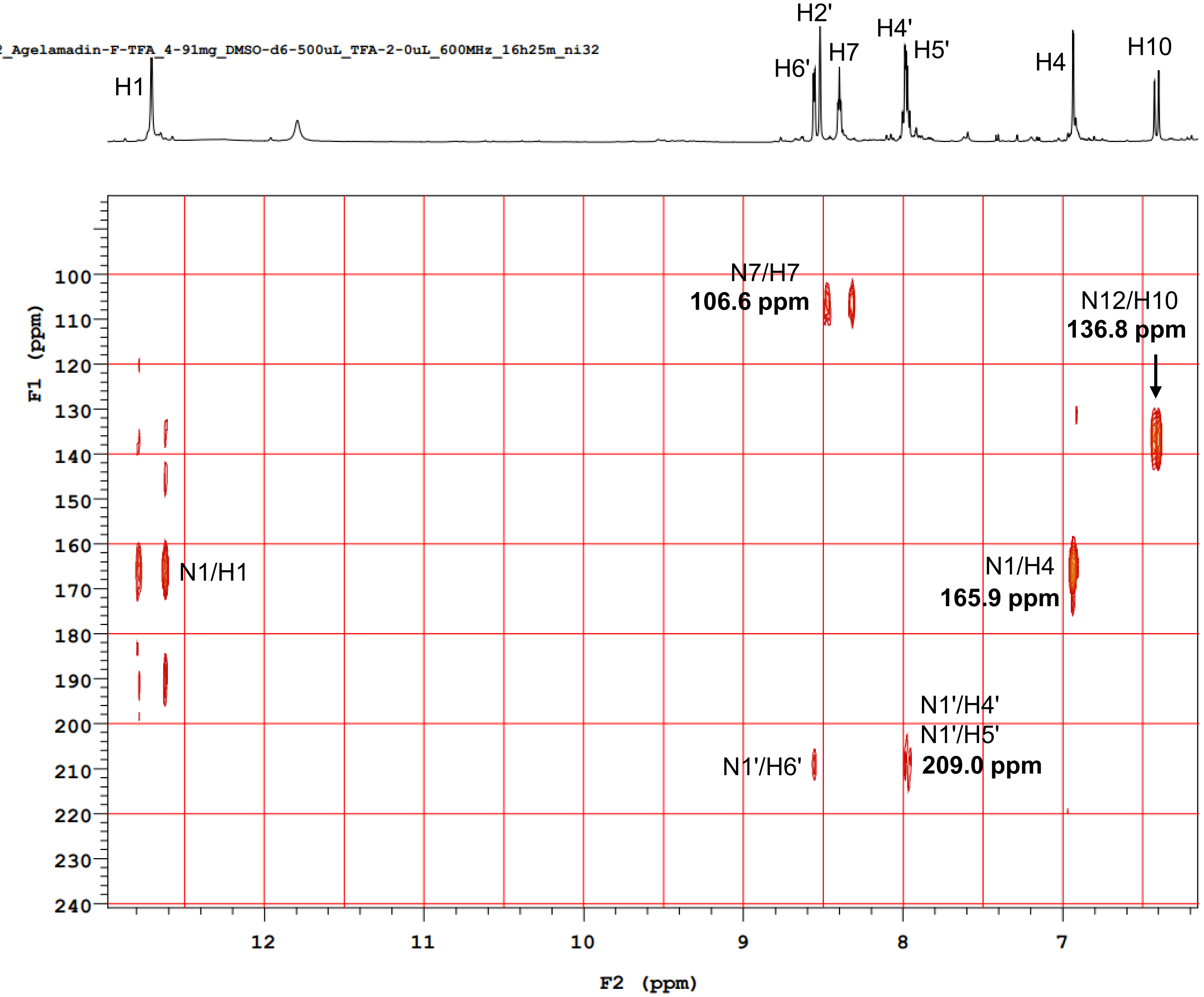

Figure S15. ^{1}H-^{15}N HMBC spectrum of **1** (4.91 mg) (600 MHz / 60.8 MHz, DMSO-d_6: 500 µL; TFA: 2.0 µL).

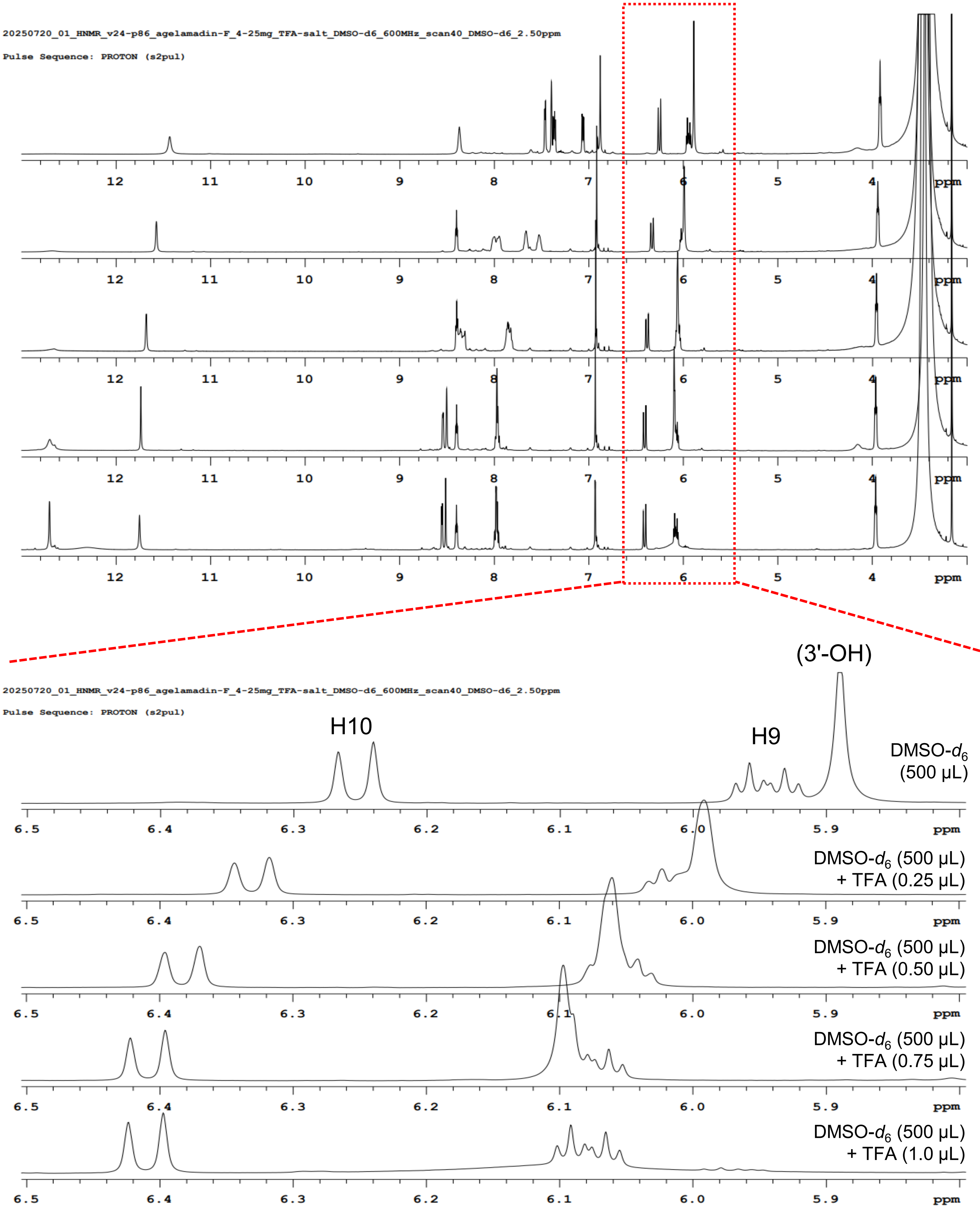

Figure S16. Comparison of ^{1}H NMR spectra showing stepwise addition of TFA (0, 0.25 μL, 0.50 μL, 0.75 μL, 1.0 μL) to agelamadin F (4.25 mg) in DMSO-d_6 (500 μL) (600 MHz).

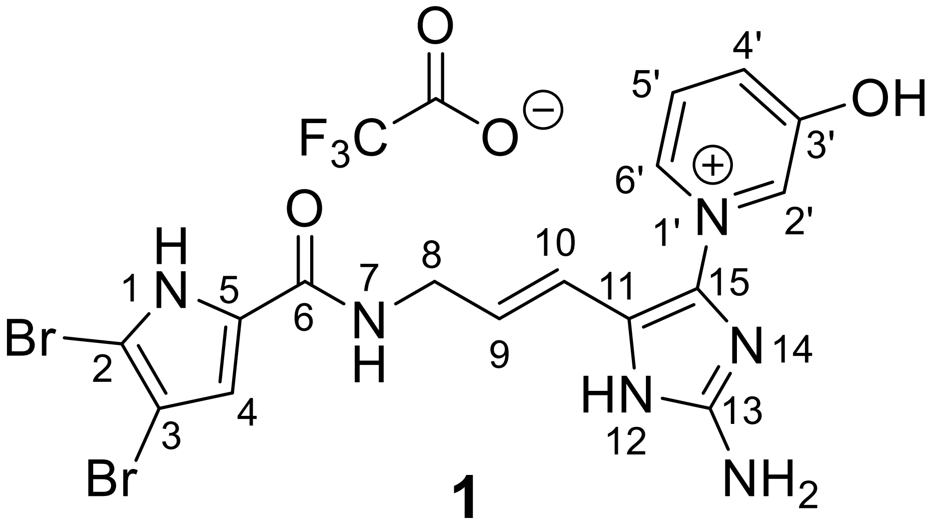

1

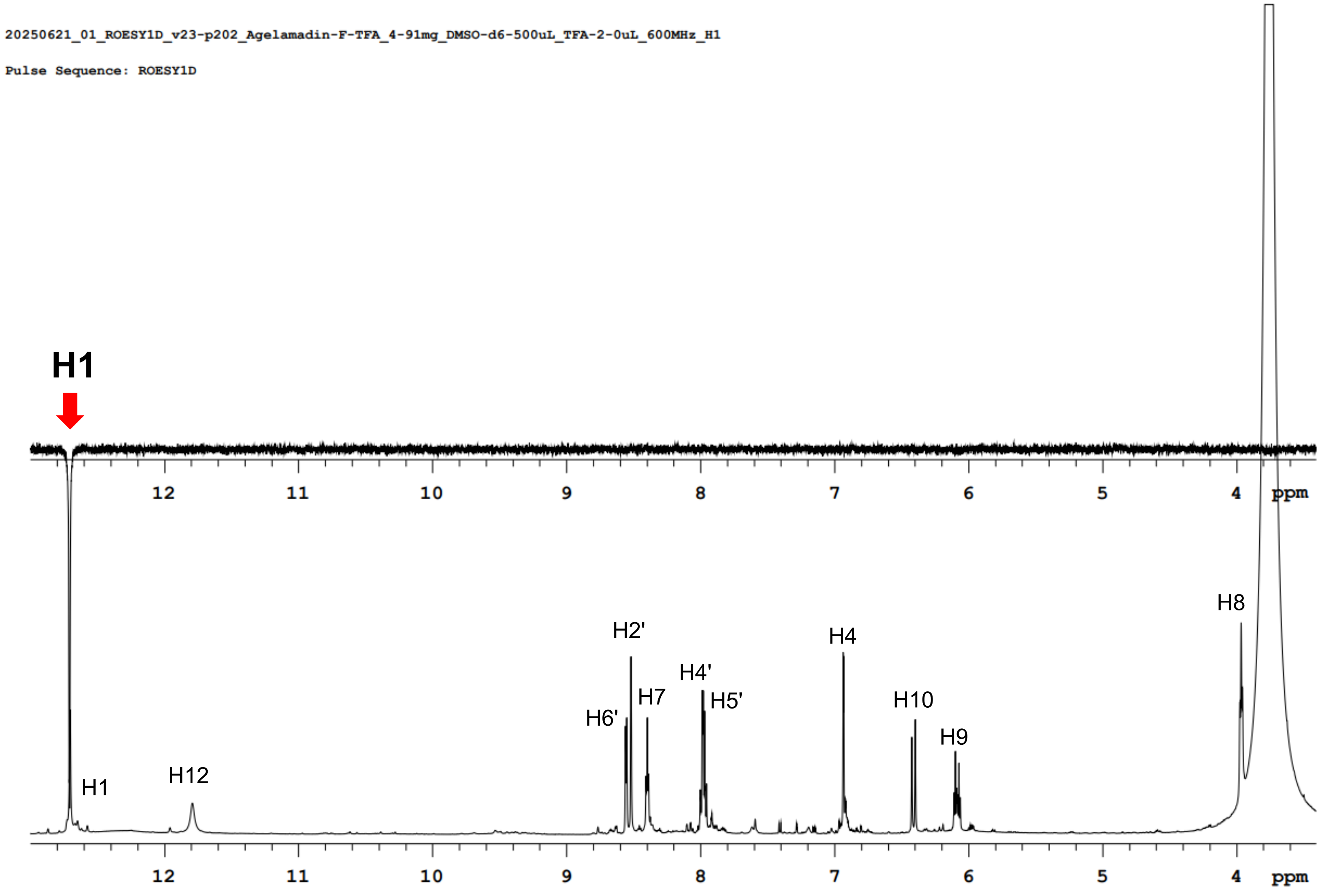

Figure S17. ROESY-1D spectrum of **1** (4.91 mg); irradiation at 11.79 ppm (600 MHz, DMSO-d_6: 500 μL; TFA: 2.0 μL).

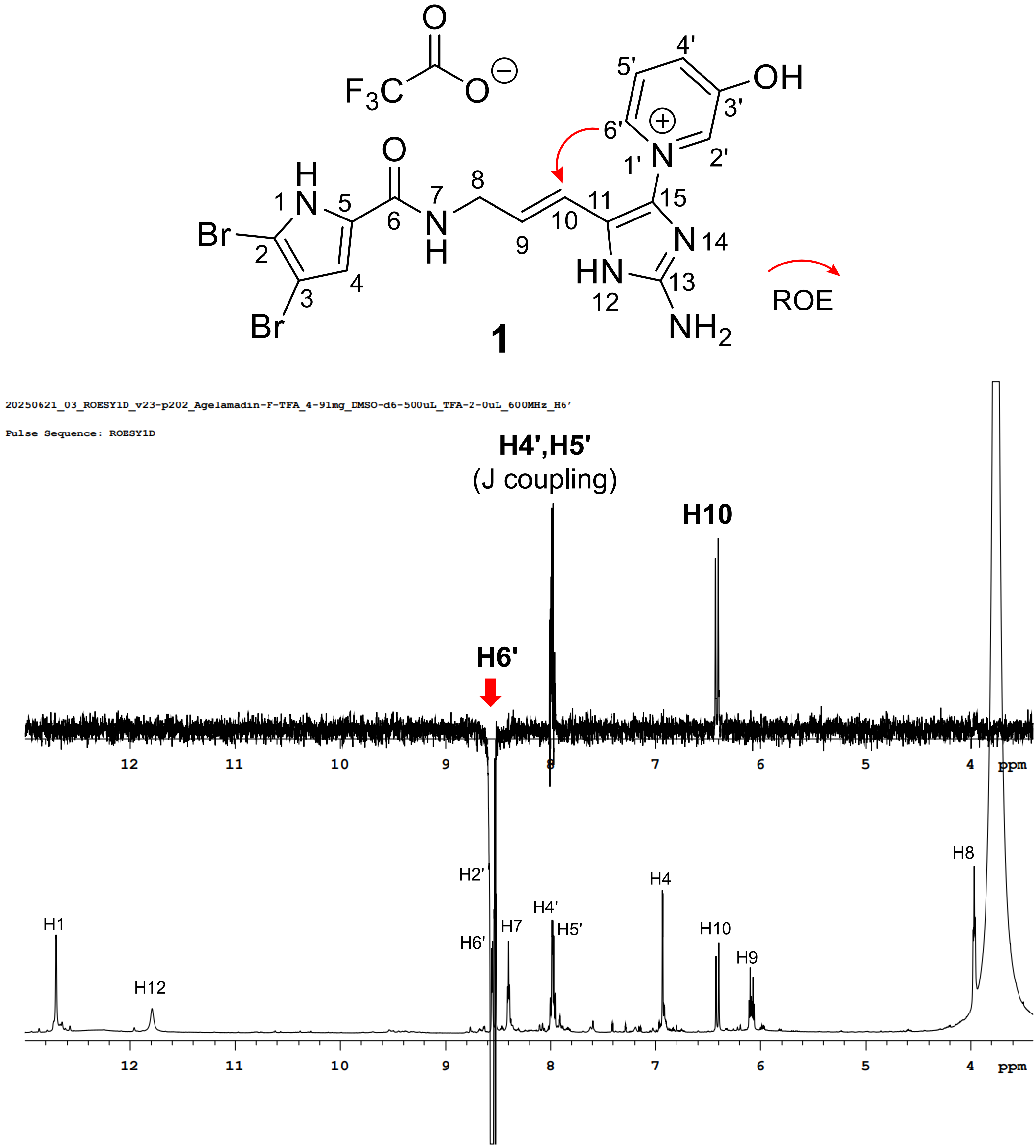

Figure S18. ROESY-1D spectrum of **1** (4.91 mg); irradiation at 8.56 ppm (600 MHz, DMSO-d_6: 500 µL; TFA: 2.0 µL).

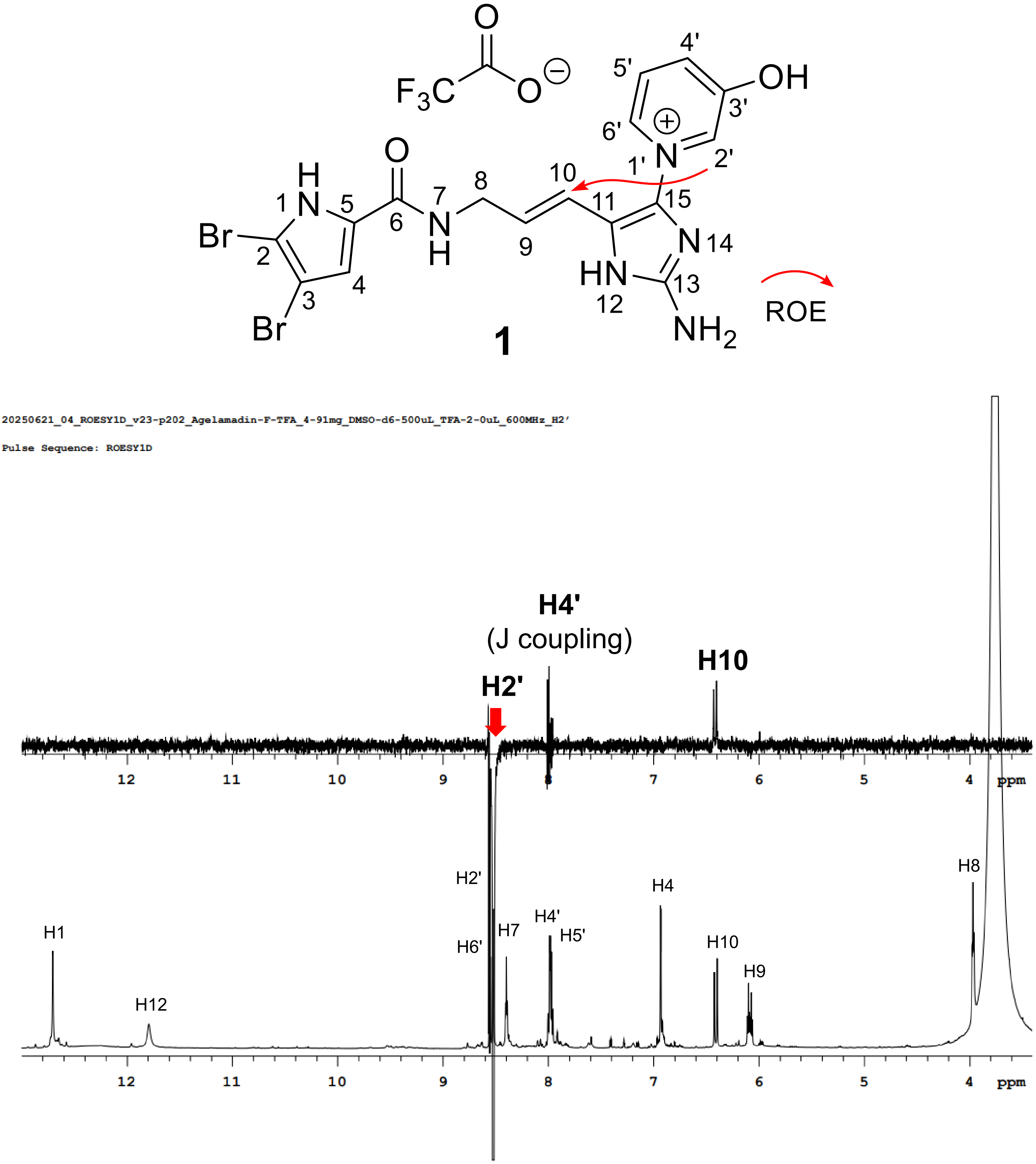

Figure S19. ROESY-1D spectrum of **1** (4.91 mg); irradiation at 8.52 ppm (600 MHz, DMSO-d_6: 500 μL; TFA: 2.0 μL).

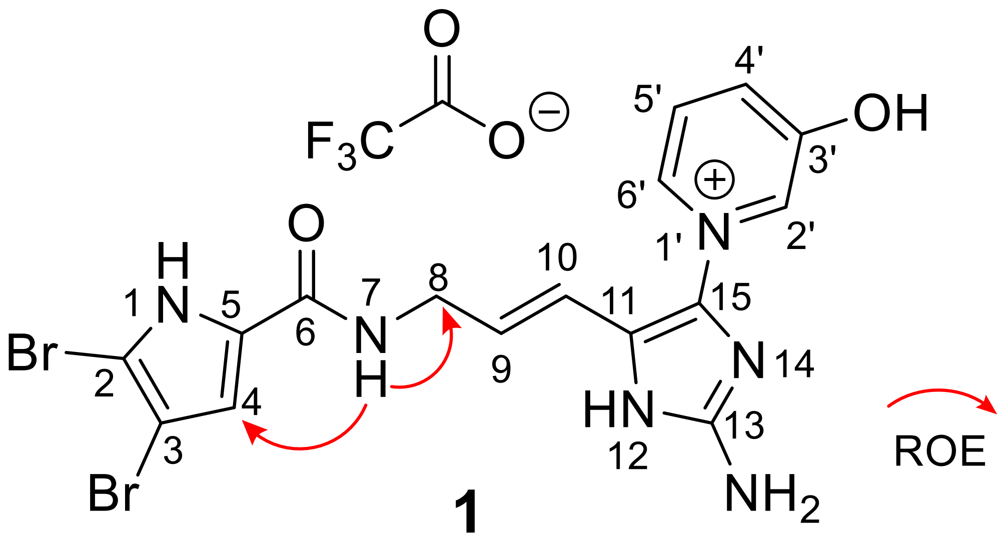

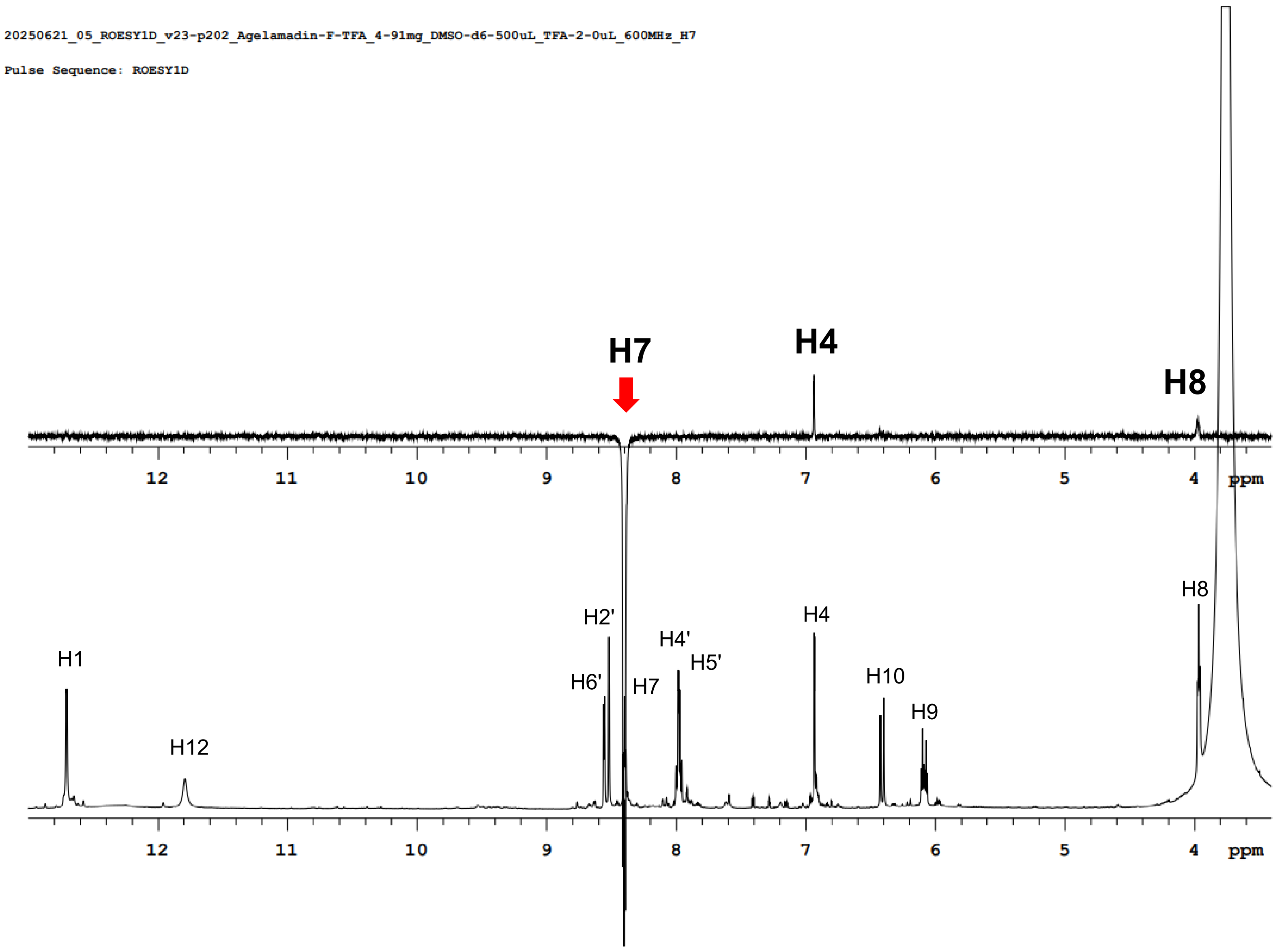

Figure S20. ROESY-1D spectrum of **1** (4.91 mg); irradiation at 8.40 ppm (600 MHz, DMSO-d_6: 500 µL; TFA: 2.0 µL).

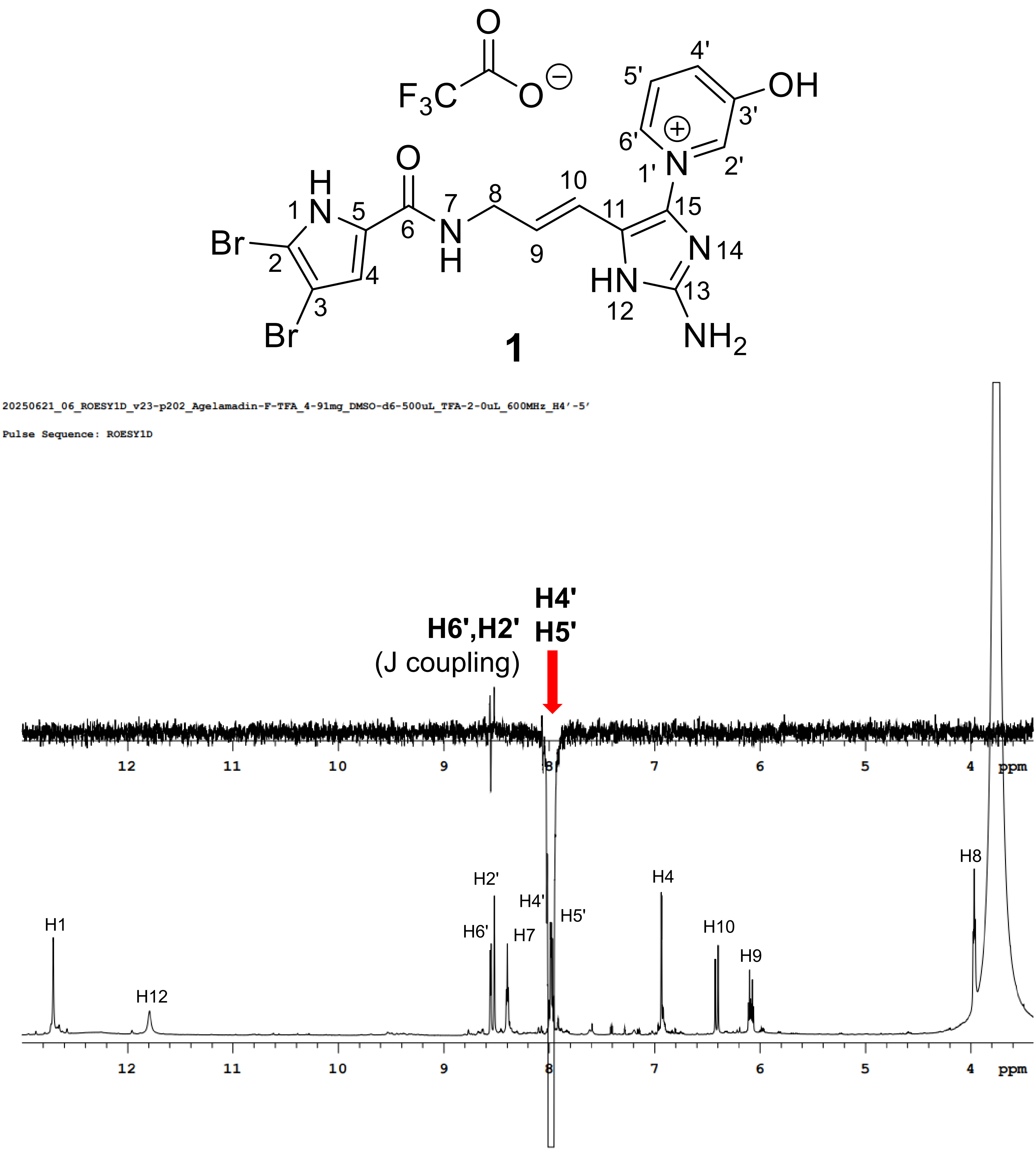

Figure S21. ROESY-1D spectrum of **1** (4.91 mg); irradiation at 7.98 ppm (600 MHz, DMSO-d_6: 500 μL; TFA: 2.0 μL).

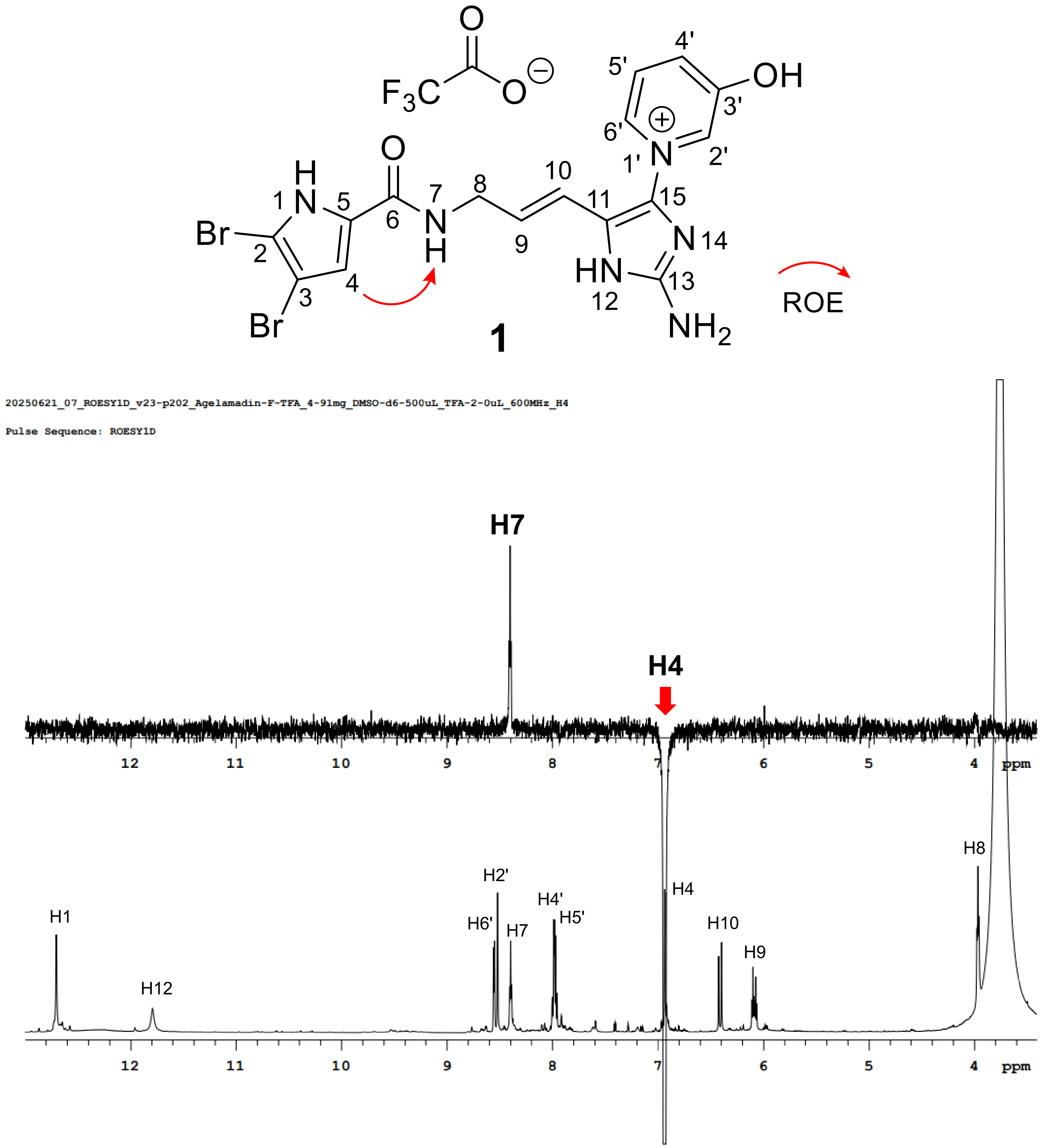

Figure S22. ROESY-1D spectrum of **1** (4.91 mg); irradiation at 6.94 ppm (600 MHz, DMSO-d_6: 500 µL; TFA: 2.0 µL).

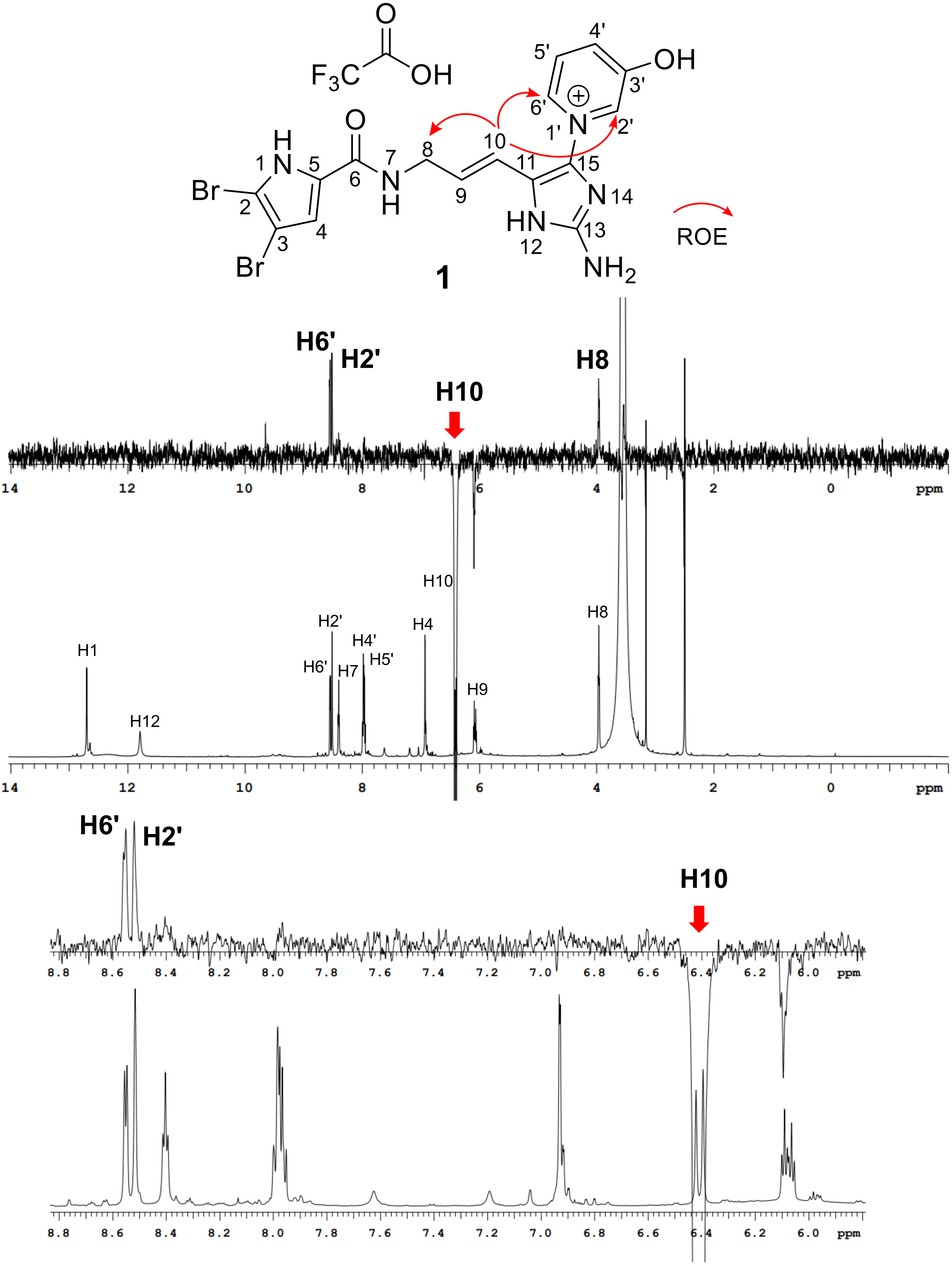

Figure S23. ROESY-1D spectrum of **1** (6.35 mg); irradiation at 6.41 ppm (600 MHz, DMSO-d_6: 500 μL; TFA: 2.5 μL).

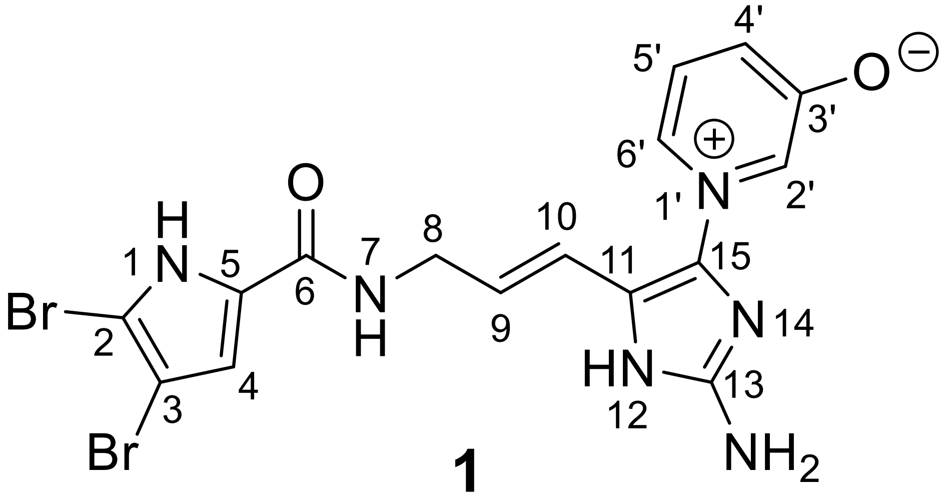

1

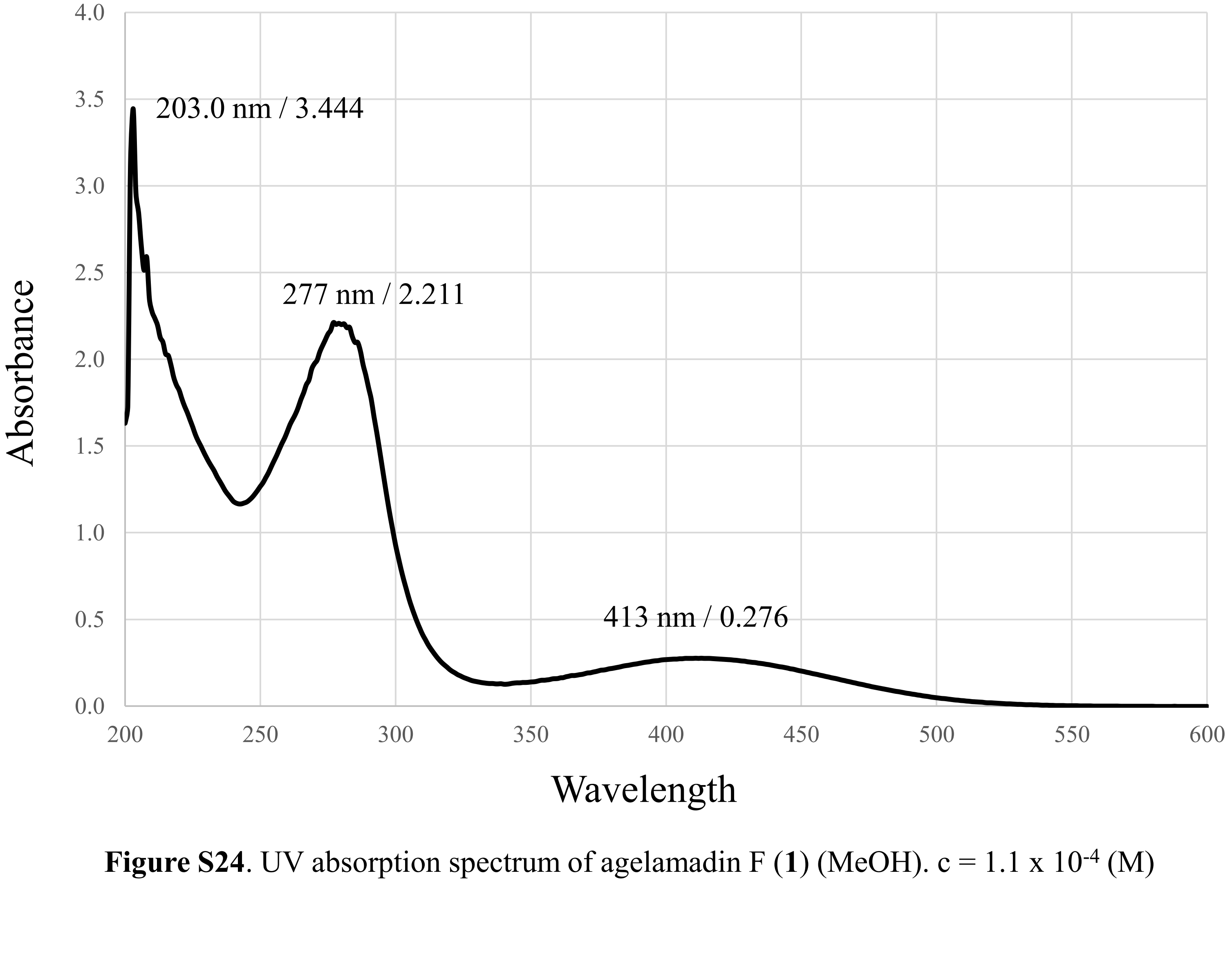

Figure S24. UV absorption spectrum of agelamadin F (**1**) (MeOH). c = 1.1 x 10^{-4} (M)

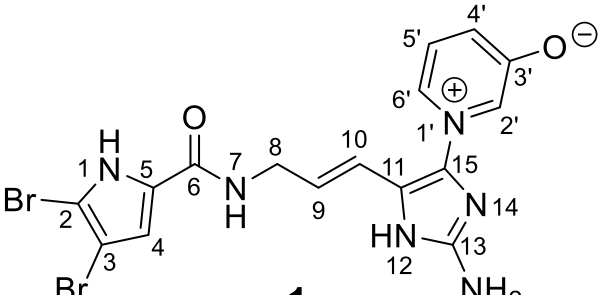

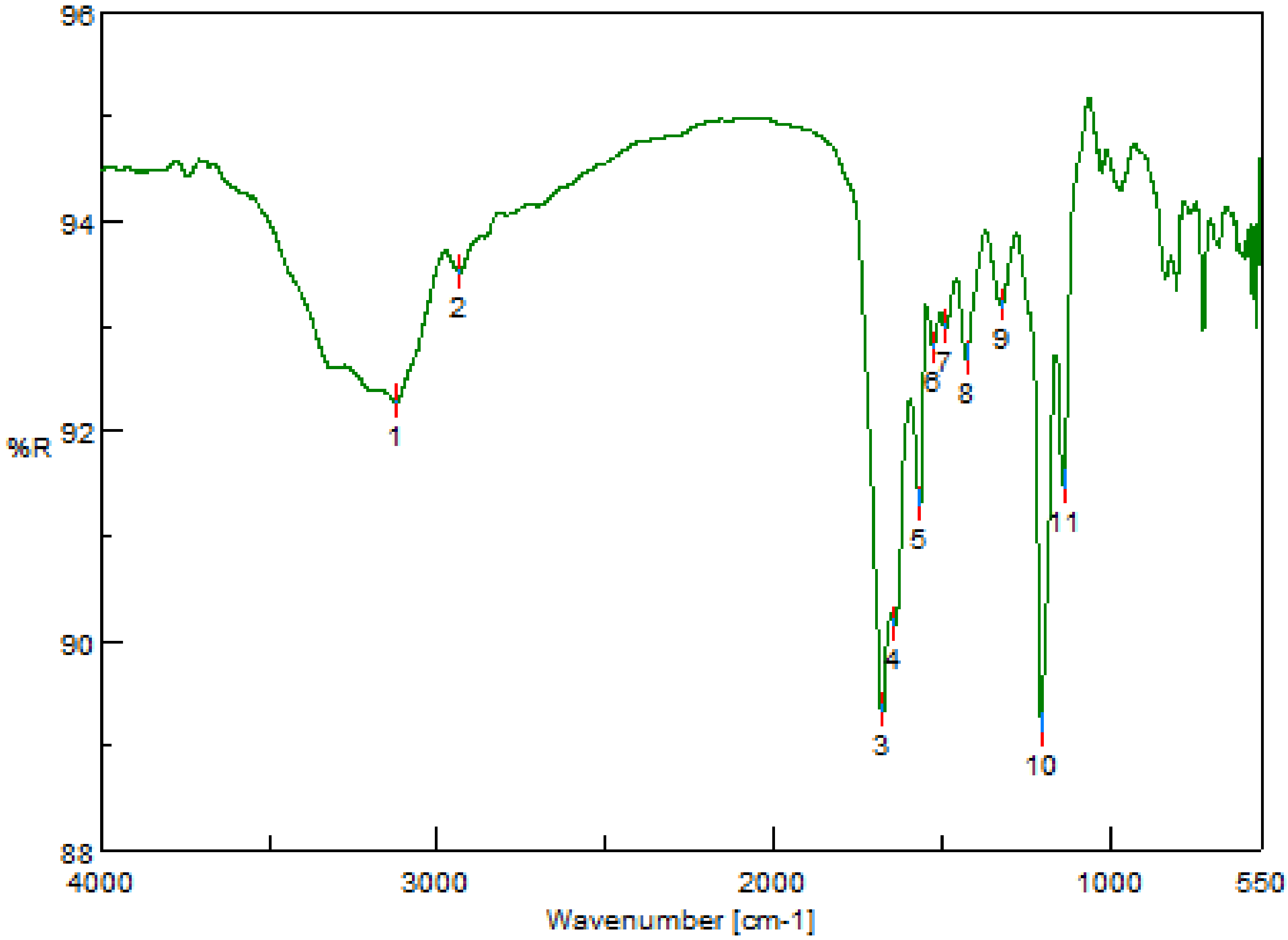

No.	Wavenumber	Strength
1	3121.2	92.2823
2	2934.2	93.5033
3	1680.7	89.3268
4	1640.2	90.1557
5	1564.0	91.3024
6	1525.4	92.7882
7	1487.8	92.9885
8	1425.1	92.6979
9	1323.9	93.1963
10	1203.4	89.1358
11	1135.9	91.4629

Figure S25. IR spectrum of **1** (ATR).

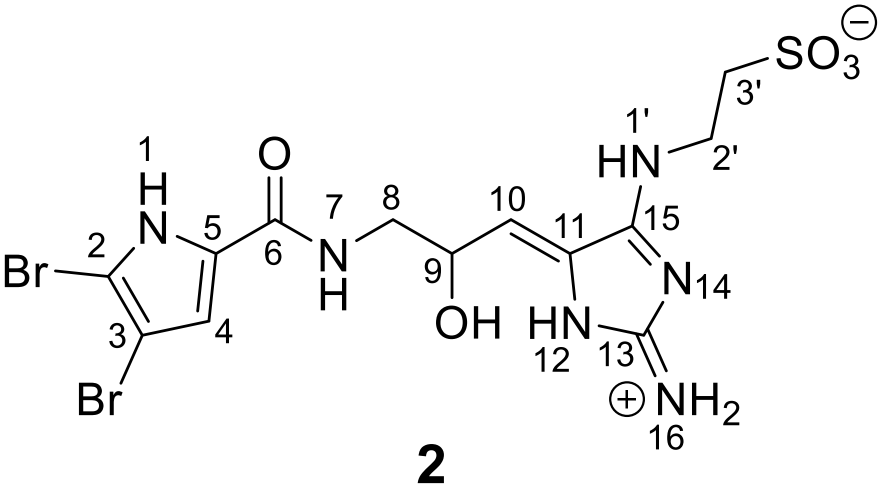

2

Tauroacidin A (**2**):

$\mathbf{R_f}$ = 0.18 (broad) ($CHCl_3$/MeOH/28% NH_3 aq. = 55:45:2, v/v/v; UV).

UV/vis λ_{max} (MeOH) nm (log ε): 306 (3.92), 272 (4.41), 200 (4.53).

^{1}H and ^{13}C NMR: See Table S3.

IR ν_{max}: 3253 (br), 2890 (w), 2770 (w), 1688 (s), 1636 (s), 1529 (m), 1430 (m), 1331 (w), 1204 (s).

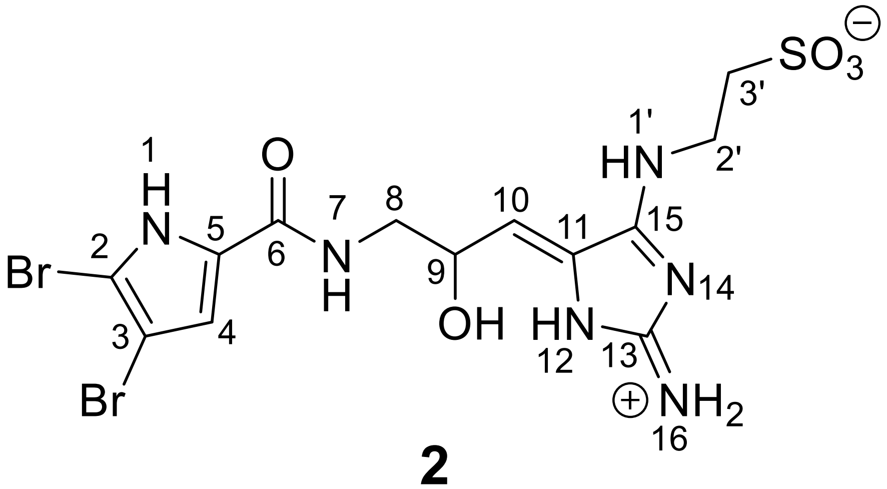

2

Table S3. ^{1}H (600 MHz), ^{13}C (151 MHz) and ^{15}N (60.8 MHz) NMR data for **2** containing TFA, and reported data for **2** (δ in ppm).

	Synthetic tauroacidin A (**2**)				Natural **2** (reported)[2]			Δδ (Synthetic **2** - Natural **2**)	
Position	δ_N	δ_C, type	δ_H (J in Hz)	^{1}H-^{13}C HMBC	δ_C, type	δ_H (J in Hz)	^{1}H-^{13}C HMBC	δ_C	δ_H
1	165.8		12.72, s	3, 4		12.70, br. s	3, 4		0.02
2		104.8, C	-		104.7, C	-		0.1	
3		97.9, C	-		97.8, C	-		0.1	
4		113.0, CH	6.95, d (1.8)	2, 5	113.0, CH	6.95, s	2, 5, 6	0.0	0.00
5		128.0, C	-		127.9, C	-		0.1	
6		159.3, C	-		159.1, C	-		0.2	
7	107.3		8.25, t (5.7)	6		8.22, t (6.0)	6		0.03
8		44.6, CH_2	3.33[a]	6, 9, 10	44.5, CH_2	3.32, m		0.1	0.01
9		67.3, CH	4.60, q (6.0)	8, 10, 11	67.3, CH	4.61, m		0.0	-0.01
9-OH			5.97, br. s			5.94 d (4.2)			0.03
10		116.5, CH	6.16, d (6.0)	8, 9, 11, 15	116.4, CH	6.17, d (4.2)	11, 15	0.1	-0.01
11		131.2, C	-		131.2, C	-		0.1	
12	113.1		10.55, s	11, 13, 15	-	10.51, s[b]	13, 15[b]		0.04
13		165.9, C	-		165.9, C	-		0.0	
14			-		-	-			
15		167.7, C	-		167.6, C	-		0.1	
16	92.5		9.32, s			9.31, br. s[d]			0.01
			8.09, br. s			8.05, br. s			0.04
1'	107.9		9.66, t (5.6)	15	-	9.64, br. t (3.0)	11, 15		0.02
2'		39.8, CH_2 (^{1}H-^{13}C HSQC)[c]	3.66, m	15, 3'	39.8, CH_2	3.67, dt (7.2, 3.0)		0.0	-0.01
3'		49.2, C	2.75, t (7.2)	2'	49.1, CH_2	2.75, t (7.2)		0.1	0.00

[a]C8-H signal at 3.33 ppm was overlayed by HDO signal, so this signal was determined by COSY, TOCSY and ^{1}H-^{13}C HSQC.

[b]The signal at 10.51 ppm was originally assigned at N14-H in the reported paper.

[c]C8 signal at 39.7 ppm was overlayed by DMSO ($-CH_3$) signal, so this signal was determined by ^{1}H-^{13}C HSQC.

[d]The signal at 9.31 ppm was originally assigned at N12-H in the reported paper.

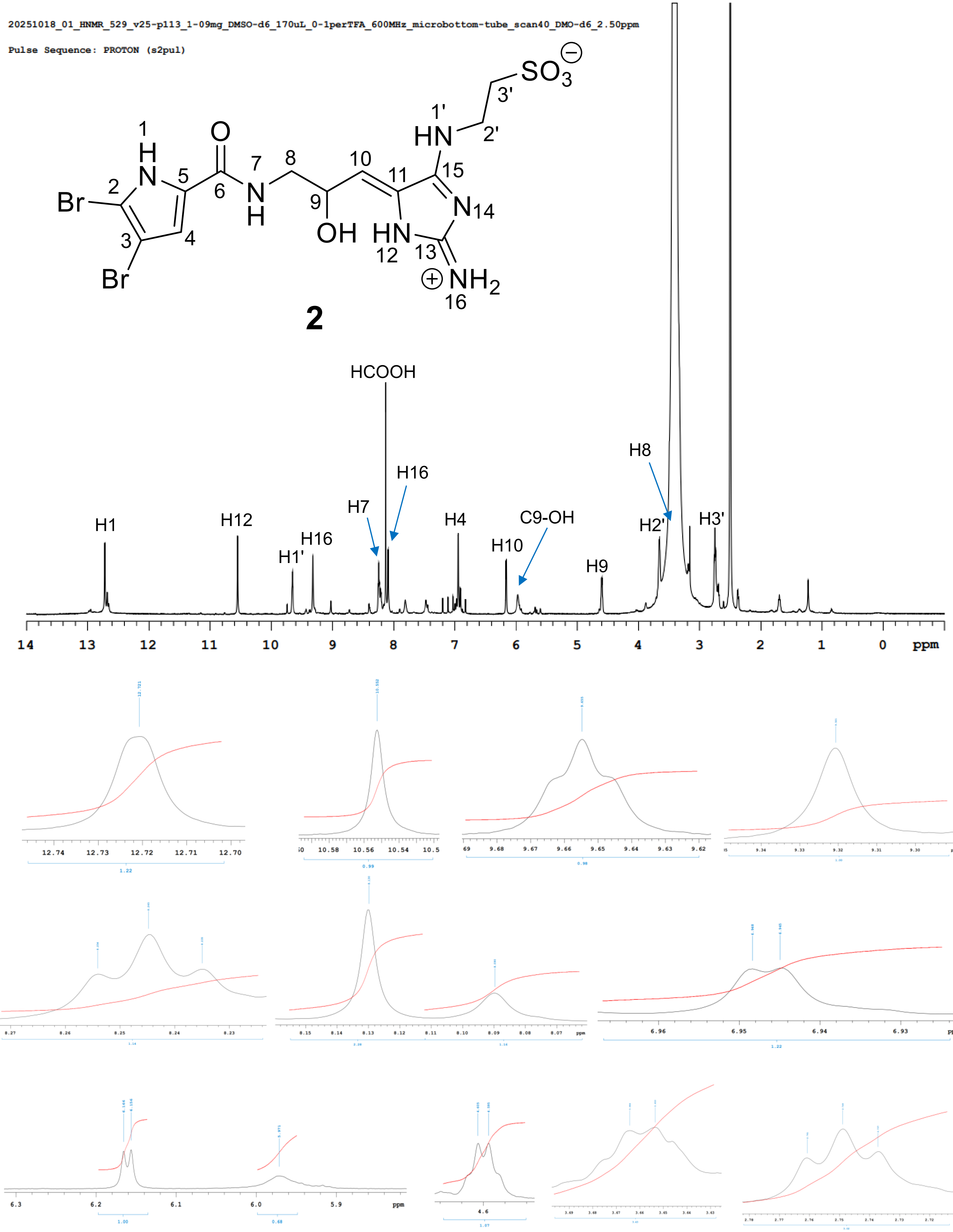

Figure S26. ^{1}H NMR spectrum of **2** (1.09 mg) (600 MHz, DMSO-d_6: 190 µL - 0.1% TFA).

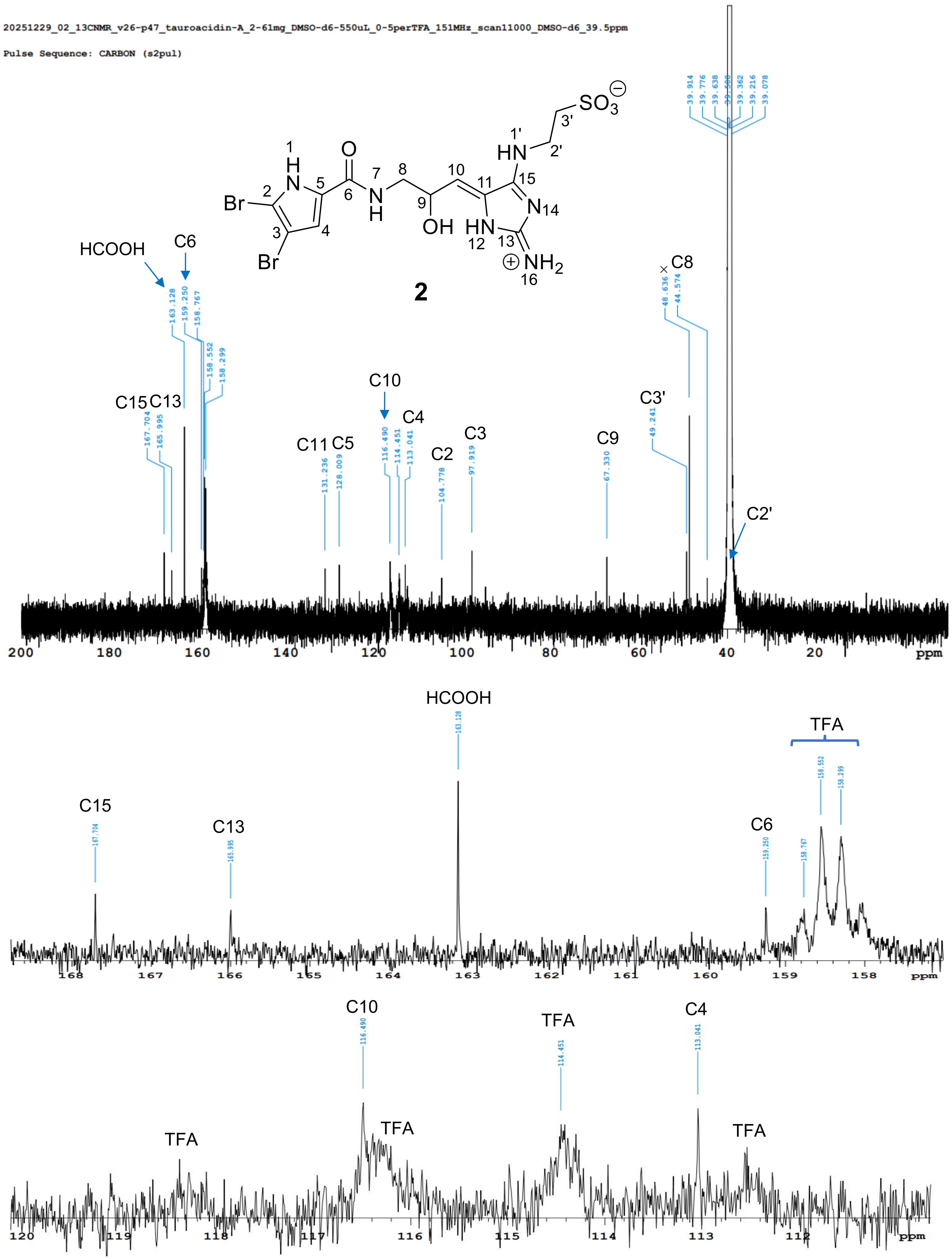

Figure S27. ^{13}C NMR spectrum of **2** (2.61 mg) (151 MHz, DMSO-d_6: 550 µL - 0.5% TFA).

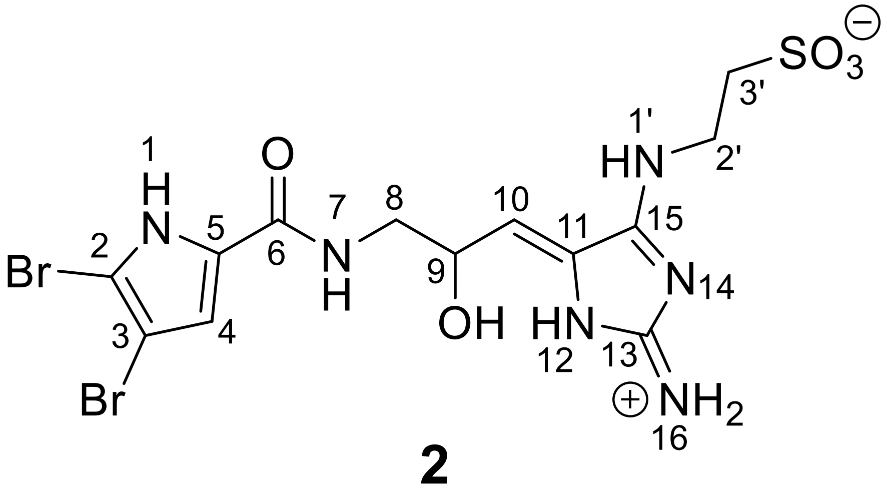

2

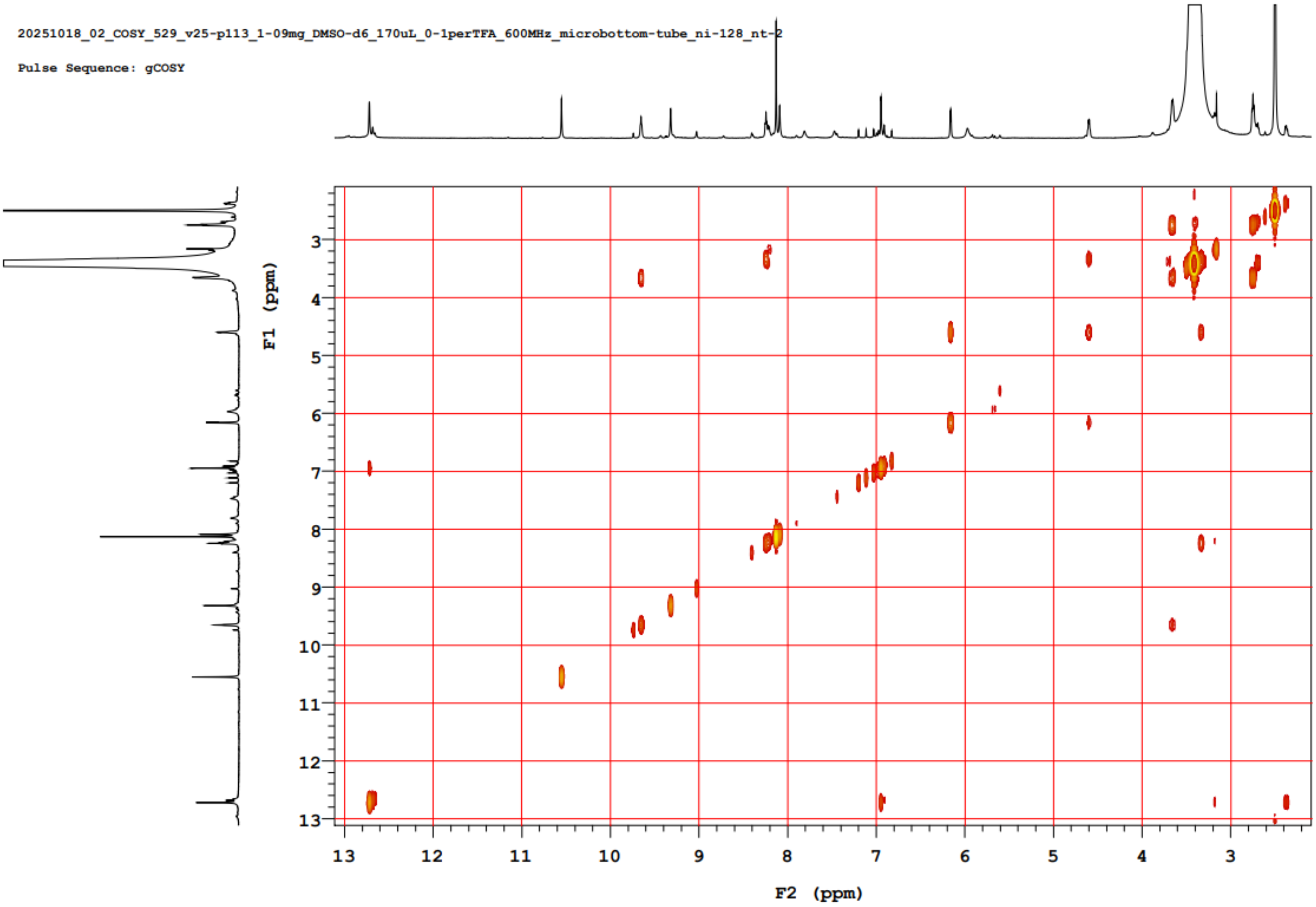

Figure S28. COSY spectrum of **2** (1.09 mg) (600 MHz, DMSO-d_6: 190 µL - 0.1% TFA).

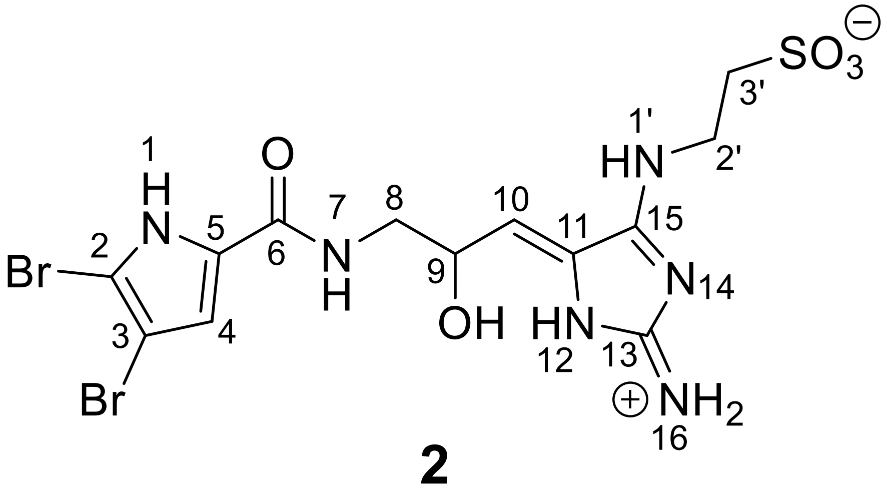

2

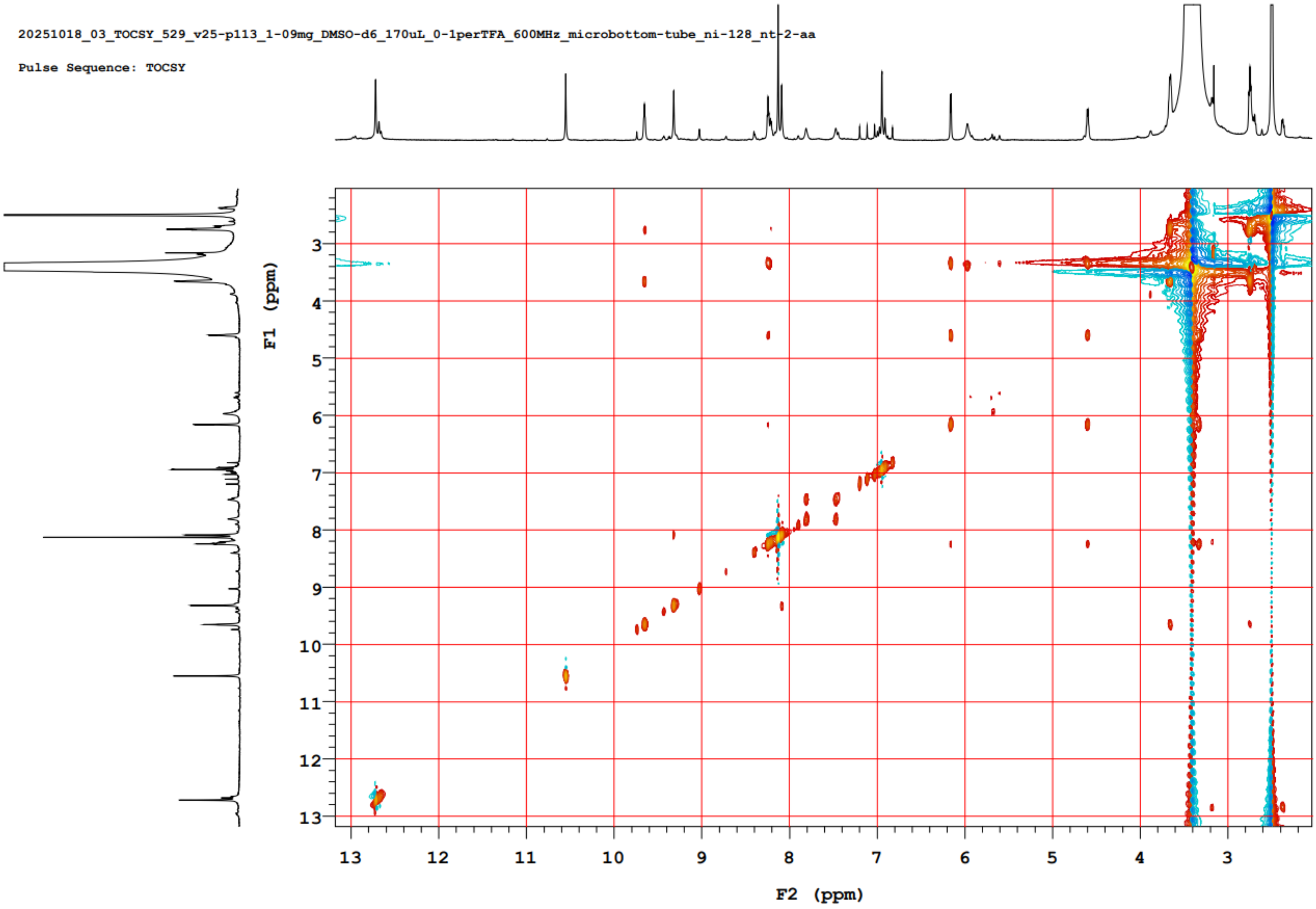

Figure S29. TOCSY spectrum of **2** (1.09 mg) (600 MHz, DMSO-d_6: 190 µL - 0.1% TFA).

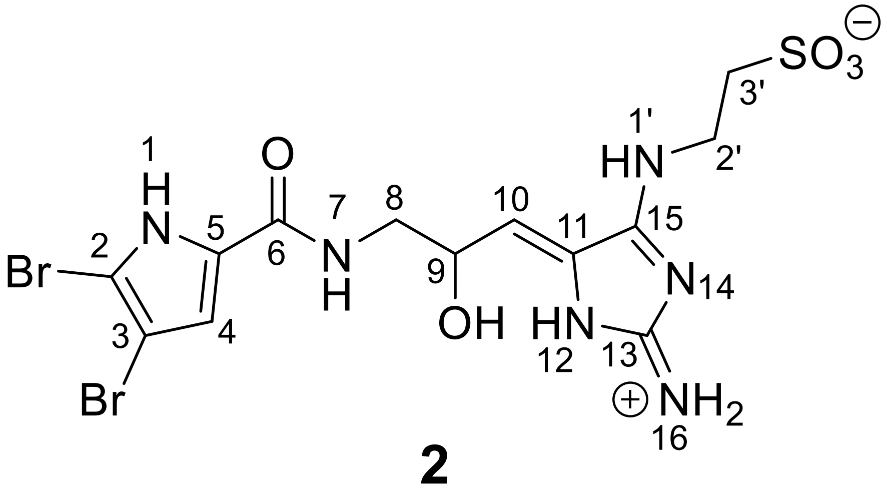

2

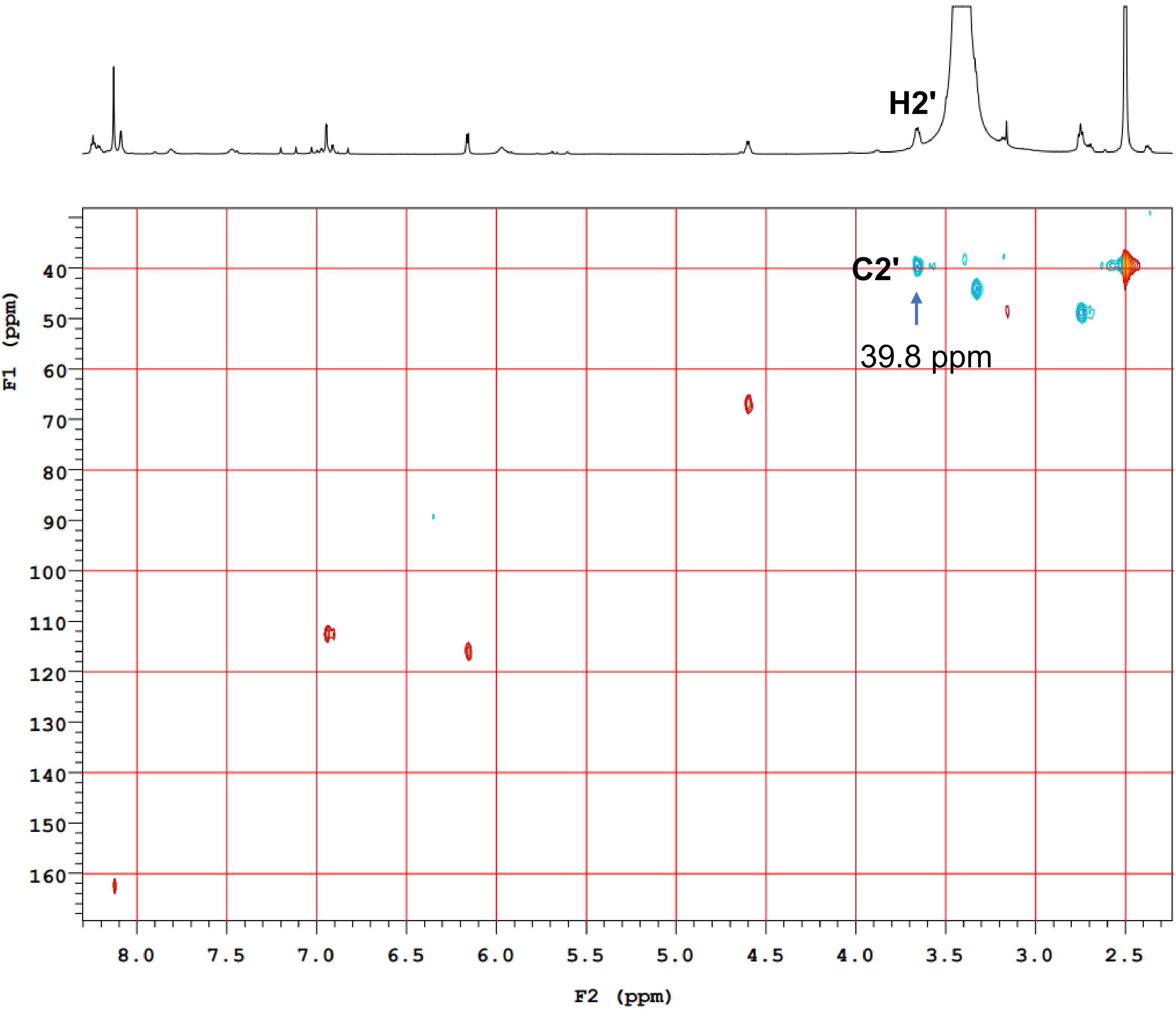

Figure S30. ^{1}H-^{13}C HSQC spectrum of **2** (1.09 mg) (600 MHz / 151 MHz, DMSO-d_6: 190 µL - 0.1% TFA).

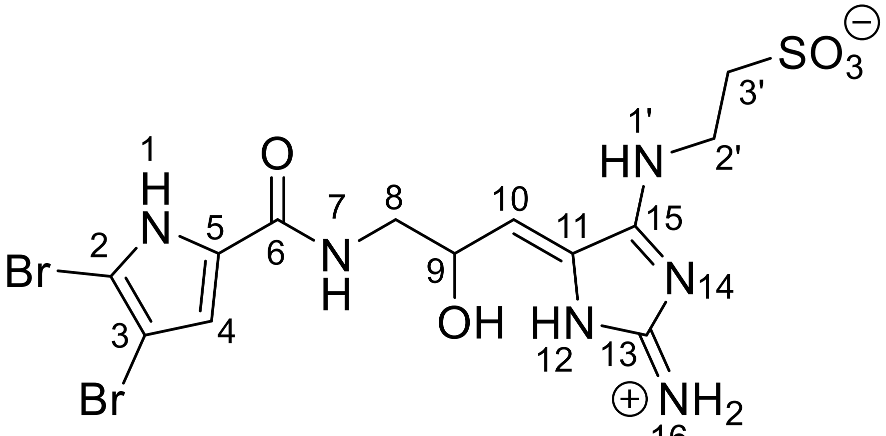

2

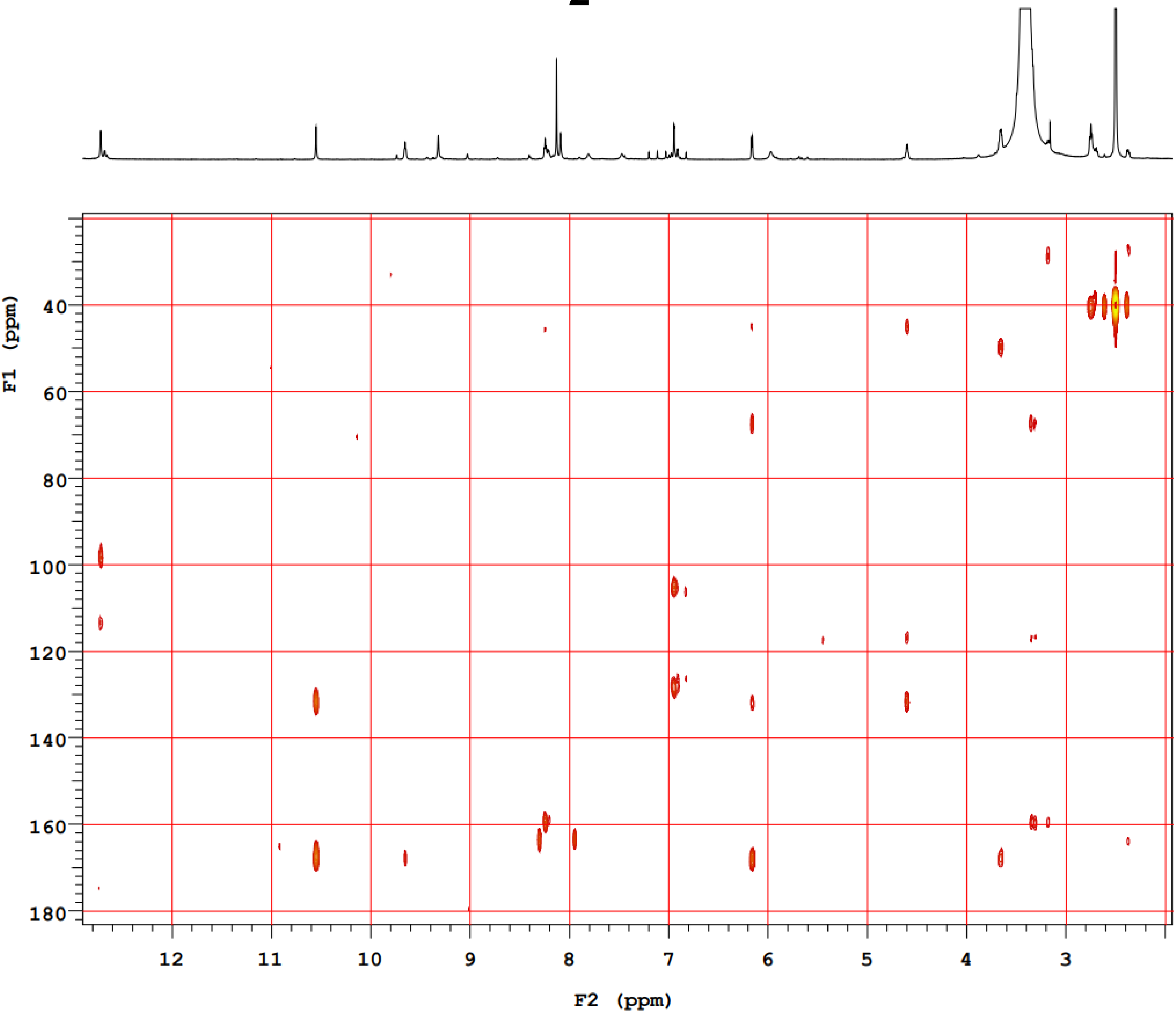

Figure S31. ^{1}H-^{13}C HMBC spectrum of **2** (1.09 mg) (600 MHz / 151 MHz, DMSO-d_6: 190 µL - 0.1% TFA).

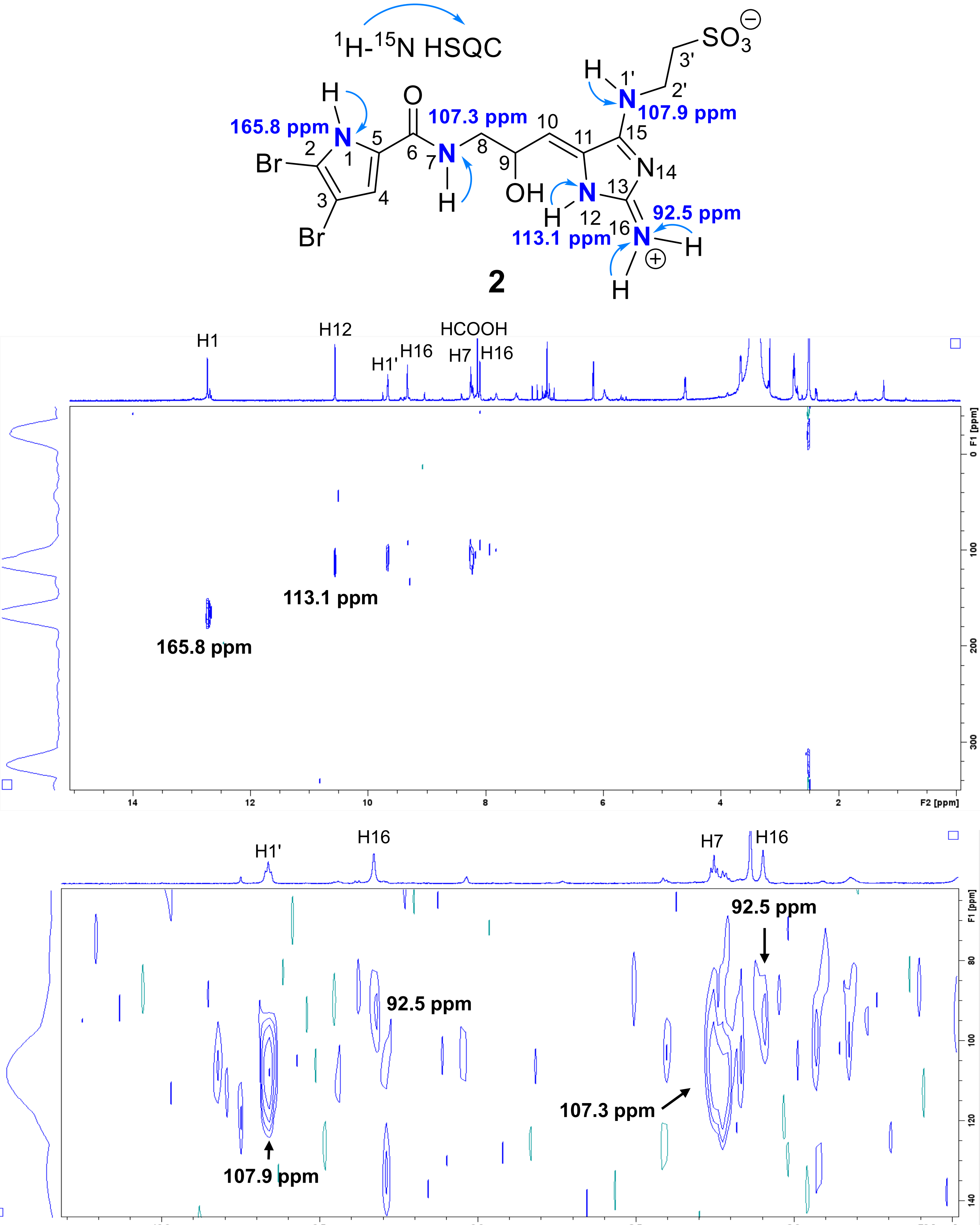

Figure S32. ^{1}H-^{15}N HMBC spectrum of **2** (1.09 mg) (600 MHz / 60.8 MHz, DMSO-d_6: 190 µL - 0.1% TFA).

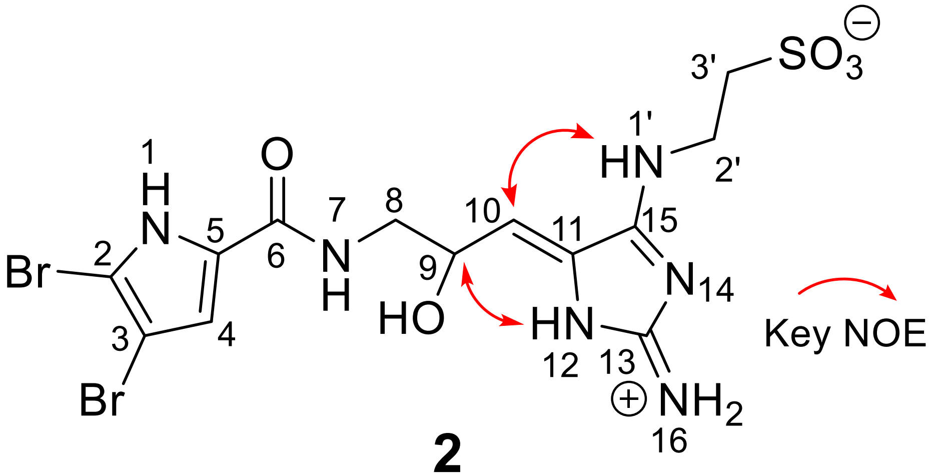

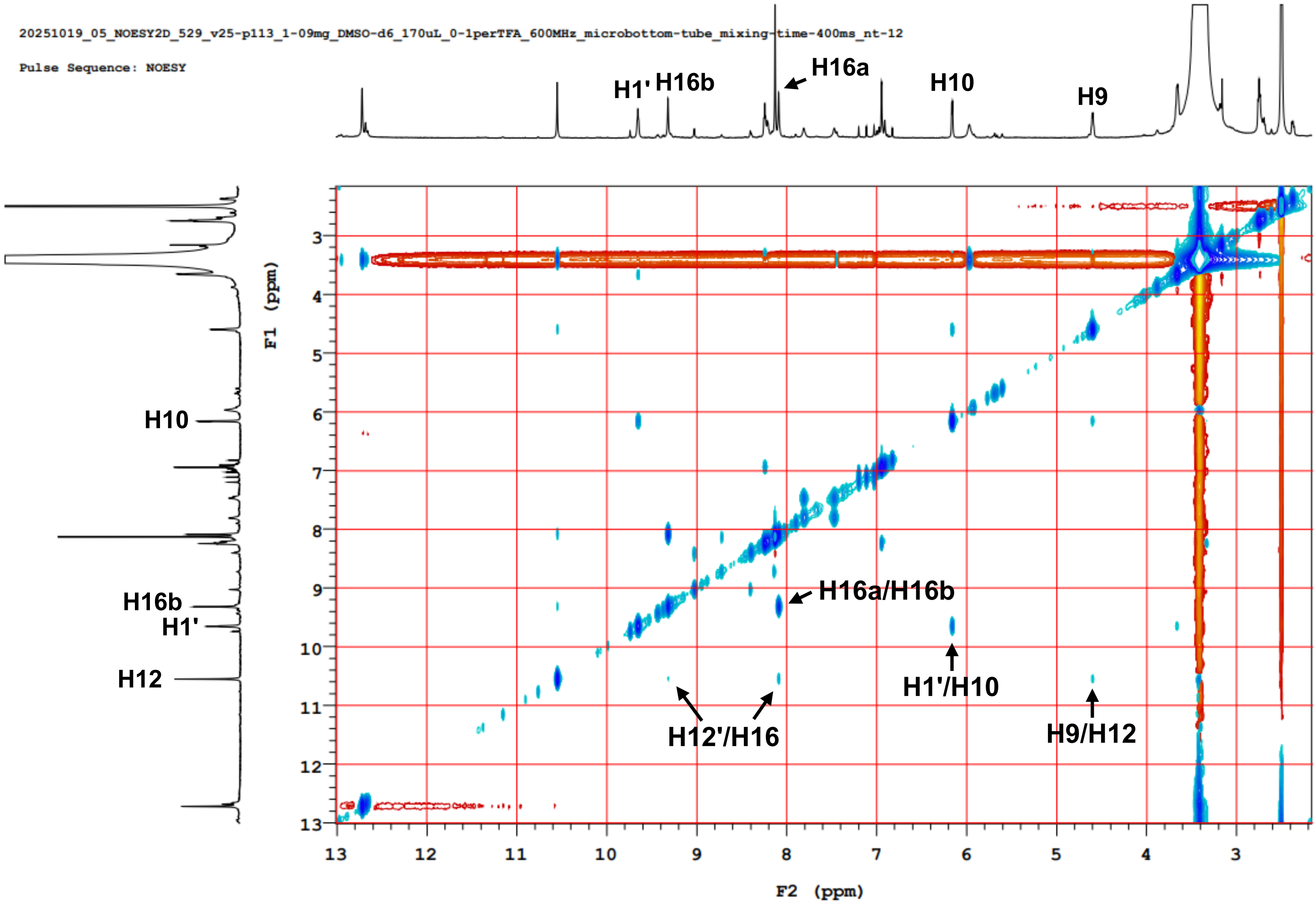

Figure S33. NOESY spectrum of **2** (1.09 mg) (600 MHz, DMSO-d_6: 190 µL - 0.1% TFA).

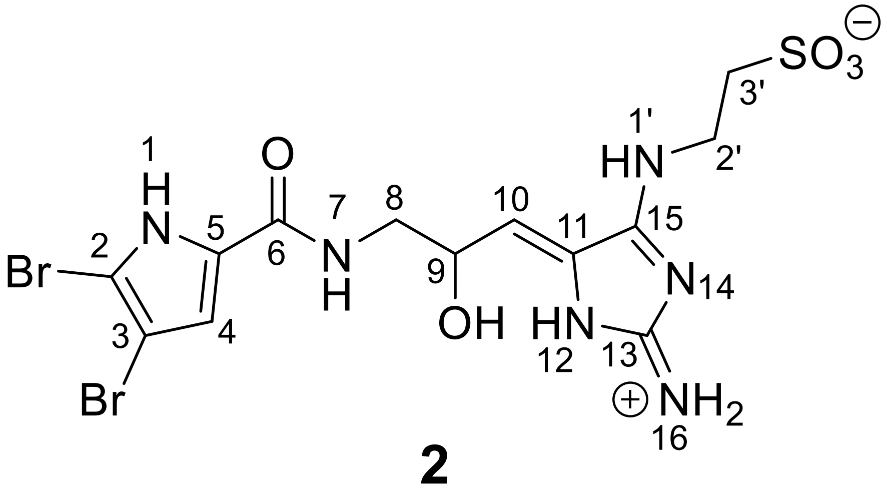

2

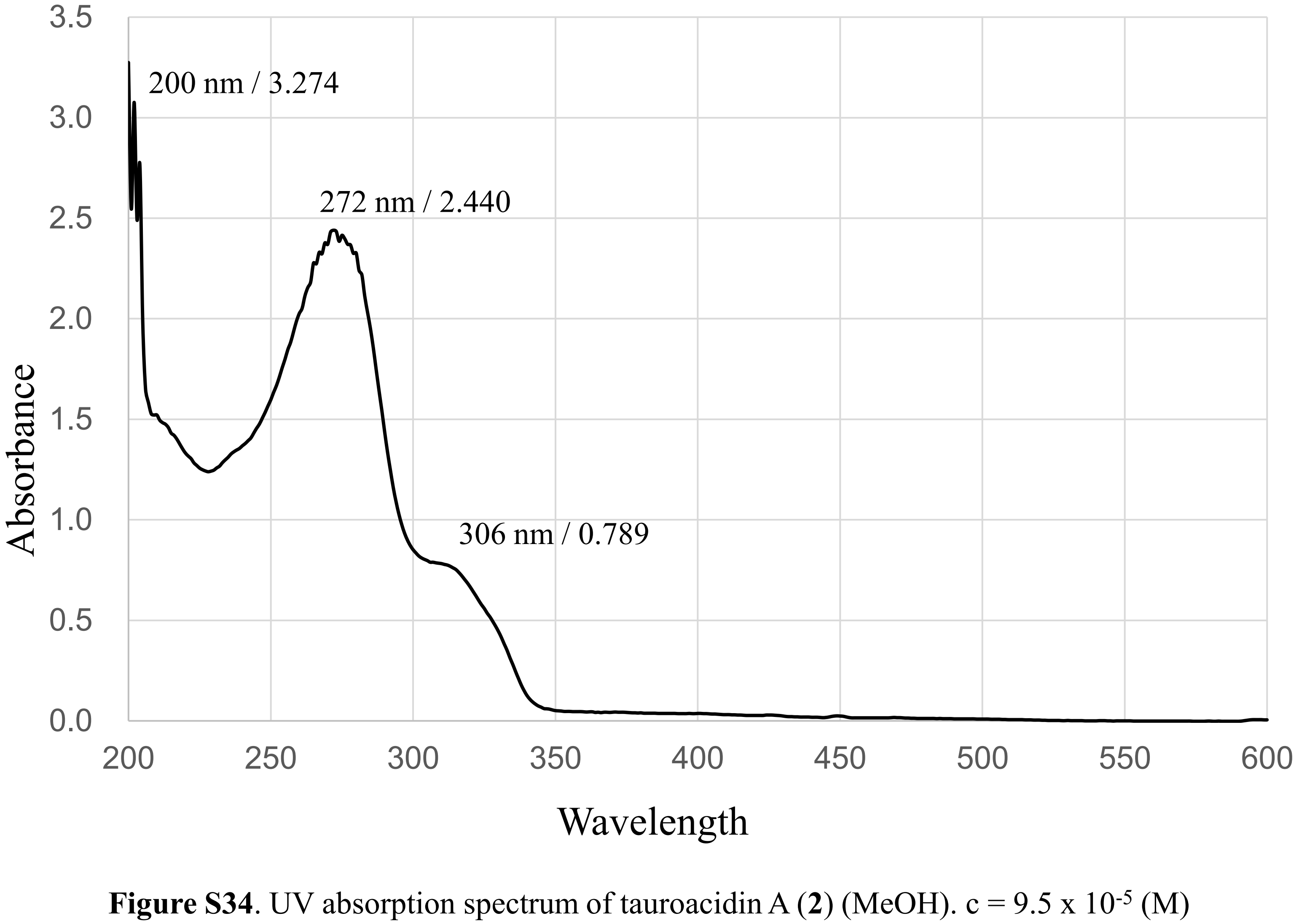

Figure S34. UV absorption spectrum of tauroacidin A (**2**) (MeOH). c = 9.5 x 10^{-5} (M)

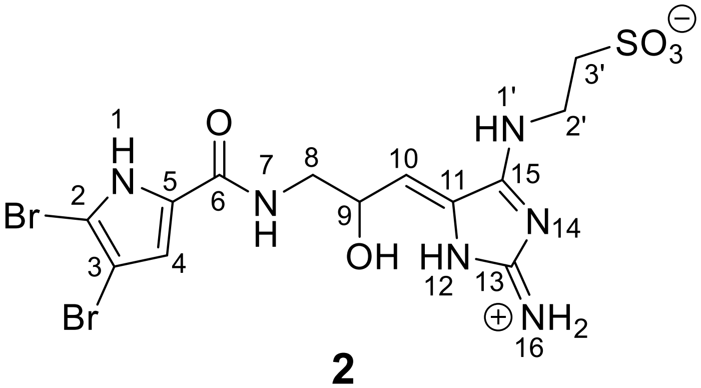

2

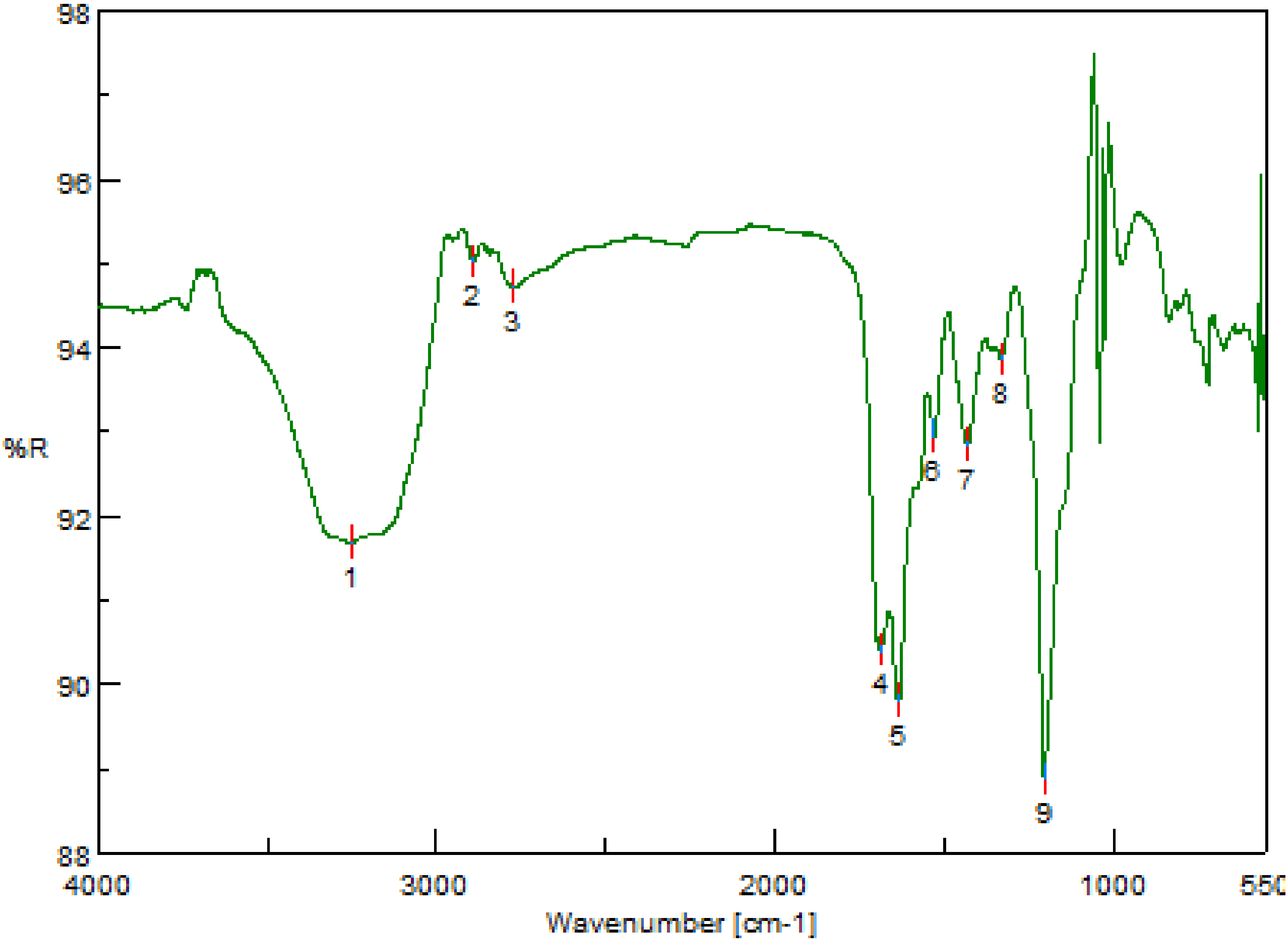

No.	Wavenumber	Strength
1	3253.3	91.6728
2	2889.8	95.0117
3	2770.2	94.7127
4	1688.4	90.3961
5	1636.3	89.8078
6	1529.3	92.951
7	1430.0	92.8457
8	1330.6	93.8529
9	1204.3	88.868

Figure S35. IR spectrum of **2** (ATR).

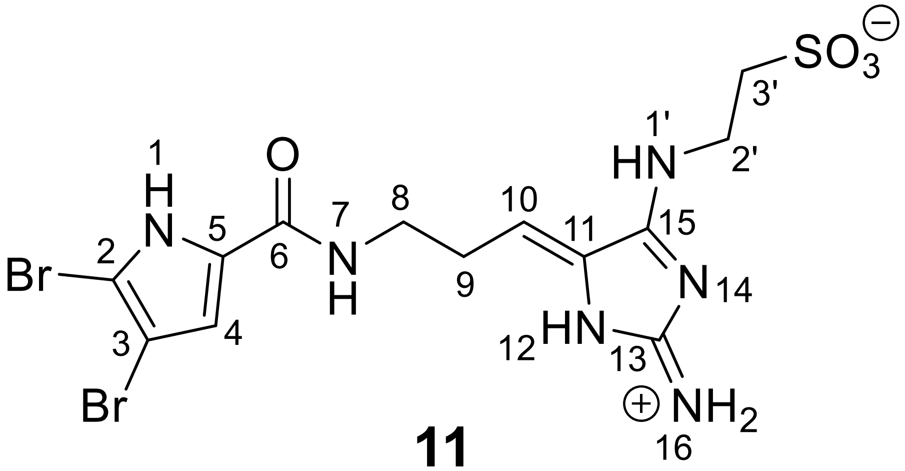

11

11:

R_f = 0.27 ($CHCl_3$/MeOH/28% NH_3 aq. = 60:40:2, v/v/v; UV).

UV/vis λ_{max} (MeOH) nm (log ε): 306.0 (3.73), 272.0 (4.13), 200 (4.44).

^{1}H NMR (600 MHz, CD_3OD): δ 6.79 (s, C4-H, 1H), 6.09 (t, J = 7.8 Hz, C10-H, 1H), 3.88 (t, J = 6.6 Hz, C2'-H, 2H), 3.46 (t, J = 6.6 Hz, C8-H, 2H), 3.12 (t, J = 6.9 Hz, C3'-H, 2H), 2.58 (q, J = 7.0 Hz, C9-H, 2H).

^{1}H NMR (600 MHz, DMSO-d_6 containing 0.1% TFA): δ 12.72 (s, N1-H, 1H), 11.18 (s, N12-H, 1H), 9.59 (t, J = 5.4 Hz, N1'-H, 1H), 9.23 (s, N16-H, 1H), 8.37 (s, N16-H, 1H), 8.27 (t, J = 5.4 Hz, N7-H, 1H), 6.92 (d, J = 3.0 Hz, C4-H, 1H), 6.13 (t, J = 7.8 Hz, C10-H, 1H), 3.65 (q, J = 6.6 Hz, C2'-H, 2H), 3.34 (q, J = 6.4 Hz, C8-H, 2H), 2.73 (t, J = 7.5 Hz, C3'-H, 2H), 2.54 (q, J = 6.8 Hz, C9-H, 2H).

^{13}C NMR (151 MHz, CD_3OD) (HMBC, HSQC): 169.6 (C15), 162.5 (C6), 135.4 (C11), 129.2 (C5), 116.2 (C10), 114.6 (C4), 106.8 (C2), 50.8 (C3'), 40.9 (C2'), 39.7 (C8), 29.8 (C9). C3 and C13 were not detected.

^{13}C NMR (151 MHz, DMSO-d_6 containing 0.1% TFA) (HMBC, HSQC): 167.1 (C15), 166.4 (C13), 159.0 (C6), 132.9 (C11), 114.1 (C10), 112.3 (C4), 48.9 (C3'), 39.6 (C2'), 37.5 (C8), 27.7 (C9). C2, C3 and C5 were not detected.

IR v_{max}: 3121 (br), 1689 (s), 1636 (s), 1530 (w), 1428 (w), 1202 (s), 1147 (m), 1038 (w).

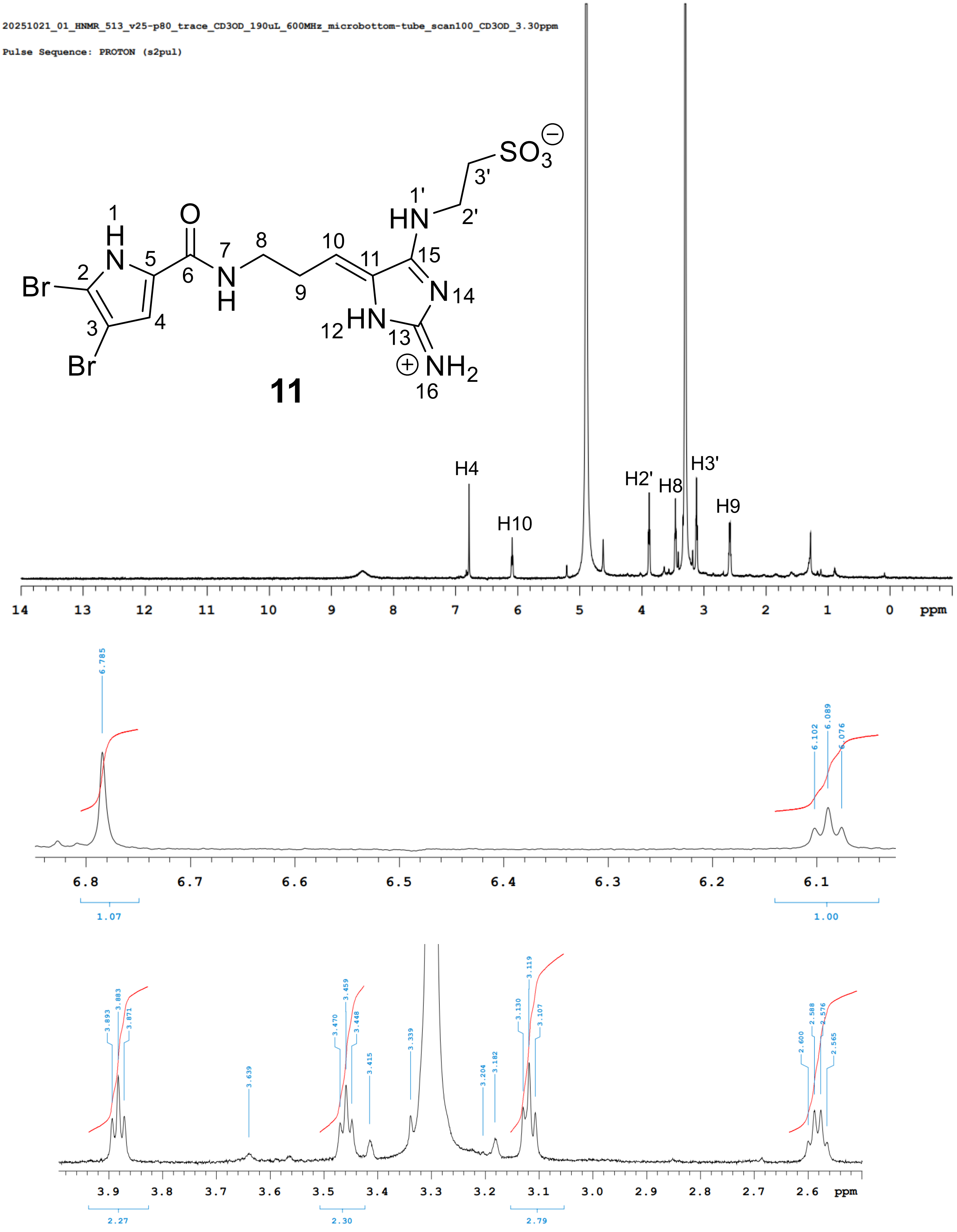

Figure S36. ^{1}H NMR spectrum of **11** (0.30 mg) (600 MHz, CD_3OD: 190 μL).

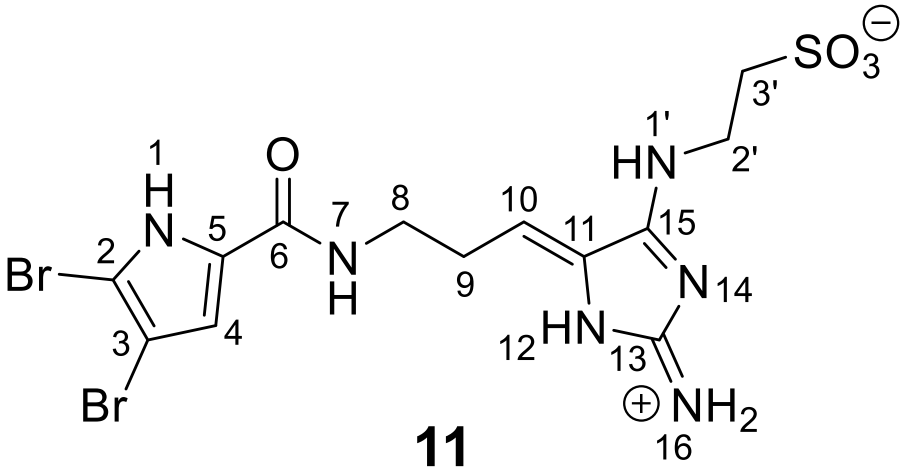

11

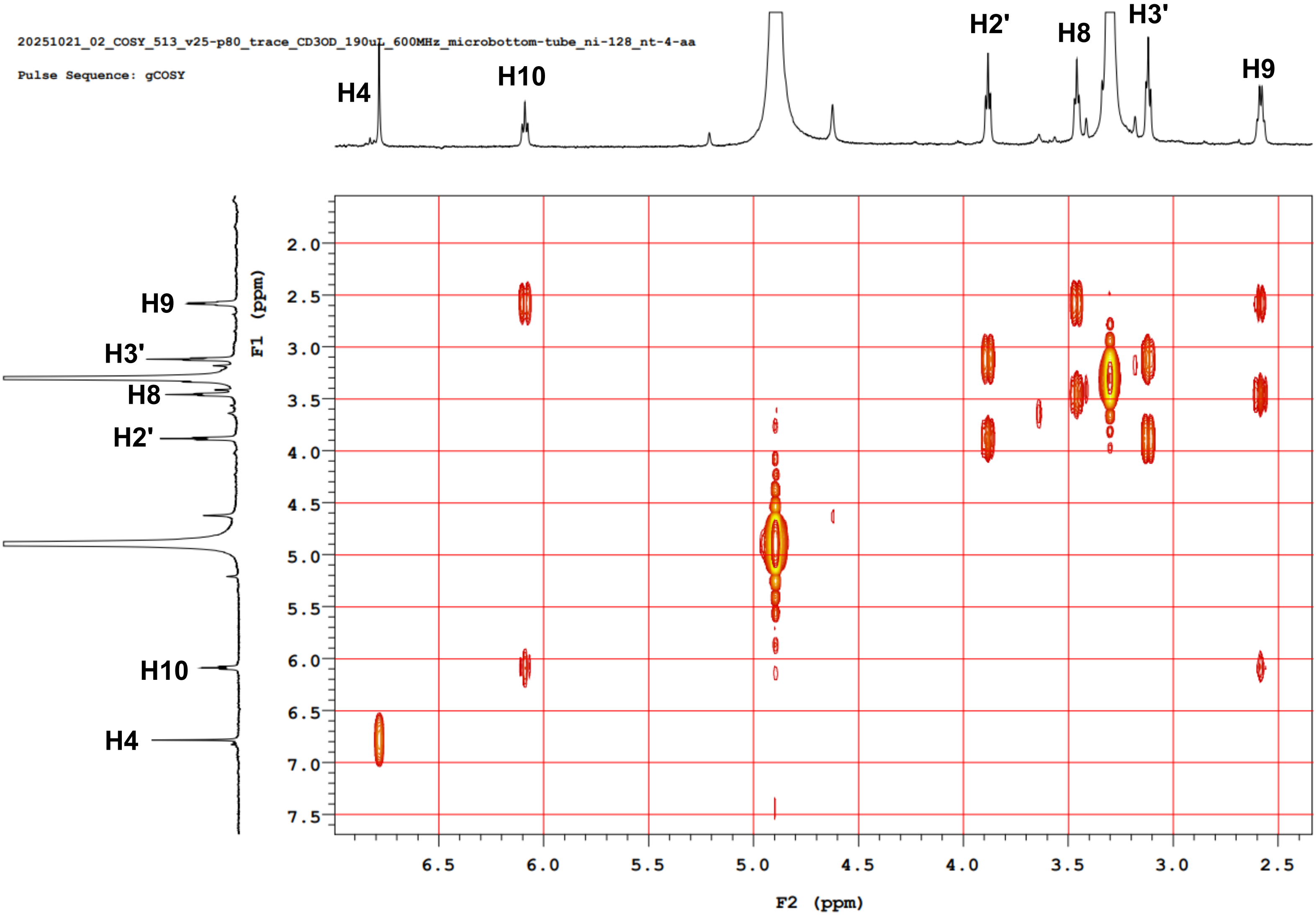

Figure S37. COSY spectrum of **11** (0.30 mg) (600 MHz, CD_3OD: 190 μL).

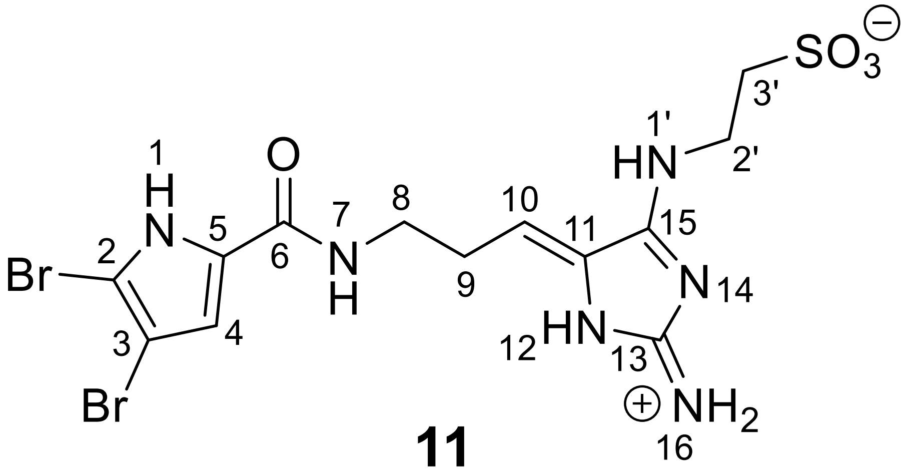

11

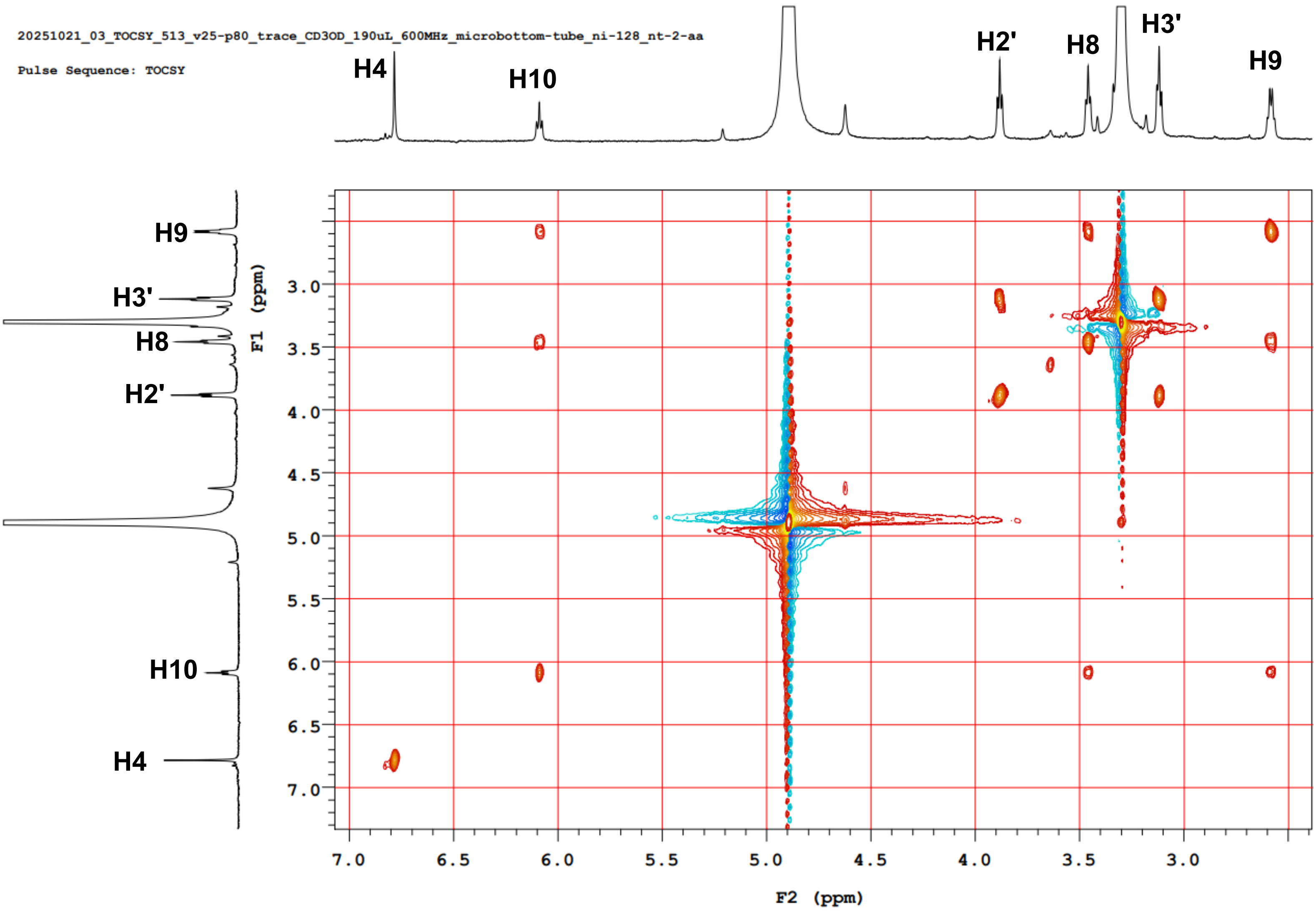

Figure S38. TOCSY spectrum of **11** (0.30 mg) (600 MHz, CD_3OD: 190 μL).

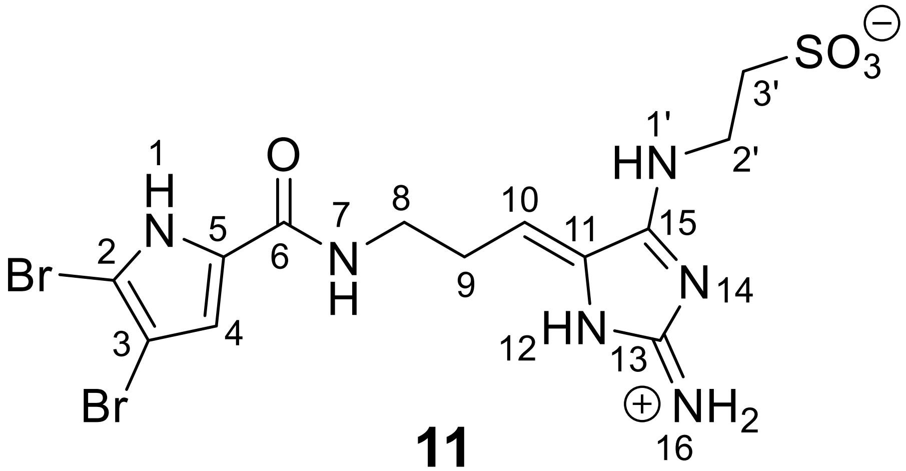

11

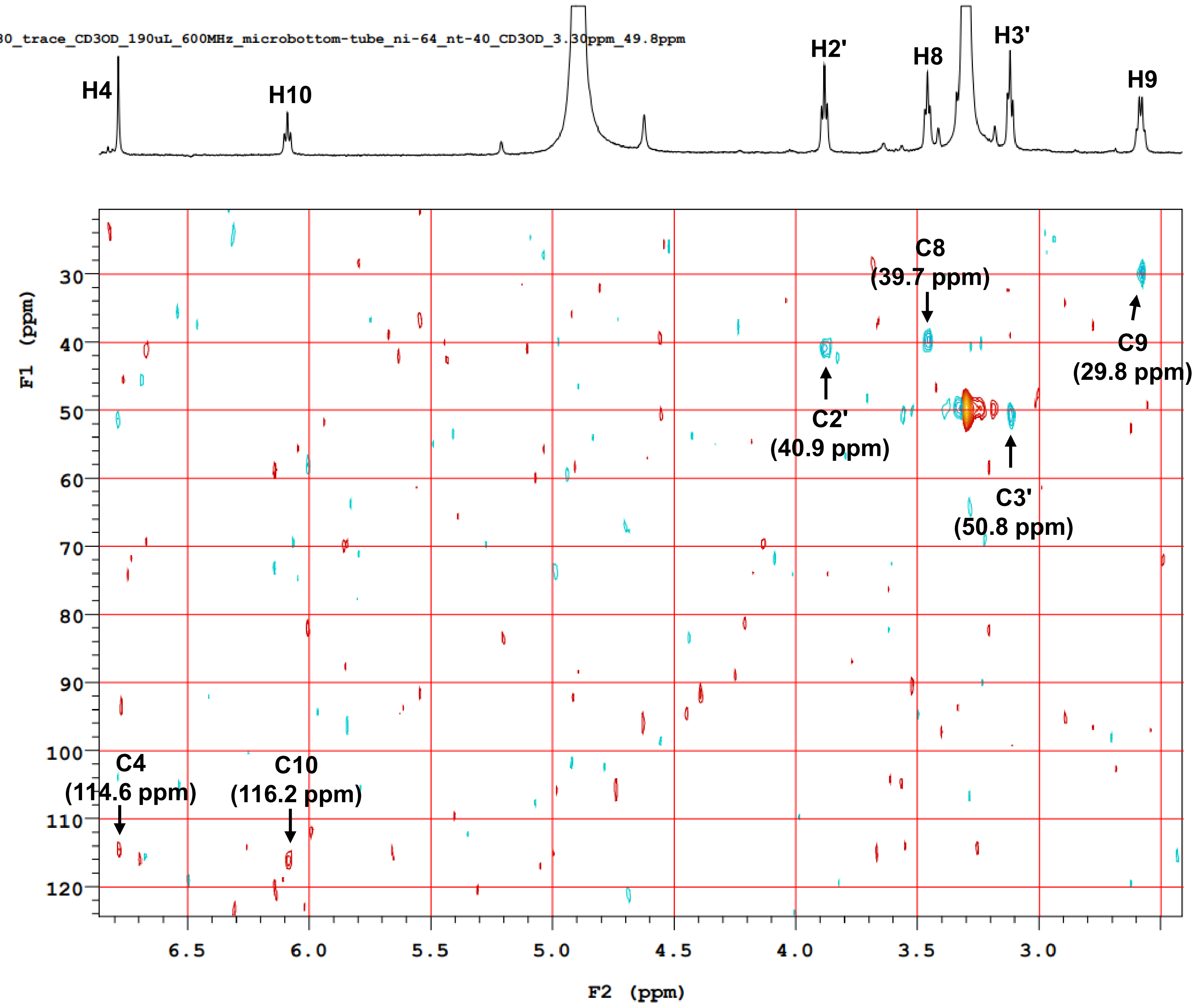

Figure S39. ^{1}H-^{13}C HSQC spectrum of **11** (0.30 mg) (600 MHz /151 MHz, CD_3OD: 190 µL).

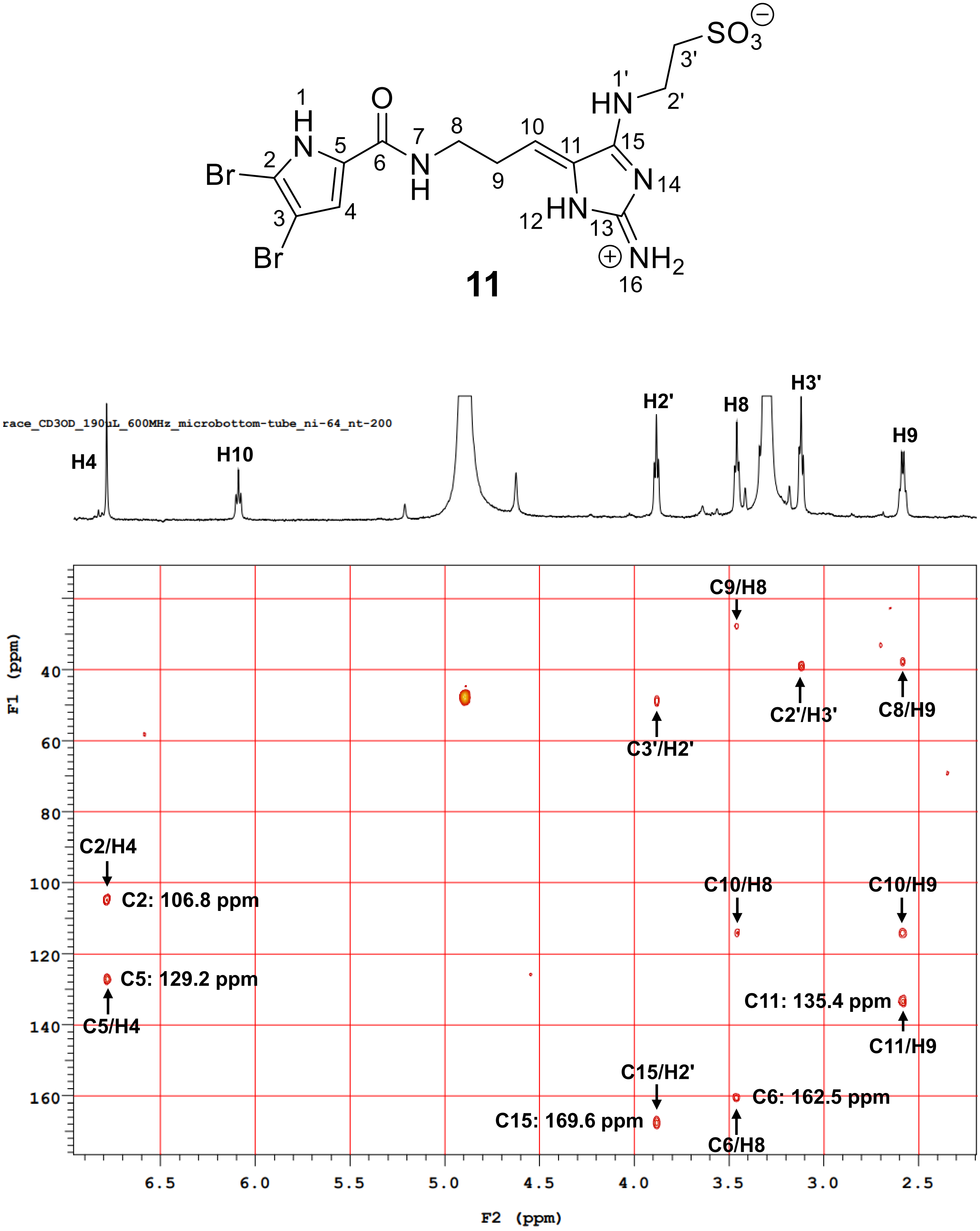

Figure S40. ^{1}H-^{13}C HMBC spectrum of **11** (0.30 mg) (600 MHz /151 MHz, CD_3OD: 190 μL).

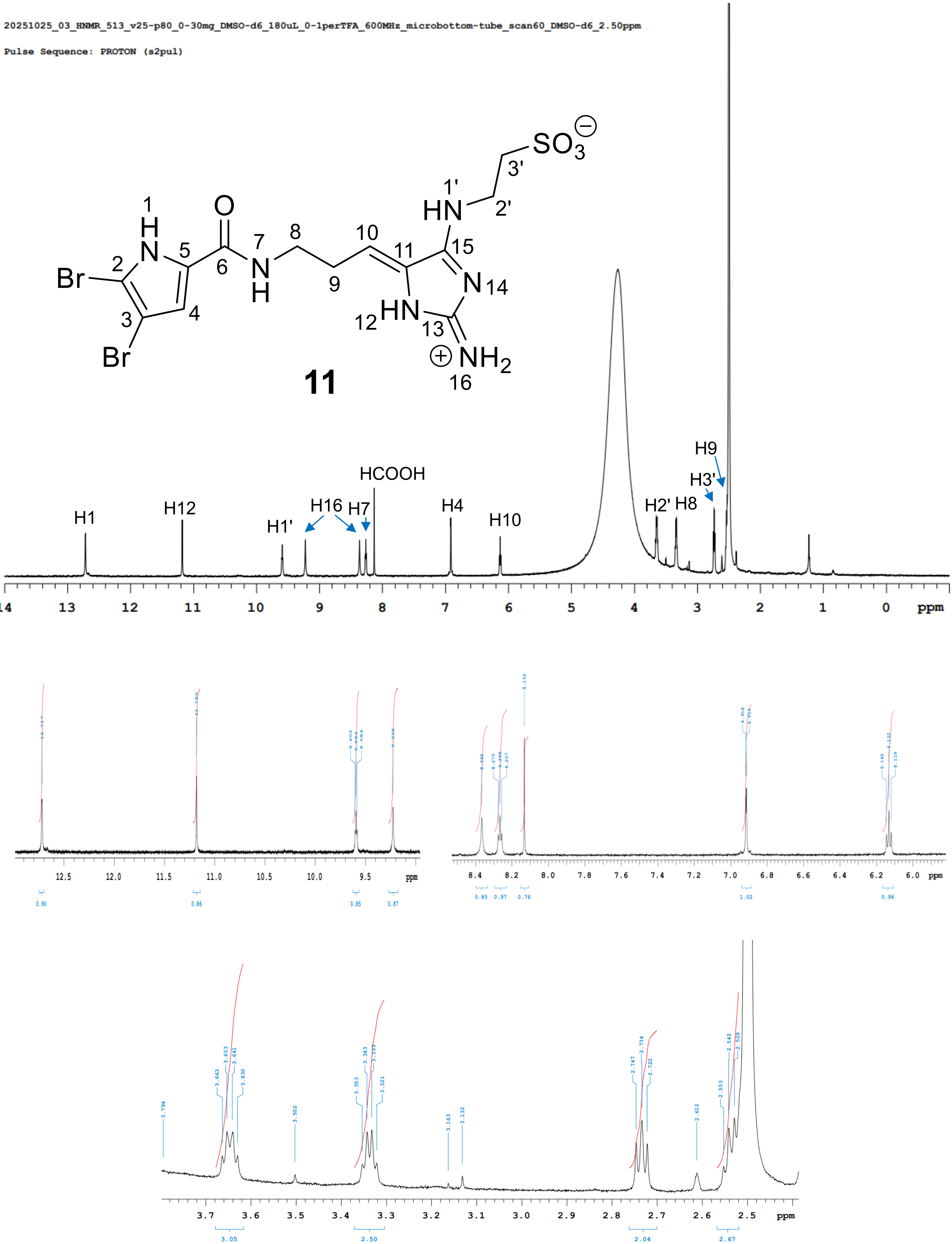

Figure S41. ^{1}H NMR spectrum of **11** (0.30 mg) (600 MHz, DMSO-d_6: 180 µL - 0.1% TFA).

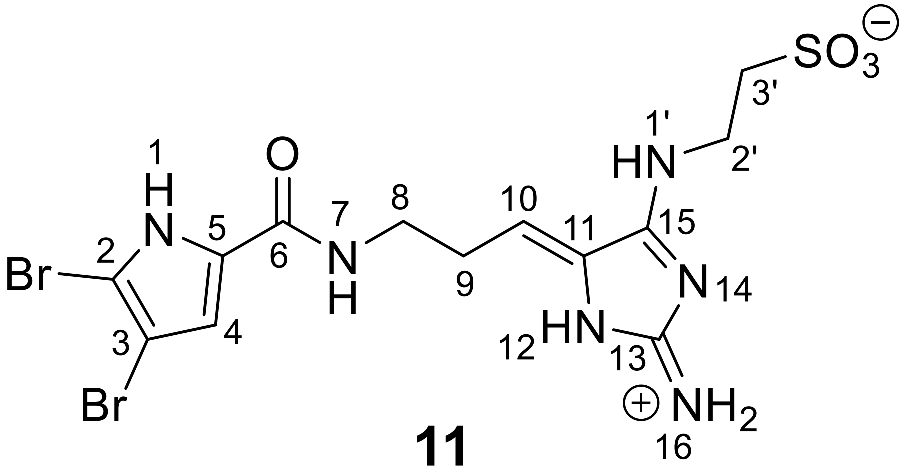

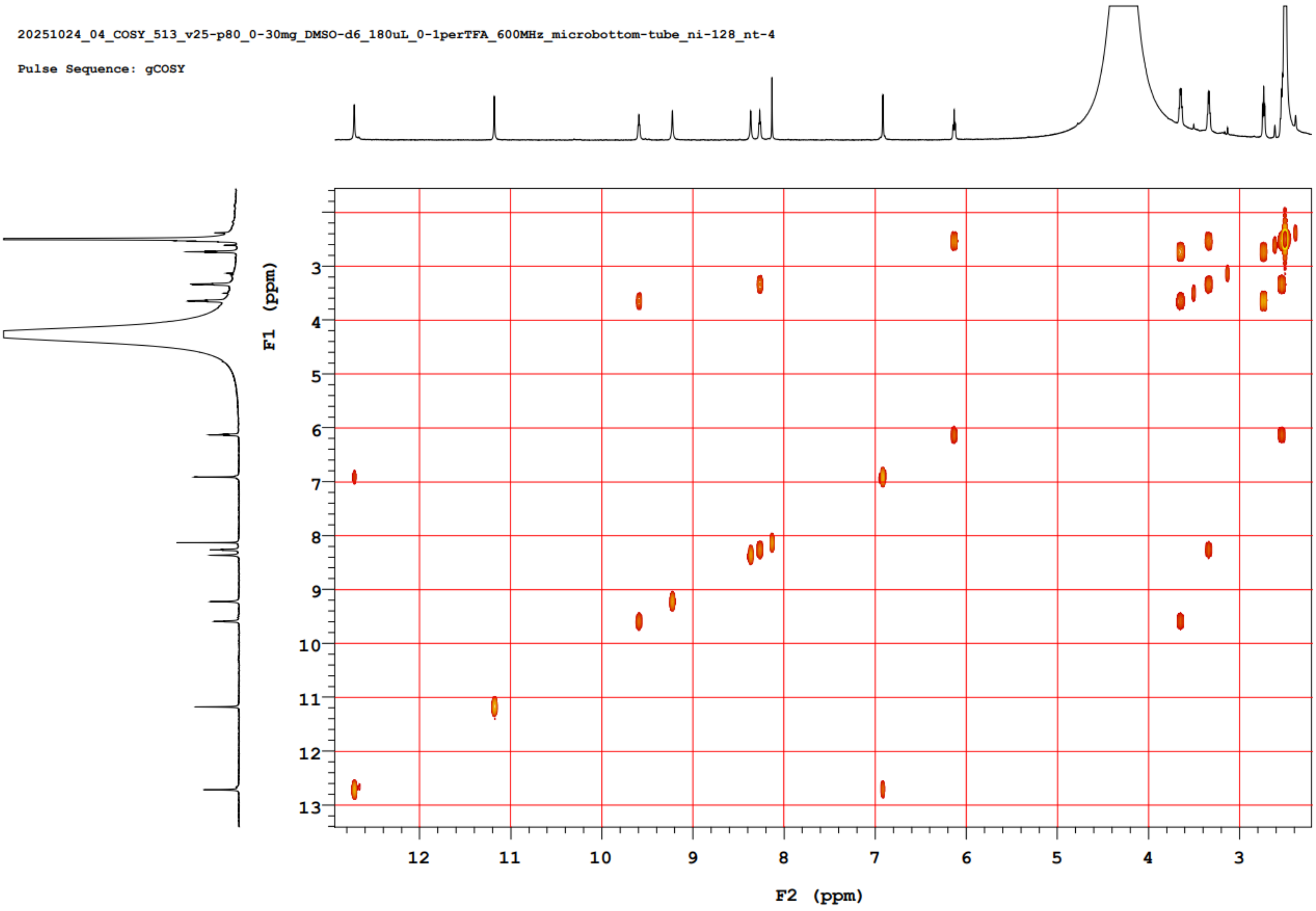

Figure S42. COSY spectrum of **11** (0.30 mg) (600 MHz, DMSO-d_6: 180 µL - 0.1% TFA).

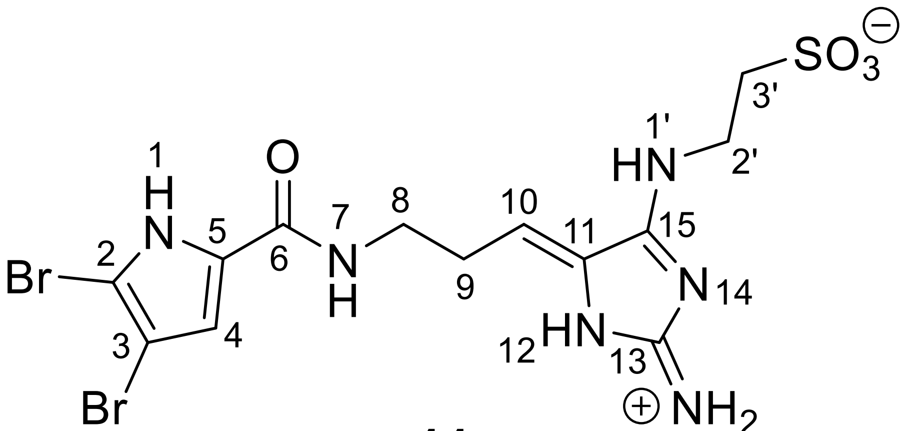

11

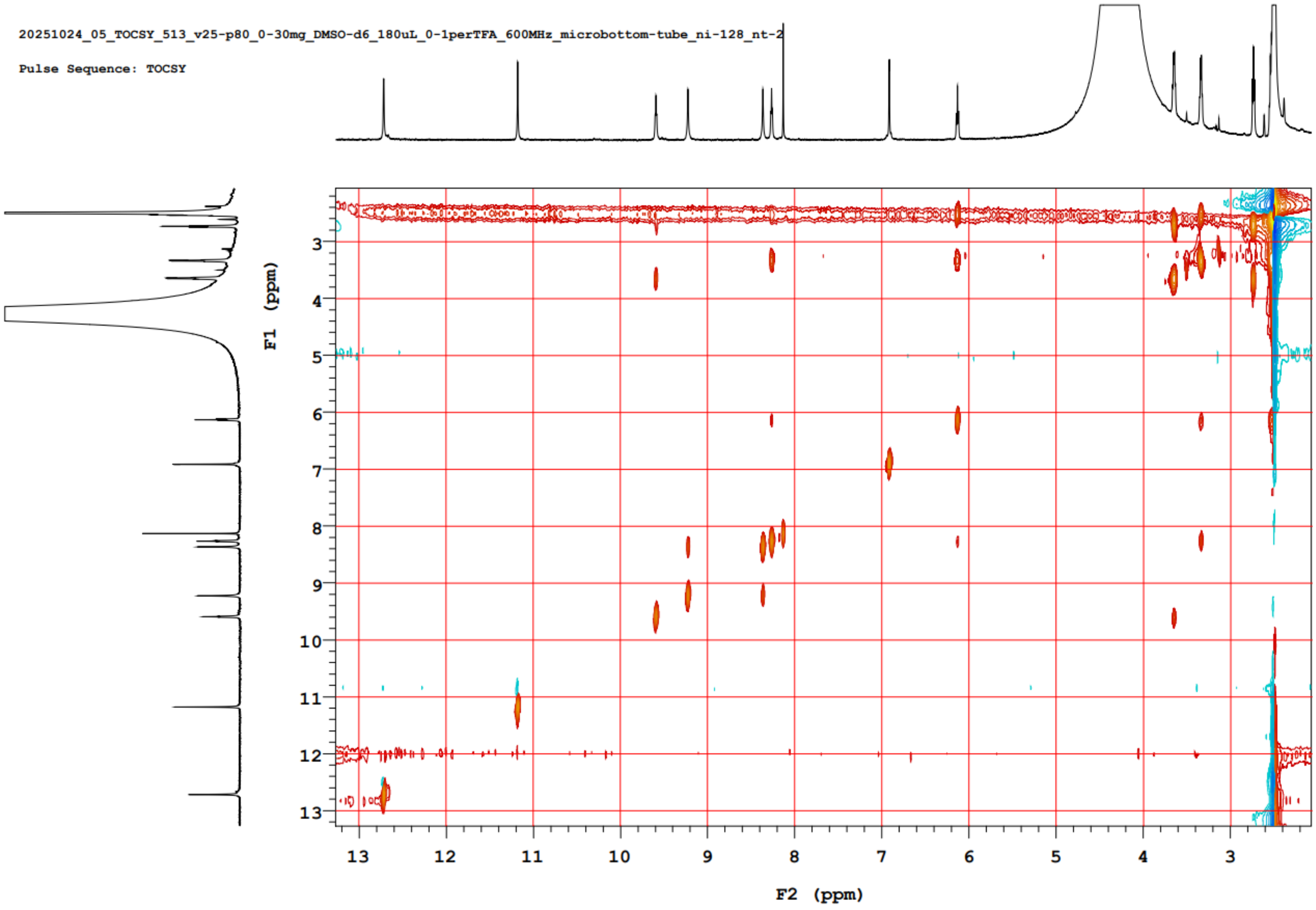

Figure S43. TOCSY spectrum of **11** (0.30 mg) (600 MHz, DMSO-d_6: 180 µL - 0.1% TFA).

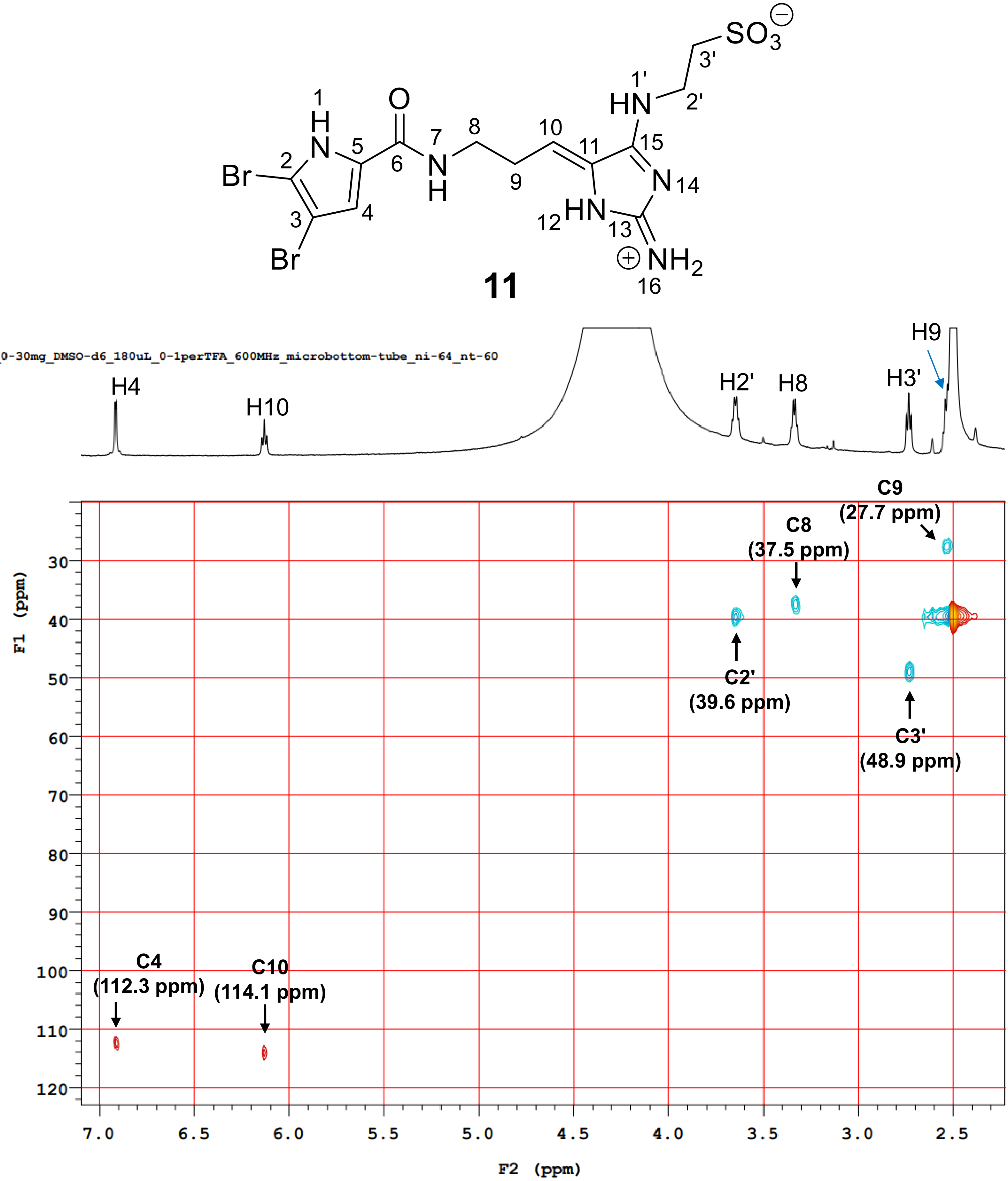

Figure S44. ^{1}H-^{13}C HSQC spectrum of **11** (0.30 mg) (600 MHz / 151 MHz, DMSO-d_6: 180 µL - 0.1% TFA).

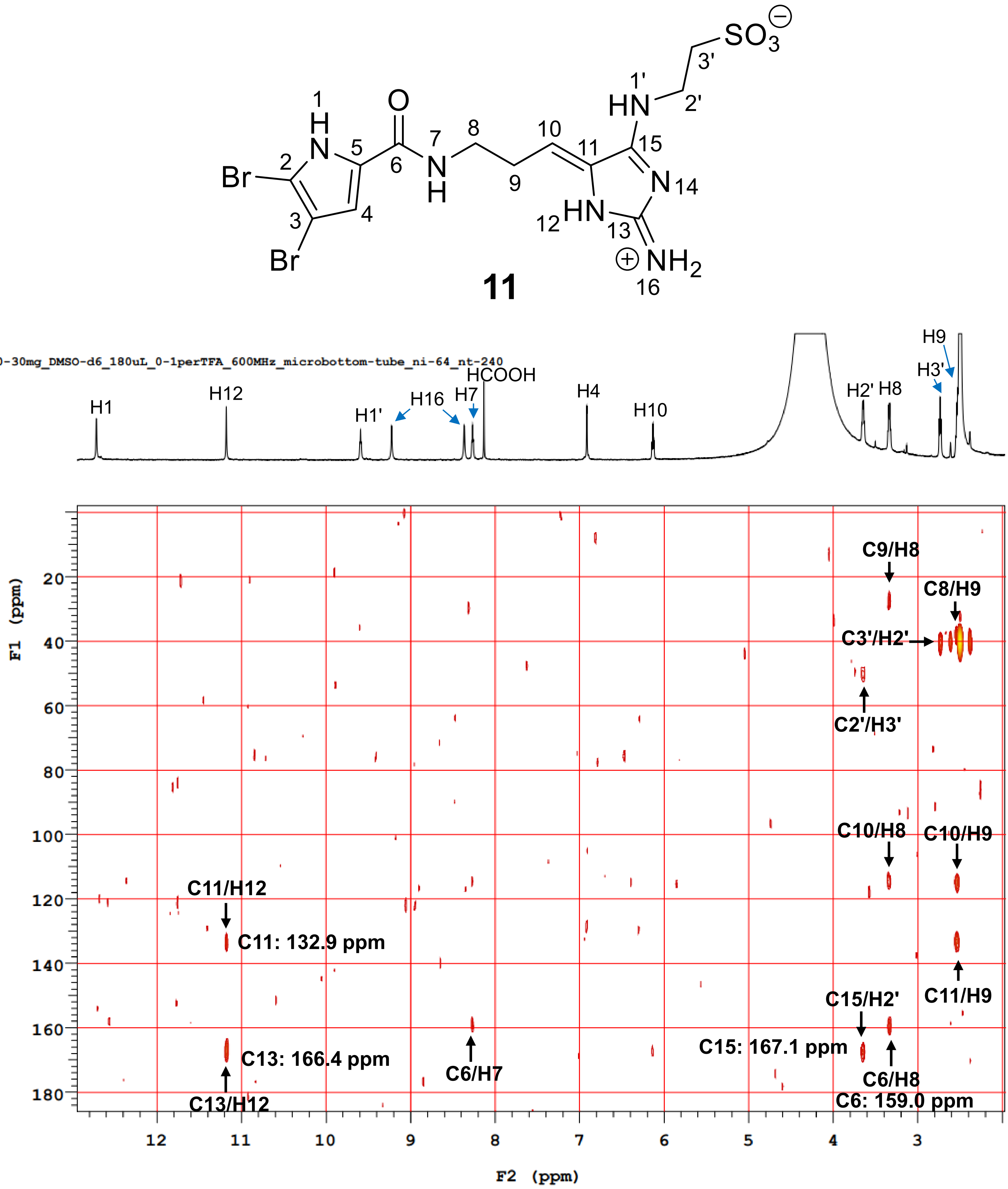

Figure S45. ^1H-^{13}C HMBC spectrum of **11** (0.30 mg) (600 MHz / 151 MHz, DMSO-d_6: 180 µL - 0.1% TFA).

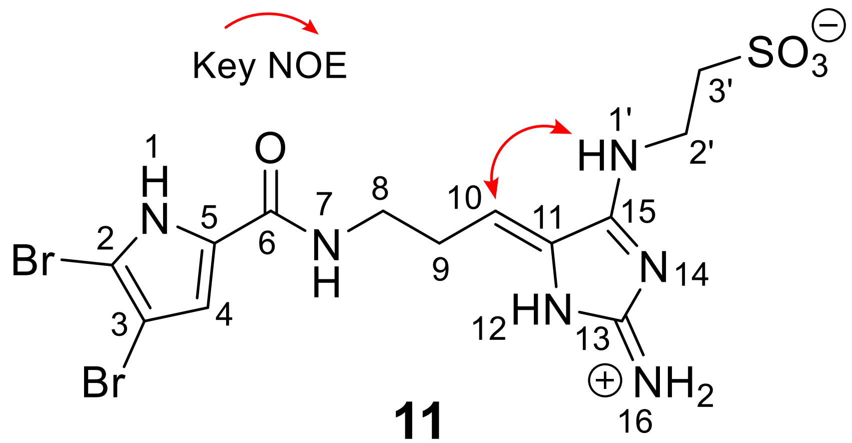

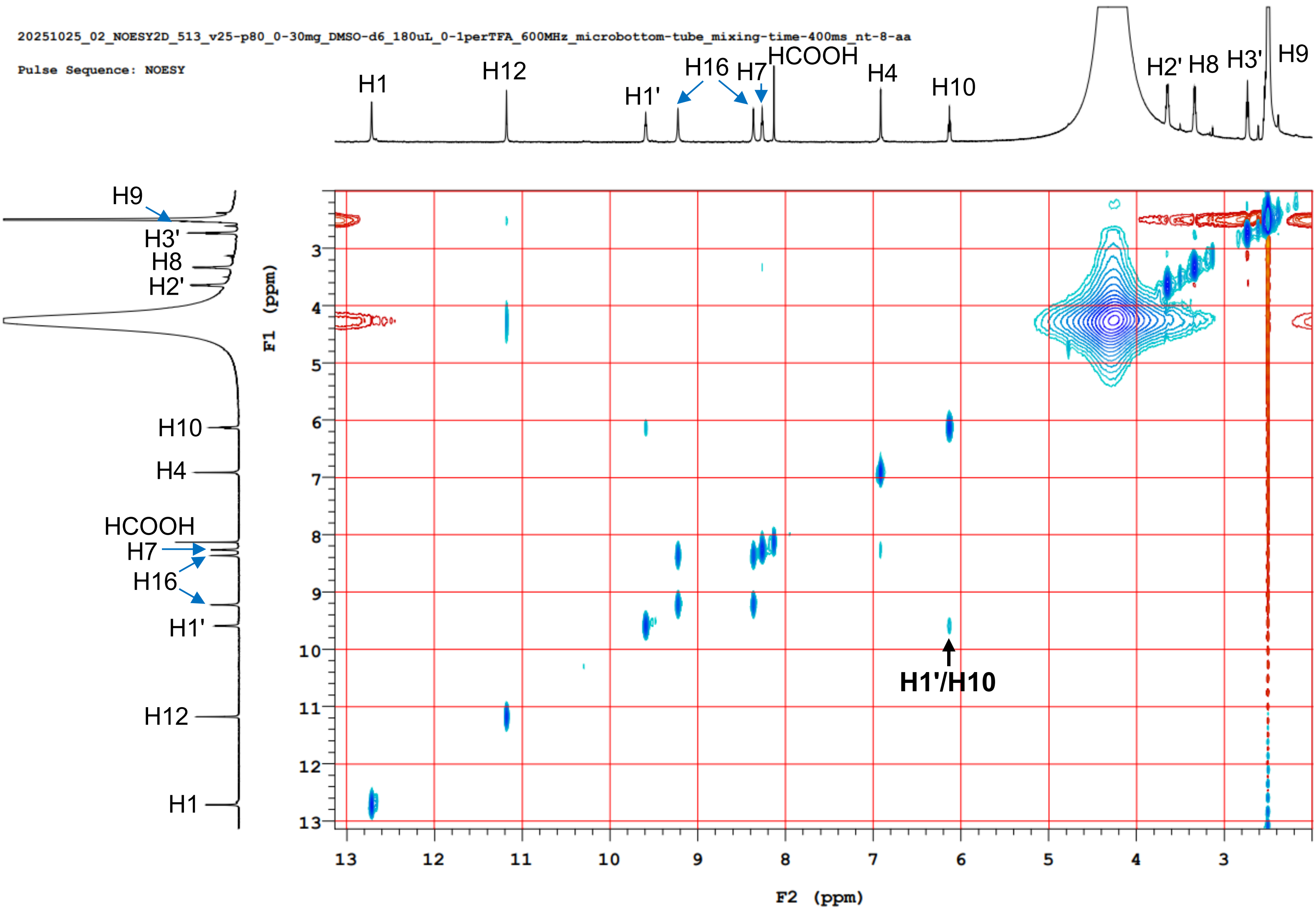

Figure S46. NOESY spectrum of **11** (0.30 mg) (600 MHz, DMSO-d_6: 180 µL - 0.1% TFA).

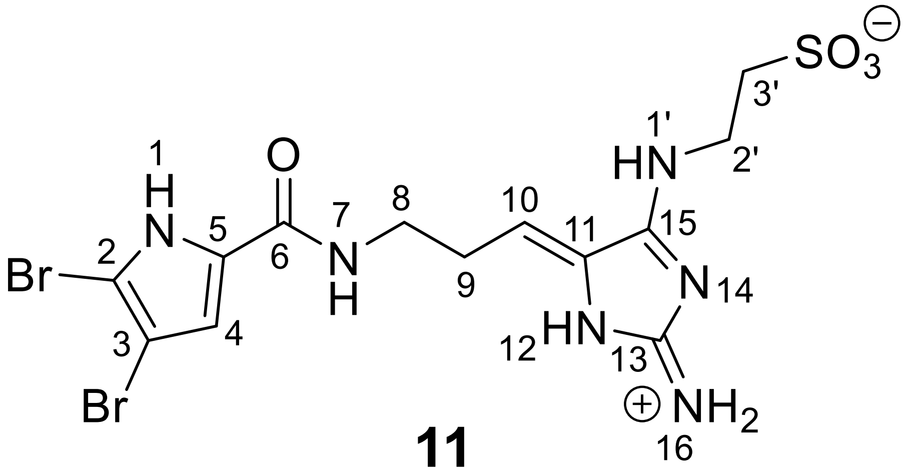

11

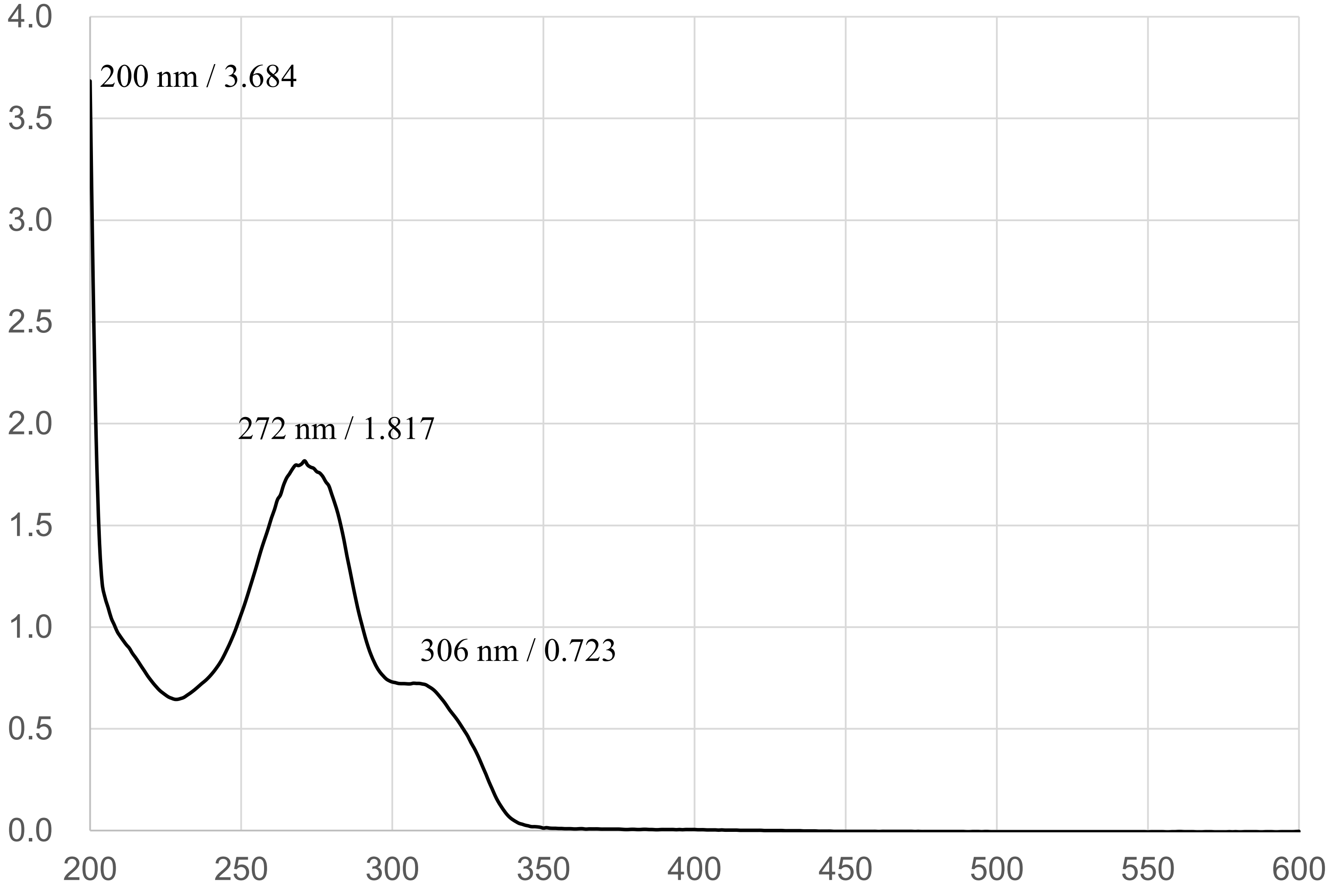

Figure S47. UV absorption spectrum of **11** (MeOH). c = 1.35 x 10^{-4} (M)

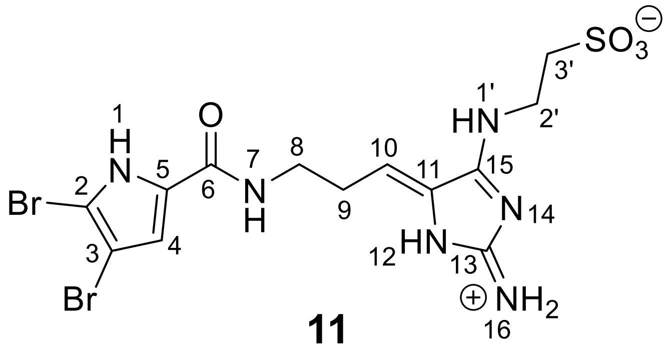

11

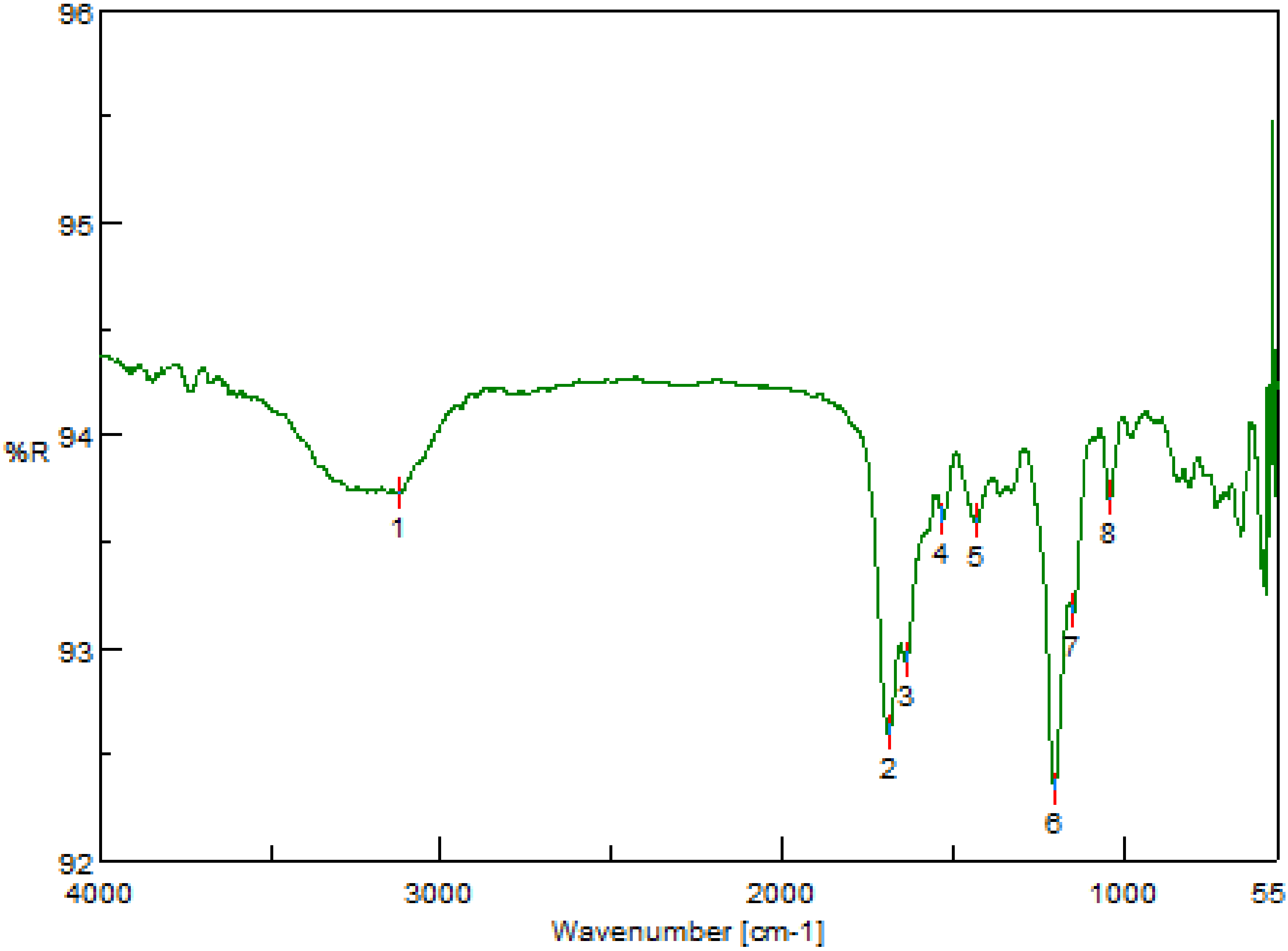

No.	Wavenumber	Strength
1	3121.2	93.7244
2	1689.3	92.5977
3	1636.3	92.9388
4	1530.2	93.6011
5	1428.0	93.5965
6	1202.4	92.3351
7	1146.5	93.1751
8	1037.5	93.7072

Figure S48. IR spectrum of **11** (ATR).

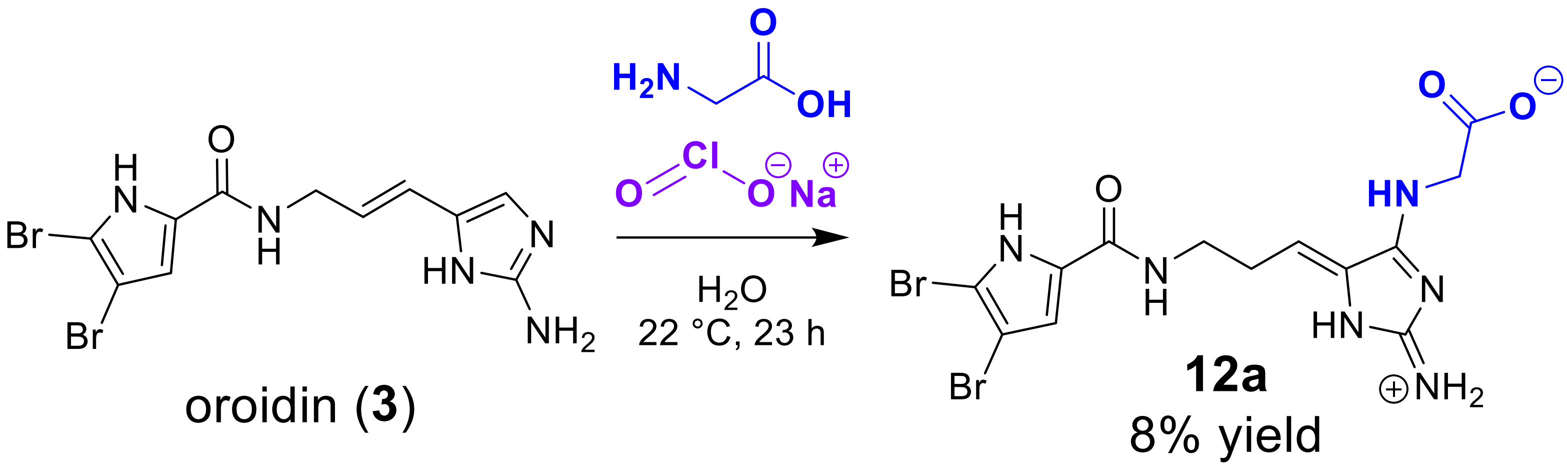

Scheme S1. Synthesis of **12a**.

Oroidin (**3**) (HCOOH salt, 3.0 mg, 0.0069 mmol) was placed in 20 mL round-bottomed flasks, and H_2O (4.0 mL) was added to the flask with stirring. Glycine (0.75 g, 10 mmol, 1449 equiv.) was then added to the mixtures, followed by addition of $NaClO_2$ (120 mg, 1.33 mmol, 193 equiv.). The flasks were sealed with septa caps, and the reaction mixtures were stirred at 22 °C for 23 h. After completion, the mixtures were combined and filtered through a small pad of Celite, rinsing the flasks and filter cake with H_2O. The filtrate was directly purified by ODS silica gel column chromatography ($MeOH/H_2O$, 0:100 to 100:0, v/v). The eluate was concentrated under reduced pressure, and the crude material was filtered through a Cosmospin filter H (0.45 µm). Further purification was performed by RP-HPLC (InertSustain AQ-C18, 5 µm, 10 mm i.d. × 250 mm; GL Science) using gradient elution (0–4 min, $MeOH/H_2O/HCOOH$ = 35:65:0.1 to 45:55:0.1, v/v; 4 min-, 45:55:0.1) at a flow rate of 2.0 mL/min. Pure **12a** was obtained at 27-33 min (0.25 mg, 0.00054 mmol, 8% yield) as an off-white to slightly brown film.

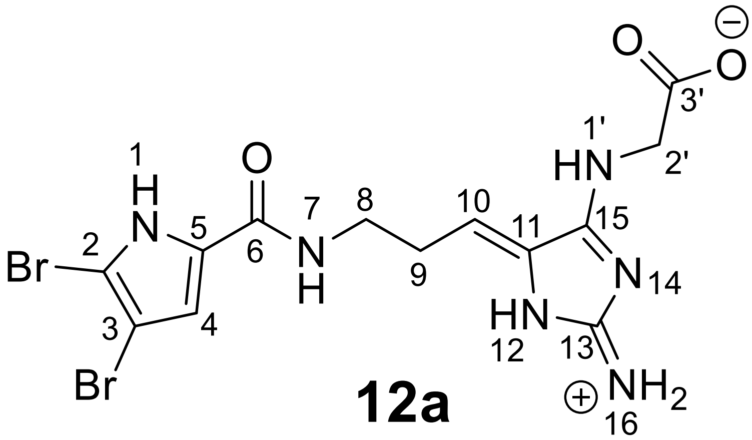

12a

12a:

$\mathbf{R_f}$ = 0.78 ($CHCl_3$/MeOH/28% NH_3 aq. = 50:50:2, v/v/v; UV).

UV/vis λ_{max} (MeOH) nm (log ε): 310 (3.61), 270 (4.05), 200 (4.28).

^{1}H NMR (600 MHz, CD_3OD containing 0.1% TFA): δ 6.78 (s, C4-H, 1H), 6.19 (t, J = 7.5 Hz, C10-H, 1H), 4.24 (s, C2'-H, 2H), 3.48 (t, J = 6.6 Hz, C8-H, 2H), 2.62 (t, J = 7.2 Hz, C9-H, 2H).

^{1}H NMR (600 MHz, DMSO-d_6 containing 0.1% TFA): δ 12.72 (s, N1-H, 1H), 11.38 (s, N12-H, 1H), 9.92 (t, J = 6.0 Hz, N1'-H, 1H), 9.27 (s, N16-H, 1H), 8.54 (s, N16-H, 1H), 8.28 (t, J = 5.4 Hz, N7-H, 1H), 6.92 (d, J = 3.0 Hz, C4-H, 1H), 6.30 (t, J = 7.5 Hz, C10-H, 1H), 4.13 (d, J = 6.0 Hz, C2'-H, 2H), 3.34 (m, C8-H, 2H), 2.57 (q, J = 7.2 Hz, C9-H, 2H).

^{13}C NMR (151 MHz, CD_3OD containing 0.1% TFA): δ 172.3 (C3'), 171.0 (C15), 168.8 (C13), 162.8 (C6), 135.6 (C11), 129.4 (C5), 117.4 (C10), 115.1 (C4), 107.2 (C2), 100.8 (C3), 45.7 (C2'), 39.9 (C8), 30.2 (C9).

HRMS (**ESI**): (*m/z*) calcd for $C_{13}H_{15}{}^{79}Br_2N_6O_3^+$ $[M+H]^+$: 460.9567, found 460.9561.

IR ν_{max}: 3152 (br), 1689 (s), 1634 (s), 1530 (w), 1427 (w), 1205 (s), 1143 (m).

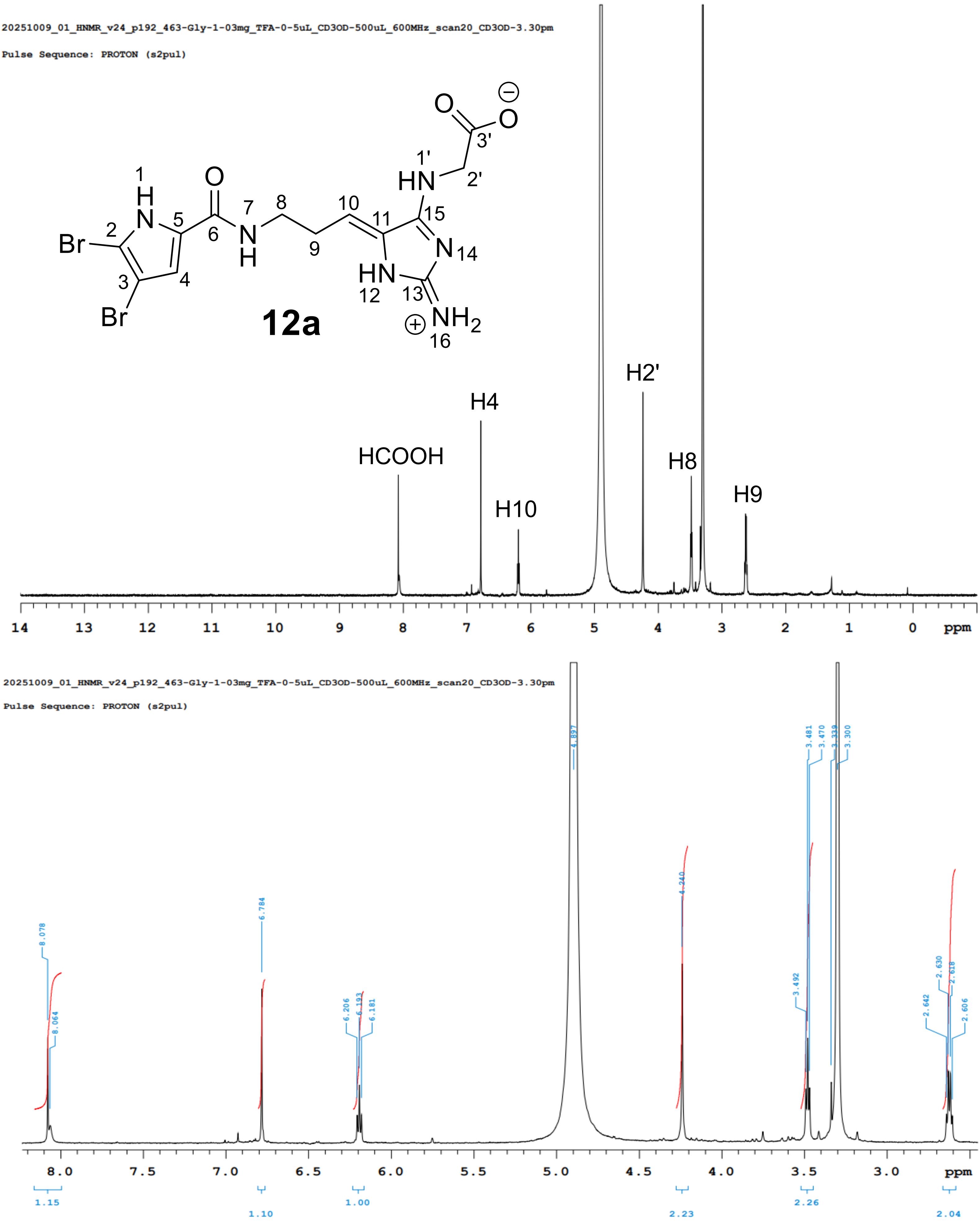

Figure S49. ^{1}H NMR spectrum of **12a** (1.03 mg) (600 MHz, CD_3OD: 500 μL - 0.1% TFA).

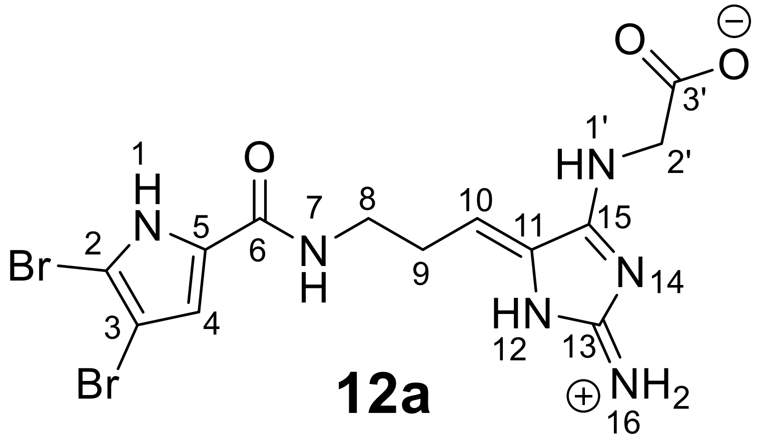

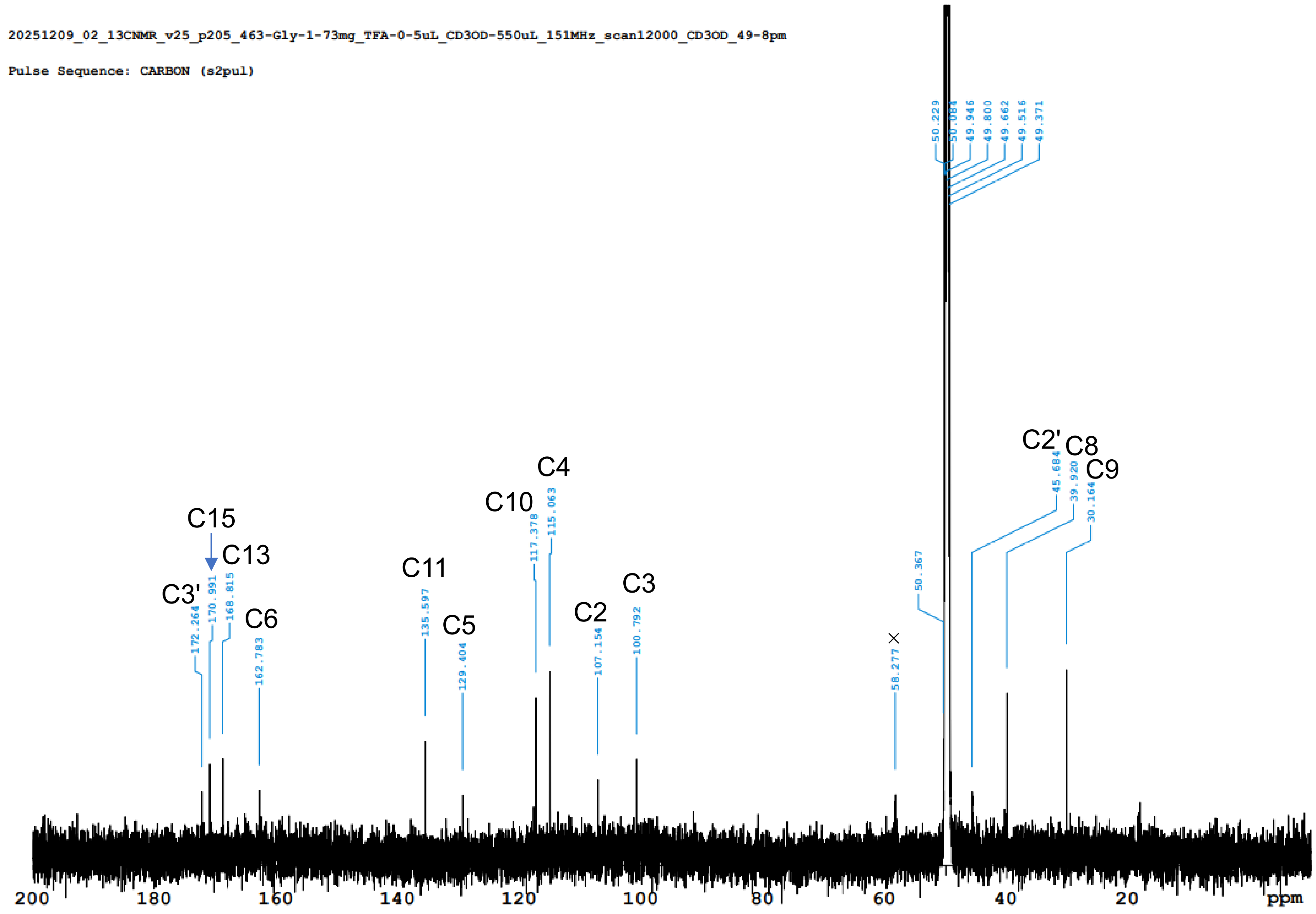

Figure S50. ^{13}C NMR spectrum of **12a** (1.73 mg) (151 MHz, CD_3OD: 550 μL - 0.1% TFA).

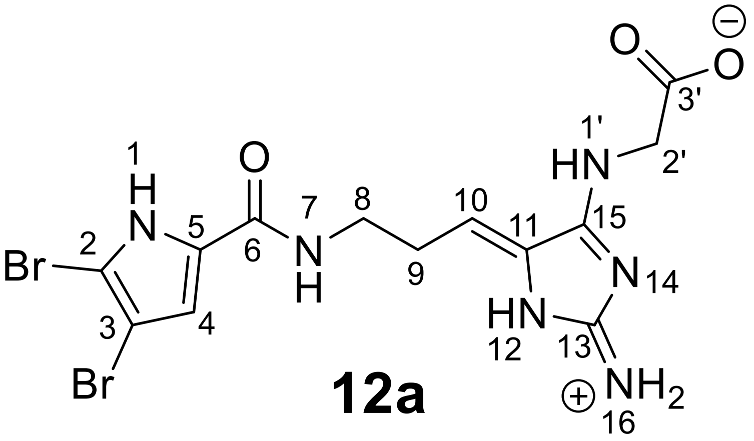

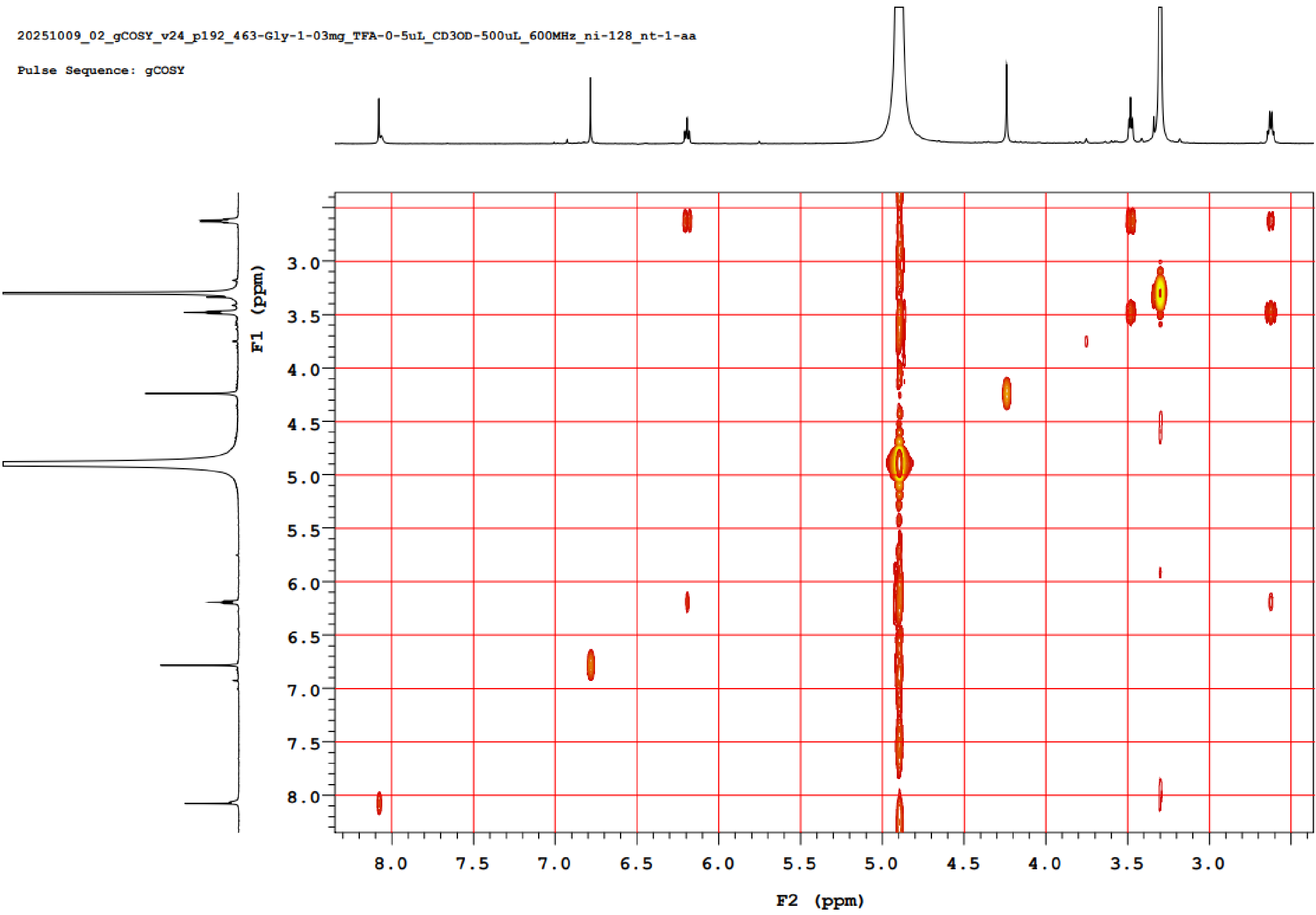

Figure S51. COSY spectrum of **12a** (1.03 mg) (600 MHz, CD_3OD: 500 µL - 0.1% TFA).

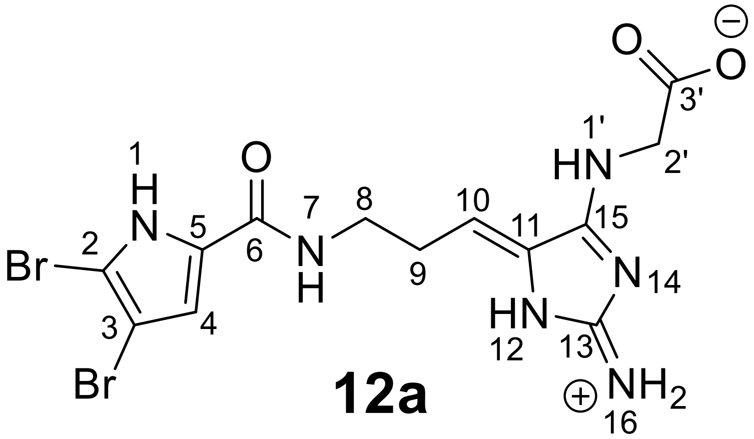

12a

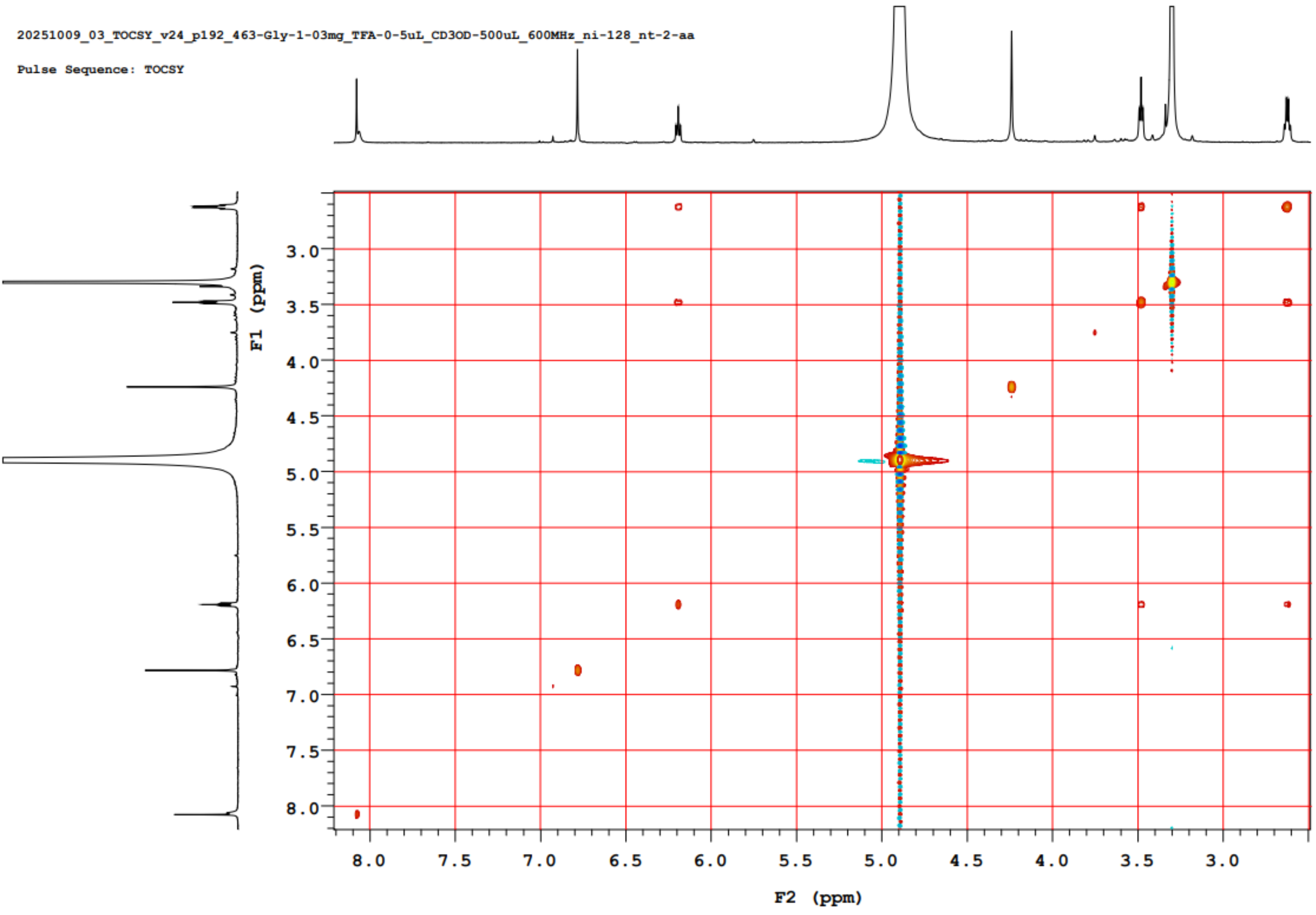

Figure S52. TOCSY spectrum of **12a** (1.03 mg) (600 MHz, CD_3OD: 500 µL - 0.1% TFA).

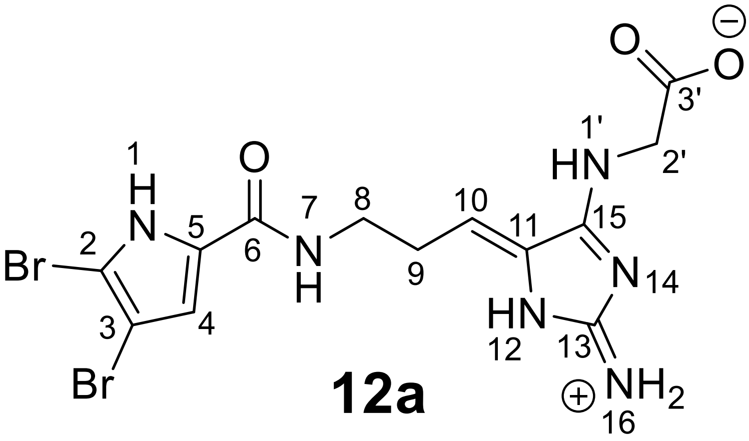

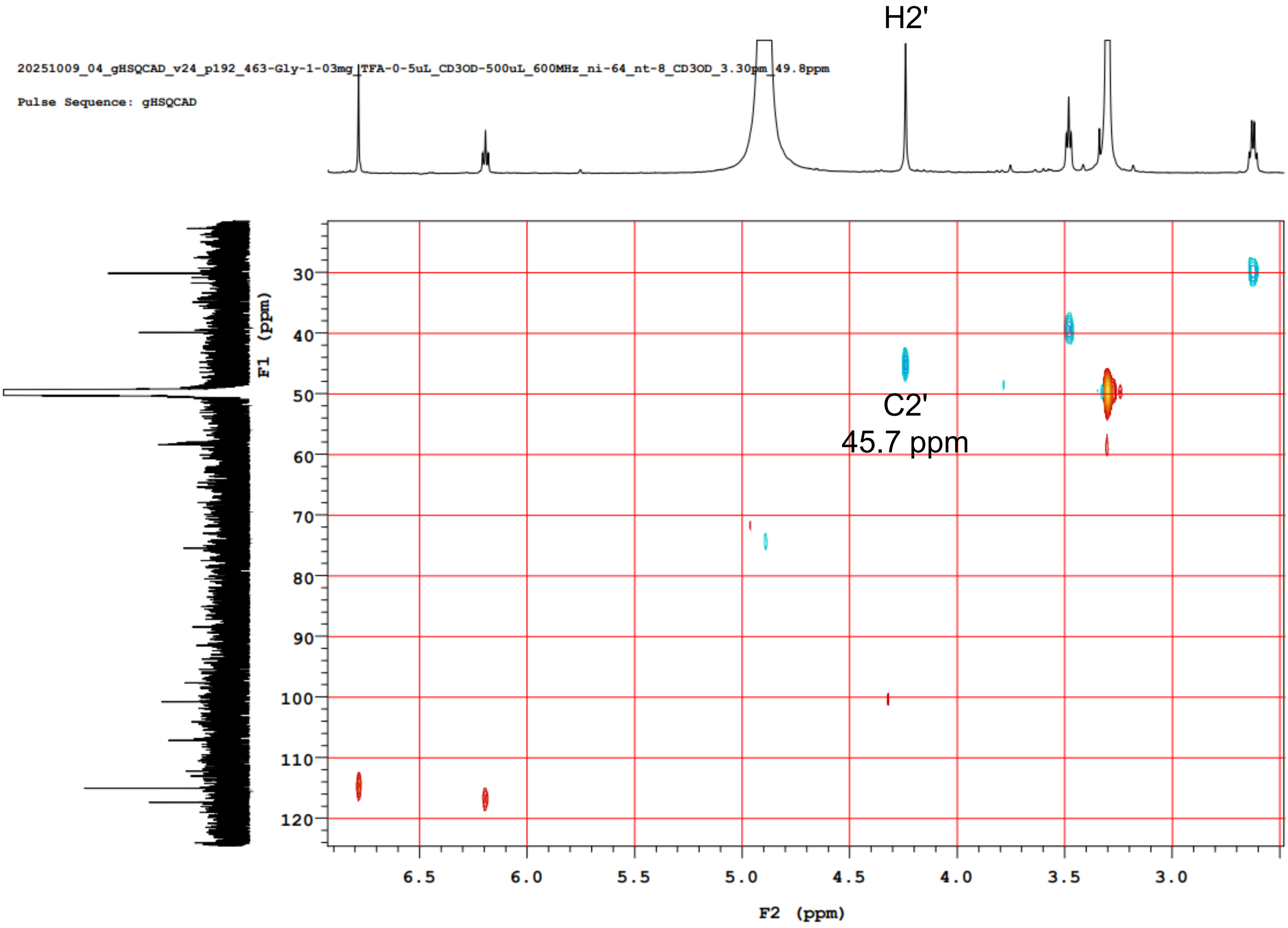

Figure S53. ^{1}H-^{13}C HSQC spectrum of **12a** (1.03 mg) (600 MHz/151 MHz, CD_3OD: 500 µL - 0.1% TFA).

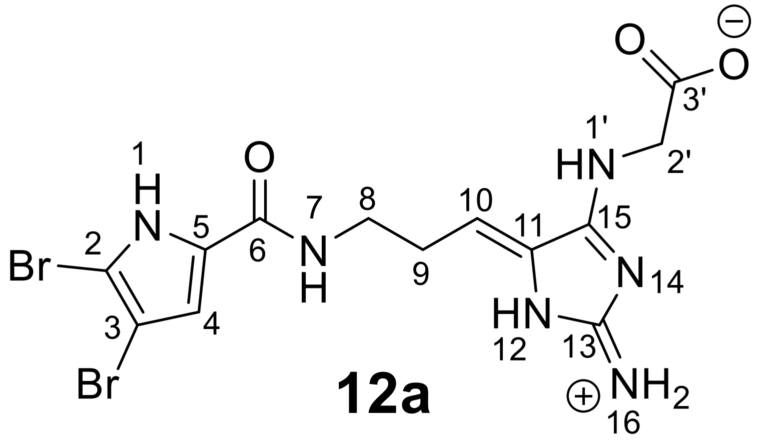

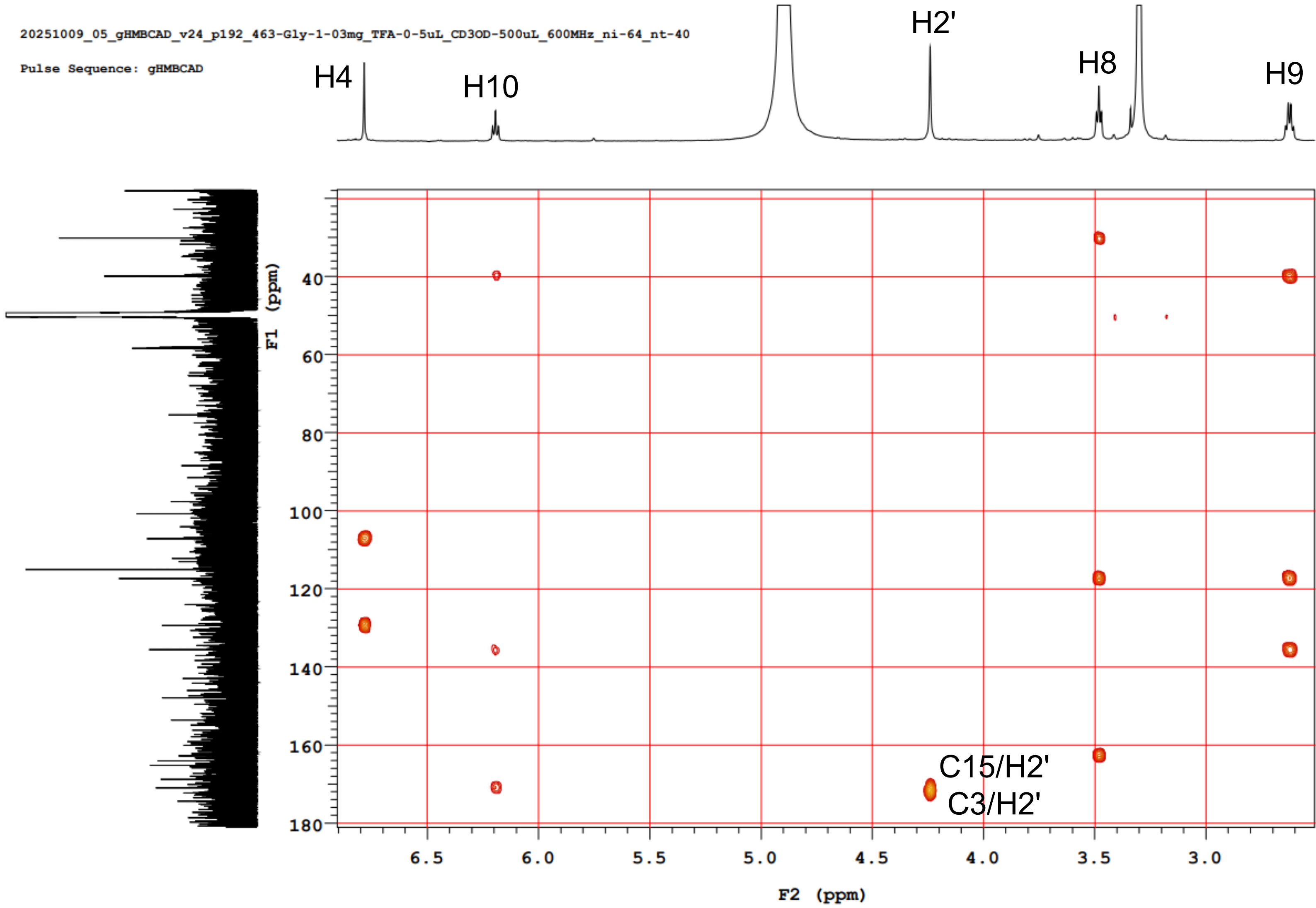

Figure S54. ^{1}H-^{13}C HMBC spectrum of **12a** (1.03 mg) (600 MHz/151 MHz, CD_3OD: 500 µL - 0.1% TFA).

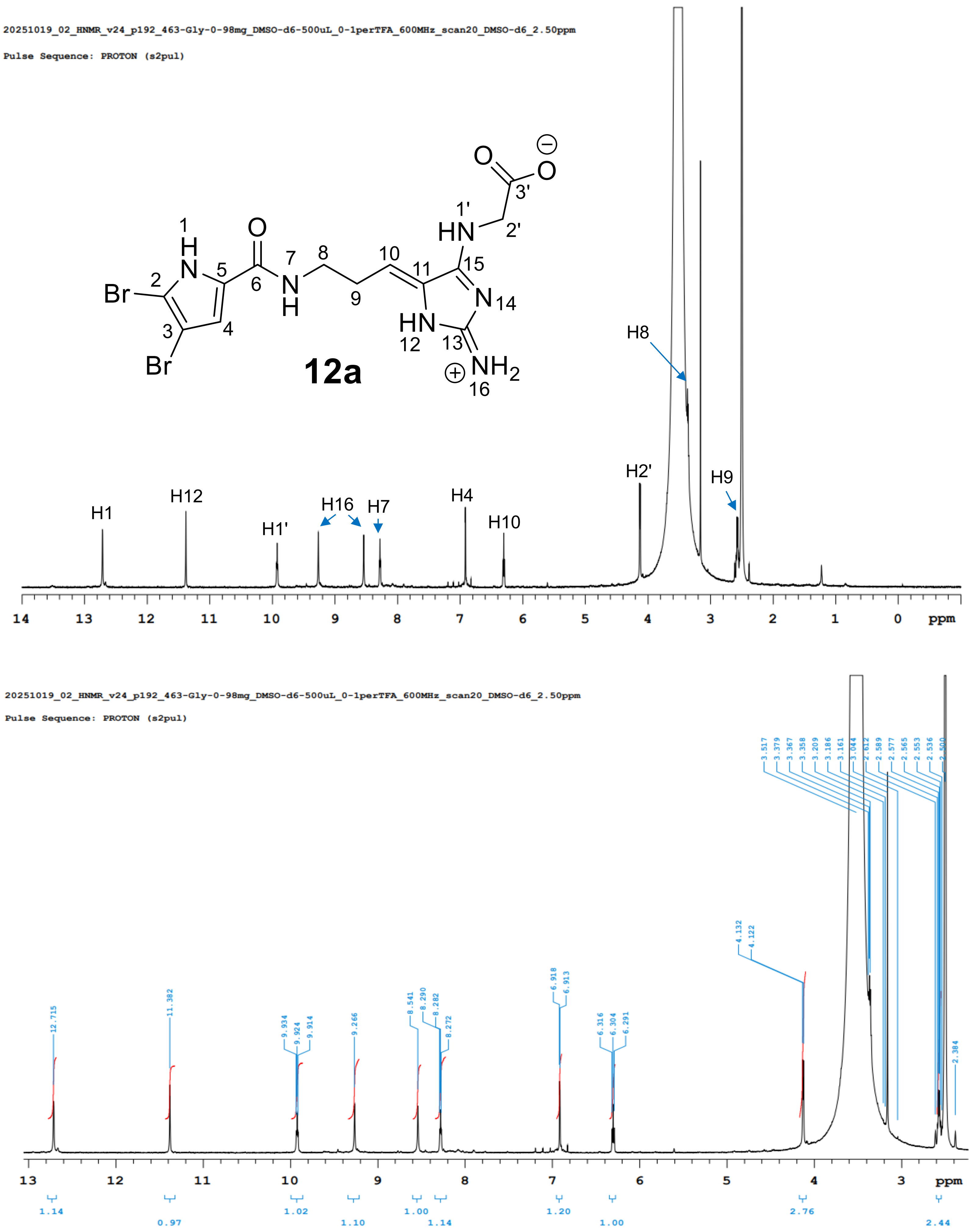

Figure S55. ^{1}H NMR spectrum of **12a** (0.98 mg) (600 MHz, DMSO-d_6: 500 µL - 0.1% TFA).

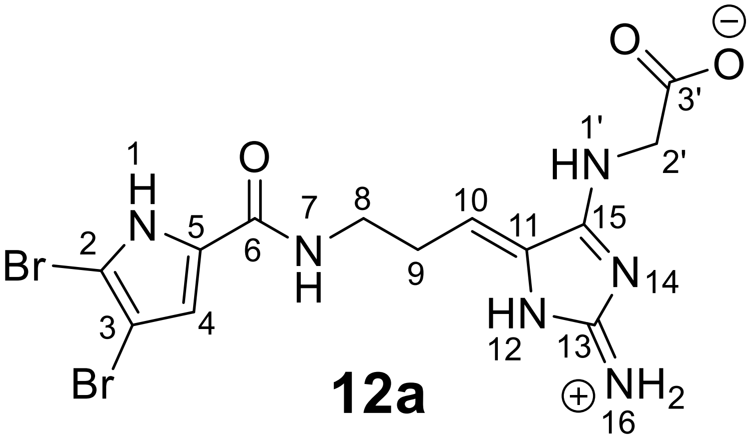

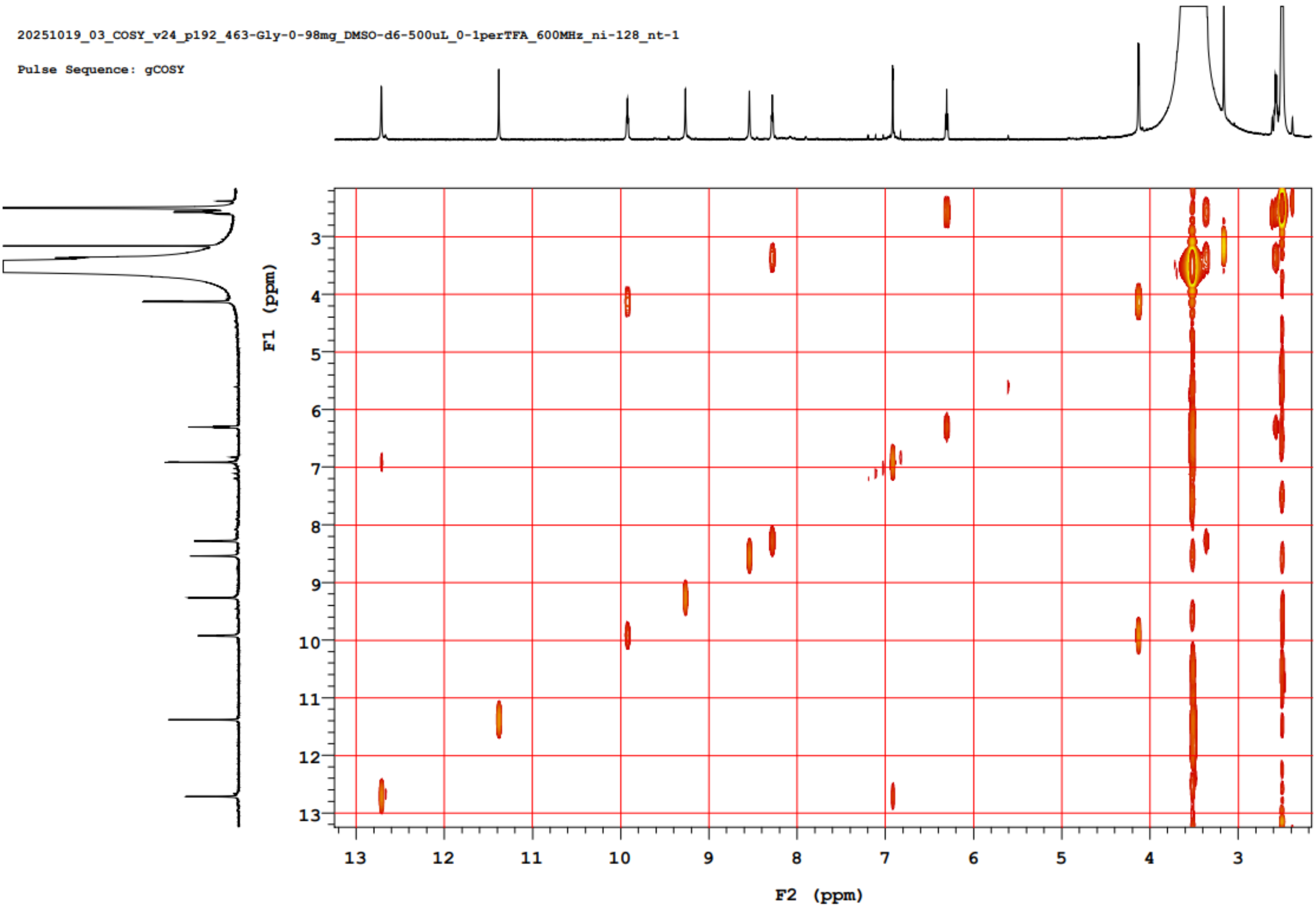

Figure S56. COSY spectrum of **12a** (0.98 mg) (600 MHz, DMSO-d_6: 500 µL - 0.1% TFA).

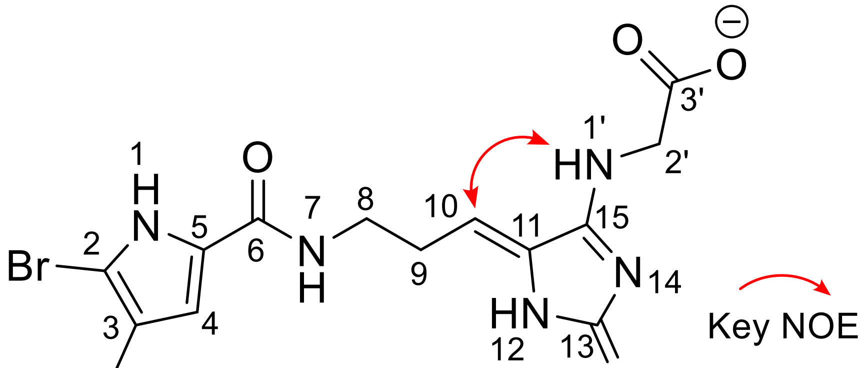

12a

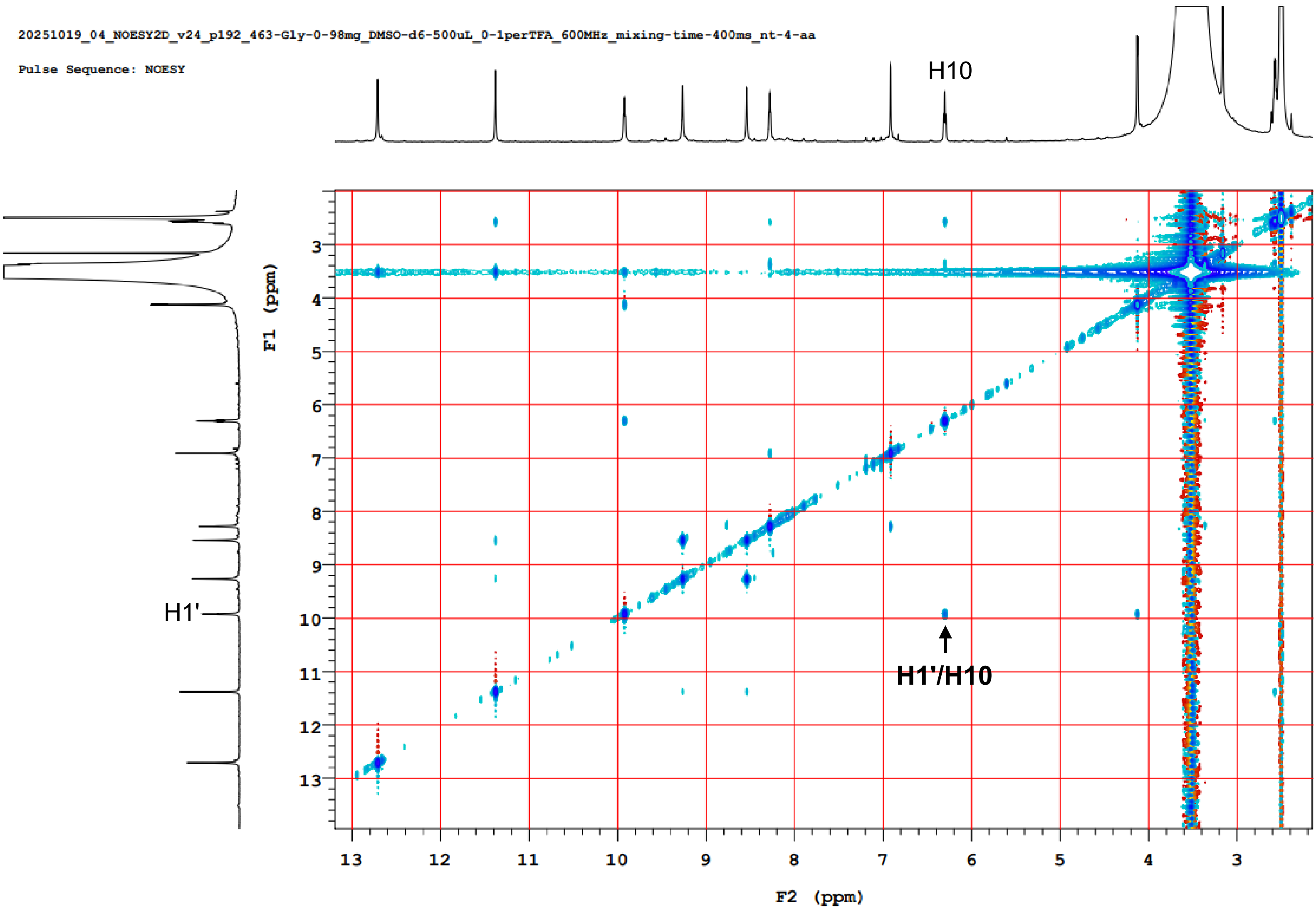

Figure S57. NOESY spectrum of **12a** (0.98 mg) (600 MHz, DMSO-d_6: 500 µL - 0.1% TFA).

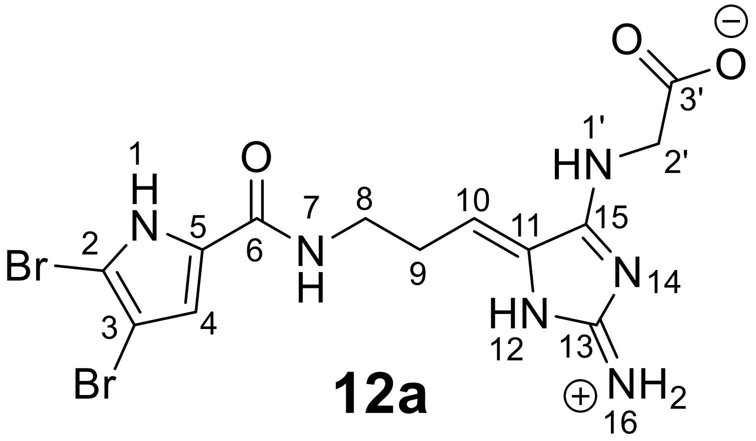

12a

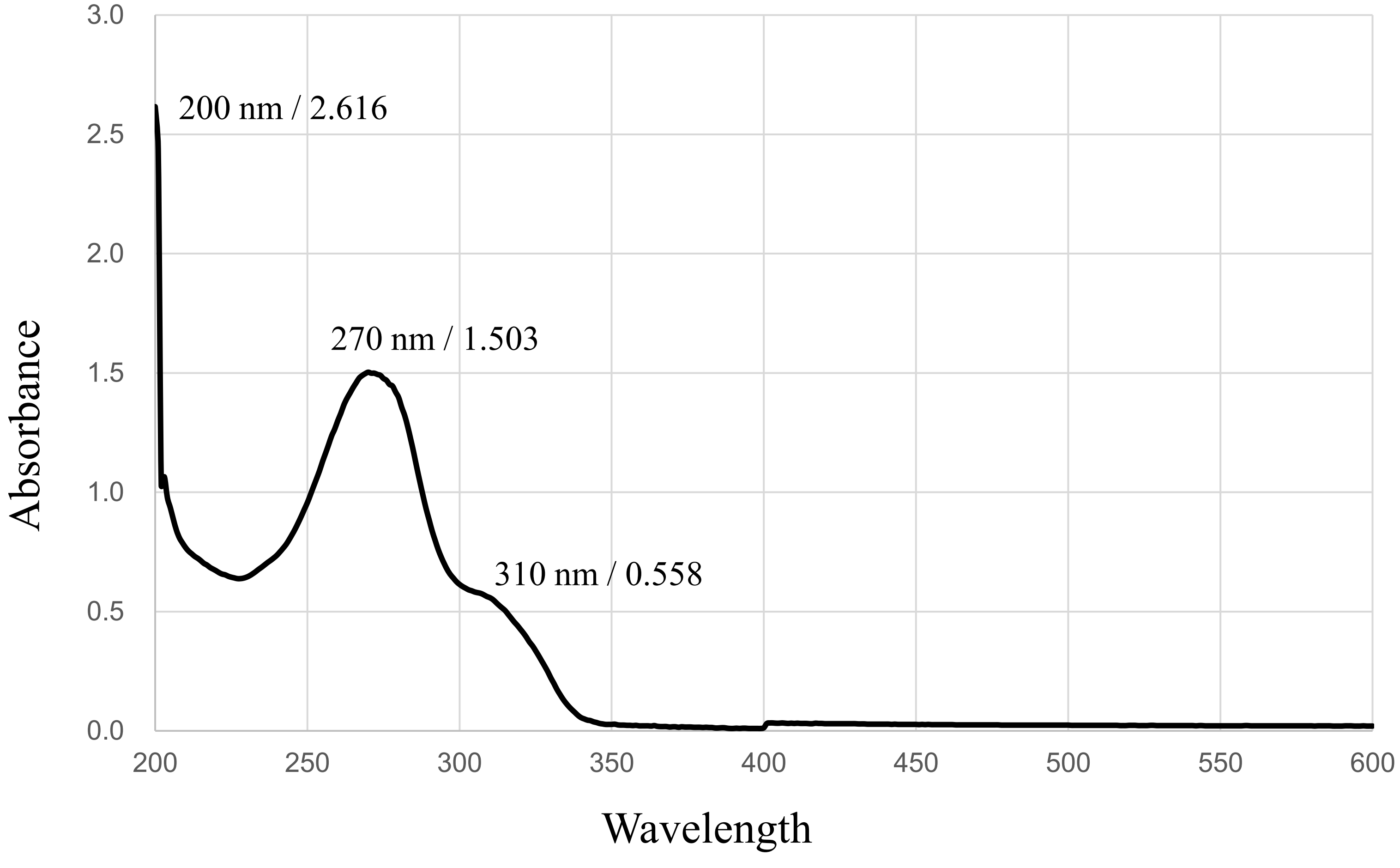

Figure S58. UV absorption spectrum of **12a** (MeOH). c = 1.36 x 10^{-4} (M)

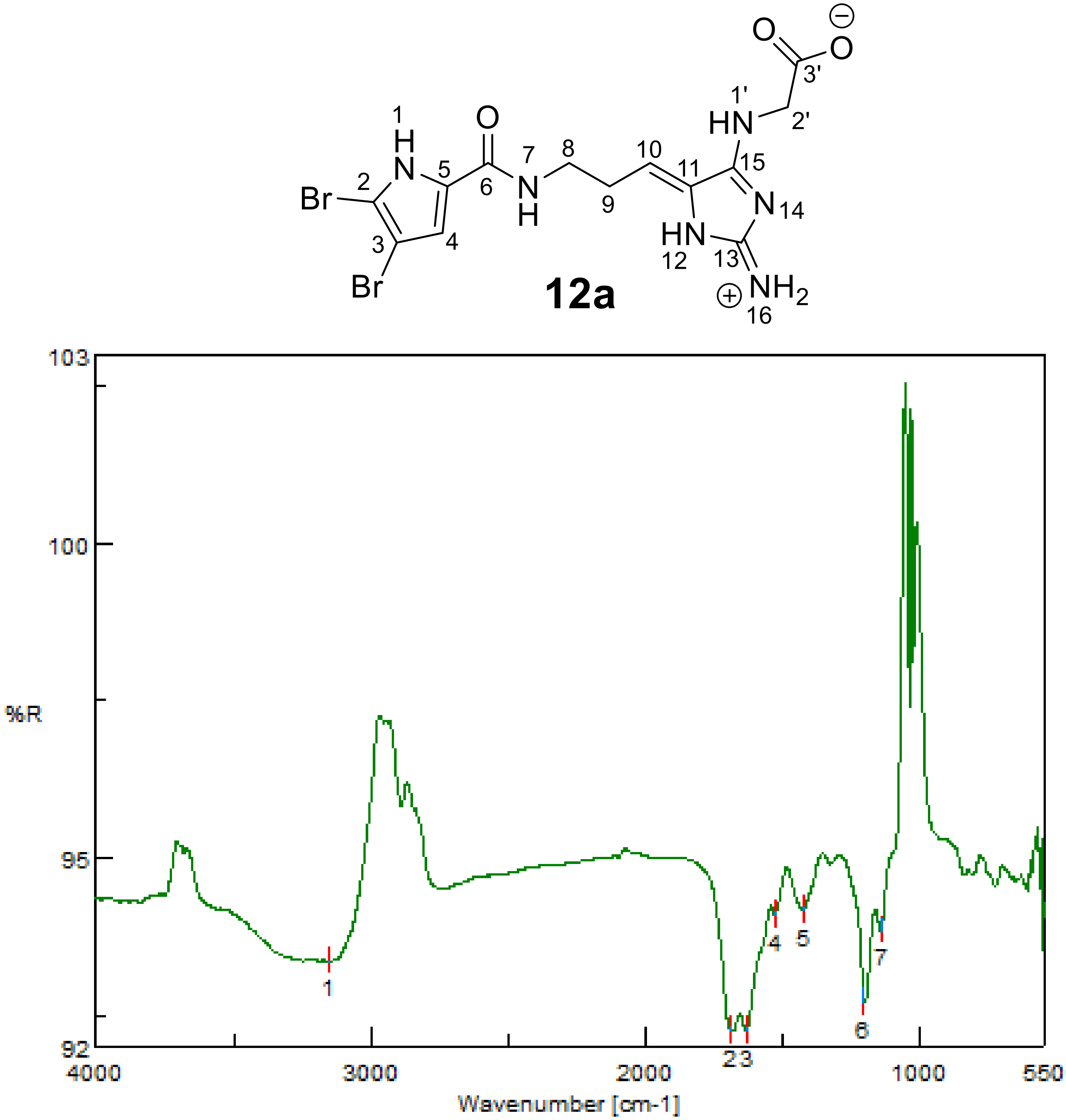

No.	Wavenumber	Strength
1	3152.1	93.3572
2	1689.3	92.2614
3	1634.4	92.2565
4	1530.2	94.109
5	1427.1	94.1748
6	1205.3	92.7057
7	1143.6	93.8527

Figure S59. IR spectrum of **12a** (ATR).

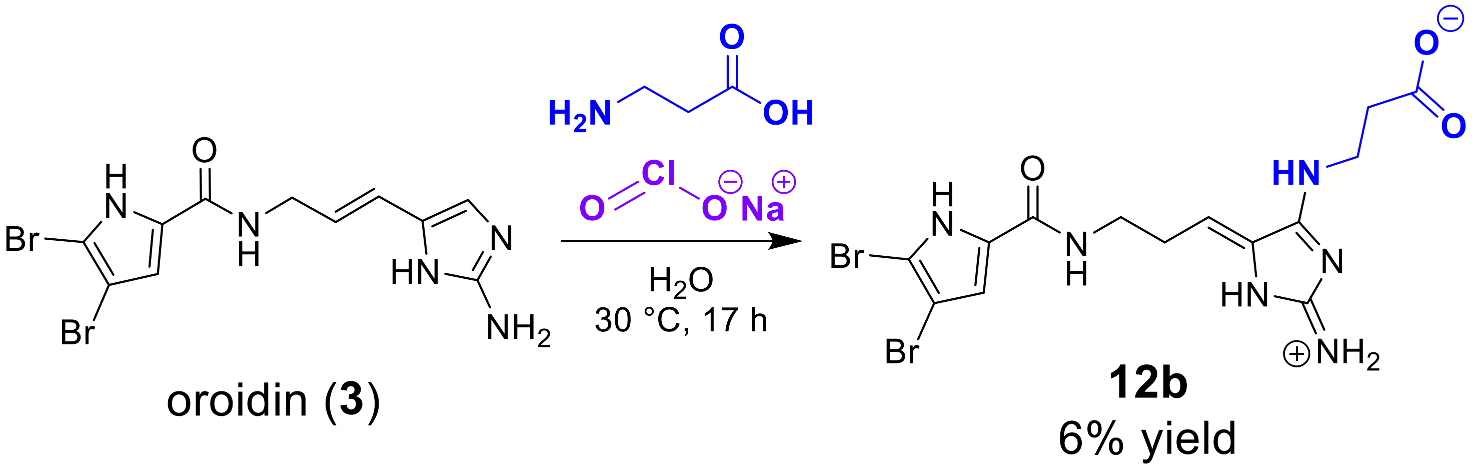

Scheme S2. Synthesis of **12b**.

Five batches of oroidin (**3**) (HCOOH salt, 3.0 mg, 0.0069 mmol each; 15 mg, 0.034 mmol in total) were placed in 20 mL round-bottomed flasks, and H_2O (4.0 mL) was added to each flask with stirring. β-Alanine (0.89 g, 10 mmol, 1449 equiv.) was then added to the mixtures, followed by addition of $NaClO_2$ (120 mg, 1.33 mmol, 193 equiv.). The flasks were sealed with septa caps, and the reaction mixtures were stirred at 30 °C for 17 h. After completion, the mixtures were combined and filtered through a small pad of Celite, rinsing the flasks and filter cake with H_2O. The filtrate was directly purified by ODS silica gel column chromatography (MeOH/H_2O, 20:80 to 40:60, v/v). The eluate was concentrated under reduced pressure, and the crude material was filtered through a Cosmospin filter H (0.45 μm). Further purification was performed by RP-HPLC (InertSustain AQ-C18, 5 μm, 10 mm i.d. × 250 mm; GL Science) using gradient elution (0–4 min, MeOH/H_2O/HCOOH = 3:97:0.1 to 40:60:0.1, v/v; 4 min–, 40:60:0.1) at a flow rate of 2.0 mL/min. Pure **12b** was obtained at 31-49 min (1.03 mg, 0.00216 mmol, 6% yield) as a slightly yellow solid.

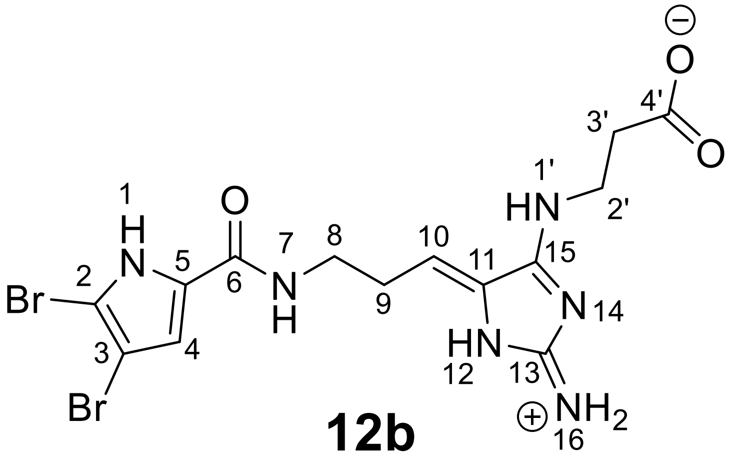

12b

12b:

$\mathbf{R_f}$ = 0.23 ($CHCl_3$/MeOH/28% NH_3 aq. = 60:40:2, v/v/v; UV).

UV/vis λ_{max} (MeOH) nm (log ε): 313 (3.66), 270 (4.16), 200 (4.39).

^{1}H NMR (600 MHz, CD_3OD containing 0.1% TFA): *δ* 6.78 (s, C4-H, 1H), 6.13 (t, *J* = 7.8 Hz, C10-H, 1H), 3.73 (t, *J* = 6.6 Hz, C2'-H, 2H), 3.46 (t, *J* = 6.9 Hz, C8-H, 2H), 2.69 (t, *J* = 6.6 Hz, C3'-H, 2H), 2.59 (q, *J* = 7.2 Hz, C9-H, 2H).

^{1}H NMR (600 MHz, DMSO-d_6 containing 0.1% TFA): *δ* 12.71 (s, N1-H, 1H), 11.32 (s, N12-H, 1H), 9.61 (t, *J* = 5.4 Hz, N1'-H, 1H), 9.20 (s, N16-H, 1H), 8.49 (s, N16-H, 1H), 8.26 (t, *J* = 5.7 Hz, N7-H, 1H), 6.91 (d, *J* = 3.0 Hz, C4-H, 1H), 6.23 (t, *J* = 7.5 Hz, C10-H, 1H), 3.58 (COSY) (overlayed by HDO signal) (C2'-H, 2H), 3.34 (q, *J* = 6.4 Hz, C8-H, 2H), 2.60 (t, *J* = 6.6 Hz, C3'-H, 2H), 2.54 (q, *J* = 6.8 Hz, C9-H, 2H).

^{13}C NMR (151 MHz, CD_3OD containing 0.1% TFA): *δ* 175.4 (C4'), 170.3 (C15), 168.9 (C13), 162.8 (C6), 135.7 (C11), 129.4 (C5), 116.5 (C10), 115.1 (C4), 107.2 (C2), 100.8 (C3), 41.0 (C2'), 39.9 (C8), 34.3 (C3'), 30.1 (C9).

HRMS (**ESI**): (*m/z*) calcd for $C_{14}H_{17}{}^{79}Br_2N_6O_3^+$ $[M+H]^+$: 474.9723, found 474.9711.

IR ν_{max}: 3186 (br), 1690 (s), 1530 (w), 1430 (w), 1201 (s), 1144 (m).

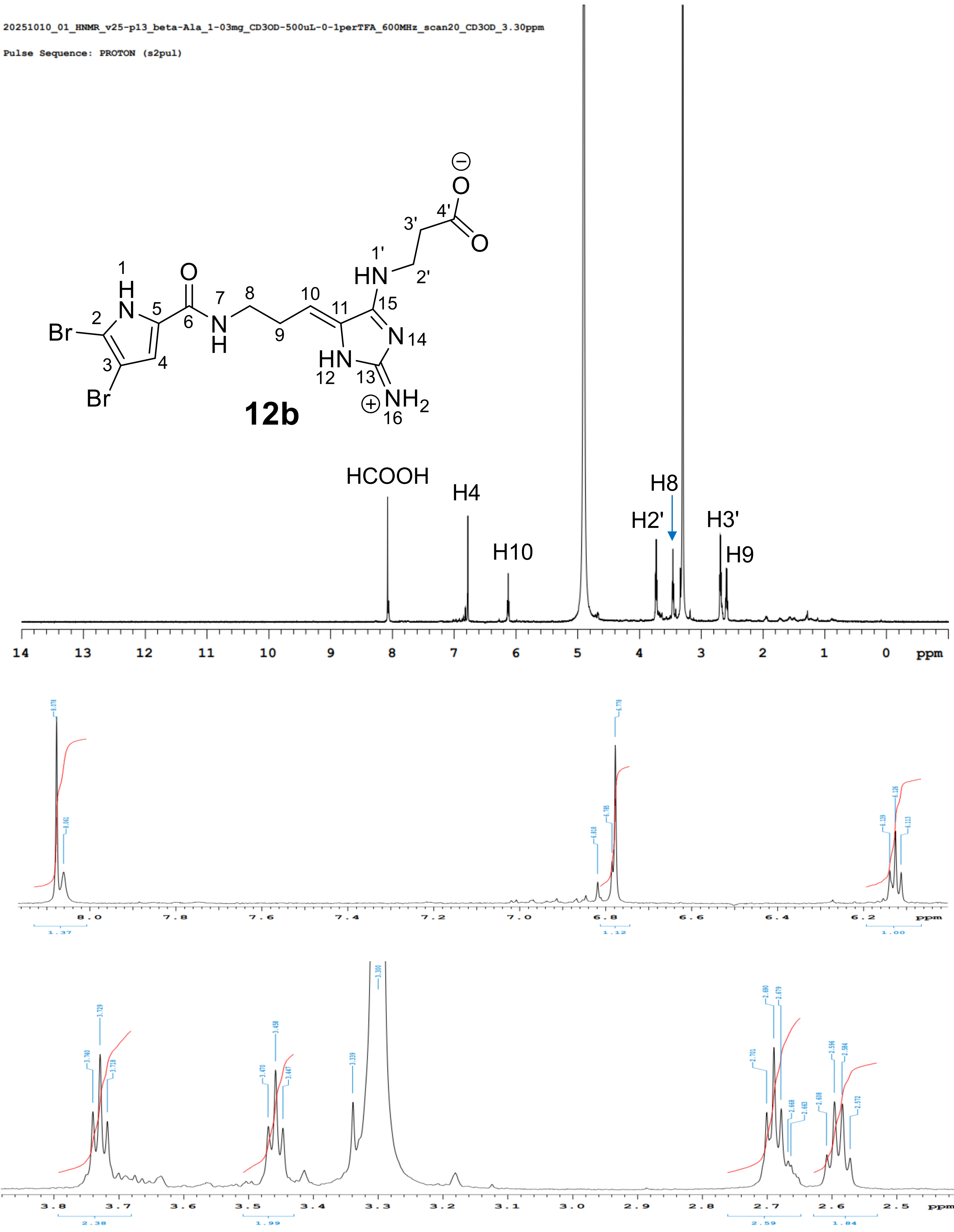

Figure S60. ^{1}H NMR spectrum of **12b** (1.03 mg) (600 MHz, CD_3OD: 500 µL - 0.1% TFA).

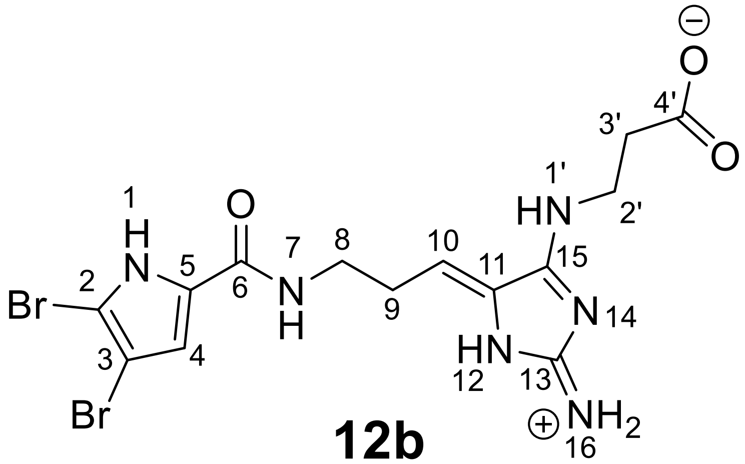

12b

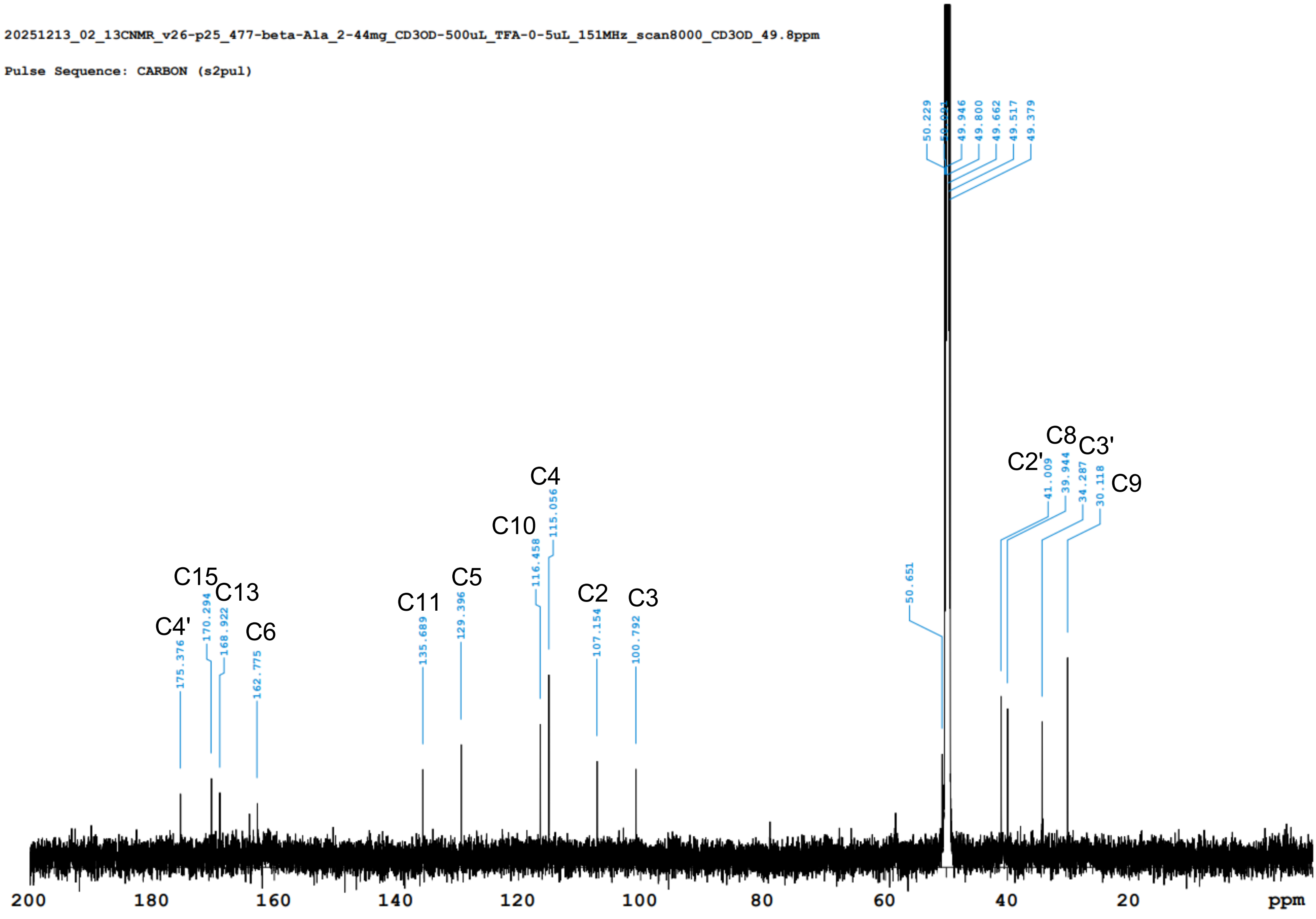

Figure S61. ^{13}C NMR spectrum of **12b** (2.44 mg) (151 MHz, CD_3OD: 500 µL - 0.1% TFA).

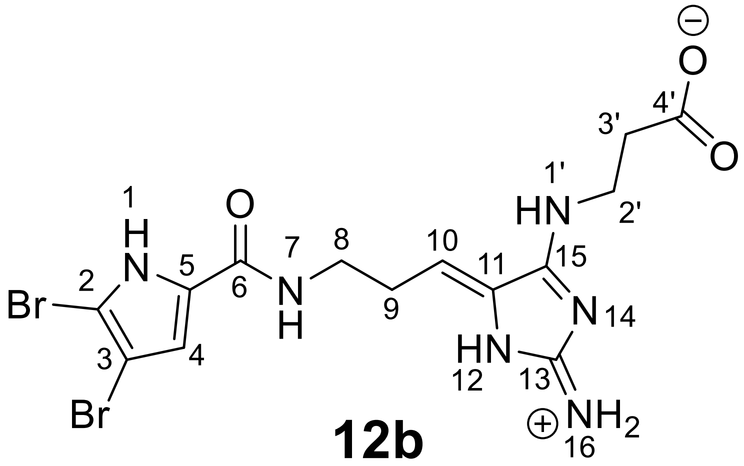

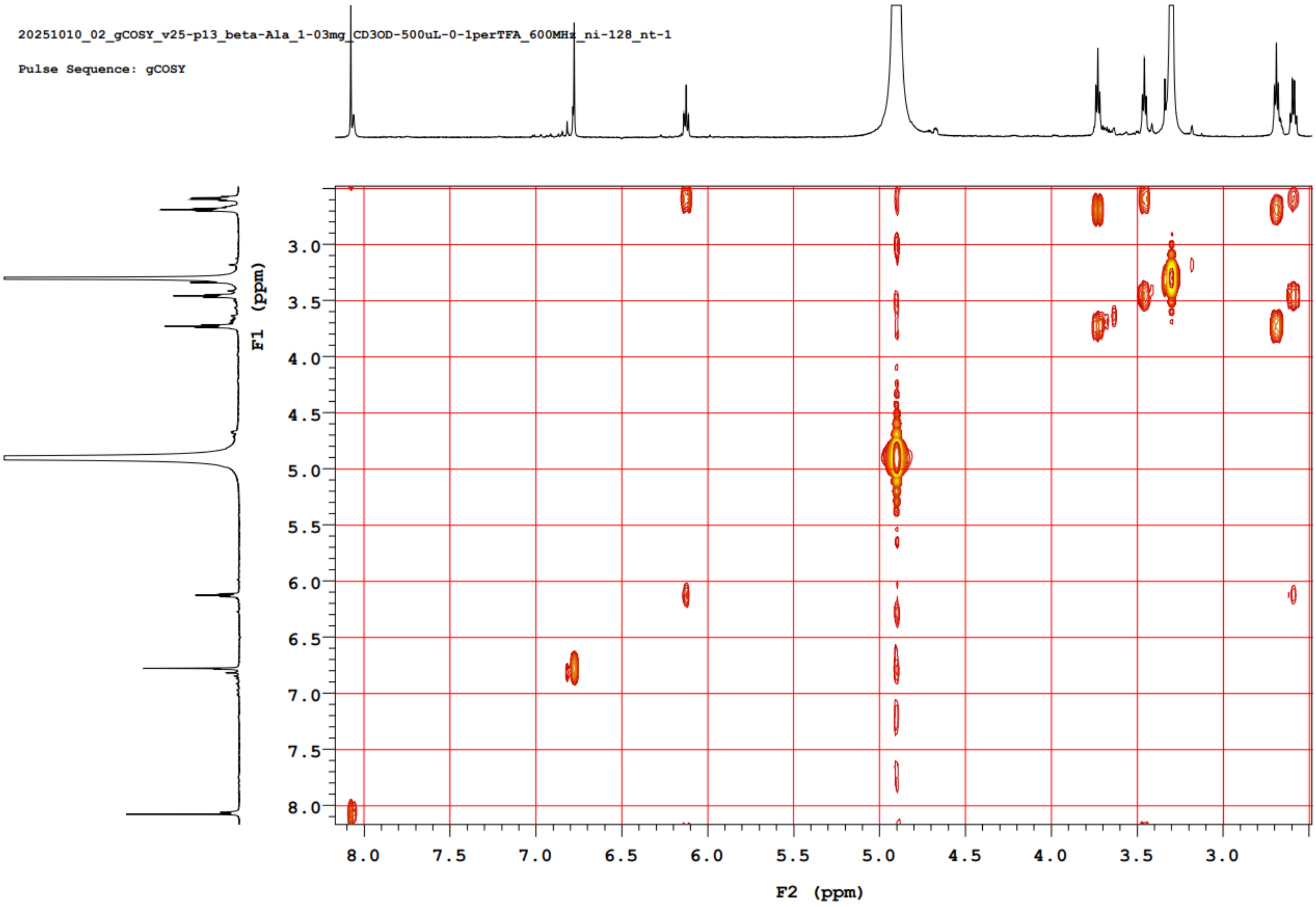

Figure S62. COSY spectrum of **12b** (1.03 mg) (600 MHz, CD_3OD: 500 µL - 0.1% TFA).

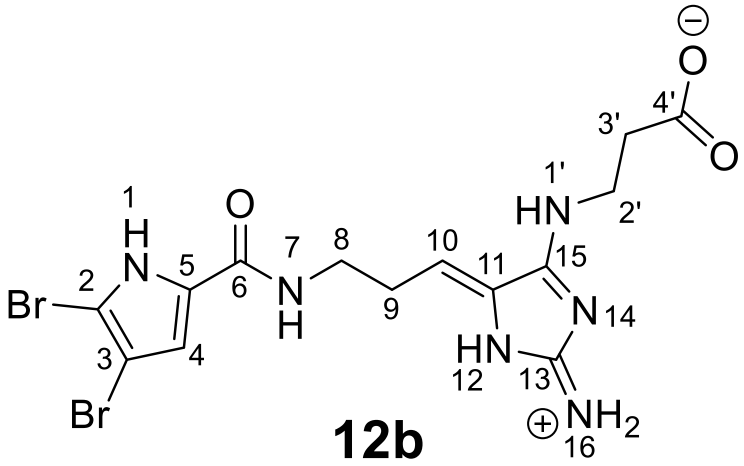

12b

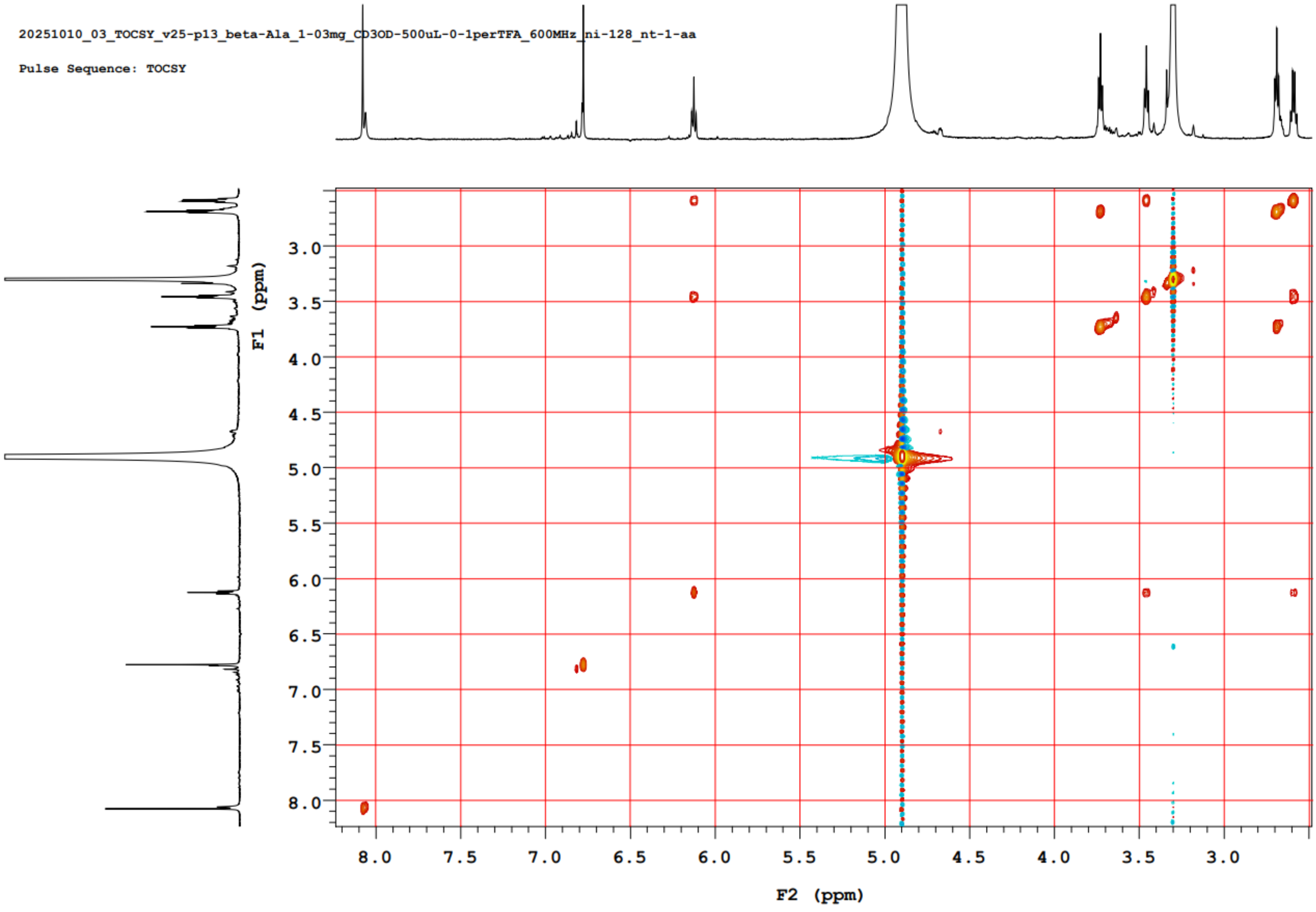

Figure S63. TOCSY spectrum of **12b** (1.03 mg) (600 MHz, CD_3OD: 500 µL - 0.1% TFA).

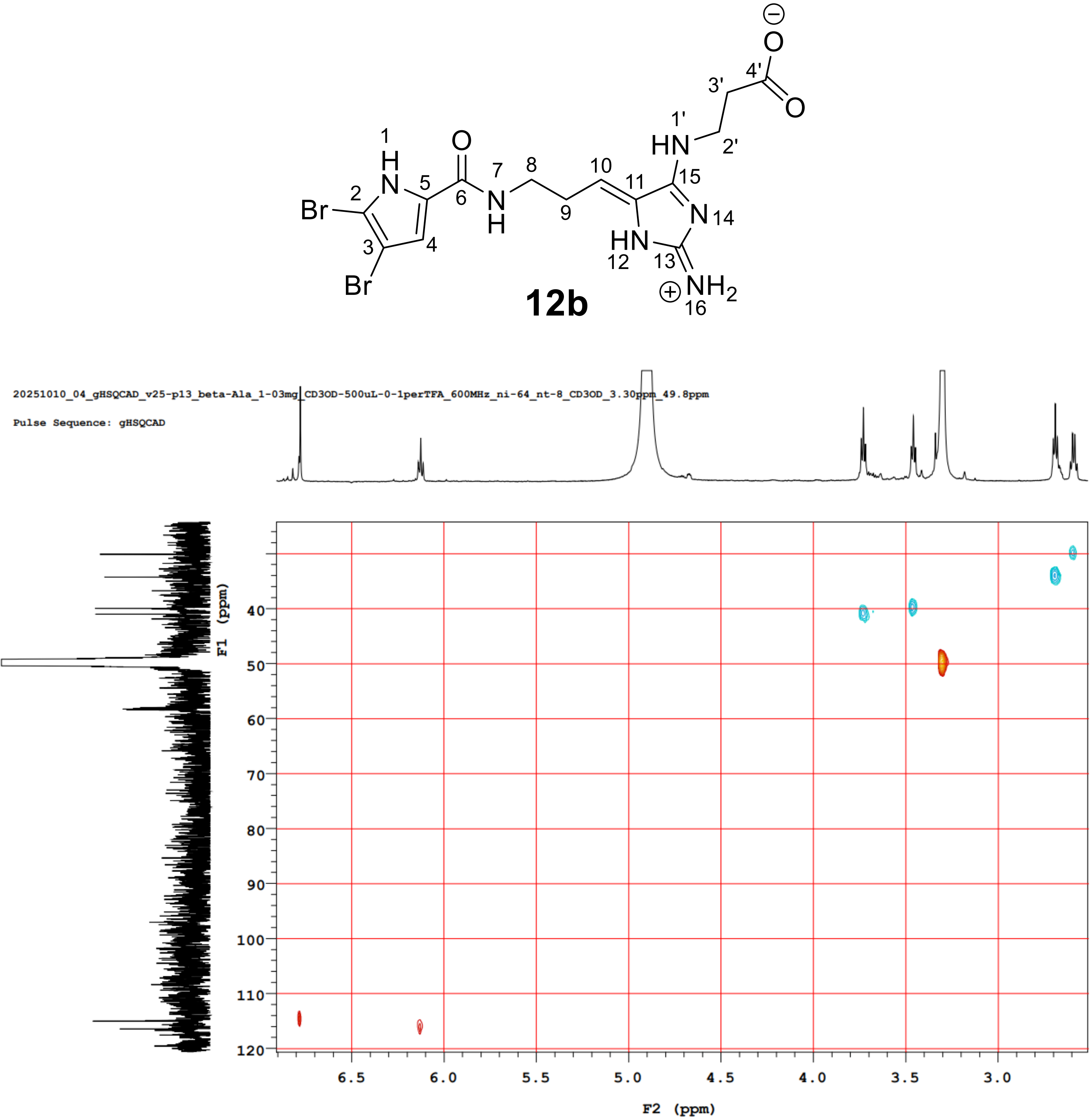

Figure S64. ^{1}H-^{13}C HSQC spectrum of **12b** (1.03 mg) (600 MHz/151 MHz, CD_3OD: 500 μL - 0.1% TFA).

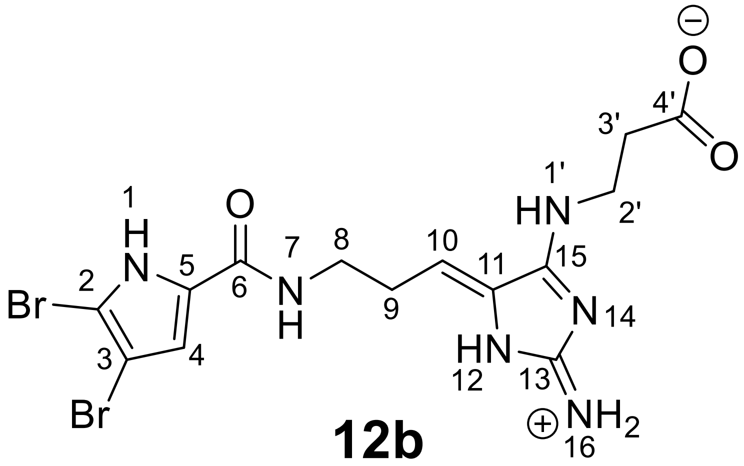

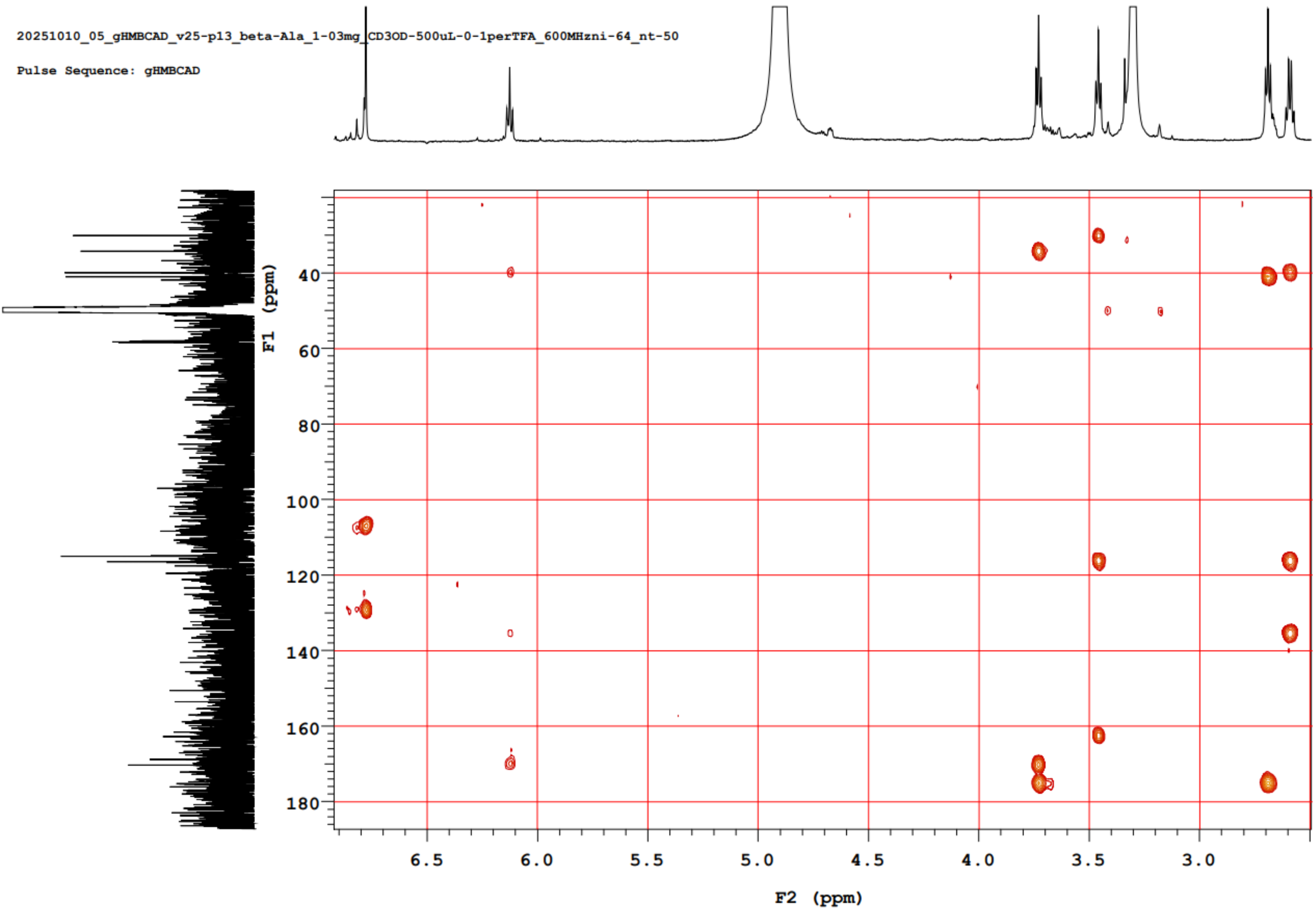

Figure S65. ^{1}H-^{13}C HMBC spectrum of **12b** (1.03 mg) (600 MHz/151 MHz, CD_3OD: 500 µL - 0.1% TFA).

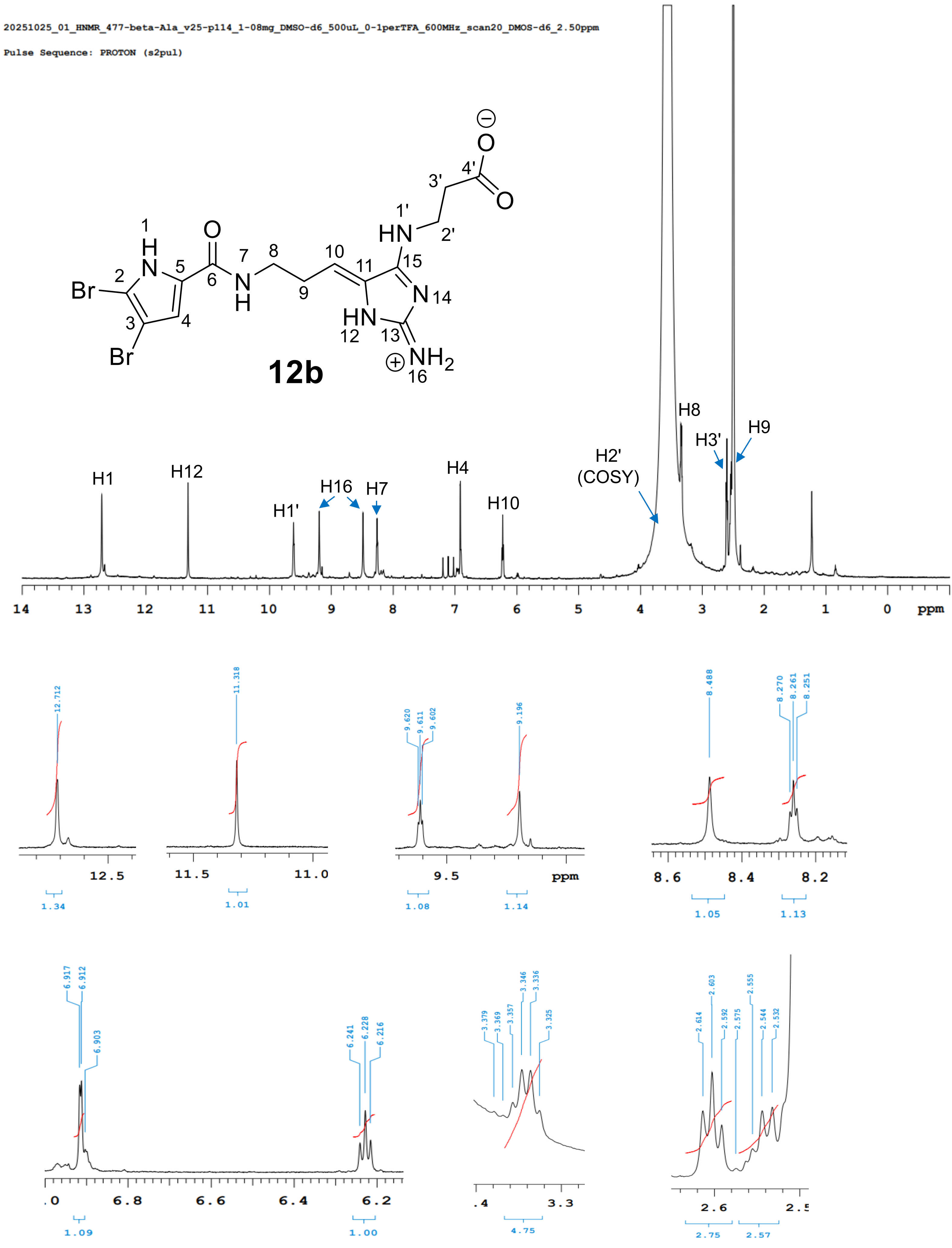

Figure S66. ^{1}H NMR spectrum of **12b** (1.08 mg) (600 MHz, DMSO-d_6: 500 µL - 0.1% TFA).

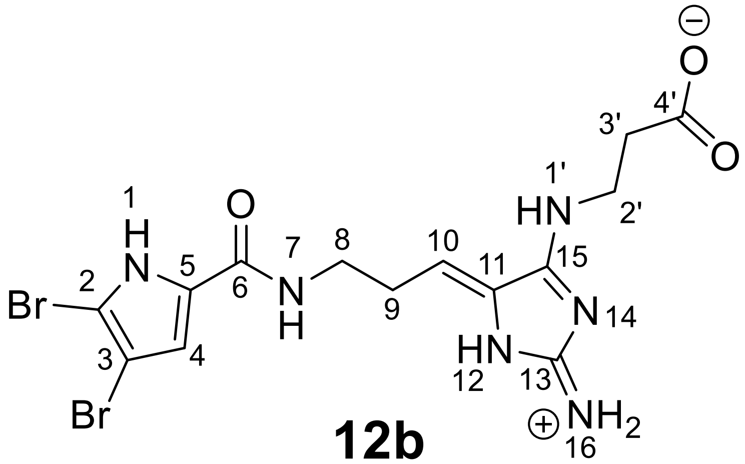

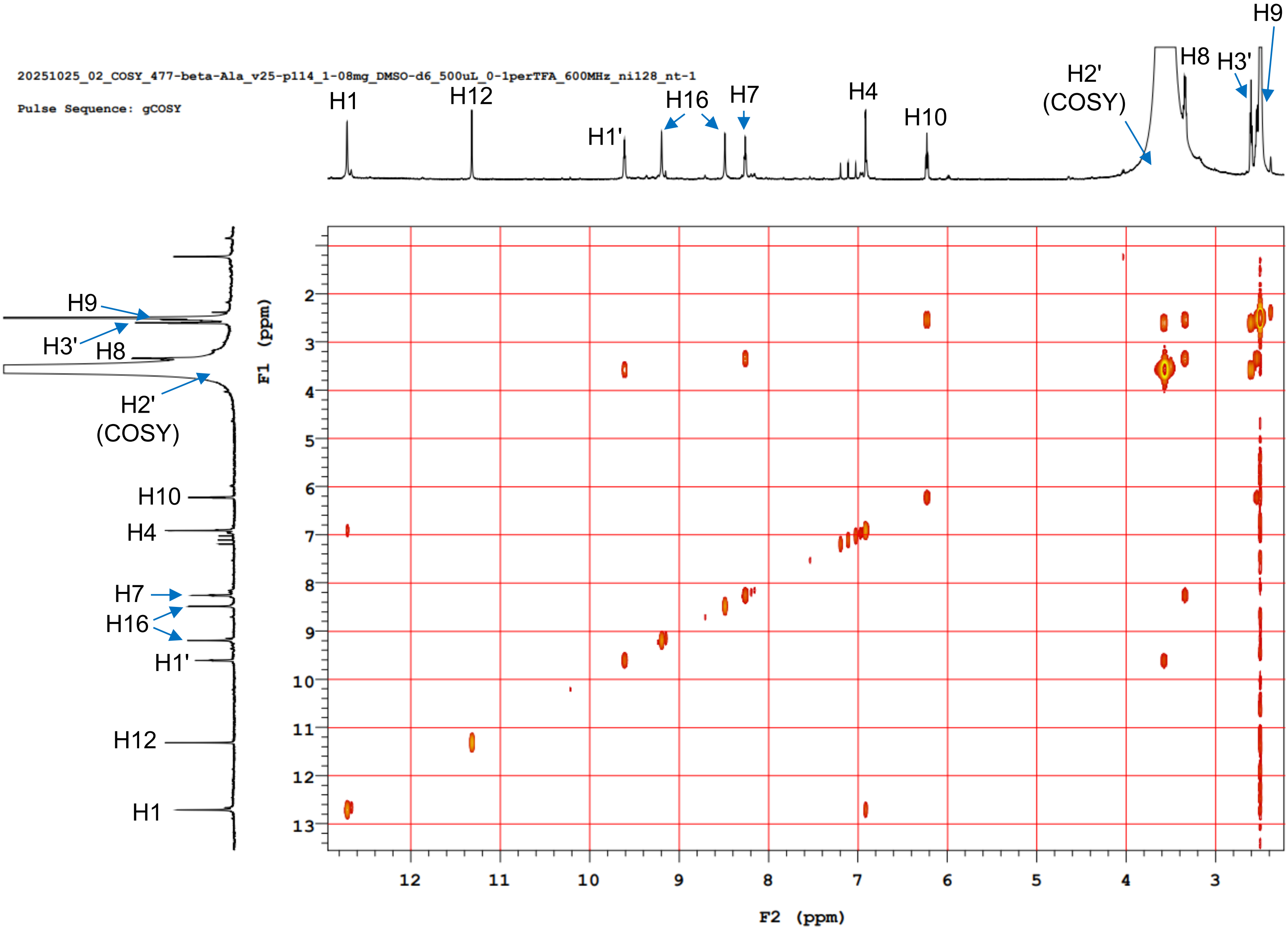

Figure S67. COSY spectrum of **12b** (1.08 mg) (600 MHz, DMSO-d_6: 500 µL - 0.1% TFA).

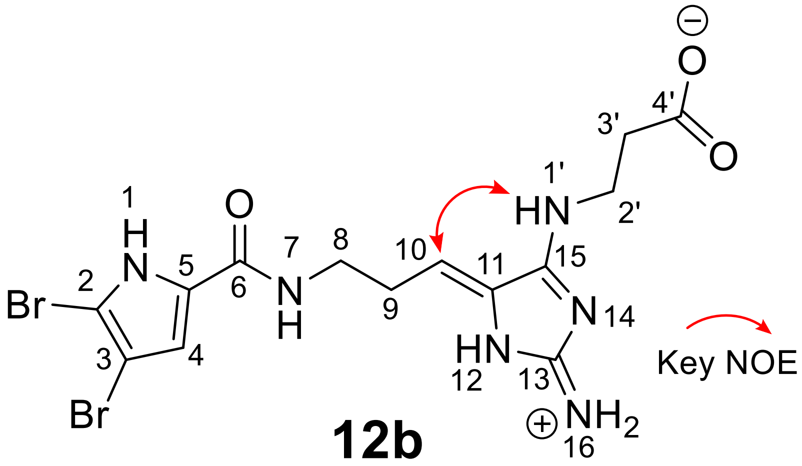

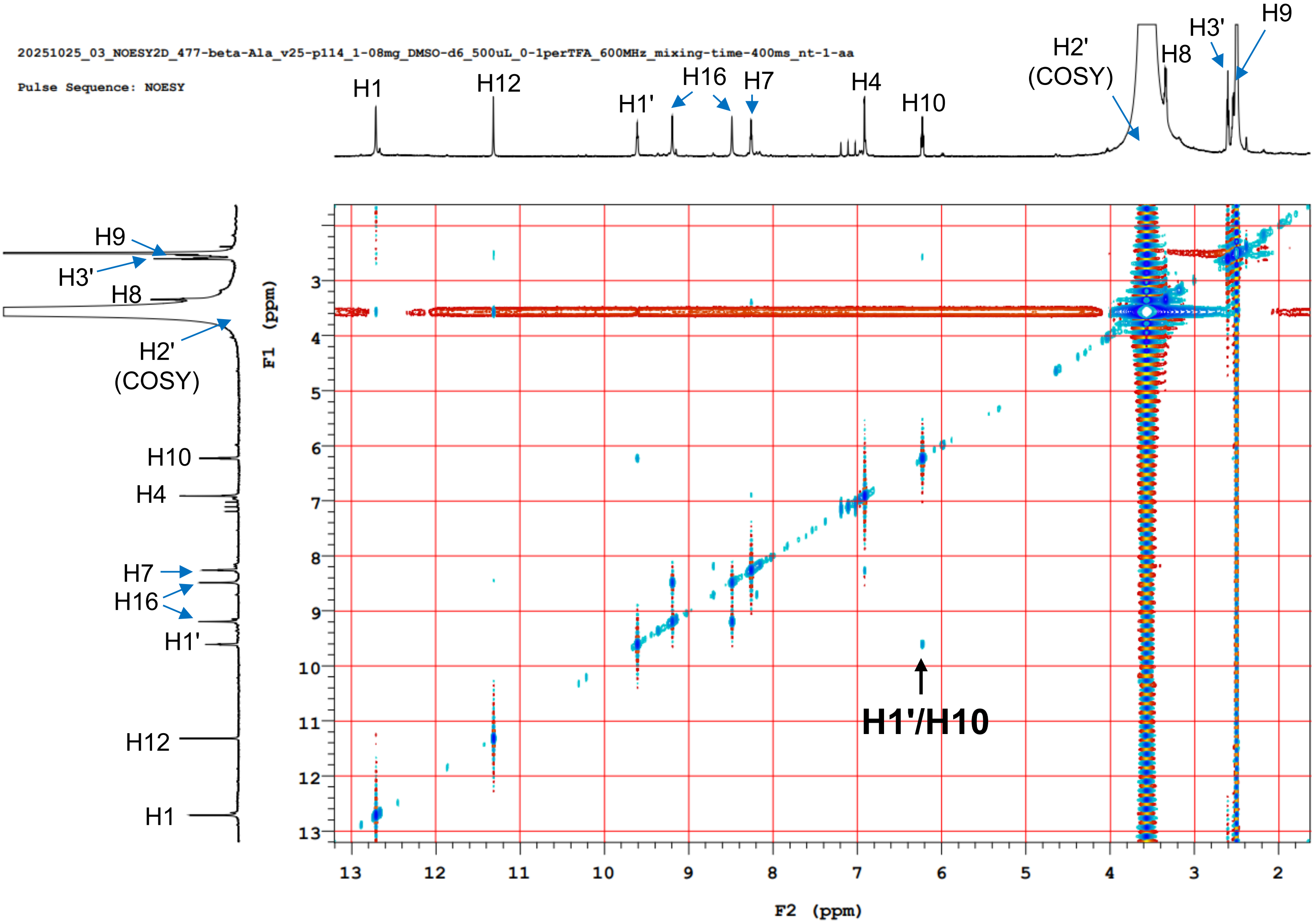

Figure S68. NOESY spectrum of **12b** (1.08 mg) (600 MHz, DMSO-d_6: 500 µL - 0.1% TFA).

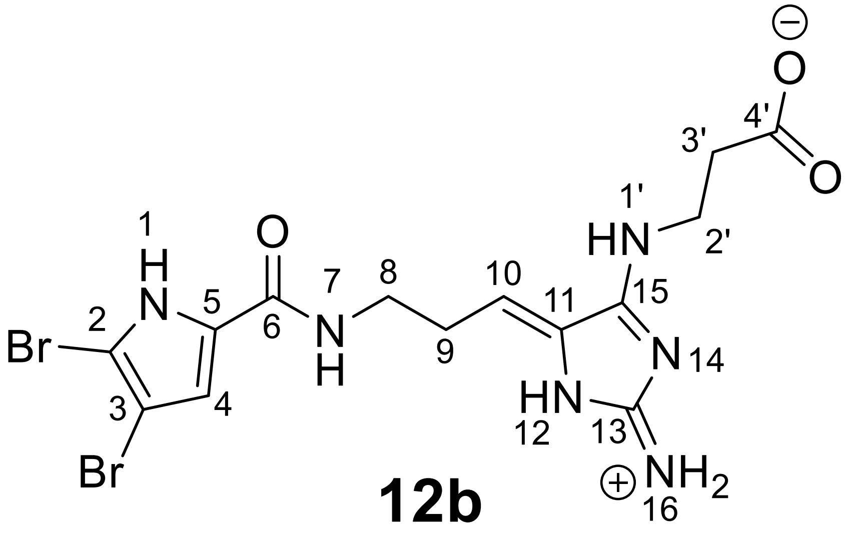

12b

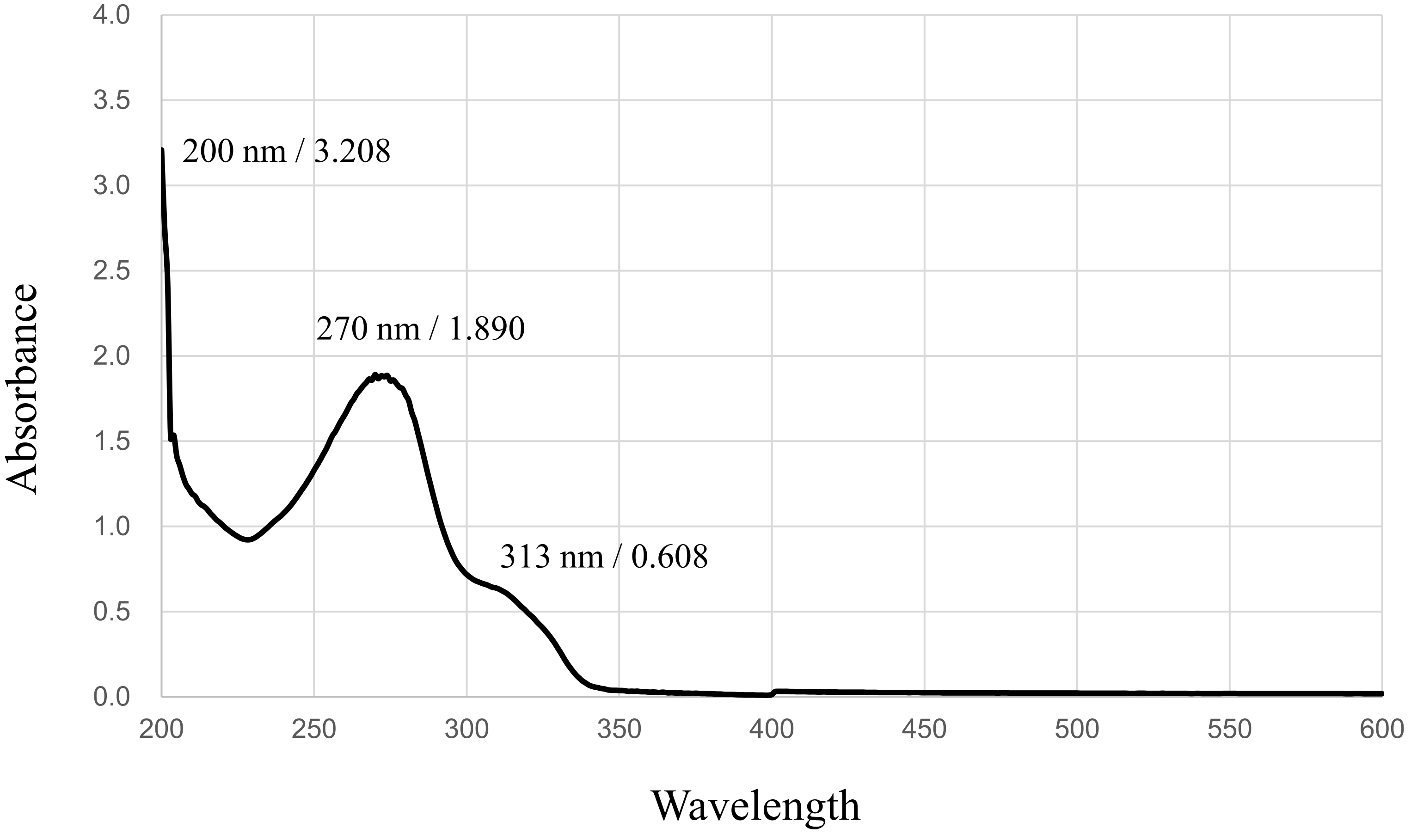

Figure S69. UV absorption spectrum of **12b** (MeOH). c = 1.32 x 10^{-4} (M)

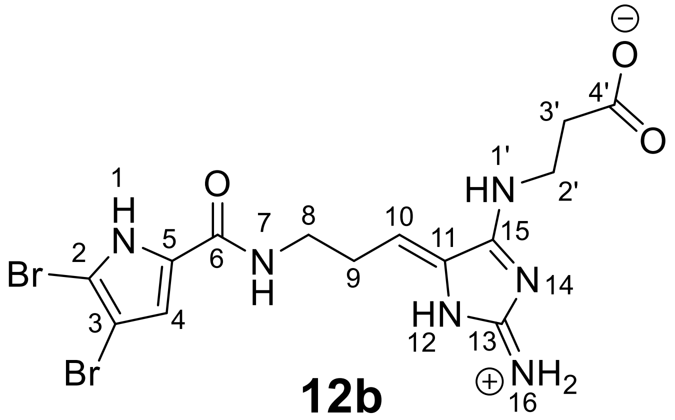

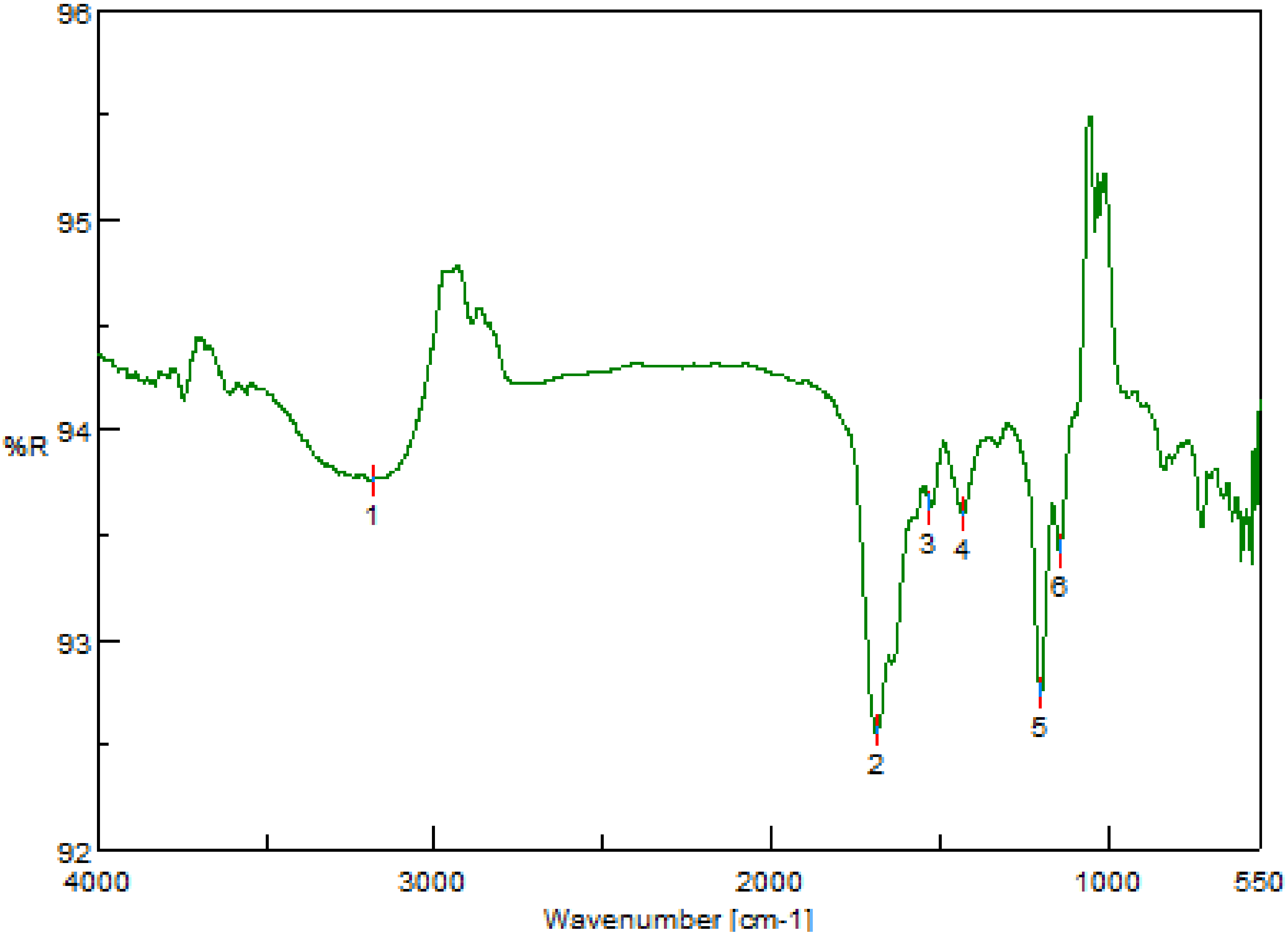

No.	Wavenumber	Strength
1	3185.8	93.754
2	1690.3	92.5632
3	1530.2	93.6254
4	1430.0	93.5937
5	1200.5	92.7407
6	1143.6	93.4149

Figure S70. IR spectrum of **12b** (ATR).

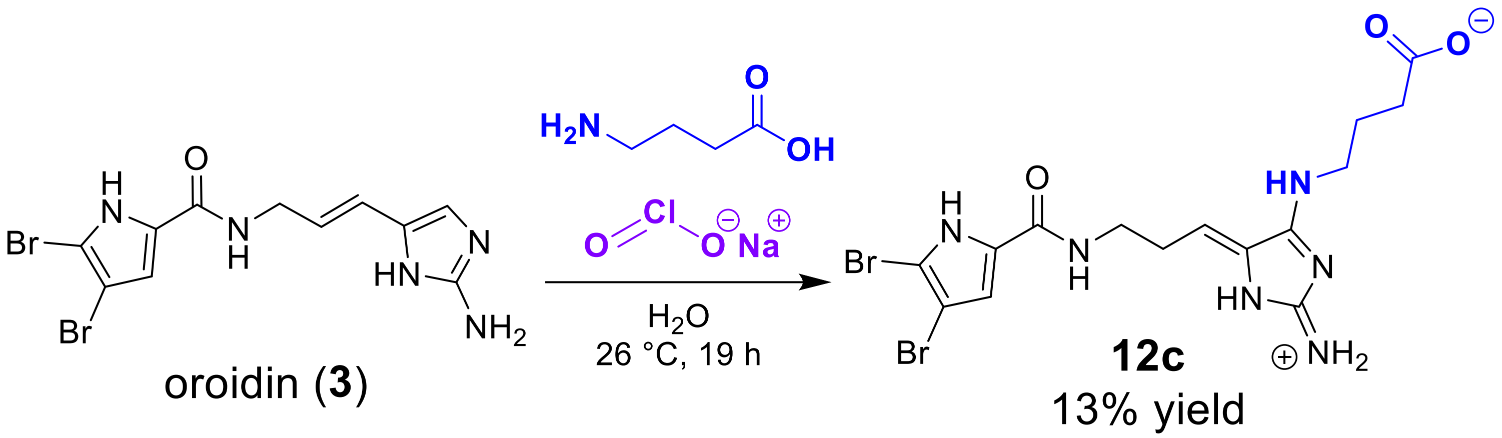

Scheme S3. Synthesis of **12c**.

Five batches of oroidin (**3**) (HCOOH salt, 3.0 mg, 0.0069 mmol each; 15 mg, 0.034 mmol in total) were placed in 20 mL round-bottomed flasks, and H_2O (4.0 mL) was added to each flask with stirring. γ-Aminobutyric acid (1.03 g, 10 mmol, 1449 equiv.) was then added to the mixtures, followed by addition of $NaClO_2$ (120 mg, 1.33 mmol, 193 equiv.). The flasks were sealed with septa caps, and the reaction mixtures were stirred at 26 °C for 19 h. After completion, the mixtures were combined and filtered through a small pad of Celite, rinsing the flasks and filter cake with H_2O. The filtrate was directly purified by ODS silica gel column chromatography (MeOH/H_2O, 20:80 to 50:50, v/v). The eluate was concentrated under reduced pressure, and the crude material was filtered through a Cosmospin filter H (0.45 μm). Further purification was performed by RP-HPLC (InertSustain AQ-C18, 5 μm, 10 mm i.d. × 250 mm; GL Science) using gradient elution (0–4 min, MeOH/H_2O/HCOOH = 3:97:0.1 to 40:60:0.1, v/v; 4-40 min, 40:60:0.1; 40.1 min-, 100:0:0.1) at a flow rate of 2.0 mL/min. Semi-pure **12c** was obtained at 28-55 min and was further purified by RP-HPLC (InertSustain AQ-C18, 5 μm, 10 mm i.d. × 250 mm; GL Science) again using gradient elution (0–4 min, MeOH/H_2O/HCOOH = 3:97:0.1 to 50:50:0.1, v/v; 4 min-, 50:50:0.1) at a flow rate of 2.0 mL/min. Pure **12c** was obtained at 17-22 min (2.14 mg, 0.0044 mmol, 13% yield) as a slightly yellow film.

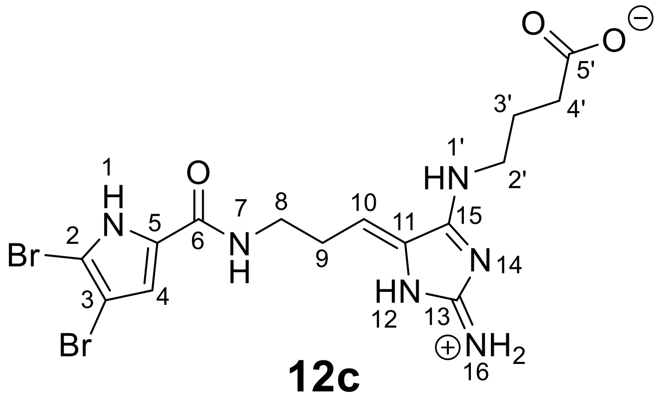

12c

12c:

$\mathbf{R_f}$ = 0.31 ($CHCl_3$/MeOH/28% NH_3 aq. = 60:40:2, v/v/v; UV).

UV/vis λ_{max} (MeOH) nm (log ε): 313 (3.81), 269 (4.27), 201 (4.41).

^{1}H NMR (600 MHz, CD_3OD containing 0.1% TFA): *δ* 6.78 (s, C4-H, 1H), 6.10 (t, *J* = 7.8 Hz, C10-H, 1H), 3.55 (t, *J* = 6.9 Hz, C2'-H, 2H), 3.47 (t, *J* = 6.9 Hz, C8-H, 2H), 2.59 (q, *J* = 7.2 Hz, C9-H, 2H), 2.37 (t, *J* = 7.2 Hz, C4'-H, 2H), 1.94 (quintet, *J* = 7.2 Hz, C3'-H, 2H).

^{1}H NMR (600 MHz, DMSO-d_6 containing 0.1% TFA): *δ* 12.72 (d, *J* = 2.4 Hz, N1-H, 1H), 11.30 (s, N12-H, 1H), 9.56 (t, *J* = 5.7 Hz, N1'-H, 1H), 9.16 (s, N16-H, 1H), 8.46 (s, N16-H, 1H), 8.27 (t, *J* = 5.4 Hz, N7-H, 1H), 6.92 (d, *J* = 3.0 Hz, C4-H, 1H), 6.21 (t, *J* = 7.8 Hz, C10-H, 1H), 3.41 (q, *J* = 6.6 Hz, C2'-H, 2H), 3.35 (q, *J* = 6.2 Hz, C8-H, 2H), 2.54 (q, *J* = 7.0 Hz, C9-H, 2H), 2.29 (t, *J* = 7.5 Hz, C4'-H, 2H), 1.80 (quintet, *J* = 7.2 Hz, C3'-H, 2H).

^{13}C NMR (151 MHz, CD_3OD containing 0.1% TFA): *δ* 177.4 (C5'), 171.4 (C15), 169.0 (C13), 162.8 (C6), 135.7 (C11), 129.4 (C5), 116.1 (C10), 115.0 (C4), 107.2 (C2), 100.8 (C3), 44.5 (C2'), 39.9 (C8), 32.7 (C4'), 30.1 (C9), 25.8 (C3').

HRMS (**ESI**): (*m/z*) calcd for $C_{15}H_{19}{}^{79}Br_2N_6O_3^+$ $[M+H]^+$: 488.9880, found 488.9880.

IR $\mathbf{v_{max}}$: 3147 (br), 1694 (s), 1636 (s), 1530 (w), 1428 (w), 1198 (s), 1141 (w).

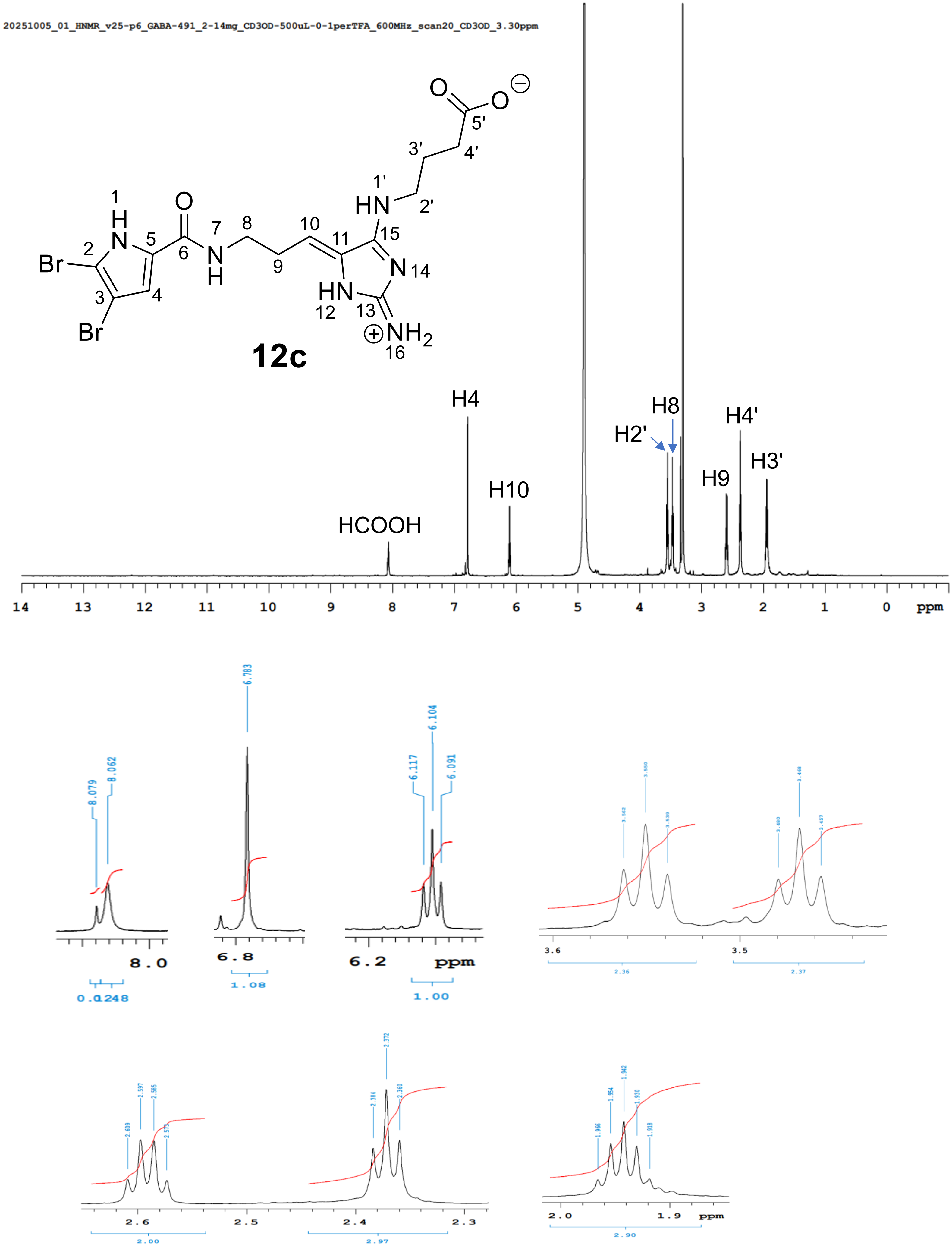

Figure S71. ^{1}H NMR spectrum of **12c** (2.14 mg) (600 MHz, CD_3OD: 500 µL - 0.1% TFA).

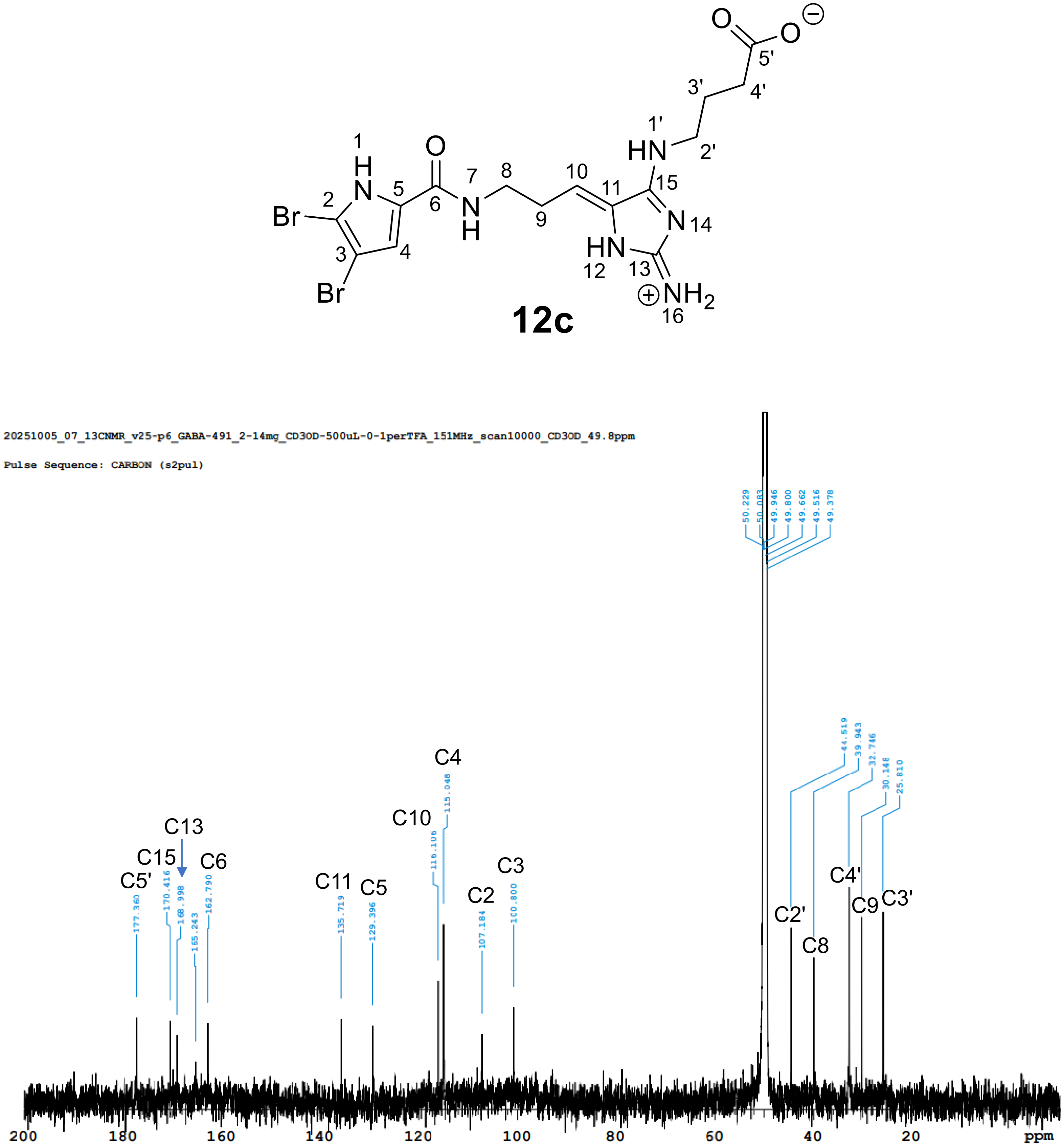

Figure S72. ^{13}C NMR spectrum of **12c** (2.14 mg) (151 MHz, CD_3OD: 500 µL - 0.1% TFA).

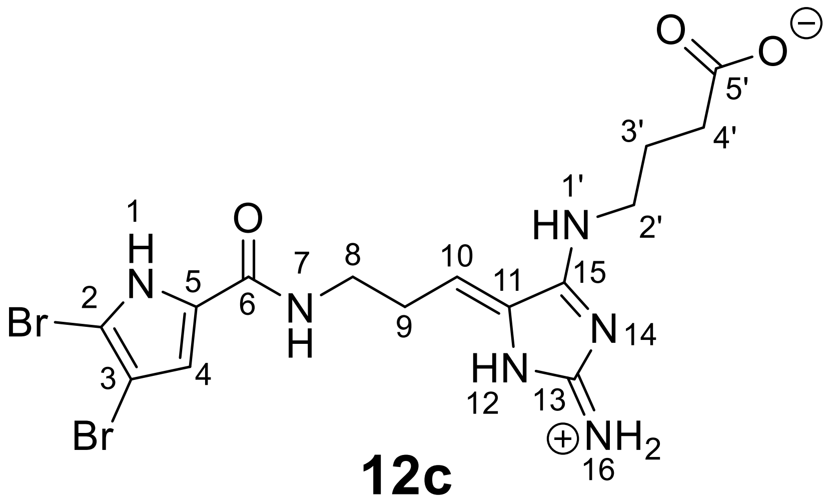

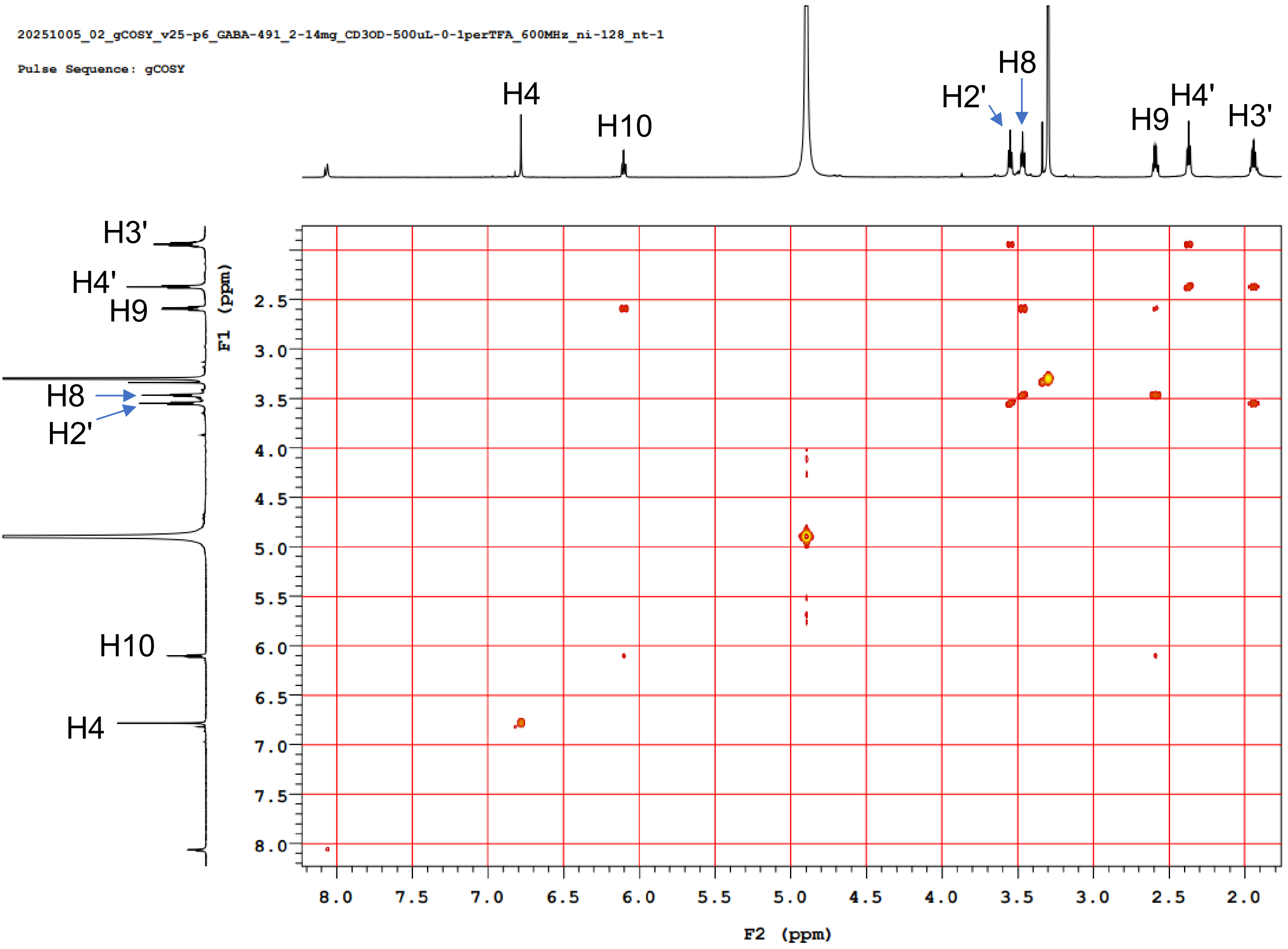

Figure S73. COSY spectrum of **12c** (2.14 mg) (600 MHz, CD_3OD: 500 µL - 0.1% TFA).

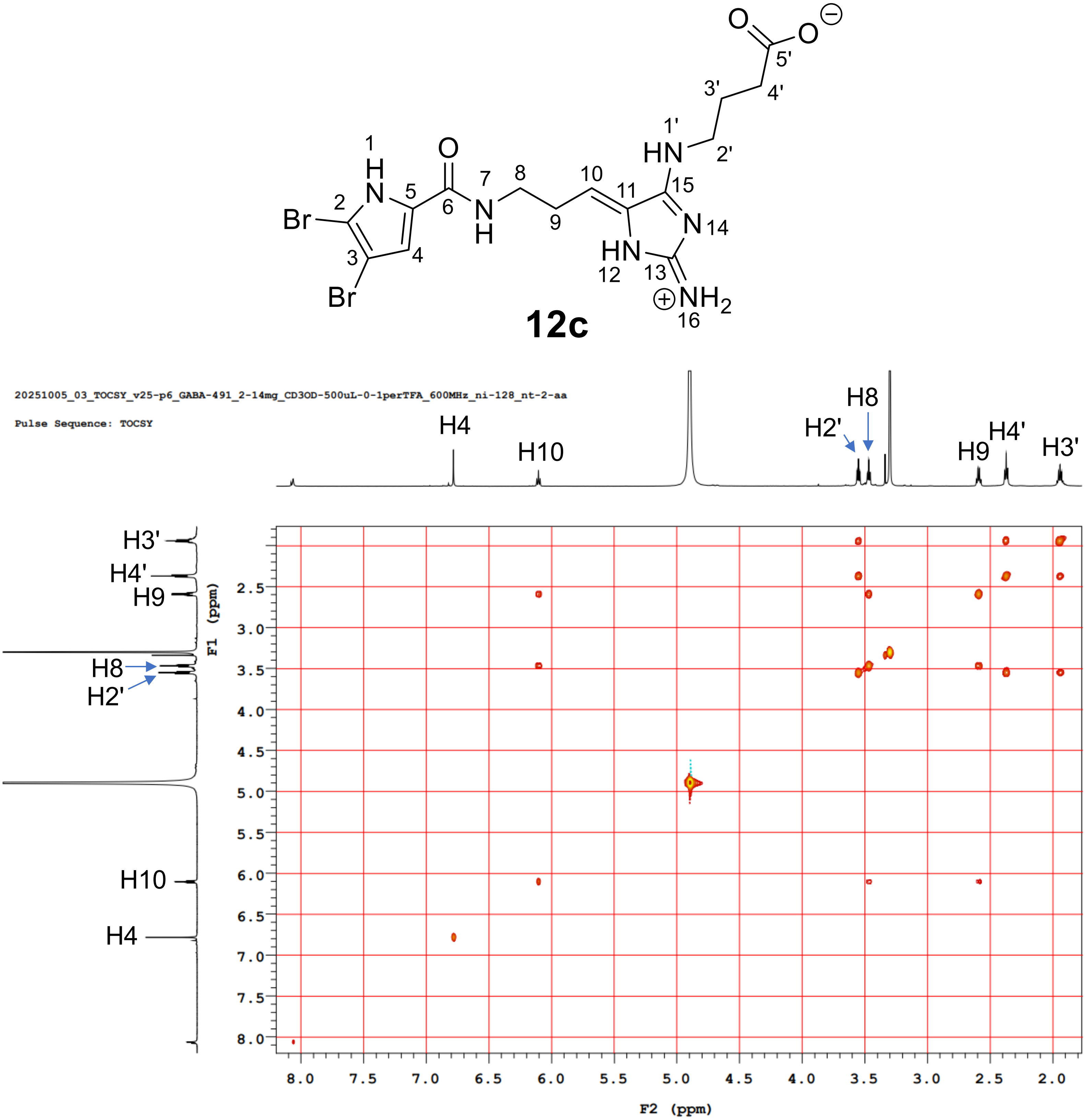

Figure S74. TOCSY spectrum of **12c** (2.14 mg) (600 MHz, CD_3OD: 500 µL - 0.1% TFA).

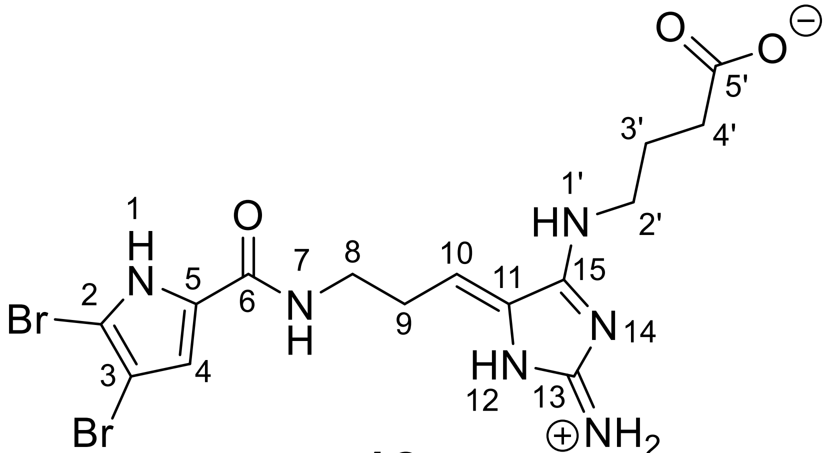

12c

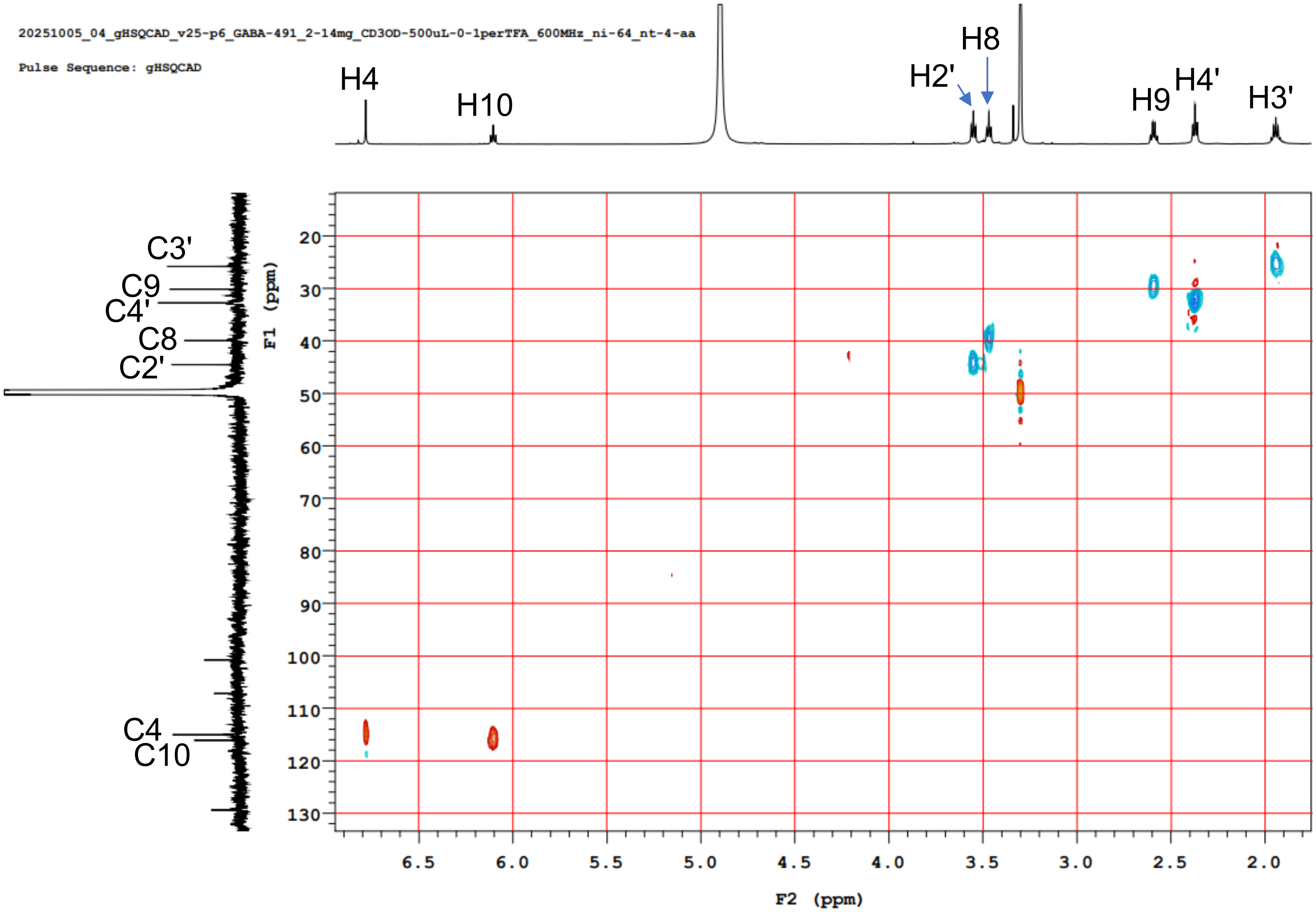

Figure S75. ^{1}H-^{13}C HSQC spectrum of **12c** (2.14 mg) (600 MHz /151 MHz, CD_3OD: 500 µL - 0.1% TFA).

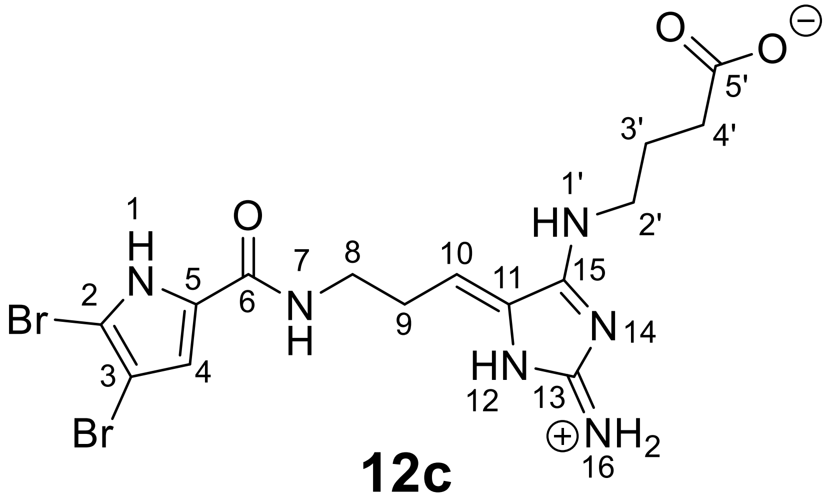

12c

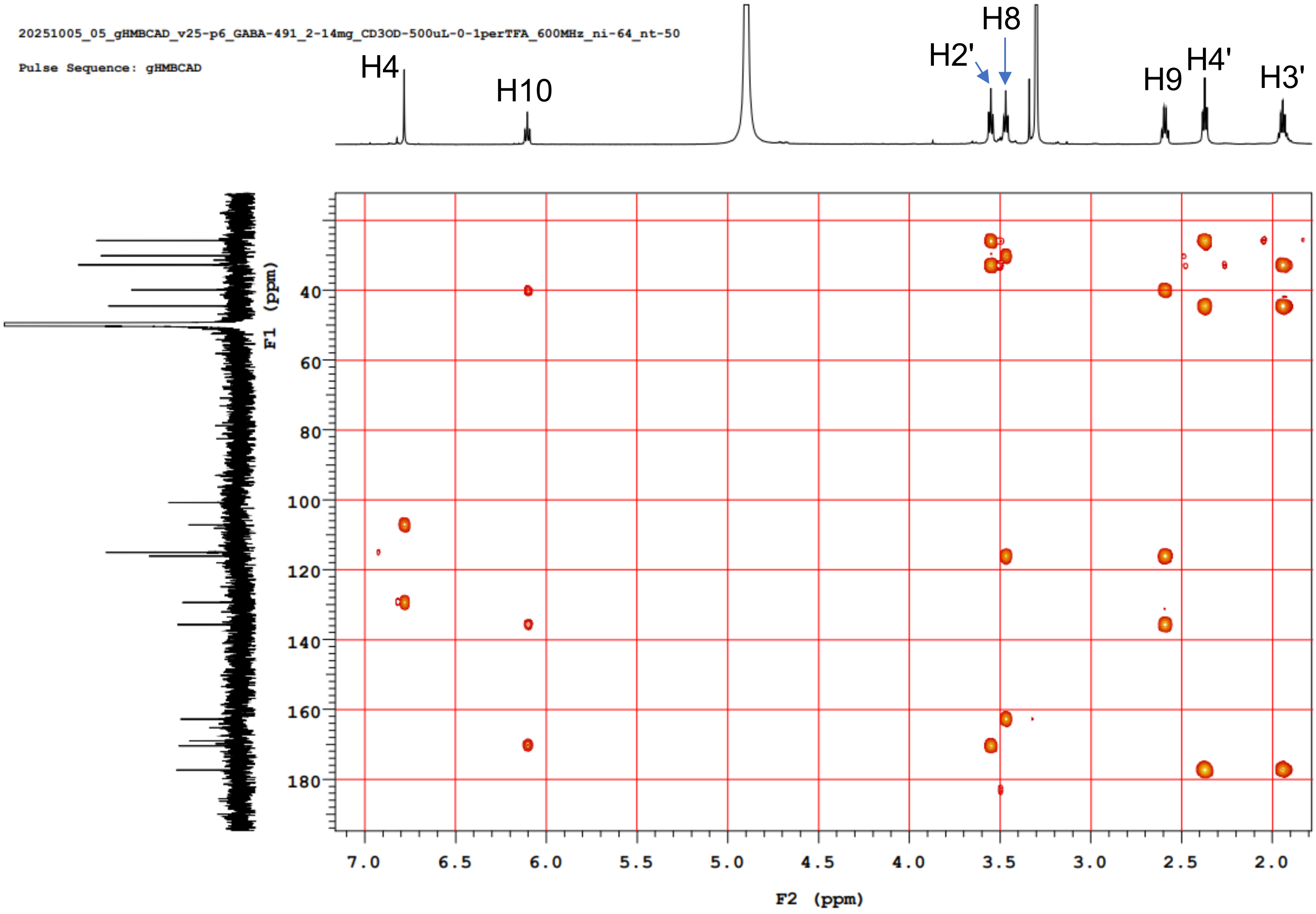

Figure S76. ^{1}H-^{13}C HMBC spectrum of **12c** (2.14 mg) (600 MHz /151 MHz, CD_3OD: 500 µL - 0.1% TFA).

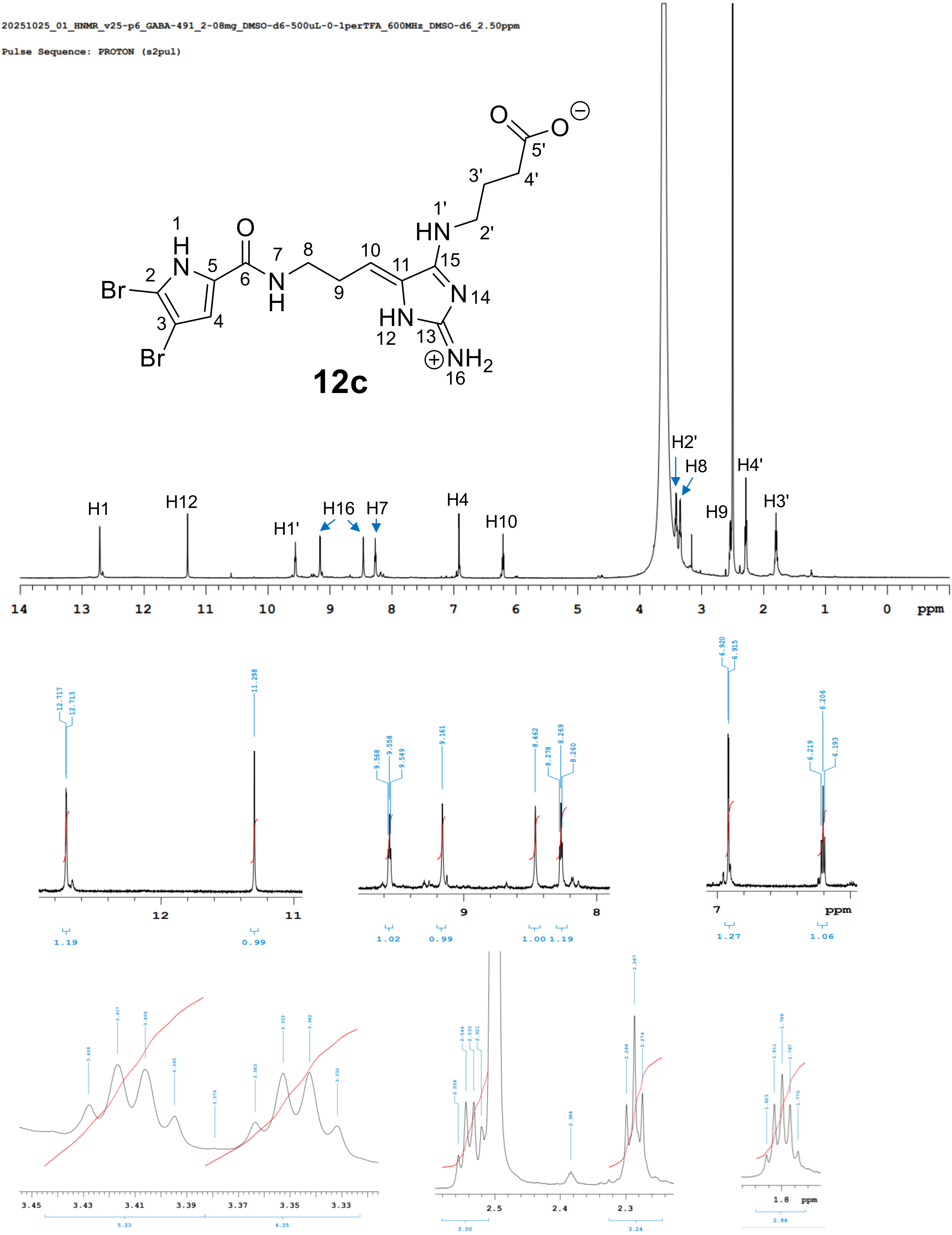

Figure S77. ^{1}H NMR spectrum of **12c** (2.08 mg) (600 MHz, DMSO-d_6: 500 µL - 0.1% TFA).

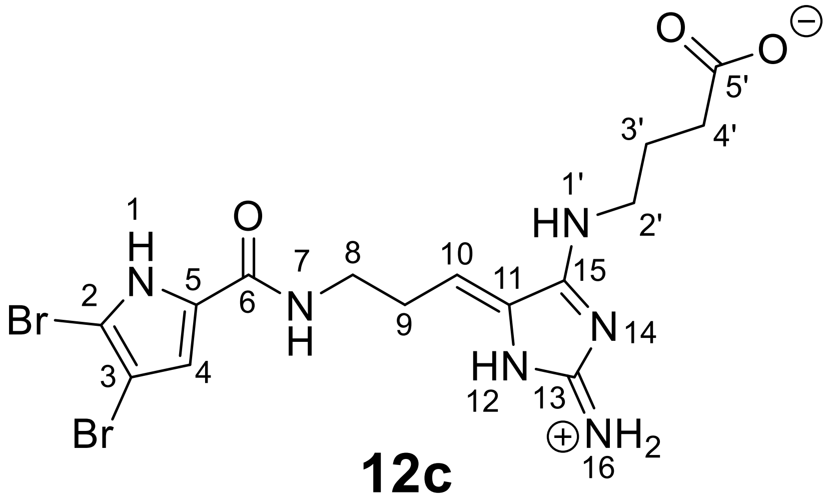

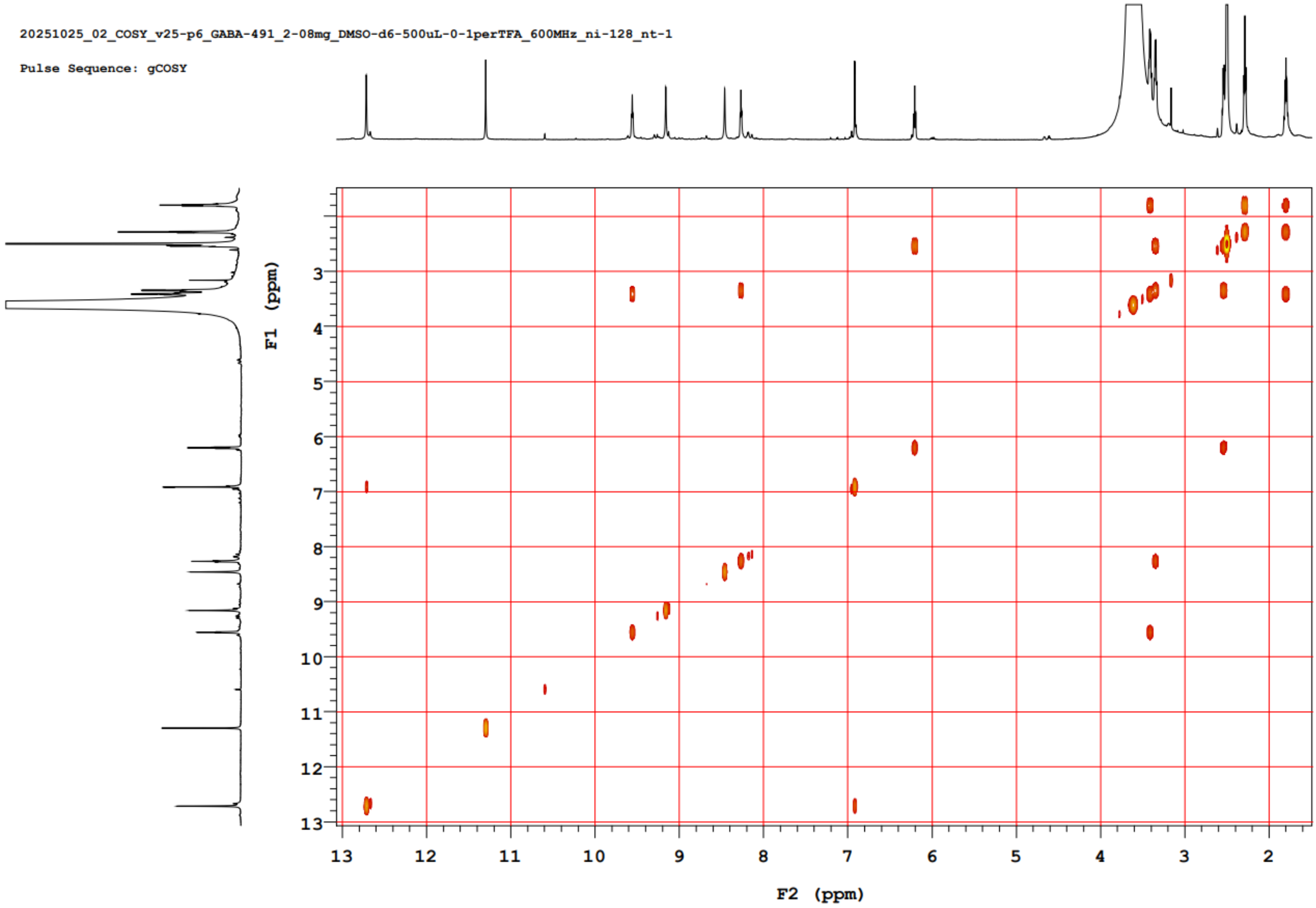

Figure S78. COSY spectrum of **12c** (2.08 mg) (600 MHz, DMSO-d_6: 500 μL - 0.1% TFA).

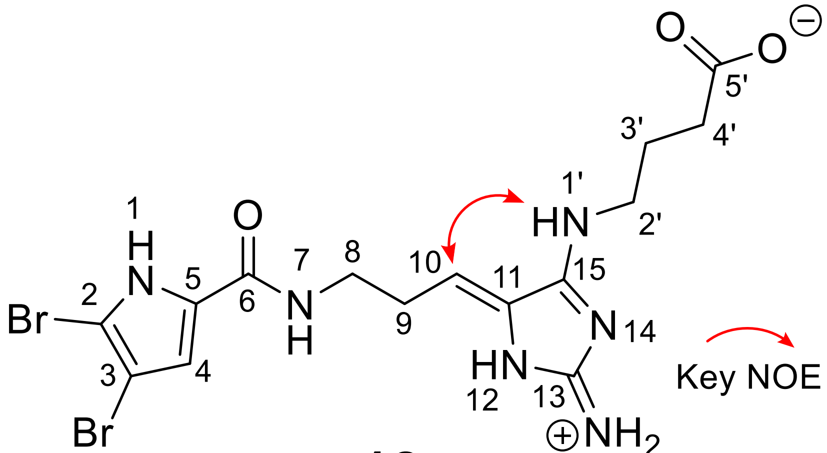

12c

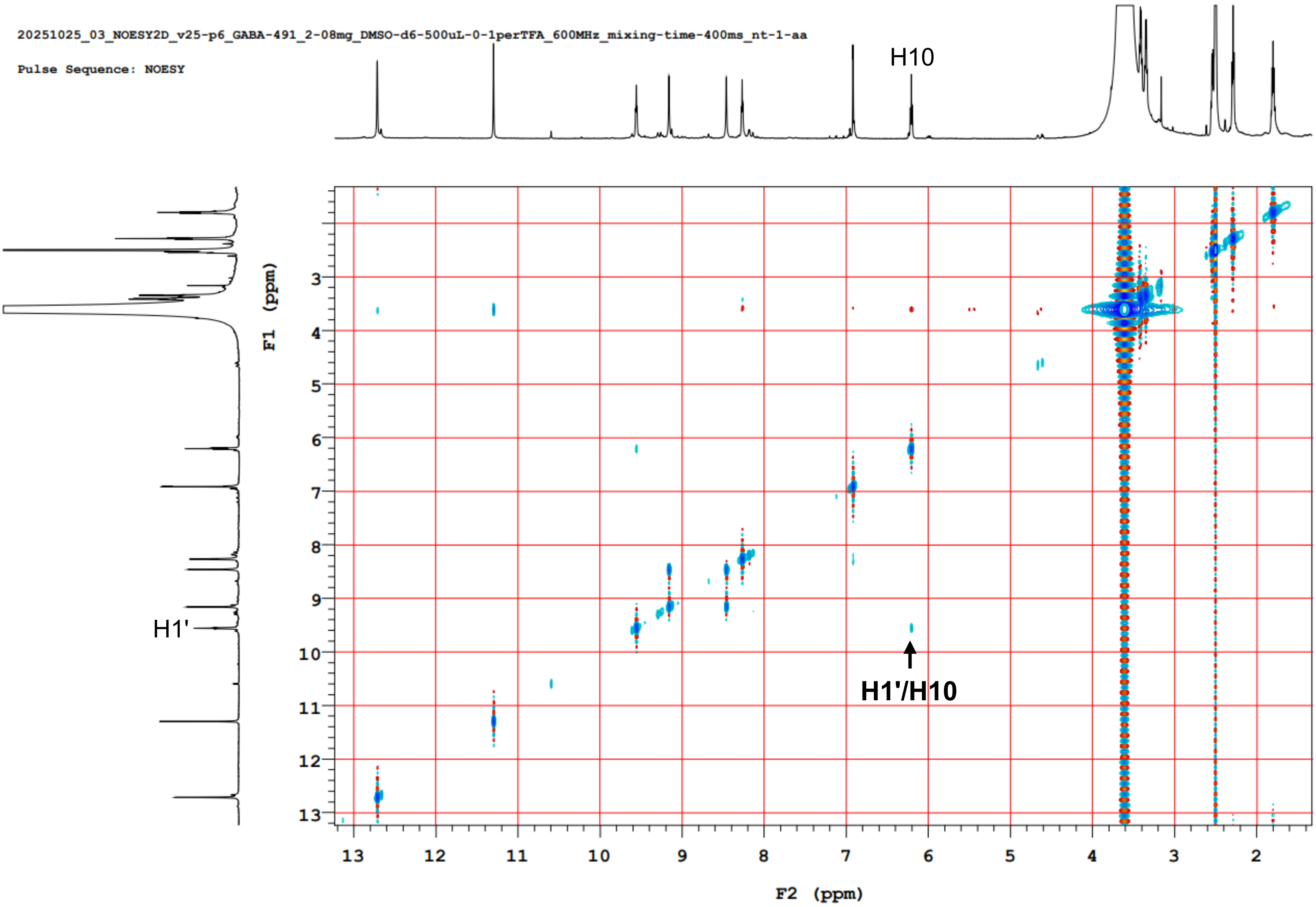

Figure S79. NOESY spectrum of **12c** (2.08 mg) (600 MHz, DMSO-d_6: 500 µL - 0.1% TFA).

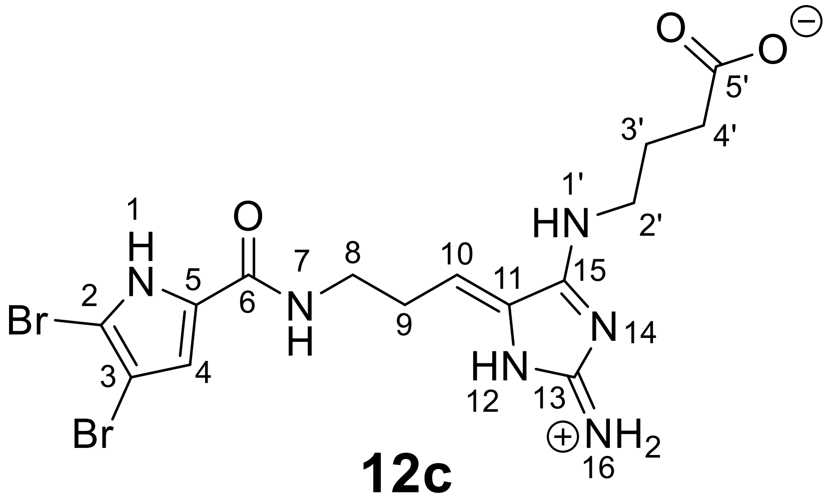

12c

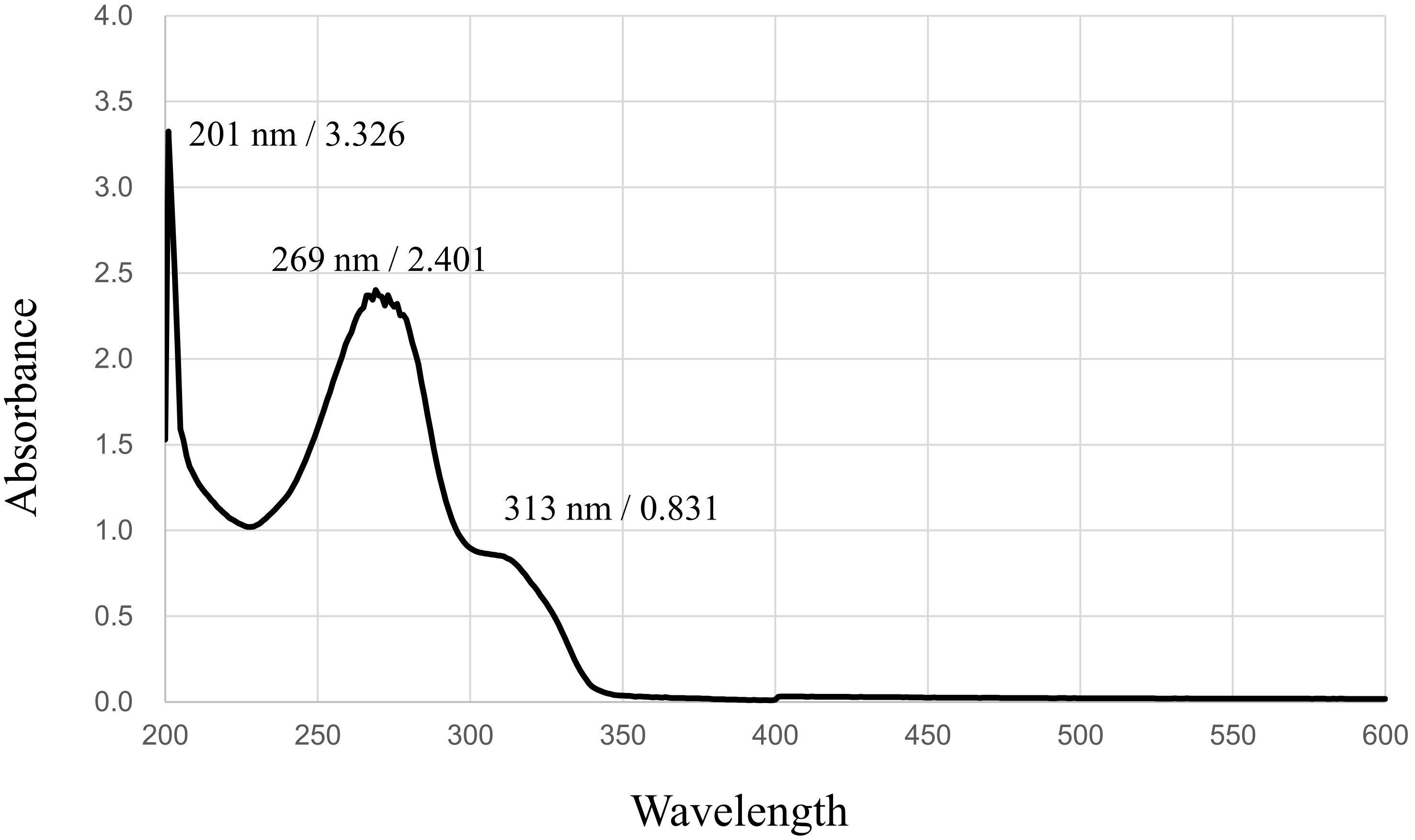

Figure S80. UV absorption spectrum of **12c** (MeOH). c = 1.28 x 10^{-4} (M)

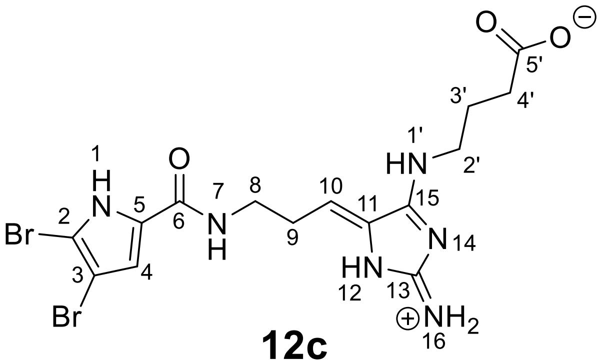

12c

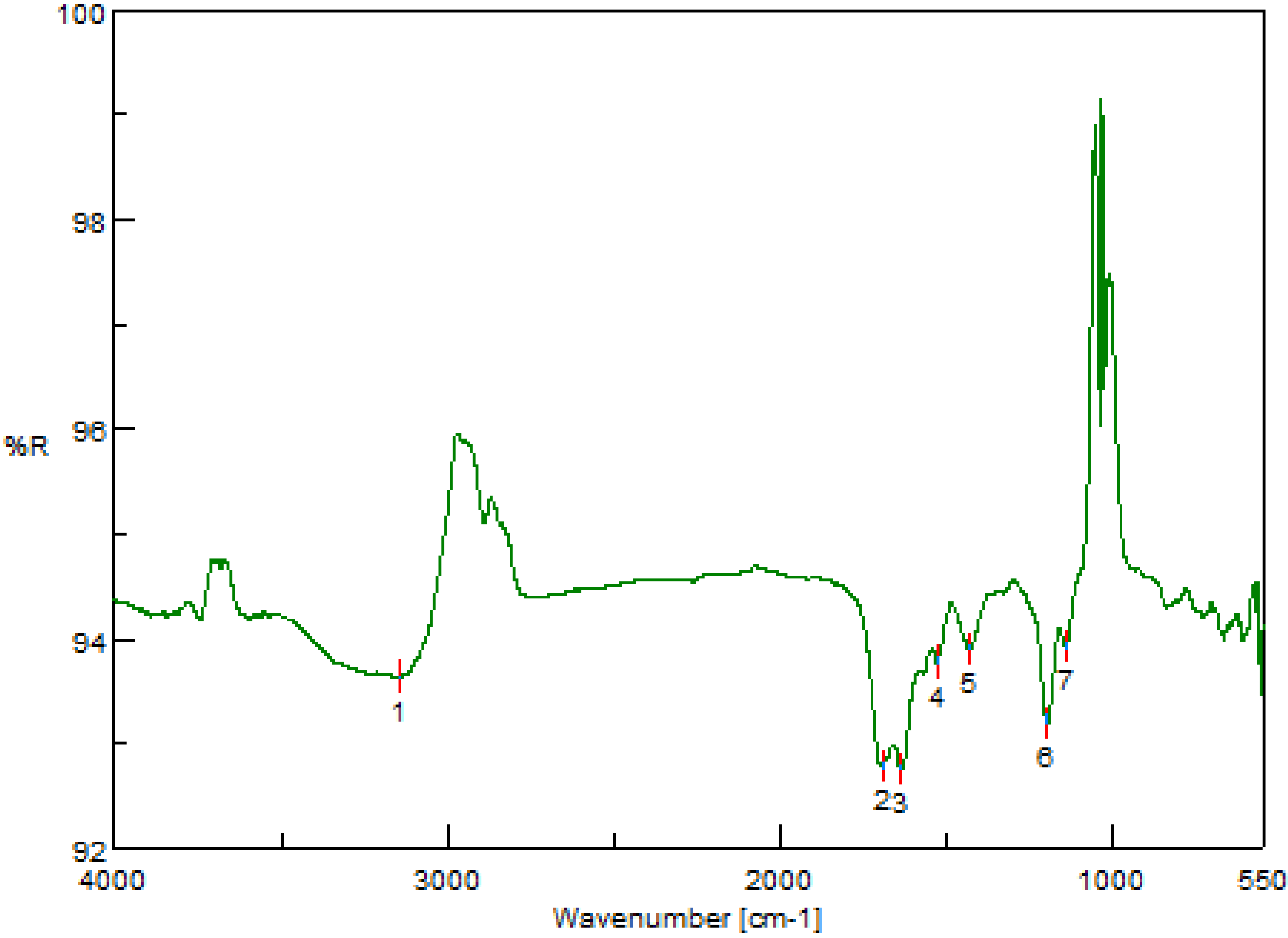

No.	Wavenumber	Strength
1	3147.3	93.6394
2	1694.2	92.7731
3	1636.3	92.7497
4	1530.2	93.7753
5	1428.0	93.8998
6	1197.6	93.1906
7	1140.7	93.9179

Figure S81. IR spectrum of **12c** (ATR).

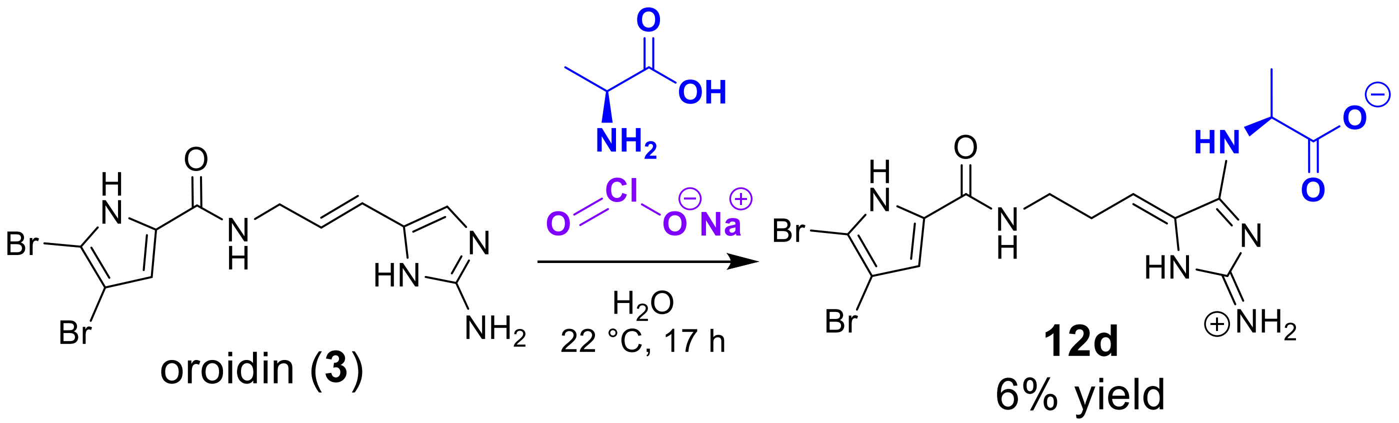

Scheme S4. Synthesis of **12d**.

Eight batches of oroidin (**3**) (HCOOH salt, 3.0 mg, 0.0069 mmol each; 24 mg, 0.055 mmol in total) were placed in 20 mL round-bottomed flasks, and H_2O (4.0 mL) was added to each flask with stirring. L-Alanine (0.89 g, 10 mmol, 1449 equiv.) was then added to the mixtures, followed by addition of $NaClO_2$ (120 mg, 1.33 mmol, 193 equiv.). The flasks were sealed with septa caps, and the reaction mixtures were stirred at 22 °C for 17 h. After completion, the mixtures were combined and filtered through a small pad of Celite, rinsing the flasks and filter cake with H_2O. The filtrate was directly purified by ODS silica gel column chromatography (MeOH/H_2O, 0:100 to 100:0, v/v). The eluate was concentrated under reduced pressure, and the crude material was filtered through a Cosmospin filter H (0.45 μm). Further purification was performed by RP-HPLC (InertSustain AQ-C18, 5 μm, 10 mm i.d. × 250 mm; GL Science) using gradient elution (0–4 min, MeOH/H_2O/HCOOH = 35:65:0.1 to 50:50:0.1, v/v; 4 min-, 50:50:0.1) at a flow rate of 2.0 mL/min. Semi-pure **12d** was obtained at 24-32 min (1.52 mg, 0.0032 mmol, 6% yield) as a yellow film.

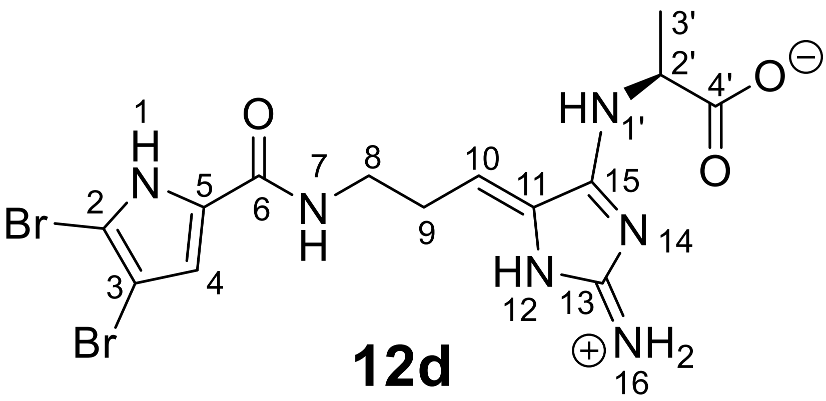

12d

12d:

$\mathbf{R_f}$ = 0.18 ($CHCl_3$/MeOH/28% NH_3 aq. = 50:50:1, v/v/v; UV).

$[\alpha]_D^{25}$: +17.9 (c = 7.6 x 10^{-4}, MeOH).

UV/vis λ_{max} (MeOH) nm (log ε): 310 (3.40), 274 (3.93), 200 (4.44).

^{1}H NMR (600 MHz, CD_3OD containing 0.1% TFA): δ 6.78 (s, C4-H, 1H), 6.30 (t, J = 7.8 Hz, C10-H, 1H), 4.67 (q, J = 7.2 Hz, C2'-H, 1H), 3.48 (t, J = 6.9 Hz, C8-H, 2H), 2.62 (q, J = 7.0 Hz, C9-H, 2H), 1.54 (d, J = 7.8 Hz, C3'-H, 3H).

^{1}H NMR (600 MHz, DMSO-d_6 containing 0.1% TFA): δ 12.72 (d, J = 1.8 Hz, N1-H, 1H), 11.45 (s, N12-H, 1H), 9.73 (d, J = 7.2 Hz, N1'-H, 1H), 9.26 (s, N16-H, 1H), 8.57 (s, N16-H, 1H), 8.29 (t, J = 5.7 Hz, N7-H, 1H), 6.93 (d, J = 3.0 Hz, C4-H, 1H), 6.40 (t, J = 7.8 Hz, C10-H, 1H), 4.46 (quintet, J = 7.4 Hz, C2'-H, 1H), 3.36 (q, J = 7.0 Hz, C8-H, 2H), 2.58 (q, J = 7.0 Hz, C9-H, 2H), 1.45 (d, J = 7.8 Hz, C3'-H, 3H).

^{13}C NMR (151 MHz, CD_3OD containing 0.1% TFA): δ 175.3 (C4'), 170.1 (C15), 168.7 (C13), 162.6 (C6), 135.6 (C11), 129.4 (C5), 117.6 (C10), 115.0 (C4), 107.2 (C2), 100.8 (C3), 53.7 (C2'), 39.9 (C8), 30.2 (C9), 17.9 (C3').

HRMS (**ESI**): (*m/z*) calcd for $C_{14}H_{17}{}^{79}Br_2N_6O_3^+$ $[M+H]^+$: 474.9723, found 474.9697.

IR ν_{max}: 3740 (w), 3680 (w), 3193 (br), 2945 (m), 2869 (m), 1692 (m), 1606 (s), 1531 (m), 1462 (m), 1406 (m), 1350 (m), 1236 (w), 1135 (w), 1052 (s), 1033 (s), 1019 (m).

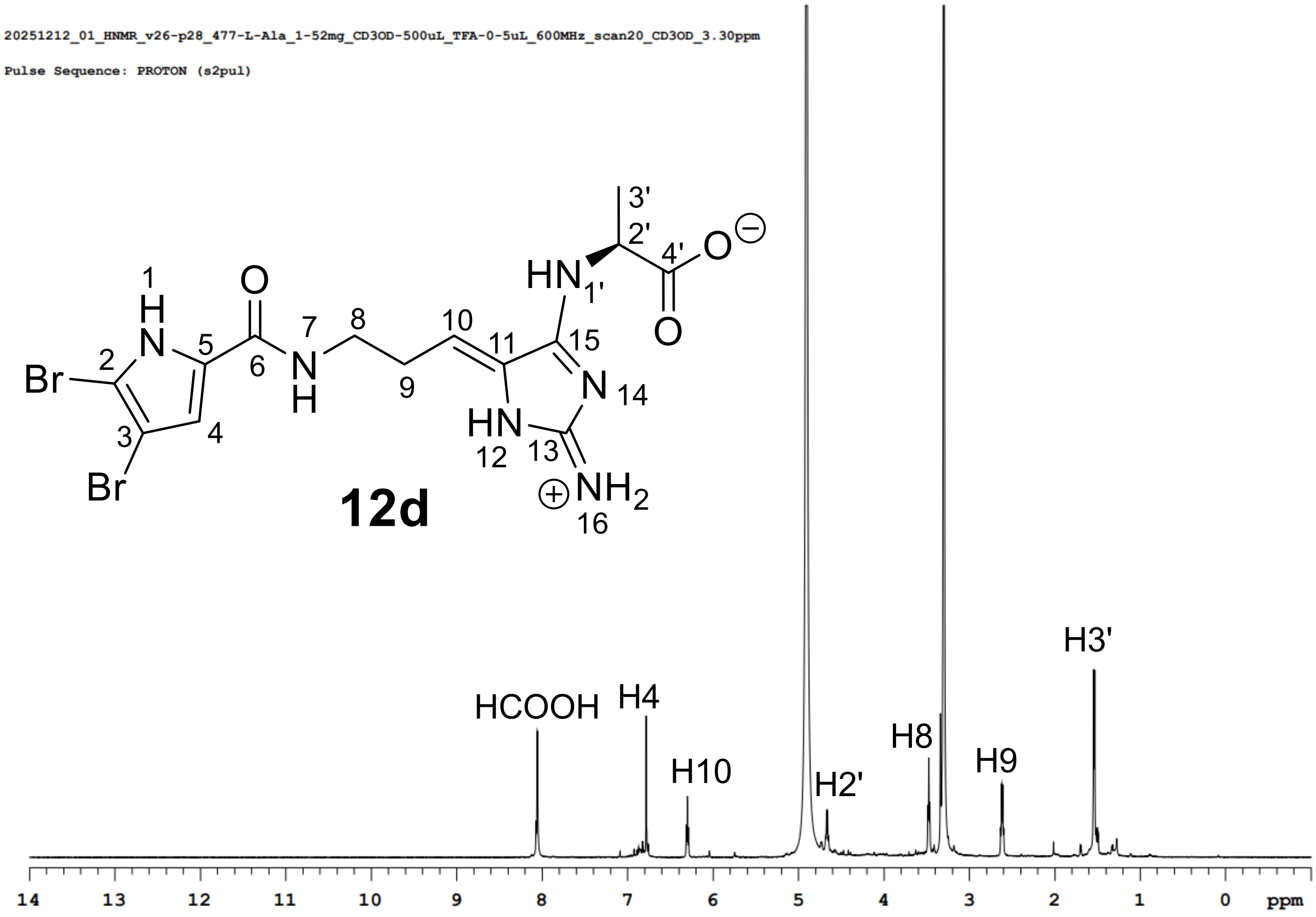

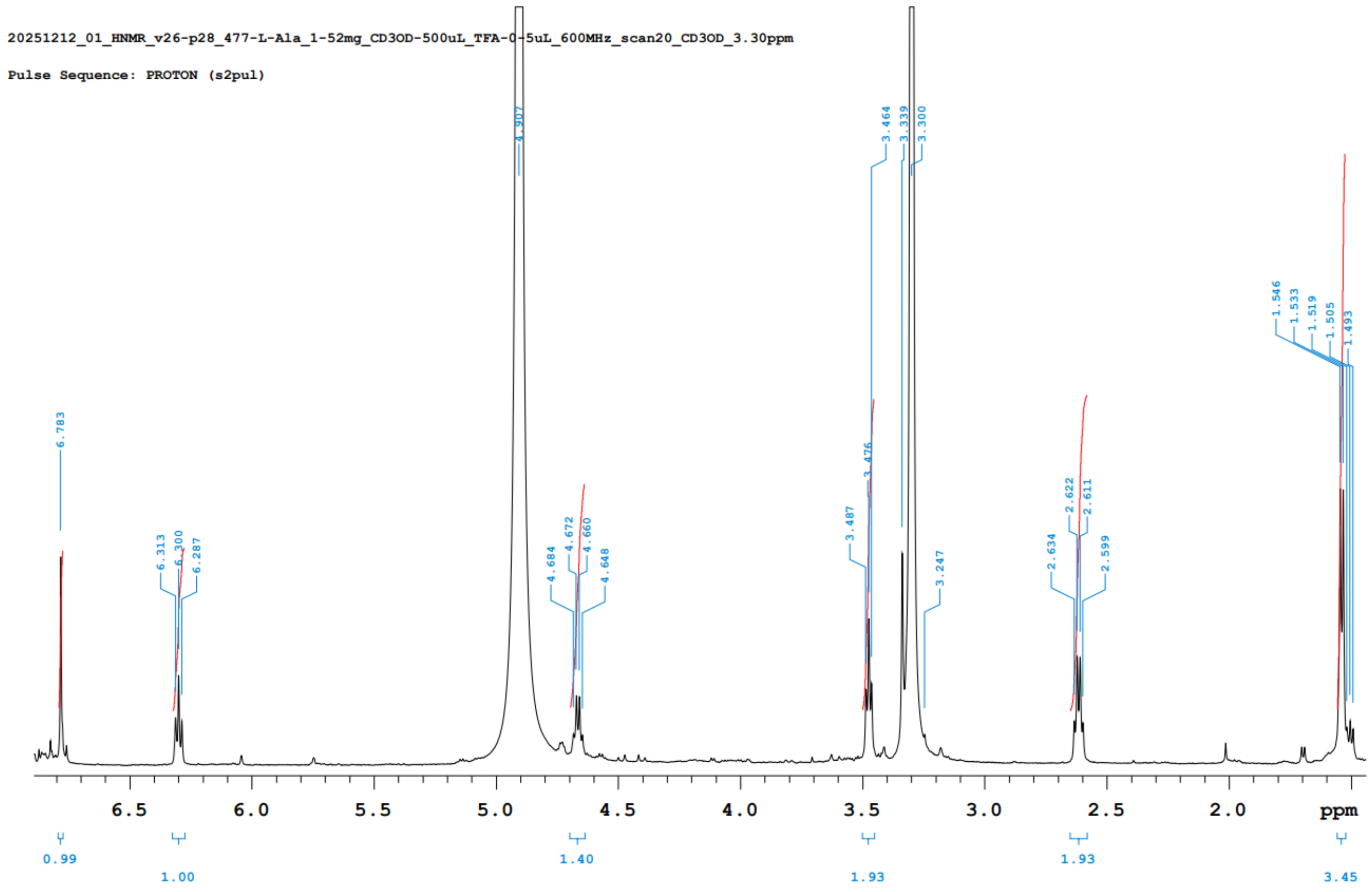

Figure S82. ^{1}H NMR spectrum of **12d** (1.52 mg) (600 MHz, CD_3OD: 500 µL - 0.1% TFA).

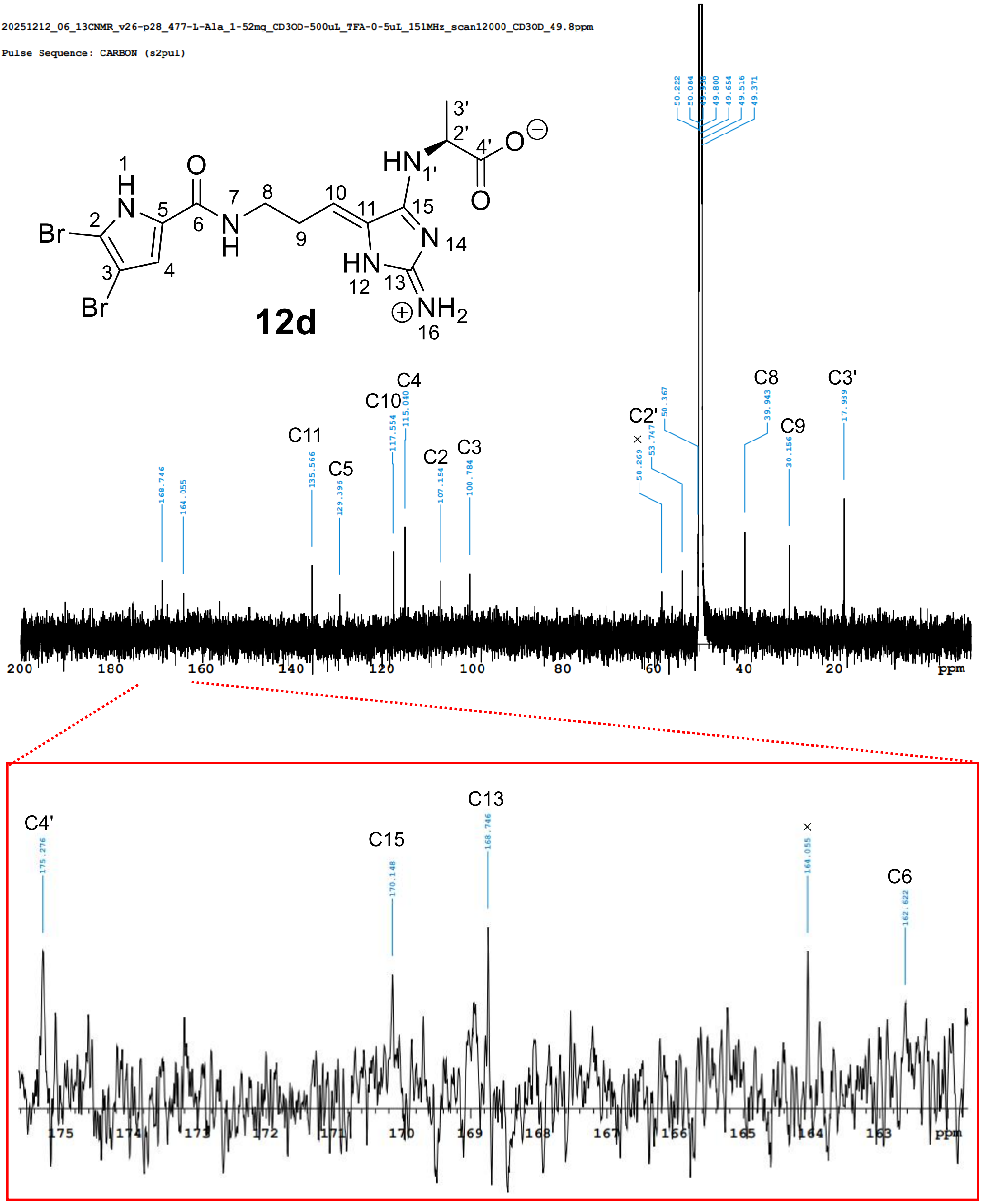

Figure S83. ^{13}C NMR spectrum of **12d** (1.52 mg) (151 MHz, CD_3OD: 500 µL - 0.1% TFA).

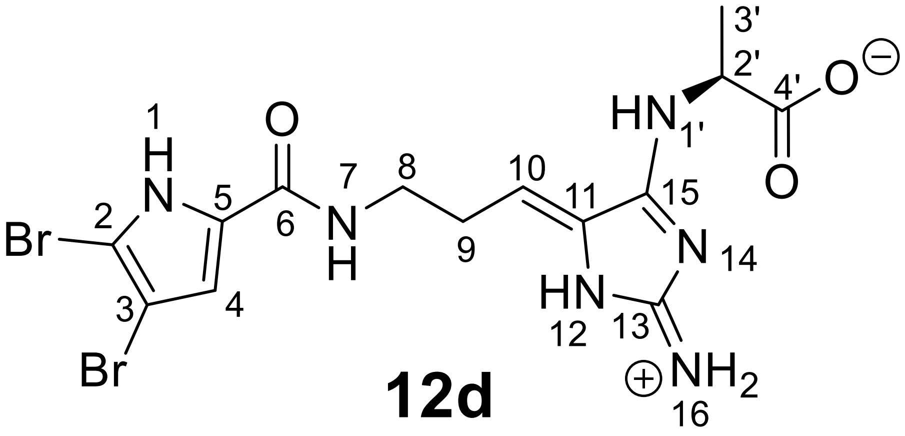

12d

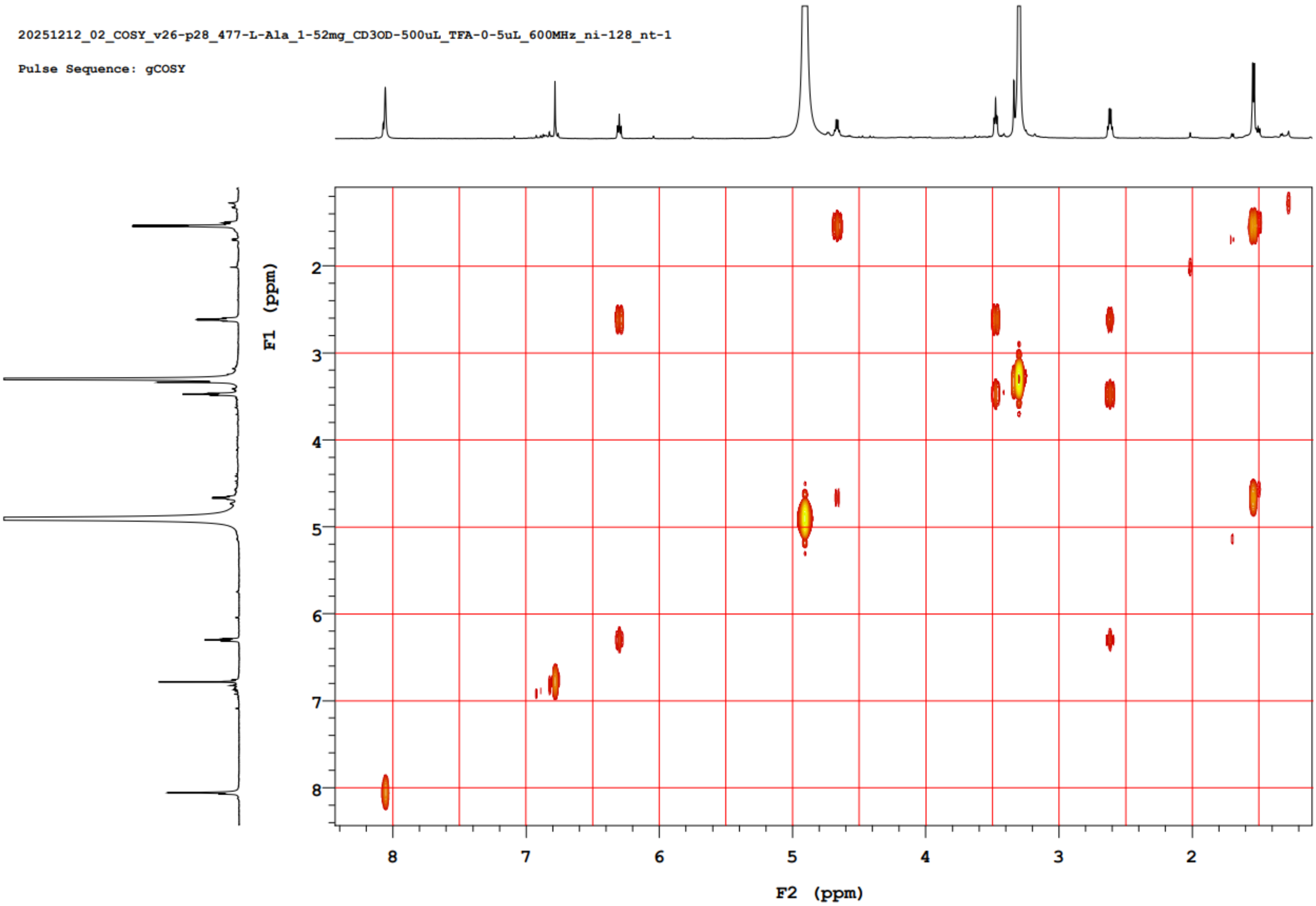

Figure S84. COSY spectrum of **12d** (1.52 mg) (600 MHz, CD_3OD: 500 µL - 0.1% TFA).

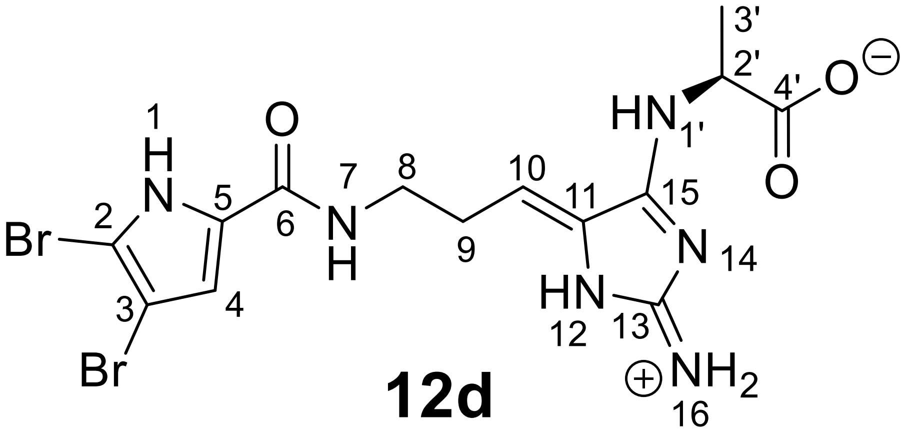

12d

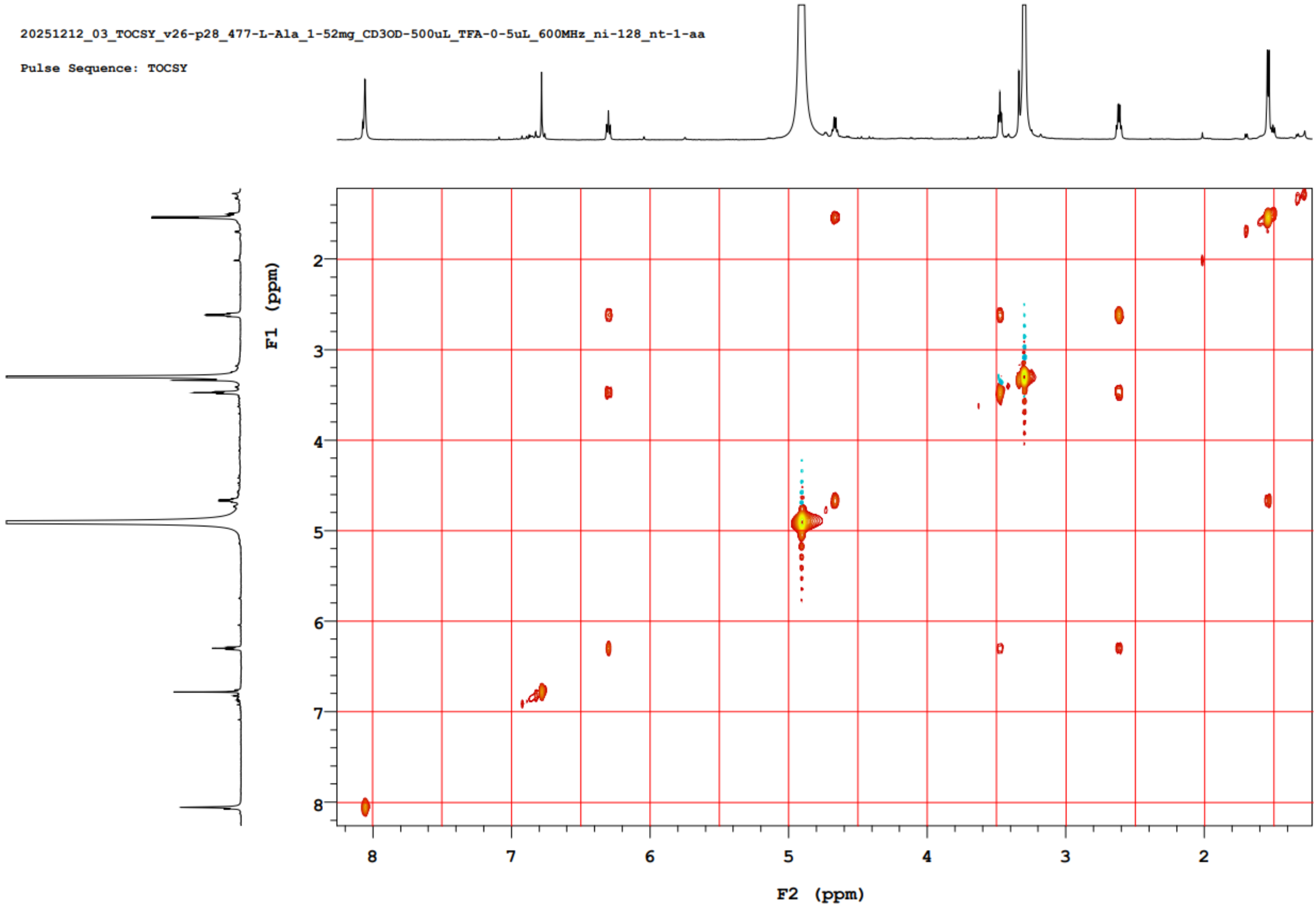

Figure S85. TOCSY spectrum of **12d** (1.52 mg) (600 MHz, CD_3OD: 500 µL - 0.1% TFA).

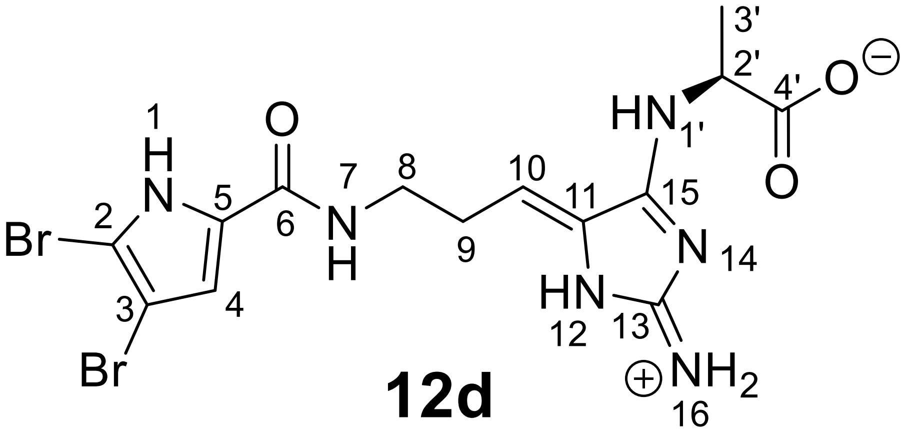

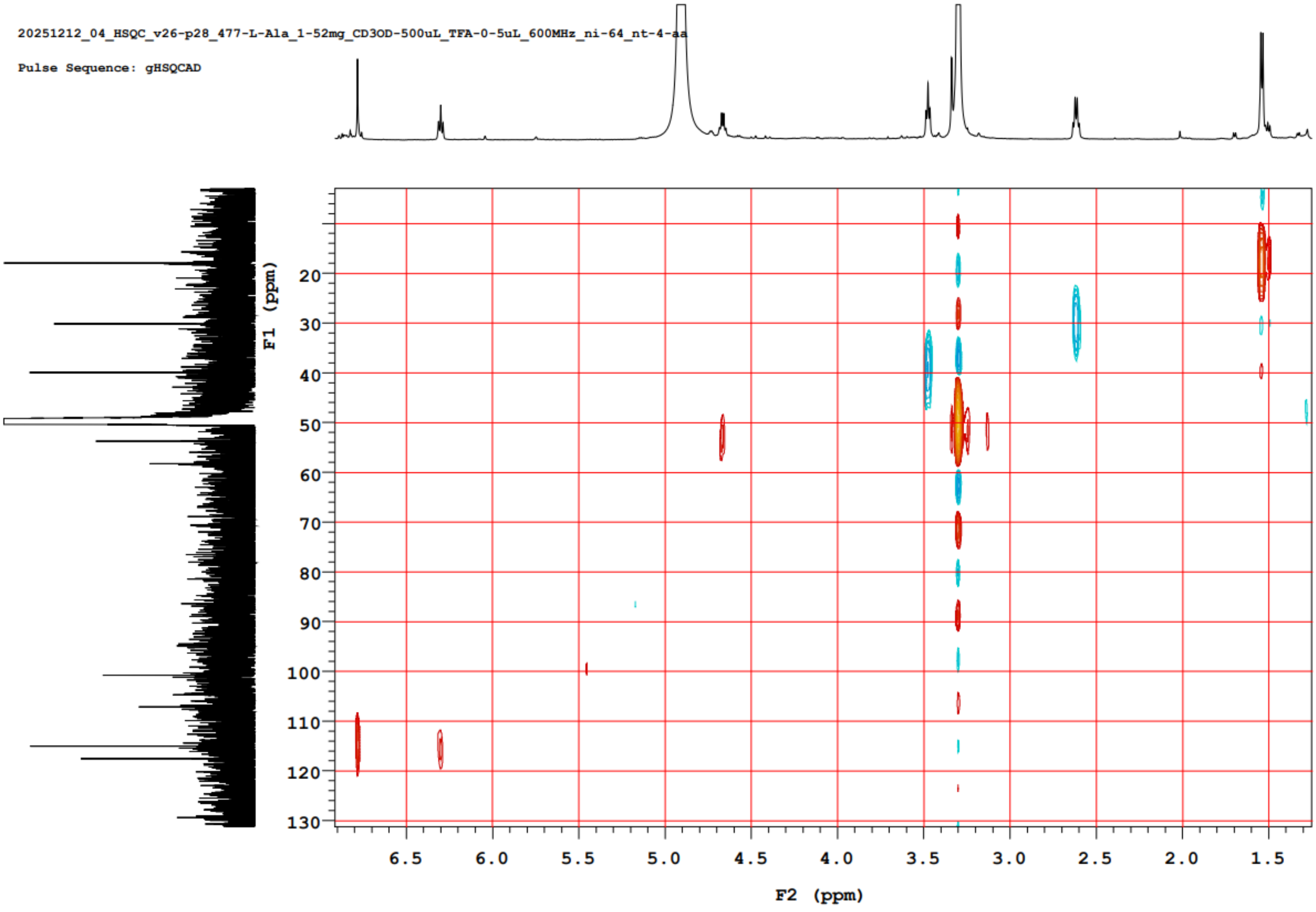

Figure S86. ^{1}H-^{13}C HSQC spectrum of **12d** (1.52 mg) (600 MHz / 151 MHz, CD_3OD: 500 μL - 0.1% TFA).

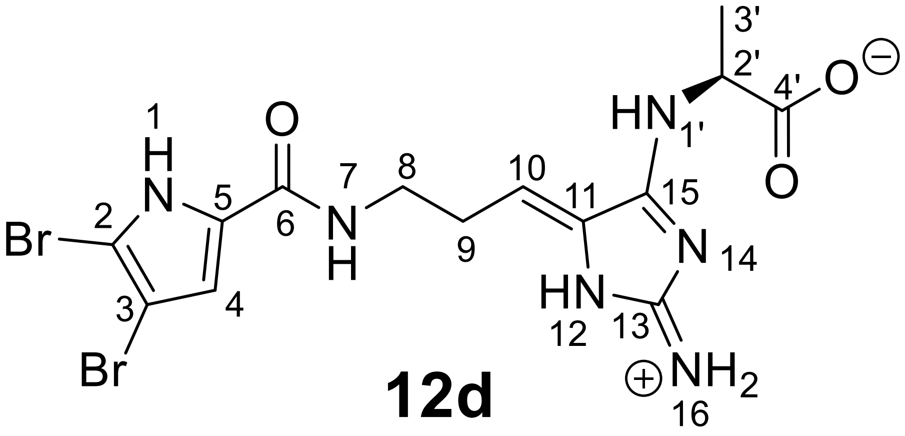

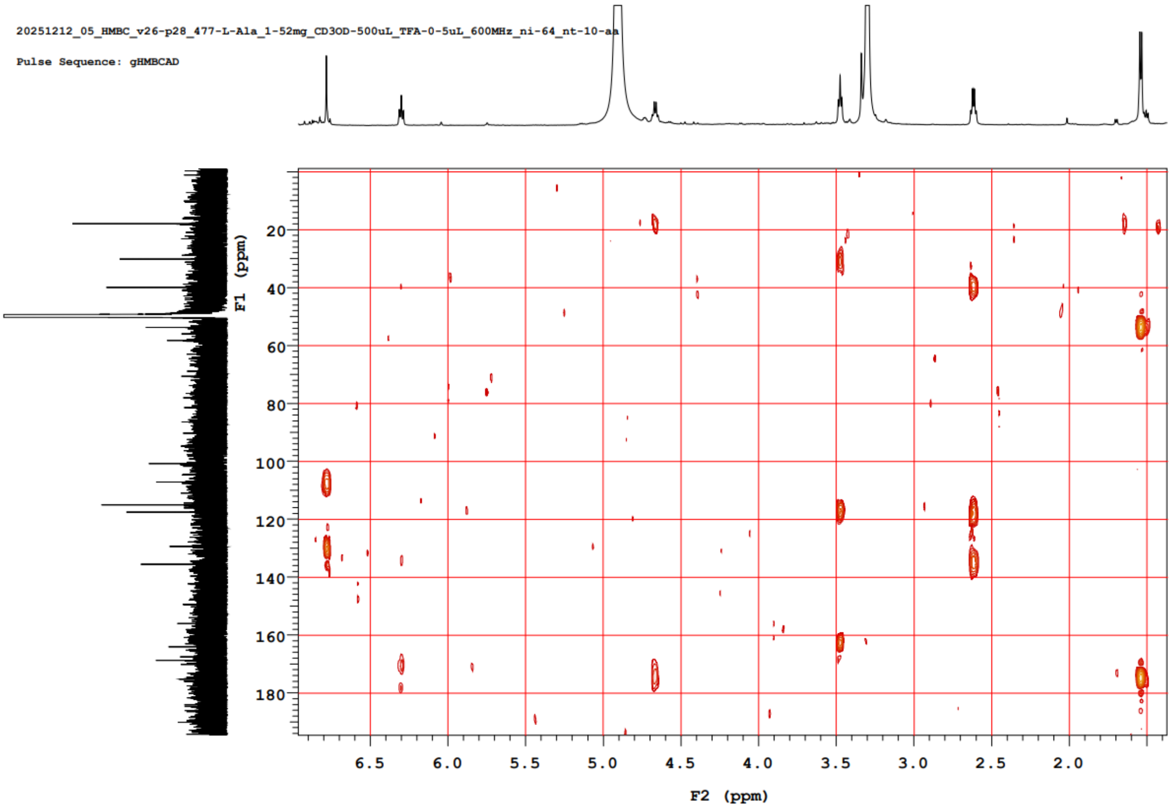

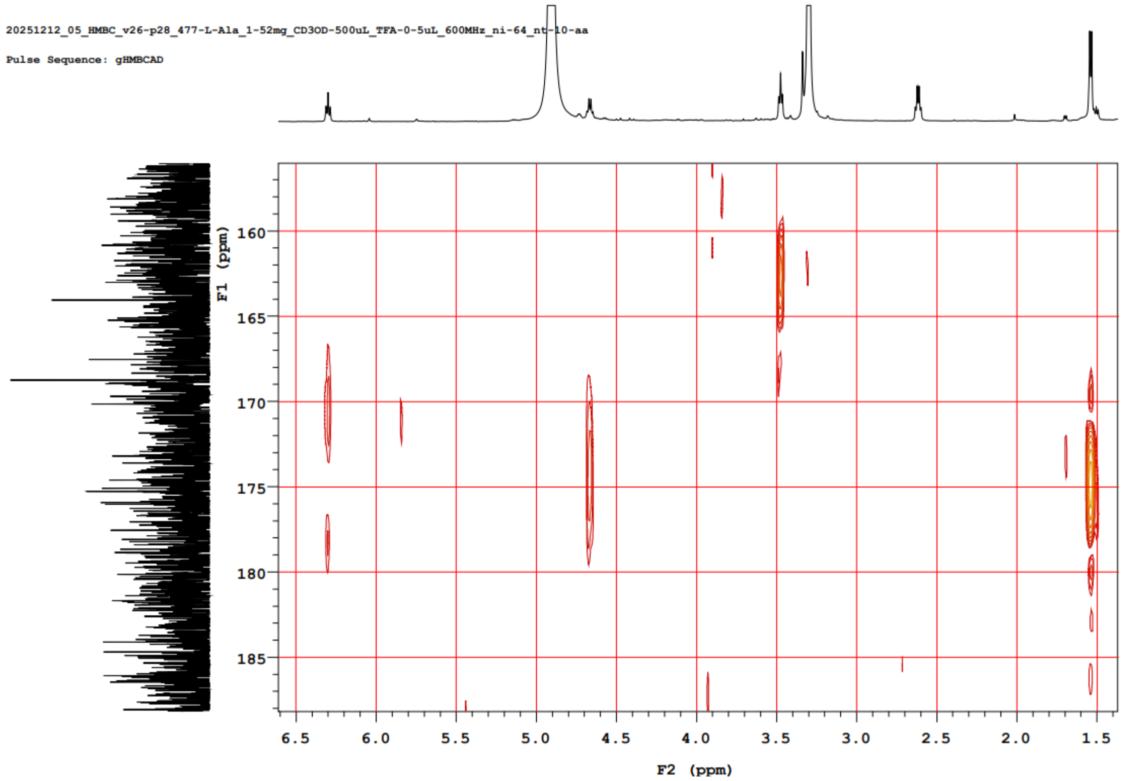

Figure S87. ^{1}H-^{13}C HMBC spectrum of **12d** (1.52 mg) (600 MHz / 151 MHz, CD_3OD: 500 µL - 0.1% TFA).

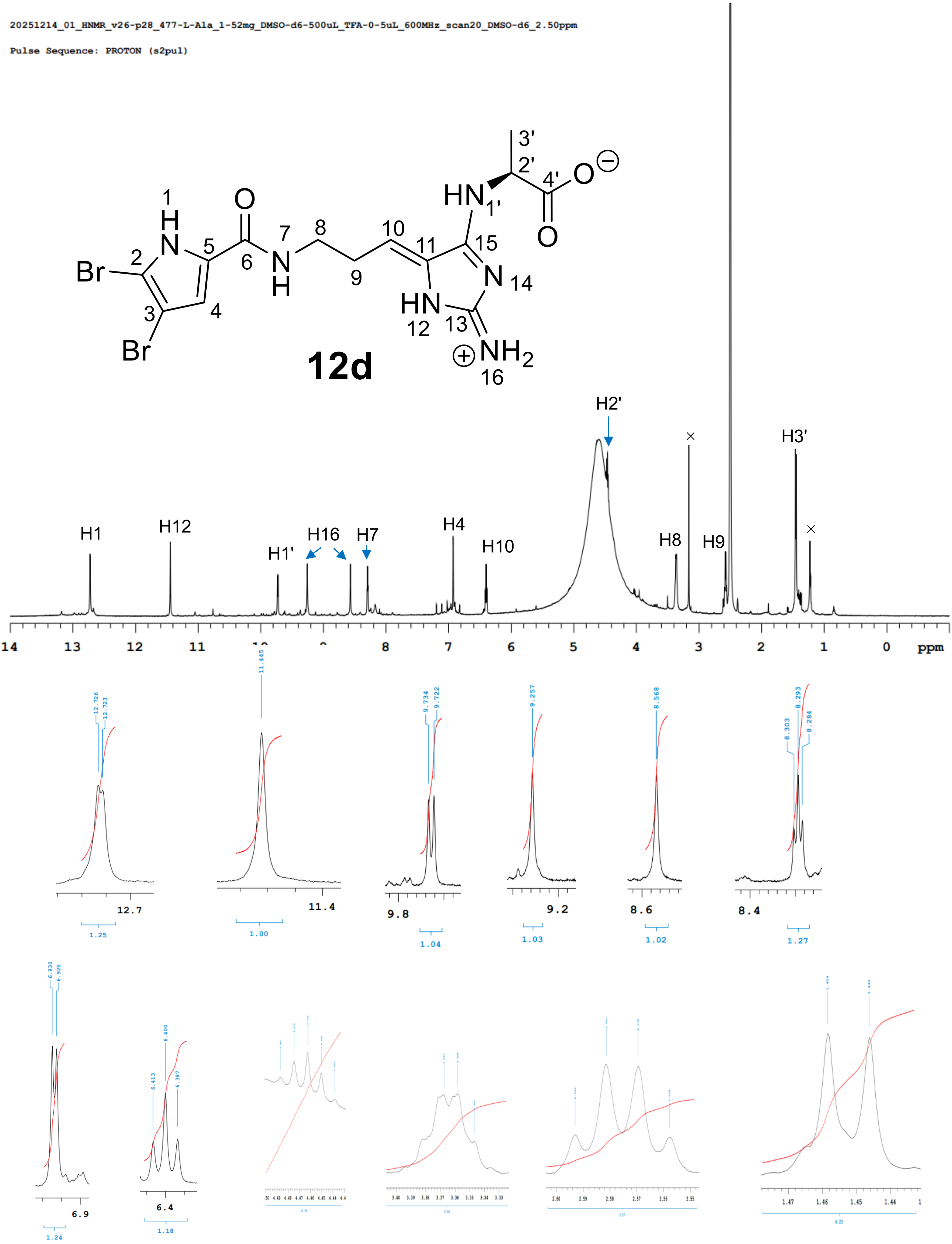

Figure S88. ^{1}H NMR spectrum of **12d** (1.52 mg) (600 MHz, DMSO-d_6: 500 μL - 0.1% TFA).

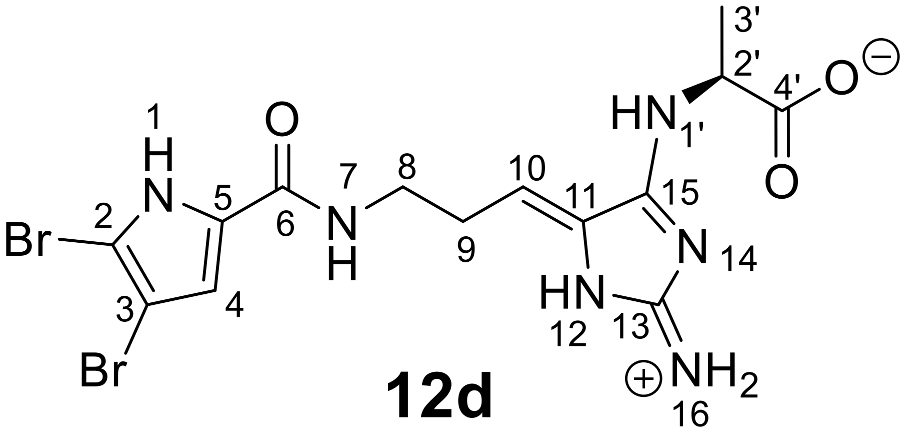

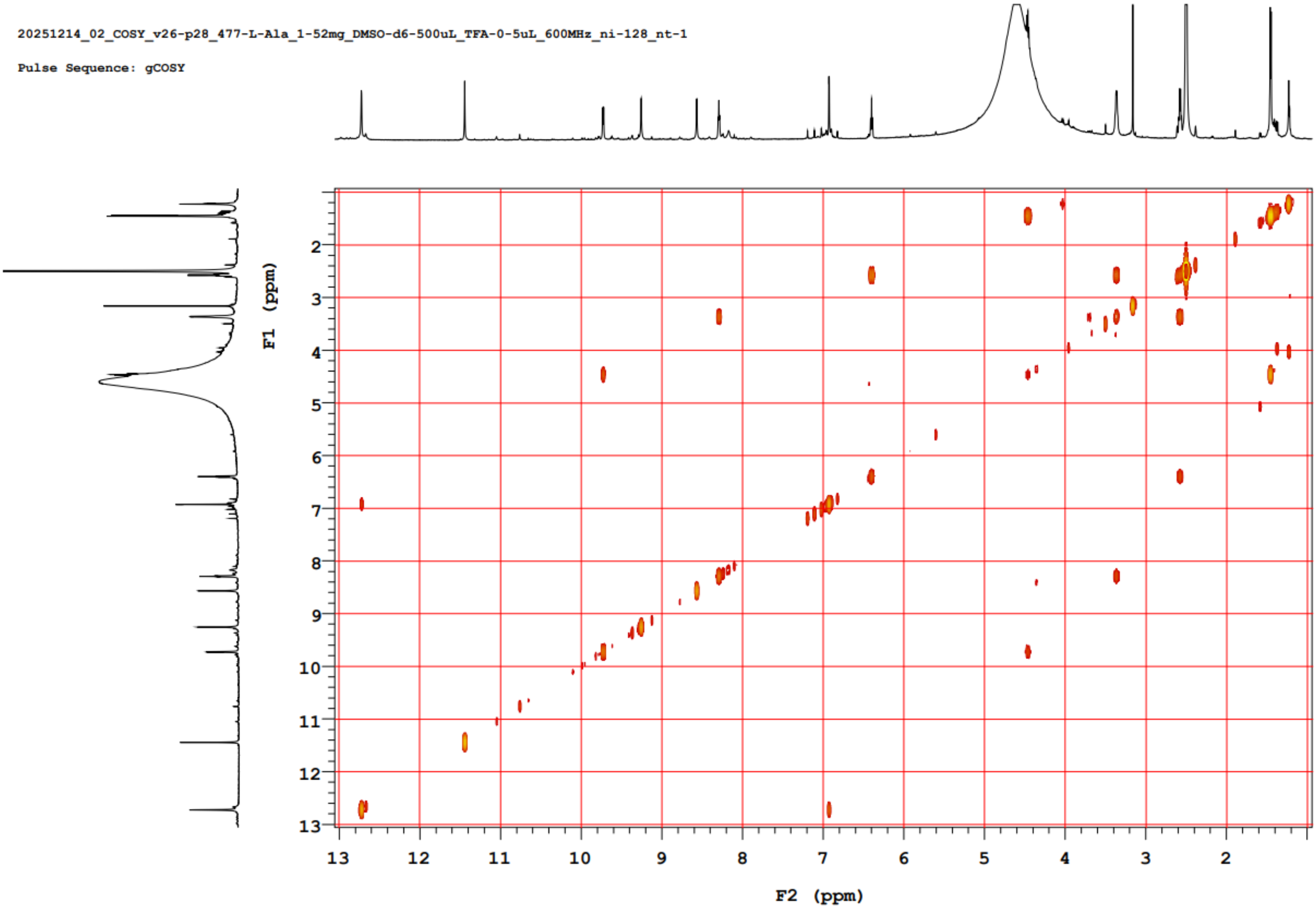

Figure S89. COSY spectrum of **12d** (1.52 mg) (600 MHz, DMSO-d_6: 500 µL - 0.1% TFA).

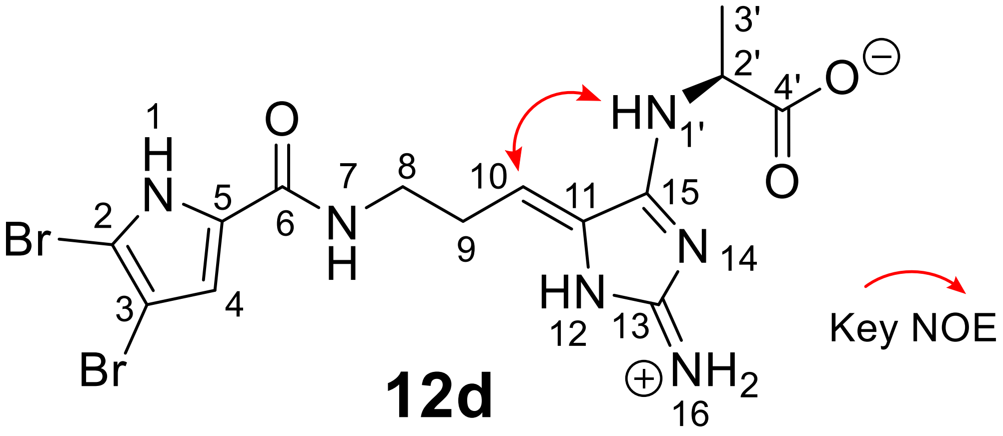

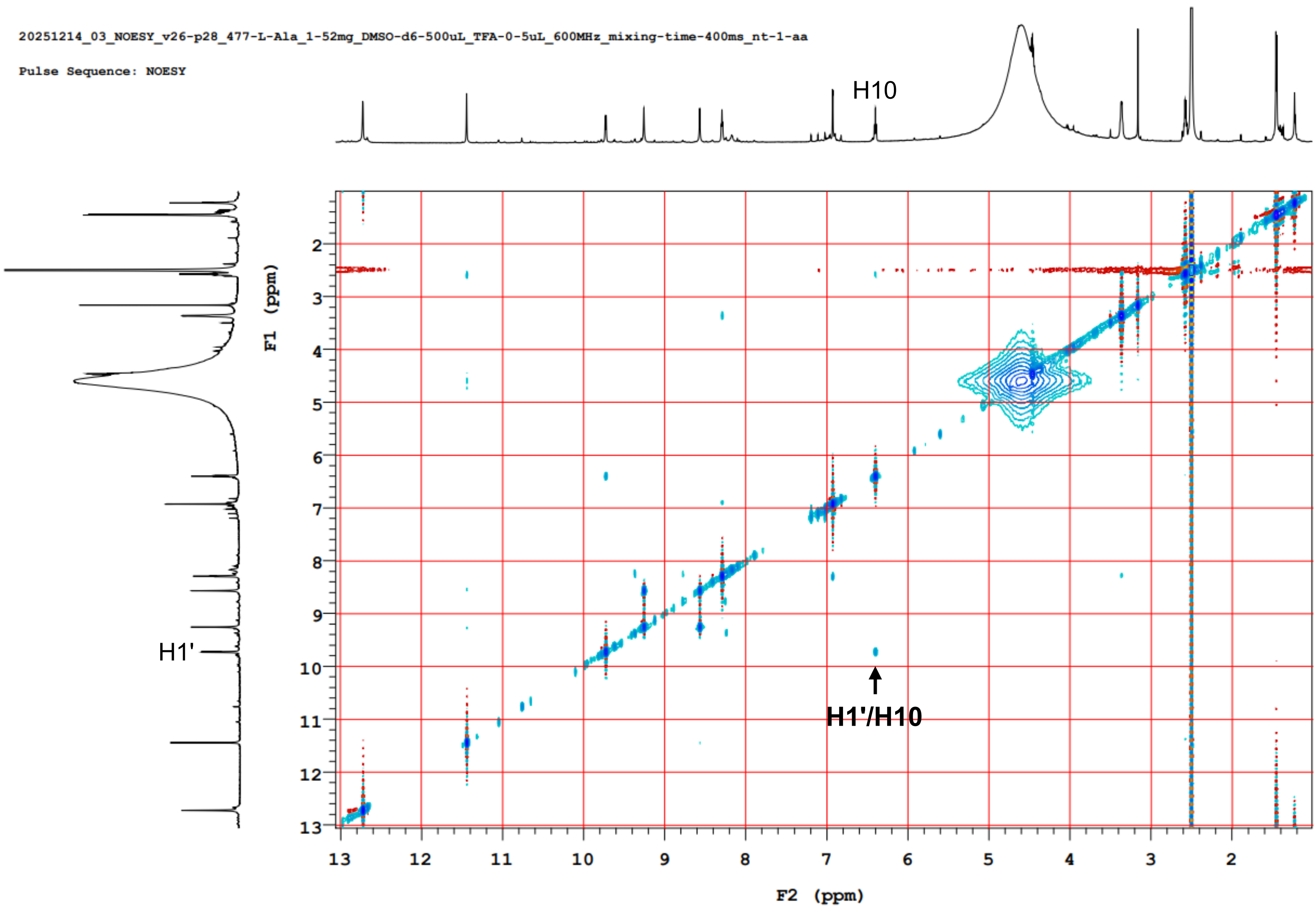

Figure S90. NOESY spectrum of **12d** (1.52 mg) (600 MHz, DMSO-d_6: 500 µL - 0.1% TFA).

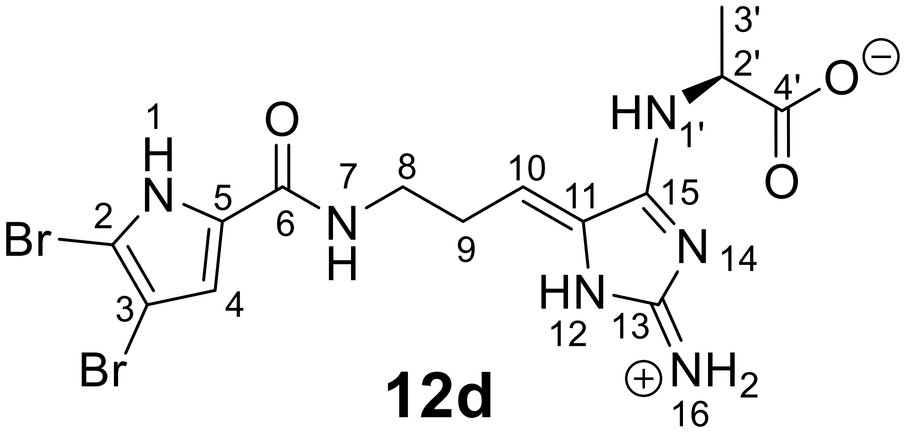

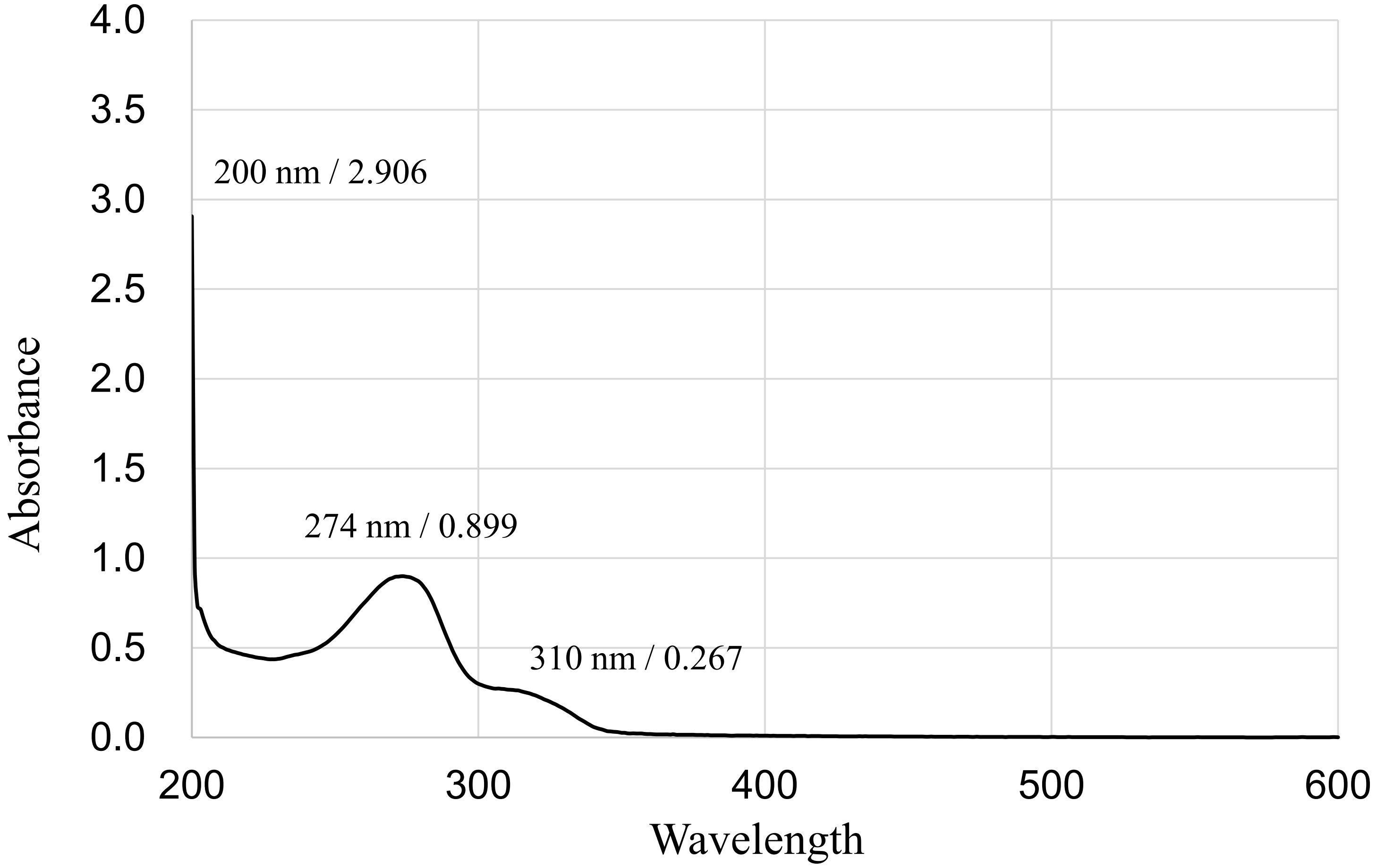

Figure S91. UV absorption spectrum of **12d** (MeOH). c = 1.05 x 10^{-4} (M)

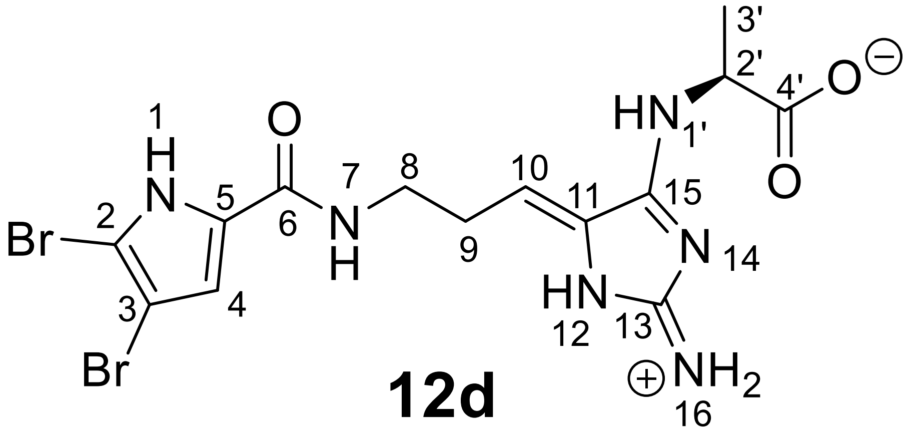

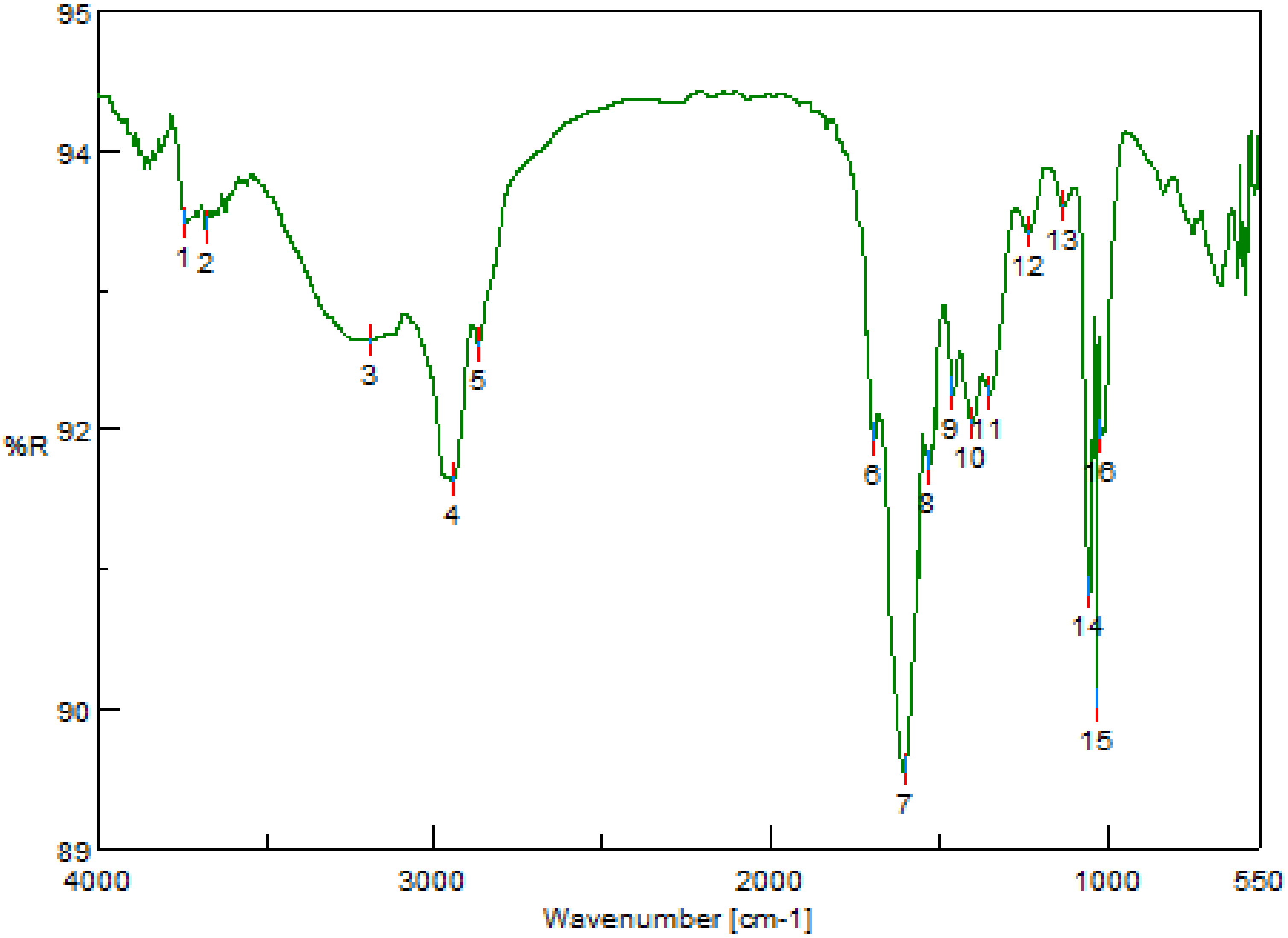

No.	Wavenumber	Strength	No.	Wavenumber	Strength
1	3740.2	93.4728	9	1461.8	92.2565
2	3679.5	93.4431	10	1405.9	92.0395
3	3192.6	92.6287	11	1349.9	92.2527
4	2945.7	91.6350	12	1236.2	93.4086
5	2868.6	92.5986	13	1134.9	93.5944
6	1692.2	91.9229	14	1052.9	90.8279
7	1605.5	89.5570	15	1032.7	90.0247
8	1531.2	91.7173	16	1019.2	91.9420

Figure S92. IR spectrum of **12d** (ATR).

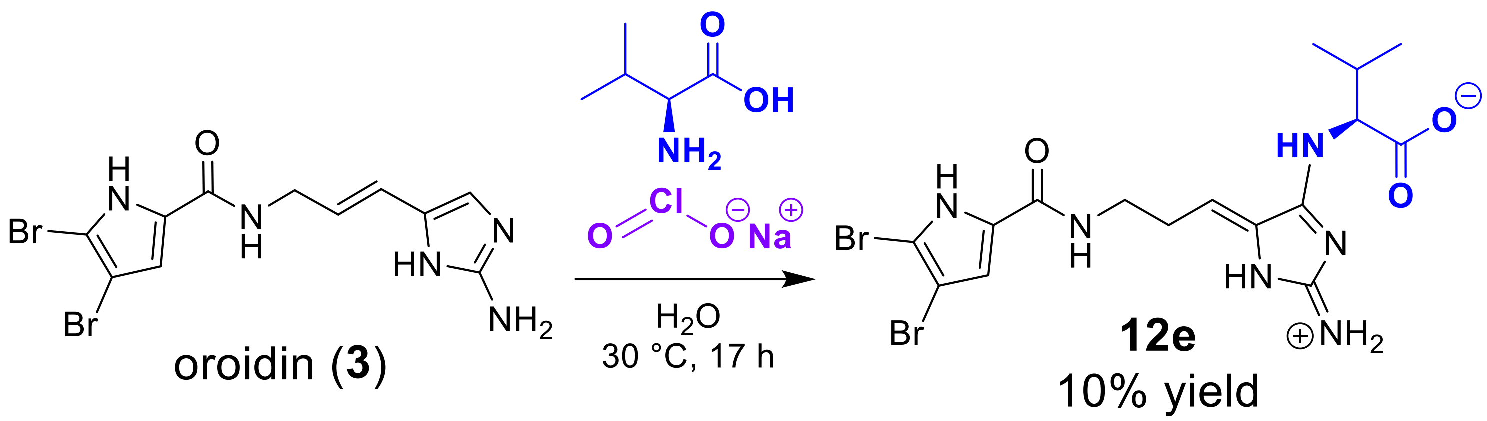

Scheme S5. Synthesis of **12e**.

Five batches of oroidin (**3**) (HCOOH salt, 3.0 mg, 0.0069 mmol each; 15 mg, 0.034 mmol in total) were placed in 20 mL round-bottomed flasks, and H_2O (4.0 mL) was added to each flask with stirring. L-Valine (0.94 g, 8.0 mmol, 1159 equiv.) was then added to the mixtures, followed by addition of $NaClO_2$ (120 mg, 1.33 mmol, 193 equiv.). The flasks were sealed with septa caps, and the reaction mixtures were stirred at 30 °C for 17 h. After completion, the mixtures were combined and filtered through a small pad of Celite, rinsing the flasks and filter cake with H_2O. The filtrate was directly purified by ODS silica gel column chromatography (MeOH/H_2O, 30:70 to 100:0, v/v). The eluate was concentrated under reduced pressure, and the crude material was filtered through a Cosmospin filter H (0.45 μm). Further purification was performed by RP-HPLC (InertSustain AQ-C18, 5 μm, 10 mm i.d. × 250 mm; GL Science) using gradient elution (0–4 min, MeOH/H_2O/HCOOH = 3:97:0.1 to 60:40:0.1, v/v; 4 min–, 60:40:0.1) at a flow rate of 2.0 mL/min. Pure **12e** was obtained at 20-24 min (1.80 mg, 0.0036 mmol, 10% yield) as a slightly yellow solid.

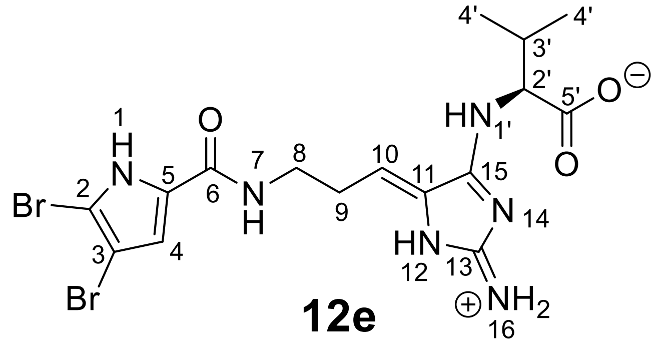

12e

12e:

$\mathbf{R_f}$ = 0.26 ($CHCl_3$/MeOH/28% NH_3 aq. = 60:40:2, v/v/v; UV).

$[\alpha]_D^{25}$: +5.2 (c = 8.7 x 10^{-4}, MeOH).

UV/vis λ_{max} (MeOH) nm (log ε): 310 (3.75), 270 (4.23), 200 (4.30).

^{1}H NMR (600 MHz, CD_3OD containing 0.1% TFA): δ 6.78 (s, C4-H, 1H), 6.47 (t, J = 8.1 Hz, C10-H, 1H), 4.56 (d, J = 6.6 Hz, C2'-H, 1H), 3.49 (m, C8-H, 2H), 2.63 (q, J = 7.2 Hz, C9-H, 2H), 2.29 (sextet, J = 6.7 Hz, C3'-H, 1H), 1.03 (dd, J = 8.4, 5.1 Hz, C4'-H, 6H).

^{1}H NMR (600 MHz, DMSO-d_6 containing 0.1% TFA): δ 12.73 (s, N1-H, 1H), 11.41 (s, N12-H, 1H), 9.55 (d, J = 8.4 Hz, N1'-H, 1H), 9.21 (s, N16-H, 1H), 8.52 (s, N16-H, 1H), 8.29 (t, J = 5.7 Hz, N7-H, 1H), 6.93 (d, J = 3.0 Hz, C4-H, 1H), 6.63 (t, J = 7.8 Hz, C10-H, 1H), 4.33 (t, J = 7.5 Hz, C2'-H, 1H), 3.37 (q, J = 6.4 Hz, C8-H, 2H), 2.58 (q, J = 7.2 Hz, C9-H, 2H), 2.21 (m, C3'-H, 1H), 0.97 (t, J = 7.8 Hz, C4'-H, 6H).

^{13}C NMR (151 MHz, CD_3OD containing 0.1% TFA): δ 174.0 (C5'), 170.8 (C15), 168.7 (C13), 162.8 (C6), 135.5 (C11), 129.4 (C5), 118.0 (C10), 115.0 (C4), 107.2 (C2), 100.8 (C3), 64.1 (C2'), 40.0 (C8), 32.6 (C3'), 30.2 (C9), 20.3 (C4'), 19.7 (C4').

HRMS (**ESI**): (m/z) calcd for $C_{16}H_{21}{}^{79}Br_2N_6O_3^+$ $[M+H]^+$: 503.0036, found 503.0031.

IR ν_{max}: 3144 (br), 1691 (s), 1627 (s), 1529 (w), 1440 (w), 1329 (w), 1199 (s), 1144 (m).

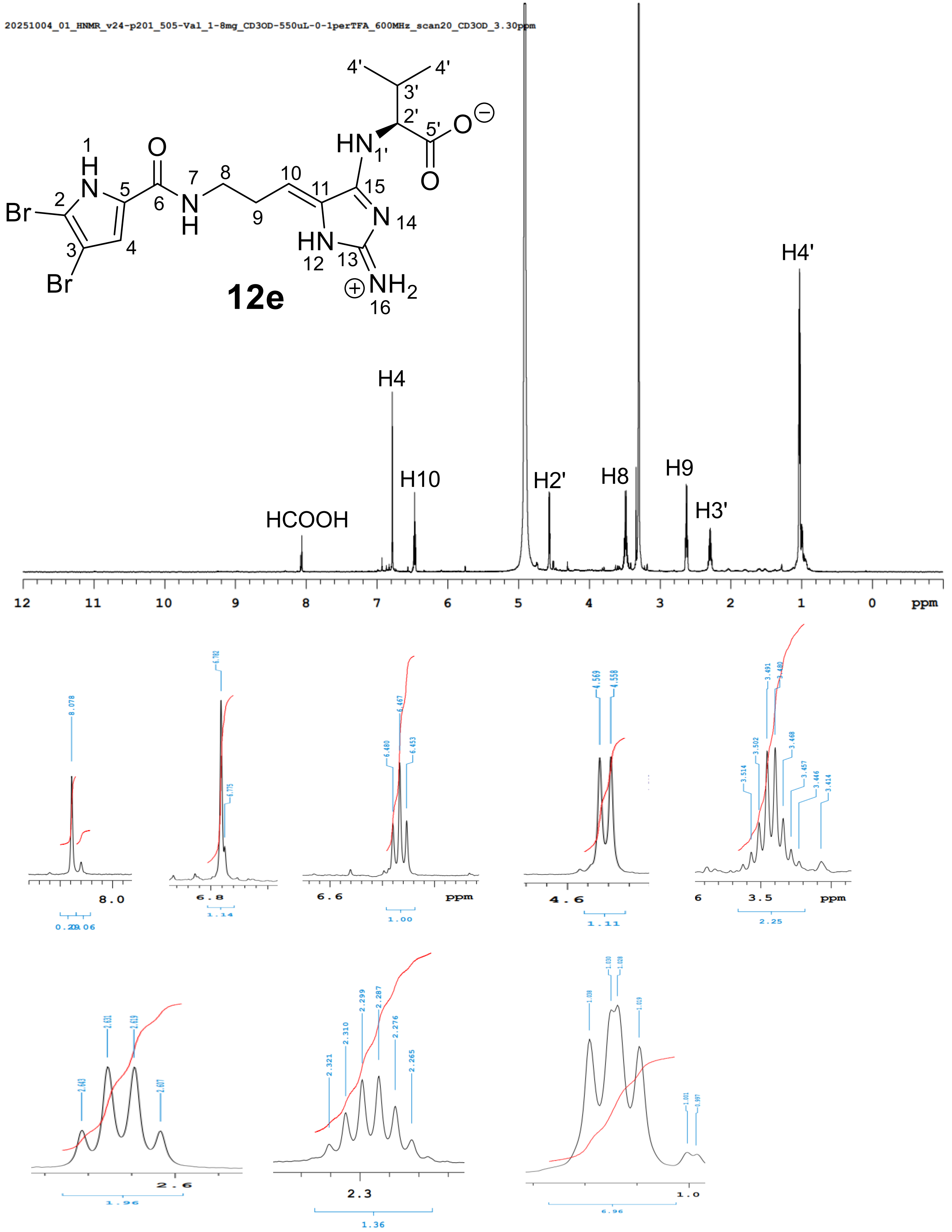

Figure S93. ^{1}H NMR spectrum of **12e** (1.80 mg) (600 MHz, CD_3OD: 550 µL - 0.1% TFA).

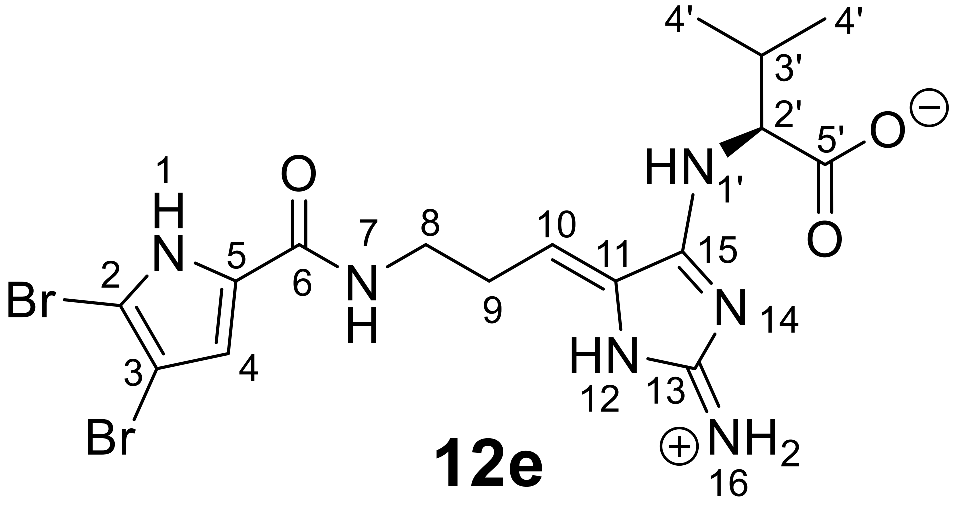

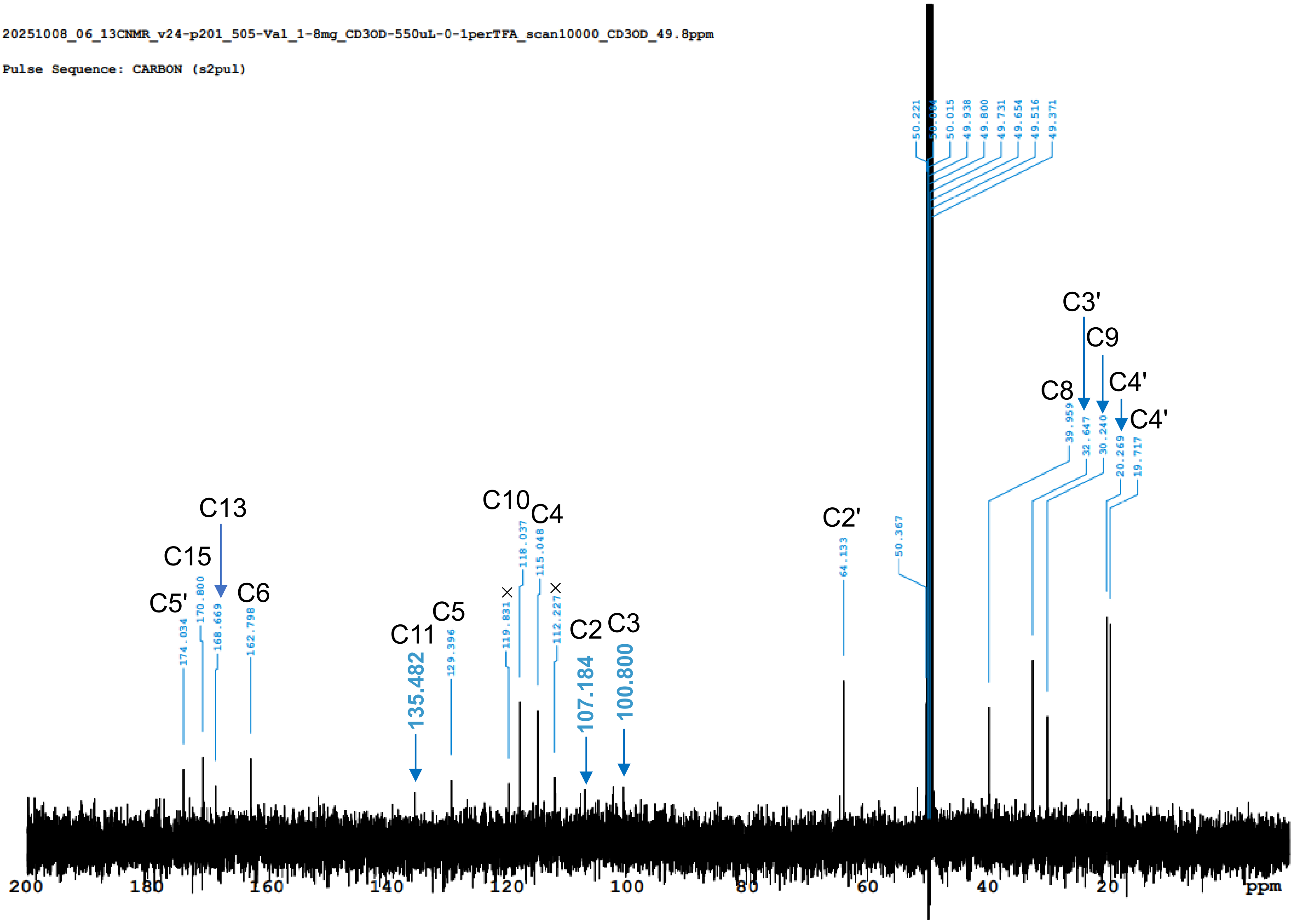

Figure S94. ^{13}C NMR spectrum of **12e** (1.80 mg) (151 MHz, CD_3OD: 550 µL - 0.1% TFA).

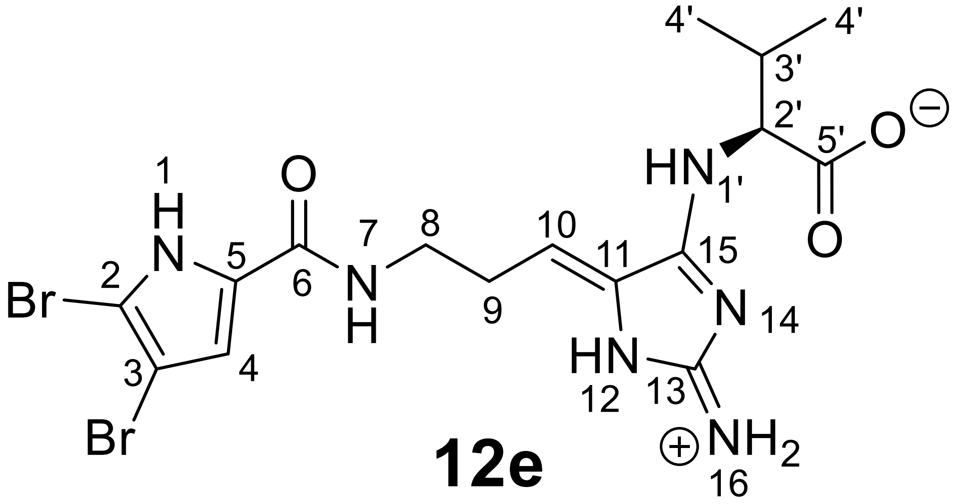

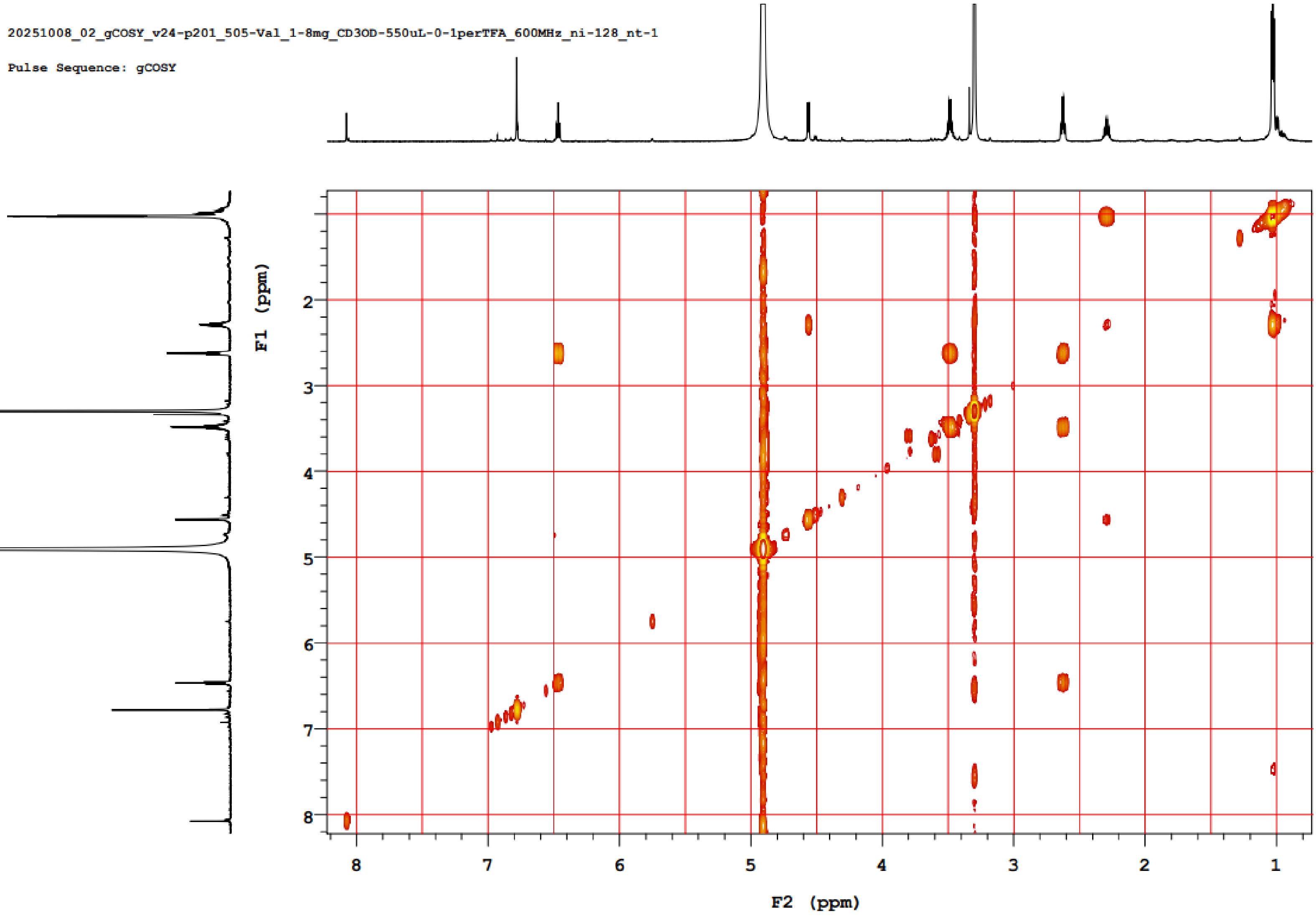

Figure S95. COSY spectrum of **12e** (1.80 mg) (600 MHz, CD_3OD: 550 µL - 0.1% TFA).

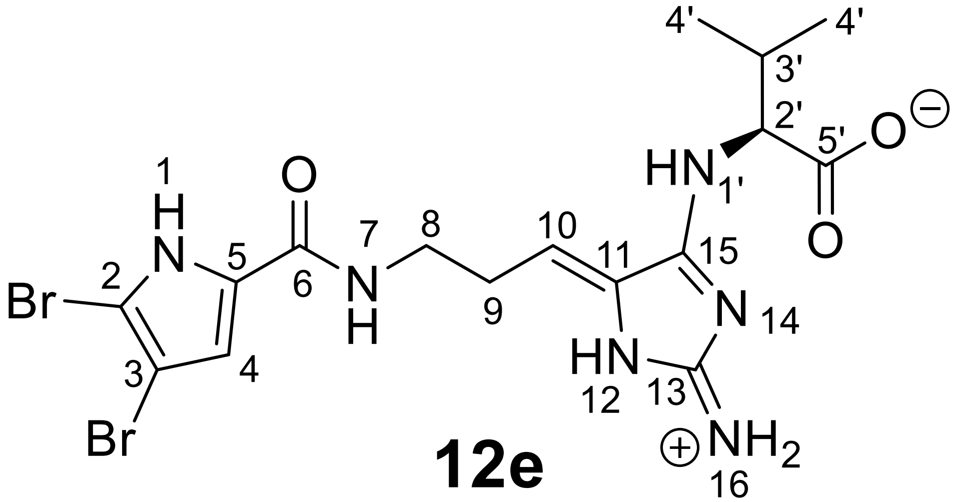

12e

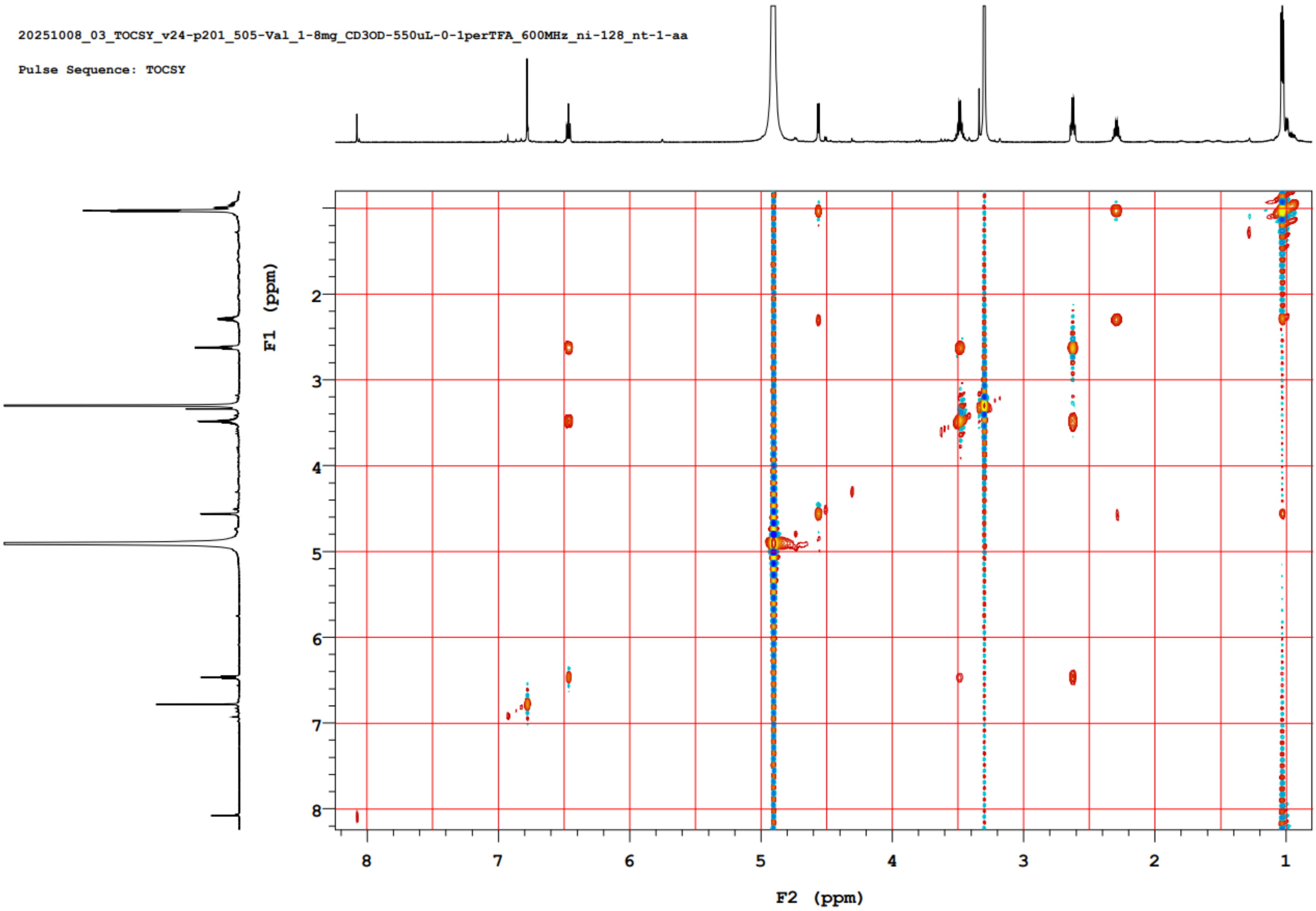

Figure S96. TOCSY spectrum of **12e** (1.80 mg) (600 MHz, CD_3OD: 550 µL - 0.1% TFA).

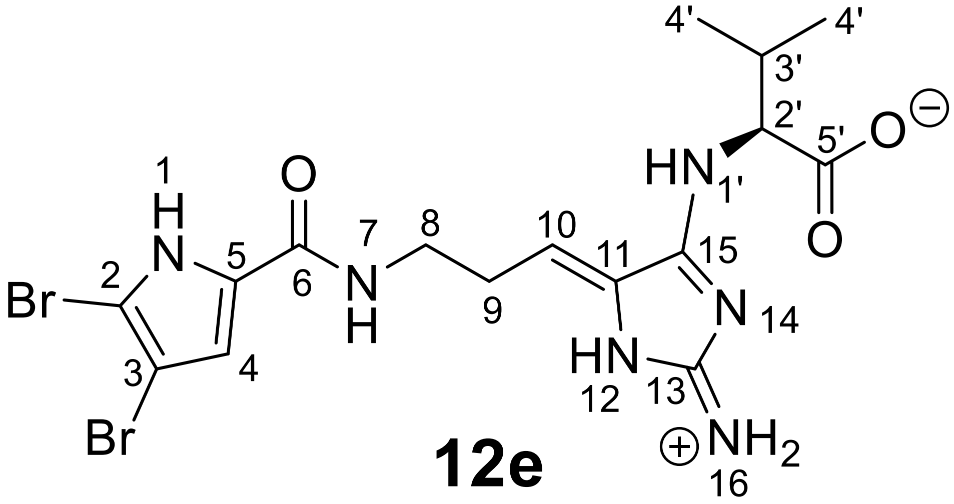

12e

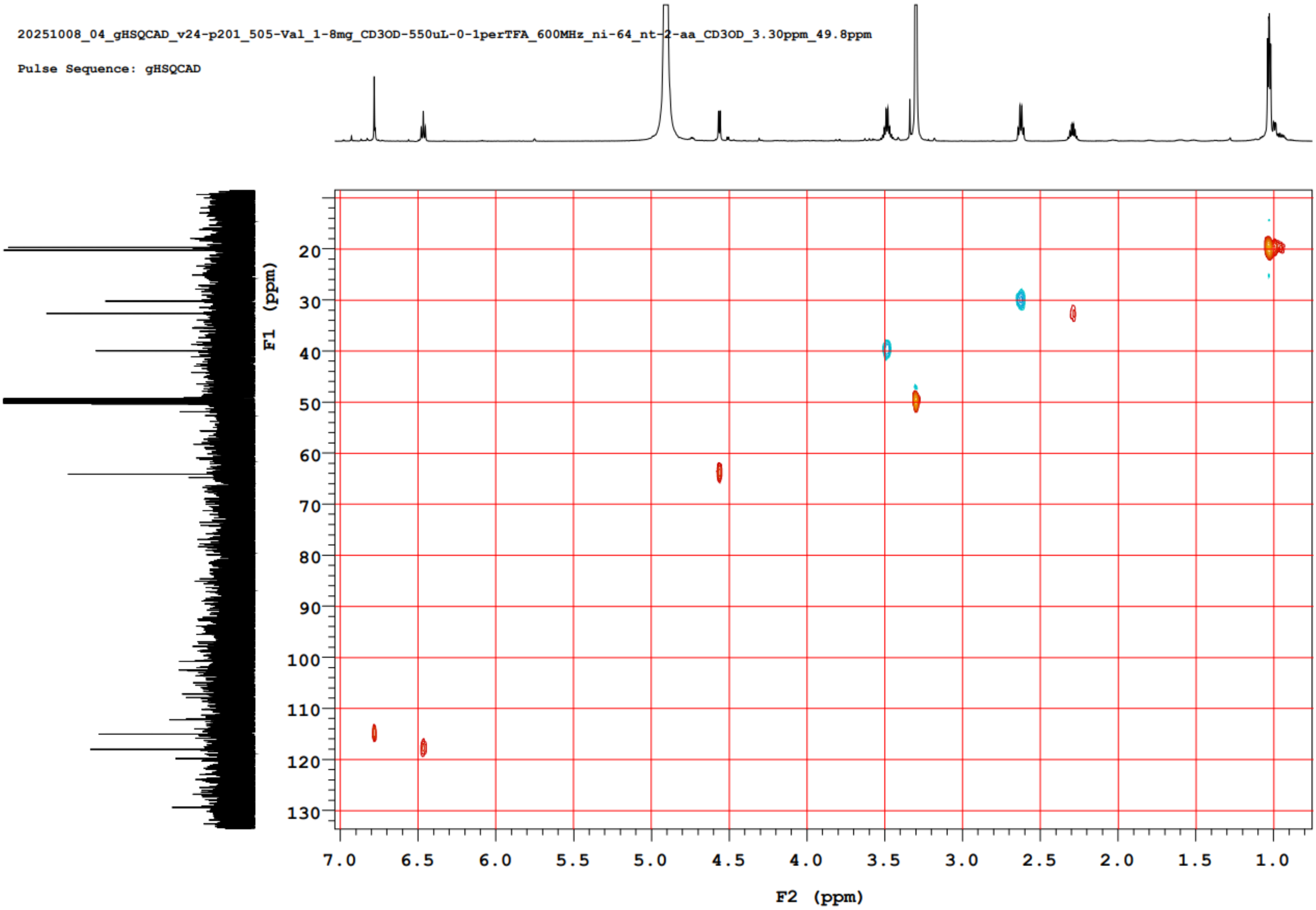

Figure S97. ^{1}H-^{13}C HSQC spectrum of **12e** (1.80 mg) (600 MHz/151 MHz, CD_3OD: 550 µL - 0.1% TFA).

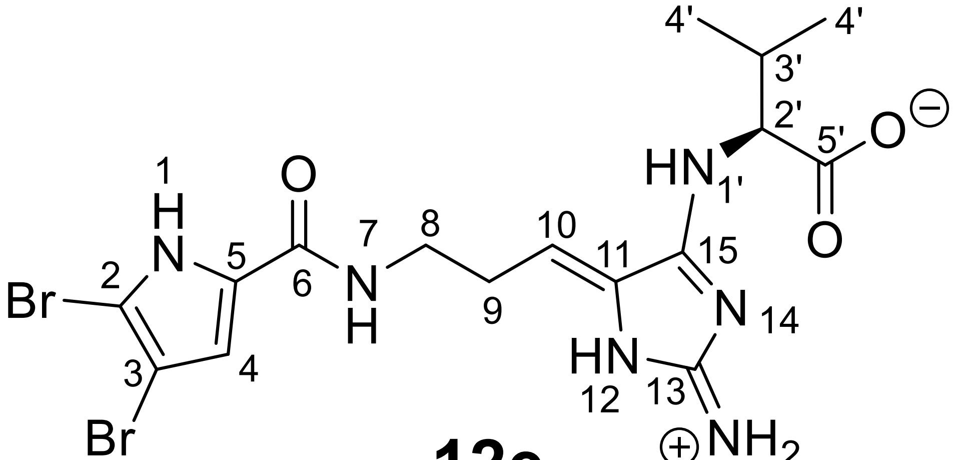

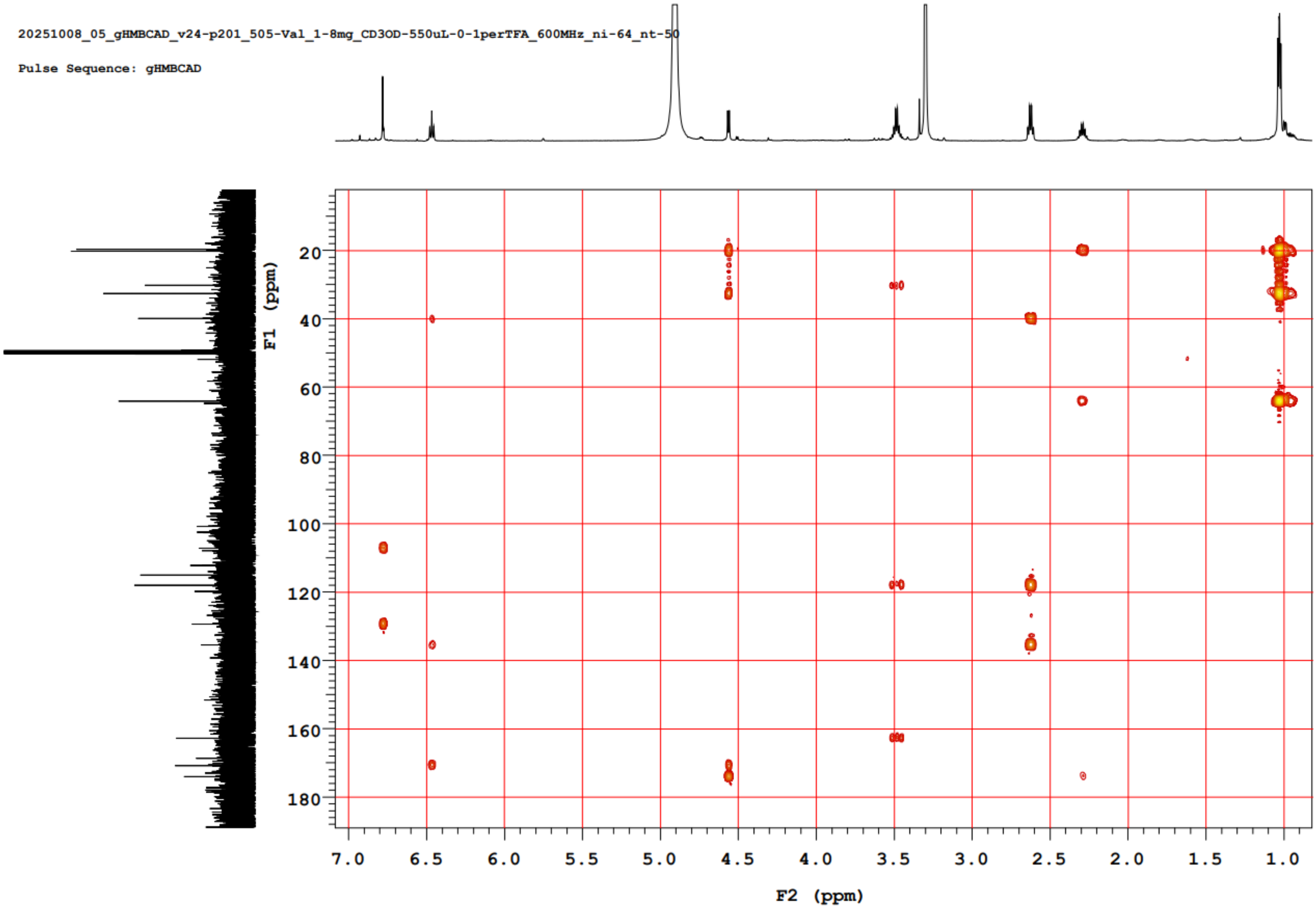

Figure S98. ^1H-^{13}C HMBC spectrum of **12e** (1.80 mg) (600 MHz/151 MHz, CD_3OD: 550 µL - 0.1% TFA).

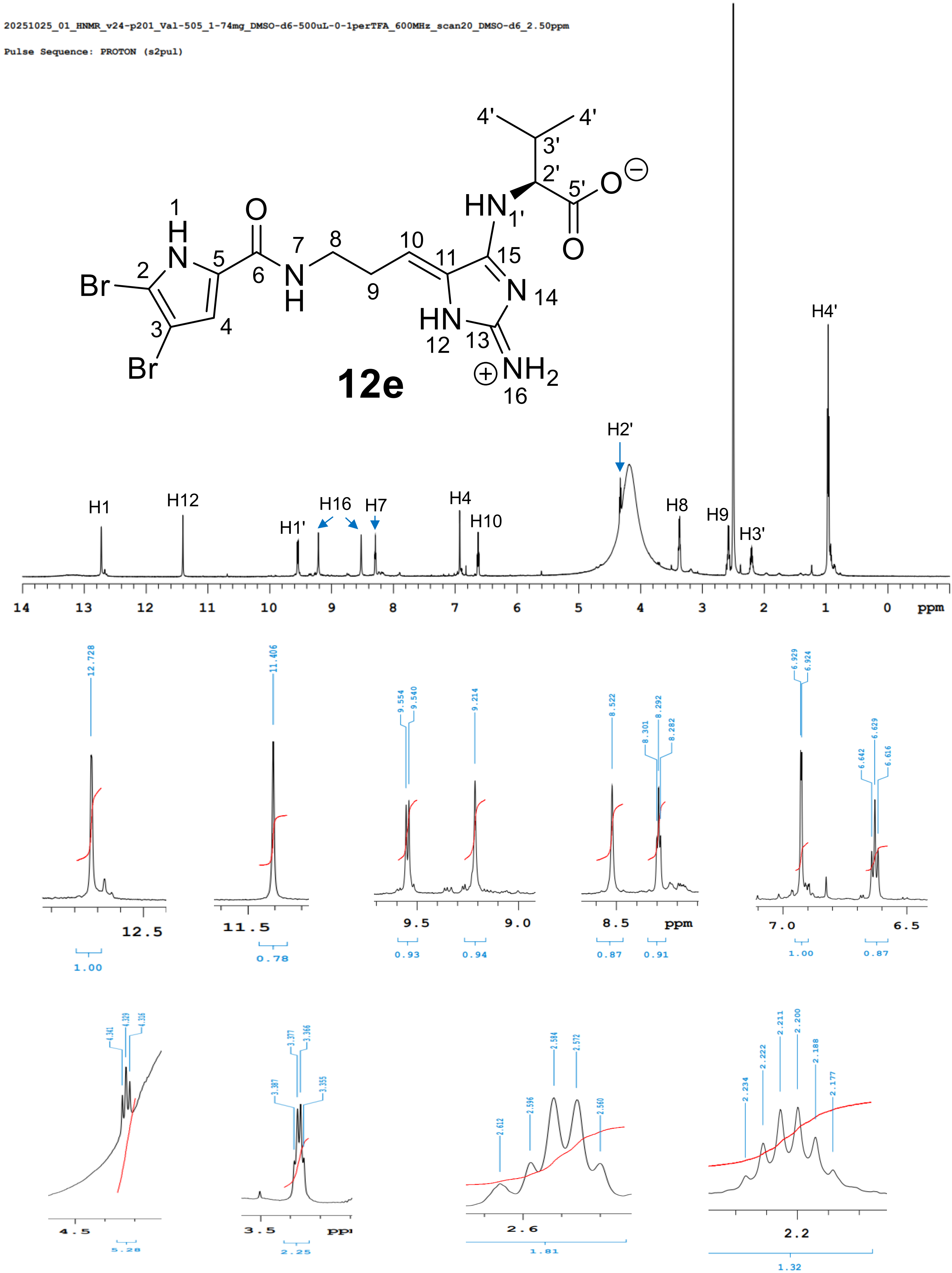

Figure S99. ^{1}H NMR spectrum of **12e** (1.74 mg) (600 MHz, DMSO-d_6: 500 µL - 0.1% TFA).

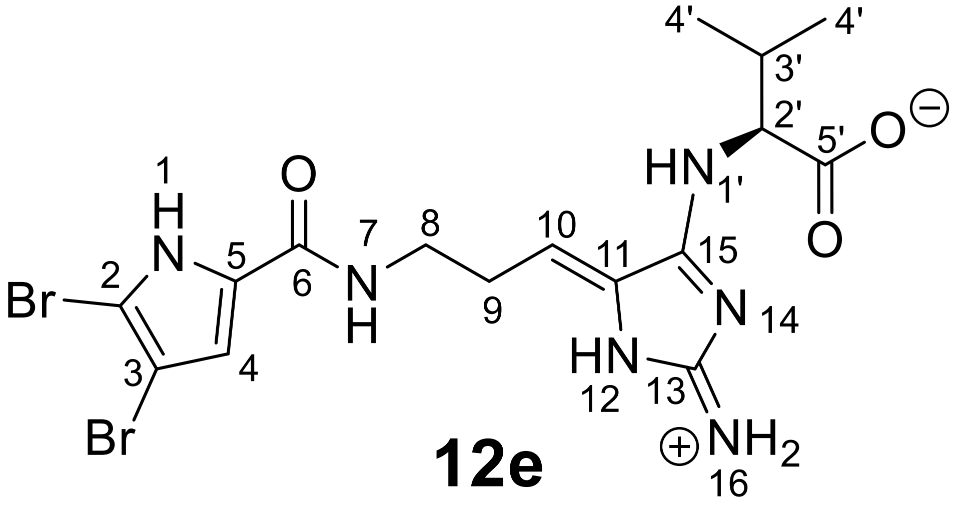

12e

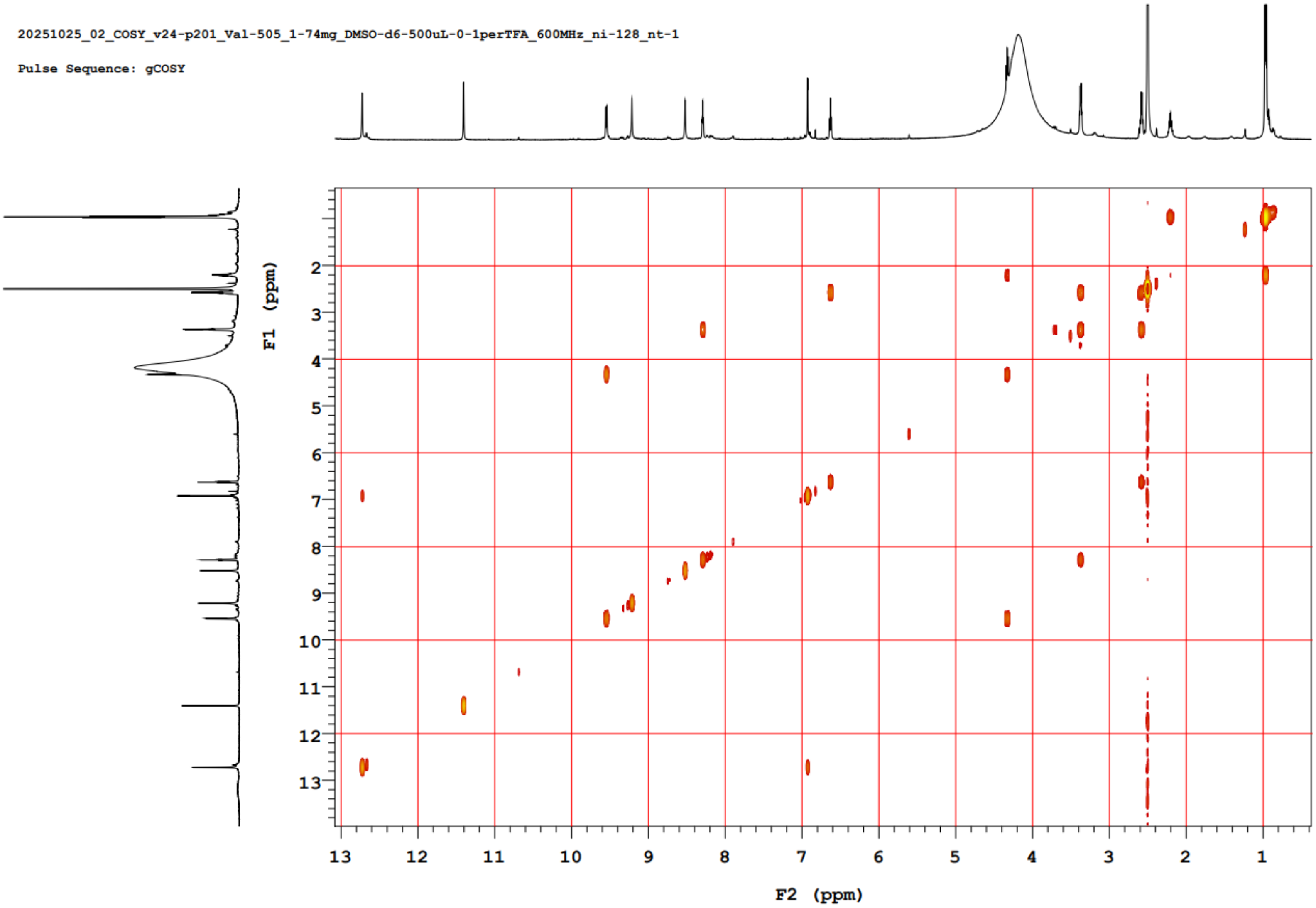

Figure S100. COSY spectrum of **12e** (1.74 mg) (600 MHz, DMSO-d_6: 500 µL - 0.1% TFA).

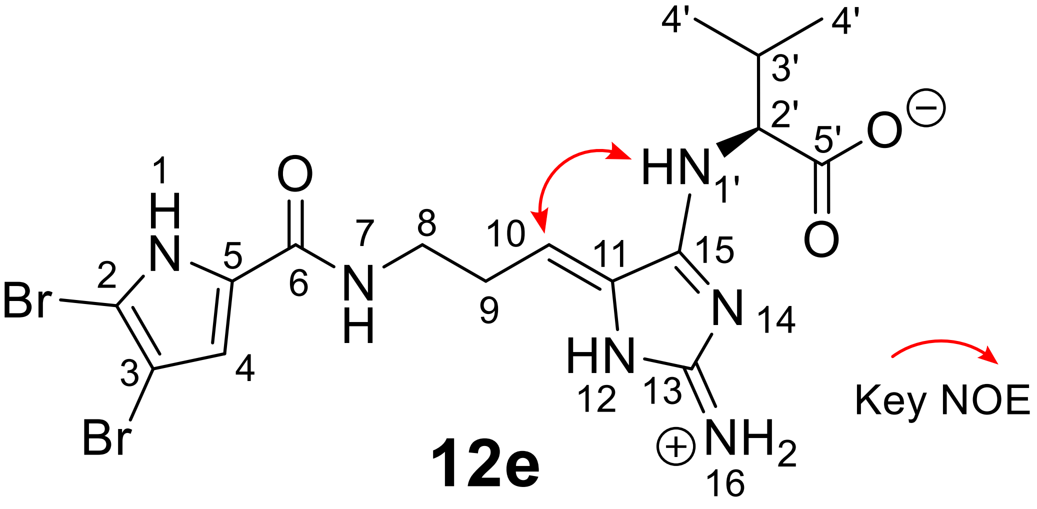

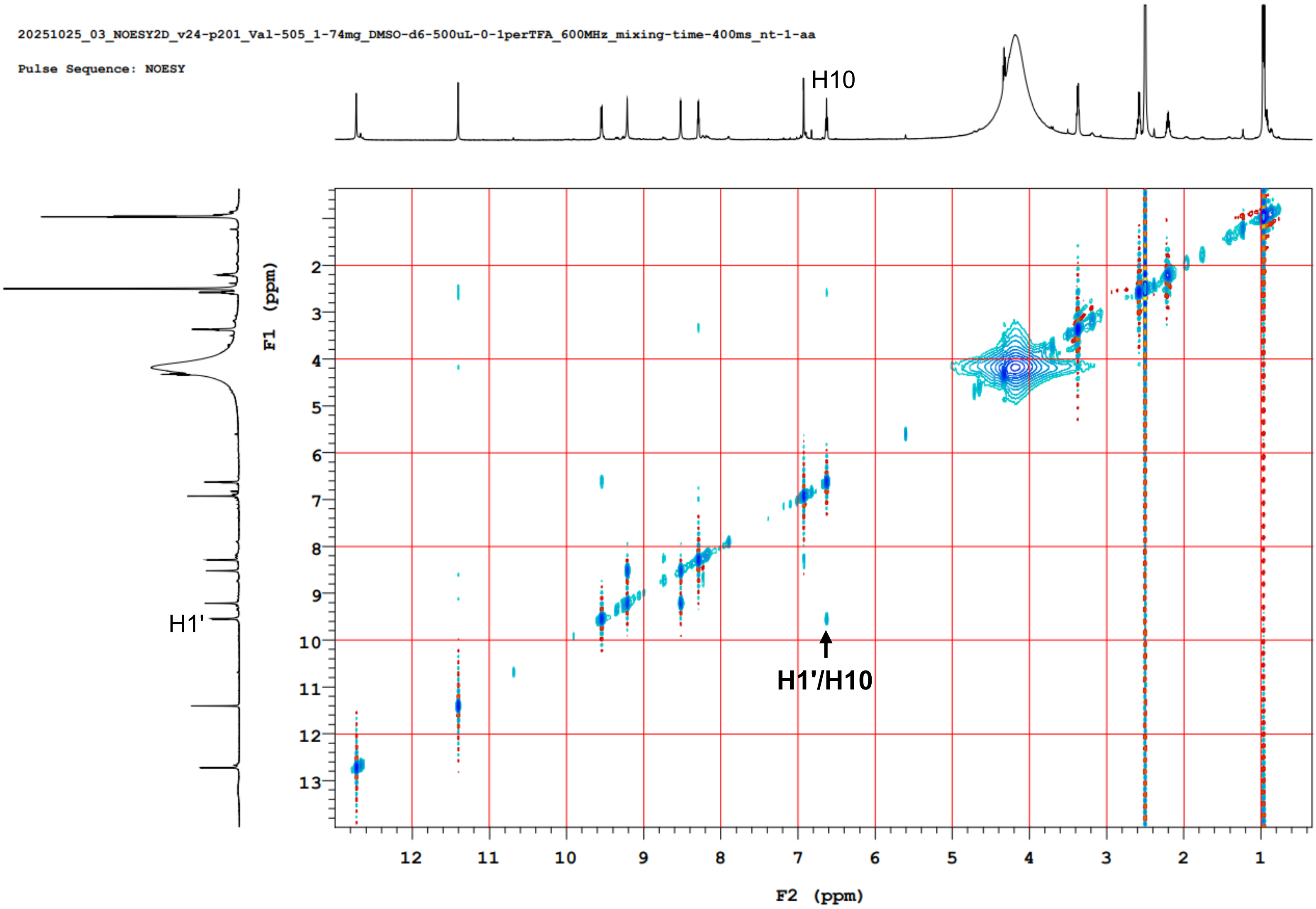

Figure S101. NOESY spectrum of **12e** (1.74 mg) (600 MHz, DMSO-d_6: 500 μL - 0.1% TFA).

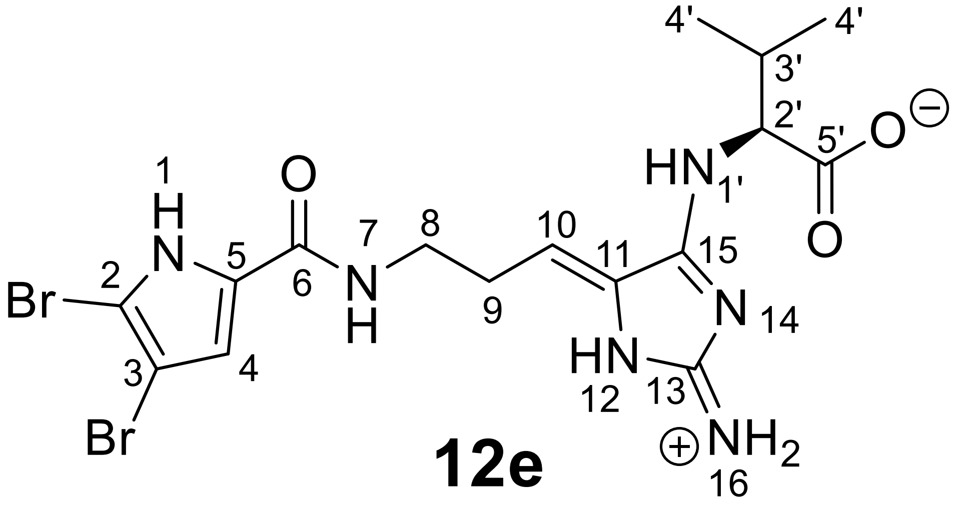

12e

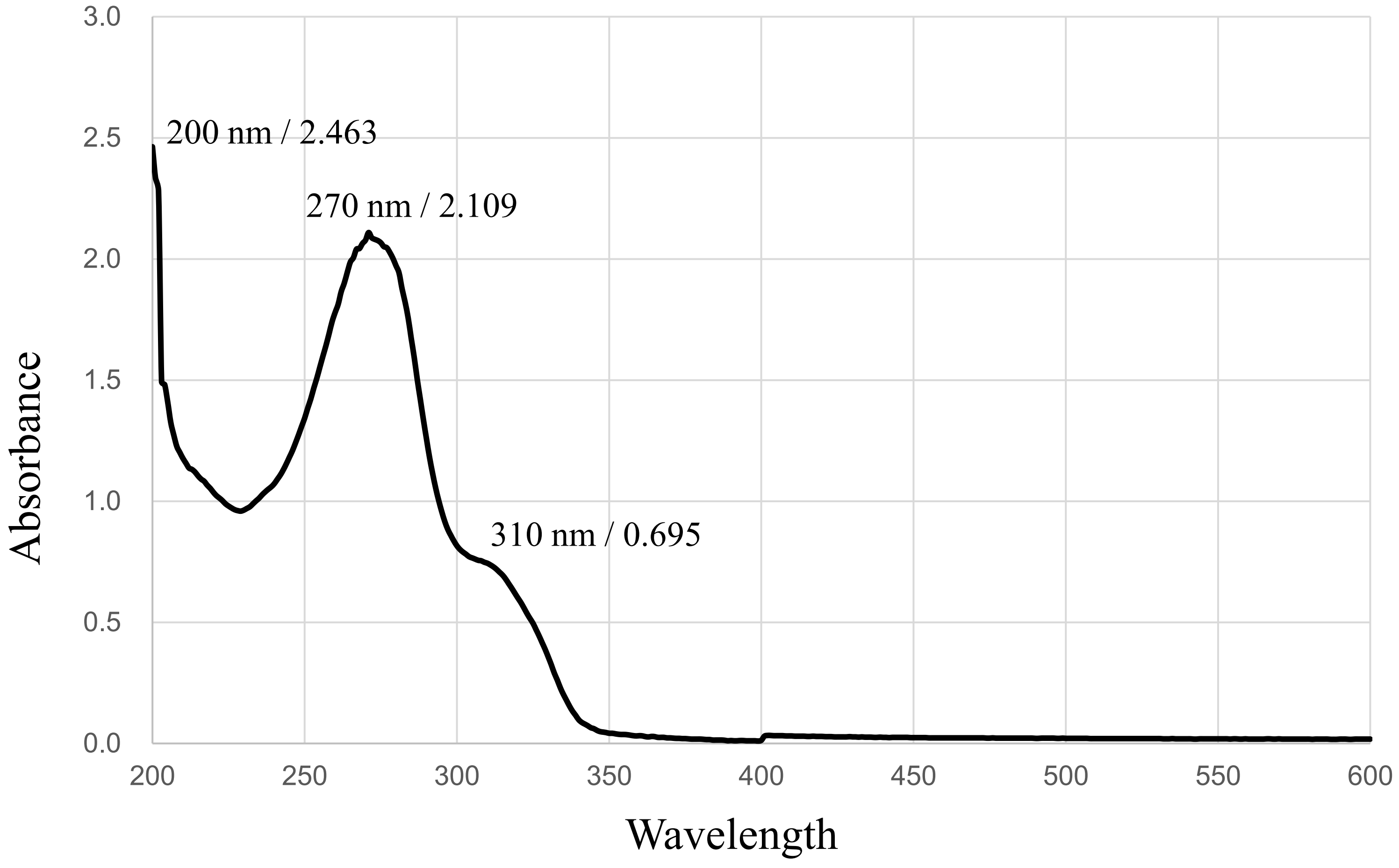

Figure S102. UV absorption spectrum of **12e** (MeOH). c = 1.24 x 10^{-4} (M)

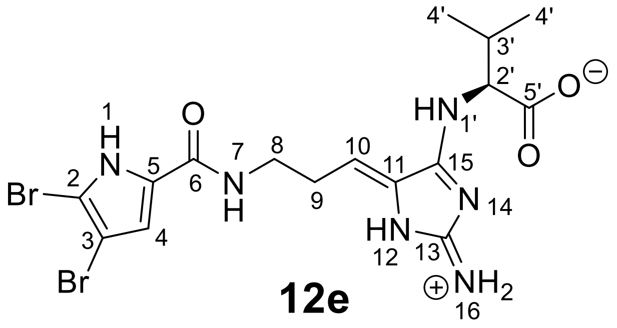

12e

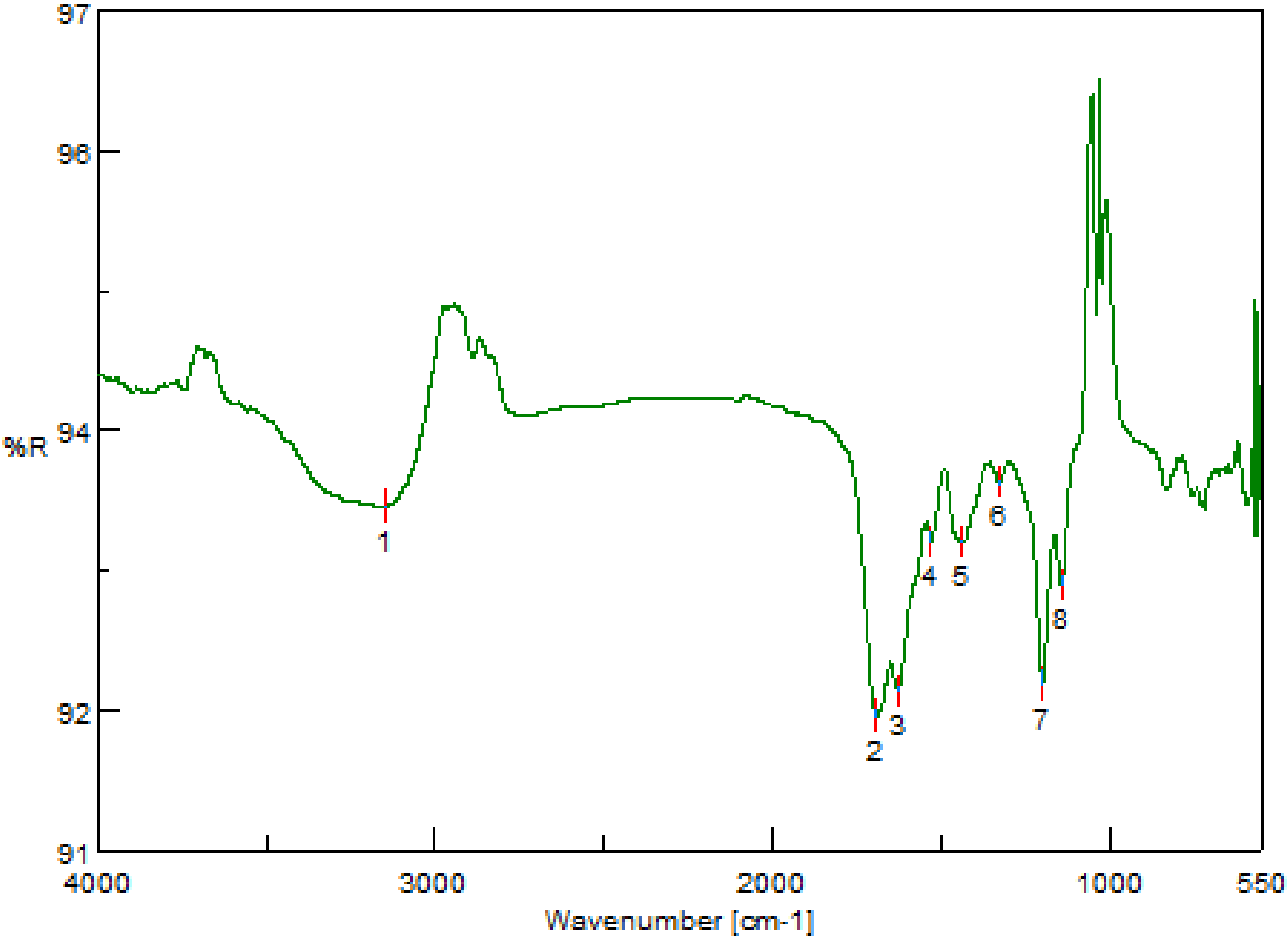

No.	Wavenumber	Strength
1	3144.4	93.451
2	1691.3	91.9655
3	1626.7	92.1348
4	1529.3	93.2026
5	1439.6	93.1995
6	1328.7	93.6273
7	1198.5	92.1836
8	1143.6	92.8924

Figure S103. IR spectrum of **12e** (ATR).

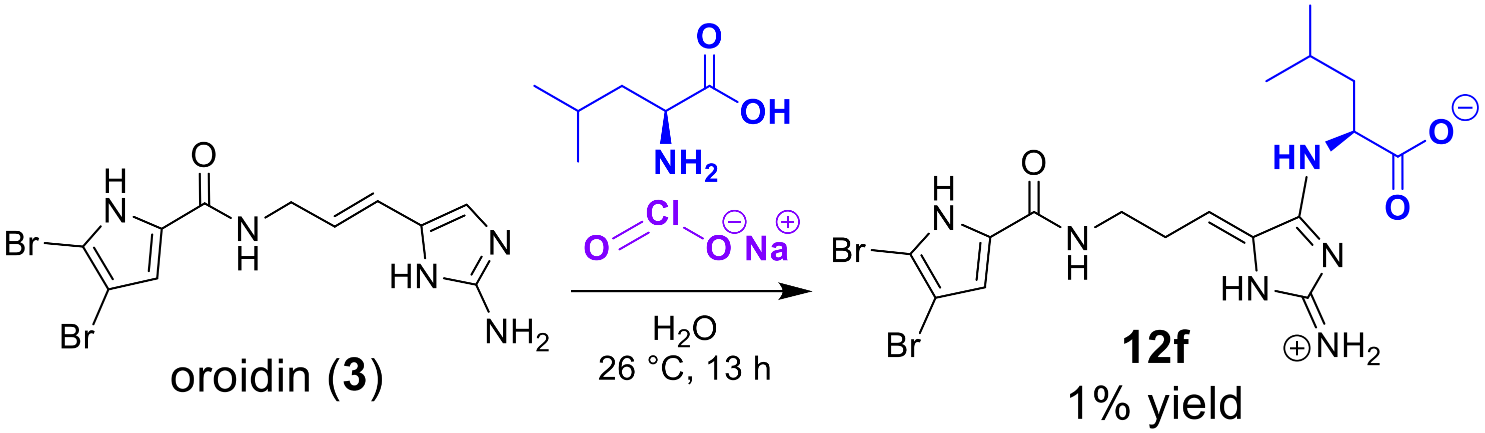

Scheme S6. Synthesis of **12f**.

Ten batches of oroidin (**3**) (HCOOH salt, 3.0 mg, 0.0069 mmol each; 30 mg, 0.069 mmol in total) were placed in 20 mL round-bottomed flasks, and H_2O (4.0 mL) was added to each flask with stirring. L-Leucine (1.31 g, 10 mmol, 1449 equiv.) was then added to the mixtures, followed by the addition of $NaClO_2$ (120 mg, 1.33 mmol, 193 equiv.). The flasks were sealed with septa caps, and the reaction mixtures were stirred at 26 °C for 13 h. After completion, the mixtures were combined and filtered through a small pad of Celite, rinsing the flasks and filter cake with H_2O. The filtrate was directly purified by ODS silica gel column chromatography (MeOH/H_2O, 0:100 to 100:0, v/v). The eluate was concentrated under reduced pressure, and the crude material was filtered through a Cosmospin filter H (0.45 µm). Further purification was performed by RP-HPLC (InertSustain AQ-C18, 5 µm, 10 mm i.d. × 250 mm; GL Science) using gradient elution (0–4 min, MeOH/H_2O/HCOOH = 3:97:0.1 to 40:60:0.1, v/v; 4-40 min, 40:60:0.1; 40.1 min-, 70:30:0.1) at a flow rate of 2.0 mL/min. Semi-pure **12f** was obtained at 50-60 min, added one drop of TFA, and further purified by RP-HPLC (InertSustain AQ-C18, 5 µm, 10 mm i.d. × 250 mm; GL Science) again using gradient elution (0–4 min, MeOH/H_2O/HCOOH = 50:50:0.1 to 60:40:0.1, v/v; 4 min-, 60:40:0.1) at a flow rate of 2.0 mL/min. Pure **12f** was obtained at 26-32 min (0.53 mg, 0.0010 mmol, 1% yield) as a white film.

Compound **12f** was difficult to separate from byproducts, and this reaction exhibited low reproducibility. Structural determination was carried out on the basis of ^{1}H NMR, COSY, and NOESY spectra recorded in DMSO-d_6 containing 0.1% TFA.

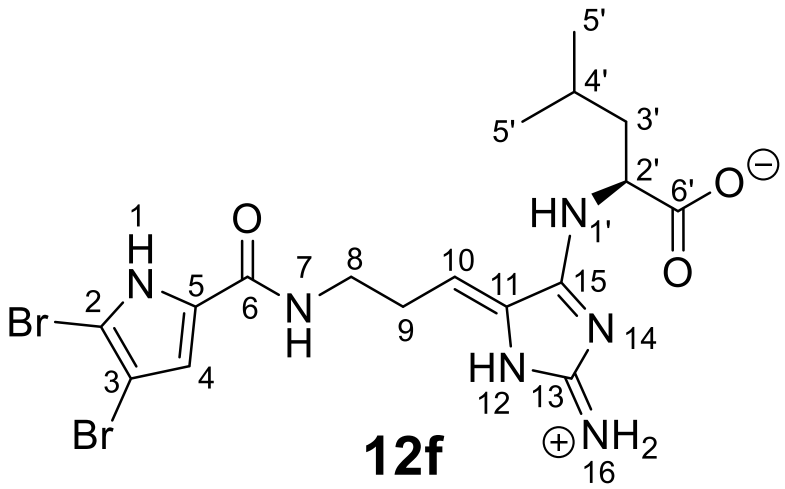

12f

12f:

R_f = 0.18 (broad) ($CHCl_3$/MeOH/28% NH_3 aq. = 50:50:1, v/v/v; UV).

UV/vis λ_{max} (MeOH) nm (log ε): 310 (3.64), 269 (4.19), 200 (4.57).

^{1}H NMR (600 MHz, DMSO-d_6 containing 0.1% TFA): δ 12.72 (s, N1-H, 1H), 11.42 (d, J = 4.8 Hz, N12-H, 1H), 9.69 (d, J = 7.8 Hz, N1'-H, 1H), 9.25 (s, N16-H, 1H), 8.55 (s, N16-H, 1H), 8.29 (t, J = 5.4 Hz, N7-H, 1H), 6.92 (s, C4-H, 1H), 6.42 (t, J = 7.8 Hz, C10-H, 1H), 4.45 (m, C2'-H, 1H), 3.37 (q, J = 6.2 Hz, C8-H, 2H), 2.58 (q, J = 7.0 Hz, C9-H, 2H), 1.78 (t, J = 9.9 Hz, C3'-H, 2H), 1.65 (m, C4'-H, 1H), 0.86 (major peak) (d, J = 6.0 Hz, C5'-H, 6H).

HRMS (**ESI**): (*m/z*) calcd for $C_{17}H_{23}{}^{79}Br^{81}BrN_6O_3^+$ $[M+H]^+$: 519.0173, found 519.0162.

IR v_{max}: 3732 (w), 3620 (w), 3207 (br), 2957 (m), 1700 (m), 1606 (s), 1546 (m), 1456 (m), 1395 (m), 1339 (m), 1235 (w), 1147 (w), 1053 (m), 1033 (m), 1013 (w).

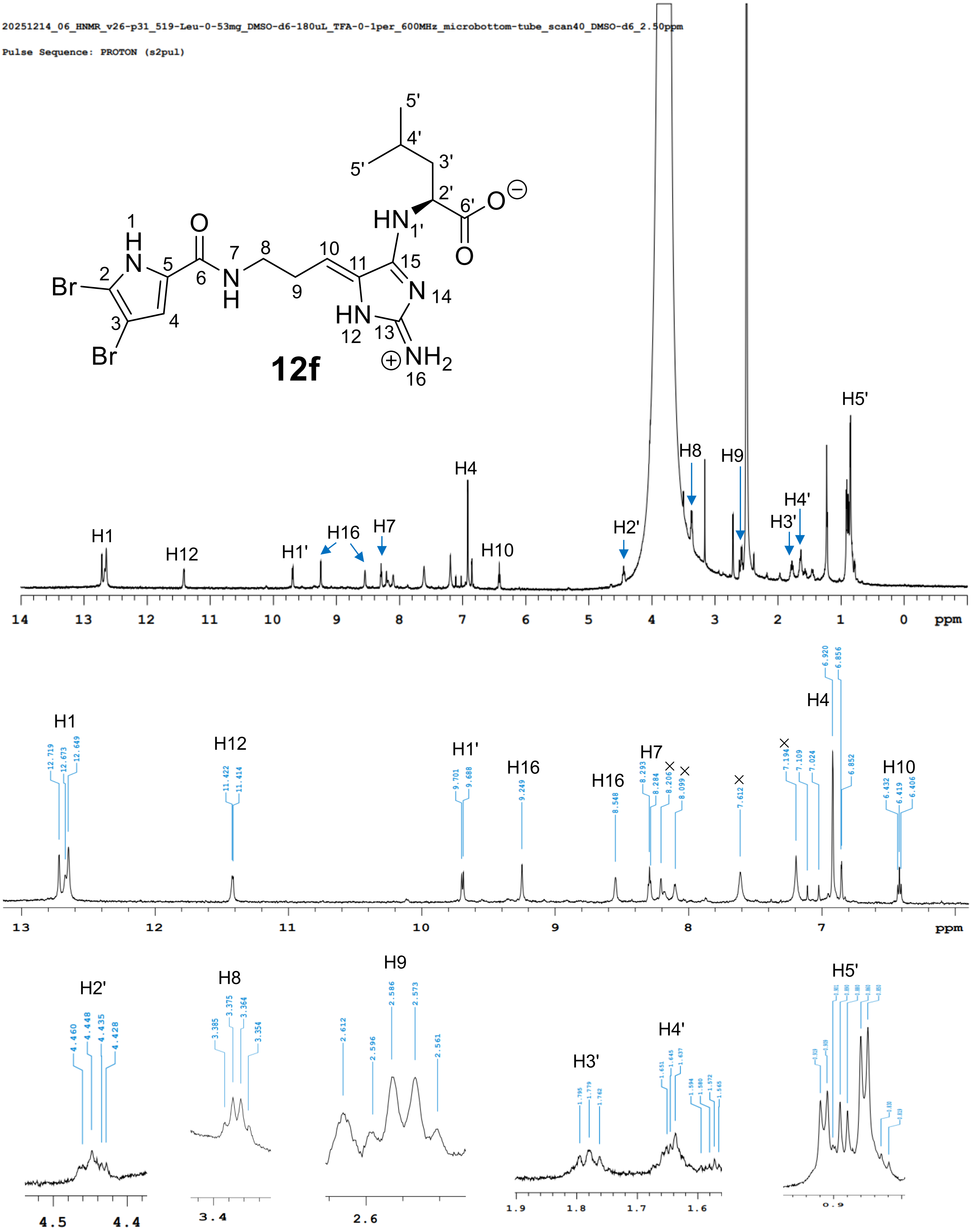

Figure S104. ^{1}H NMR spectrum of **12f** (0.53 mg) (600 MHz, DMSO-d_6: 180 µL - 0.1% TFA).

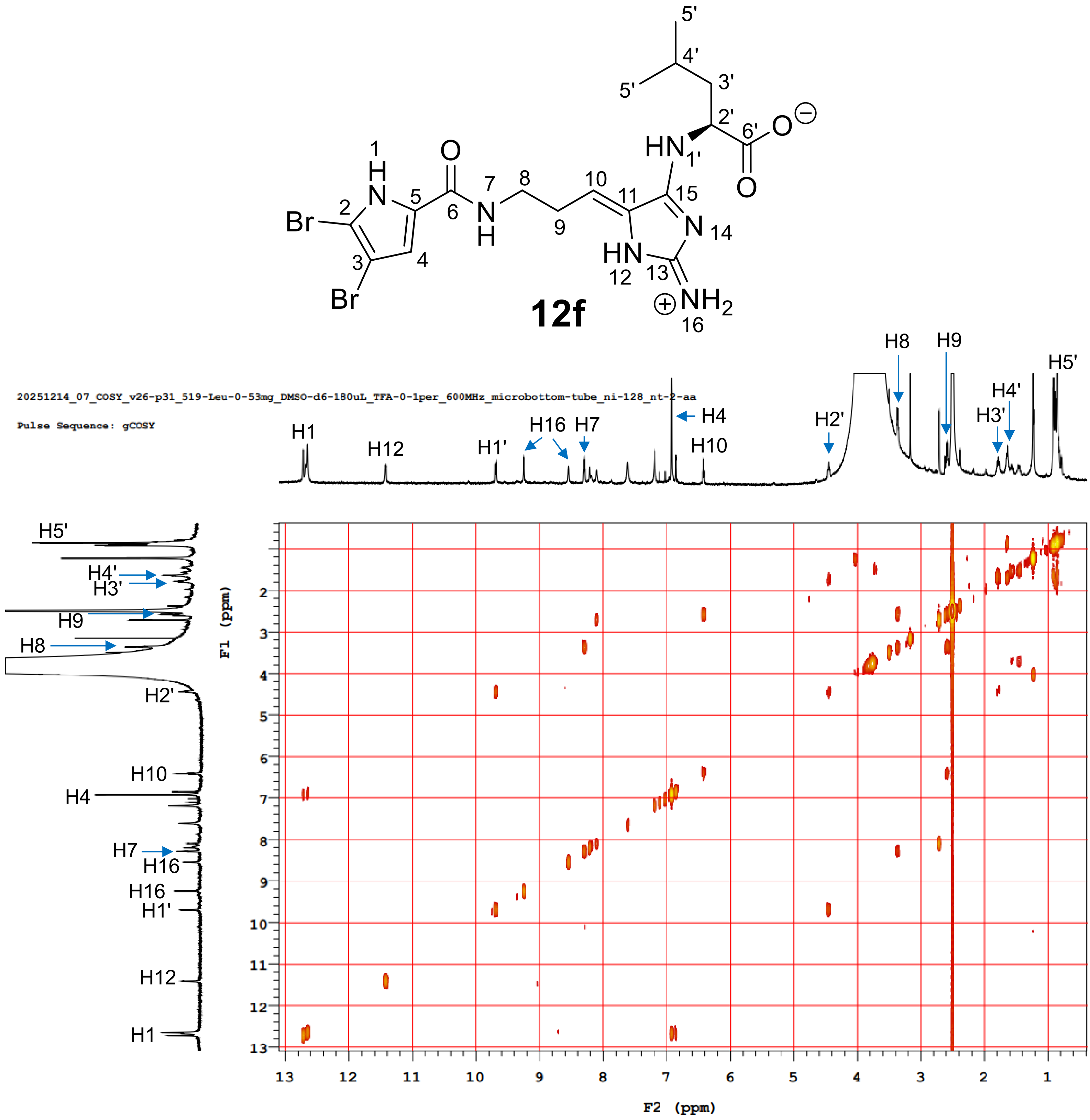

Figure S105. COSY spectrum of **12f** (0.53 mg) (600 MHz, DMSO-d_6: 180 µL - 0.1% TFA).

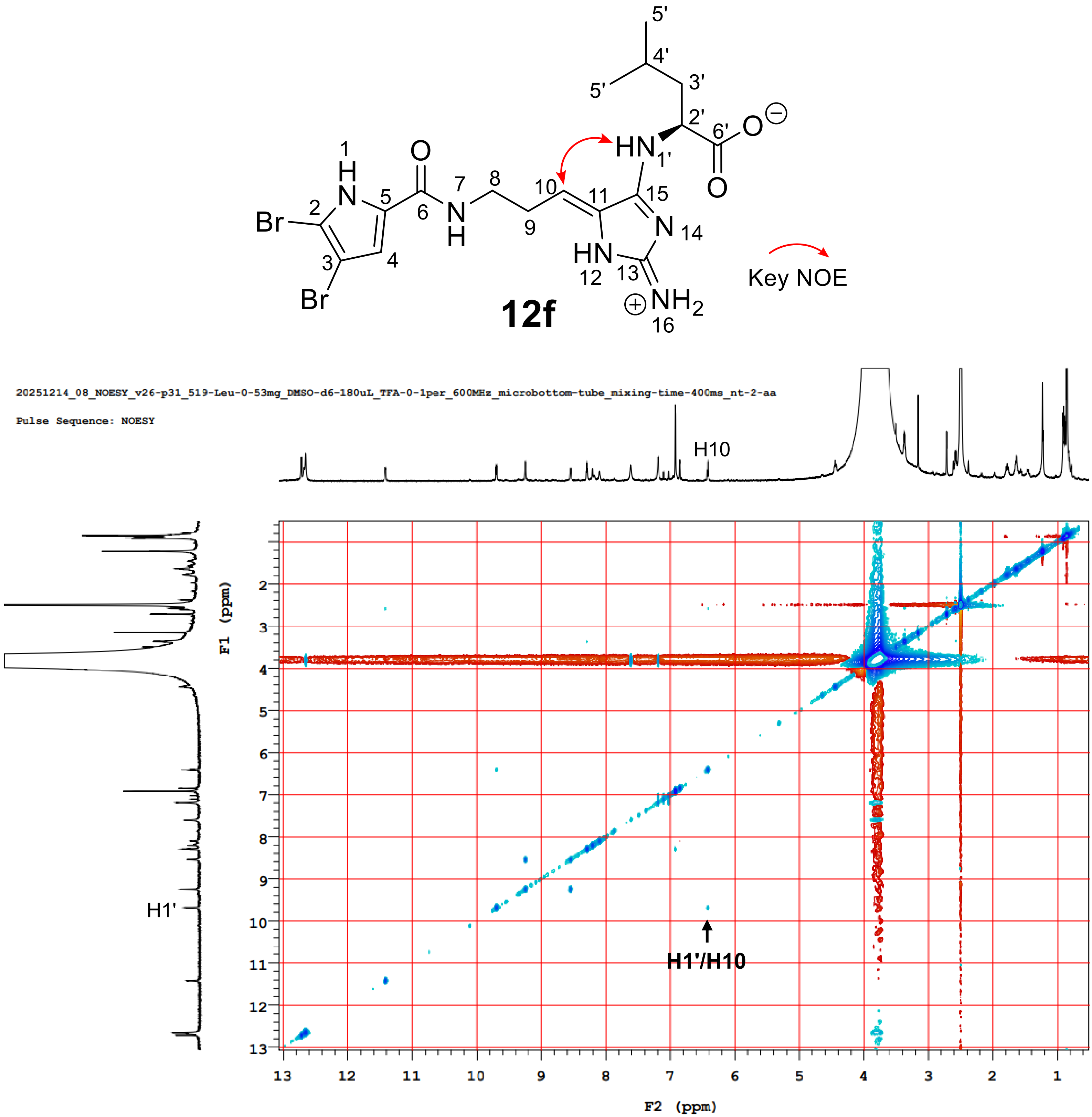

Figure S106. NOESY spectrum of **12f** (0.53 mg) (600 MHz, DMSO-d_6: 180 µL - 0.1% TFA).

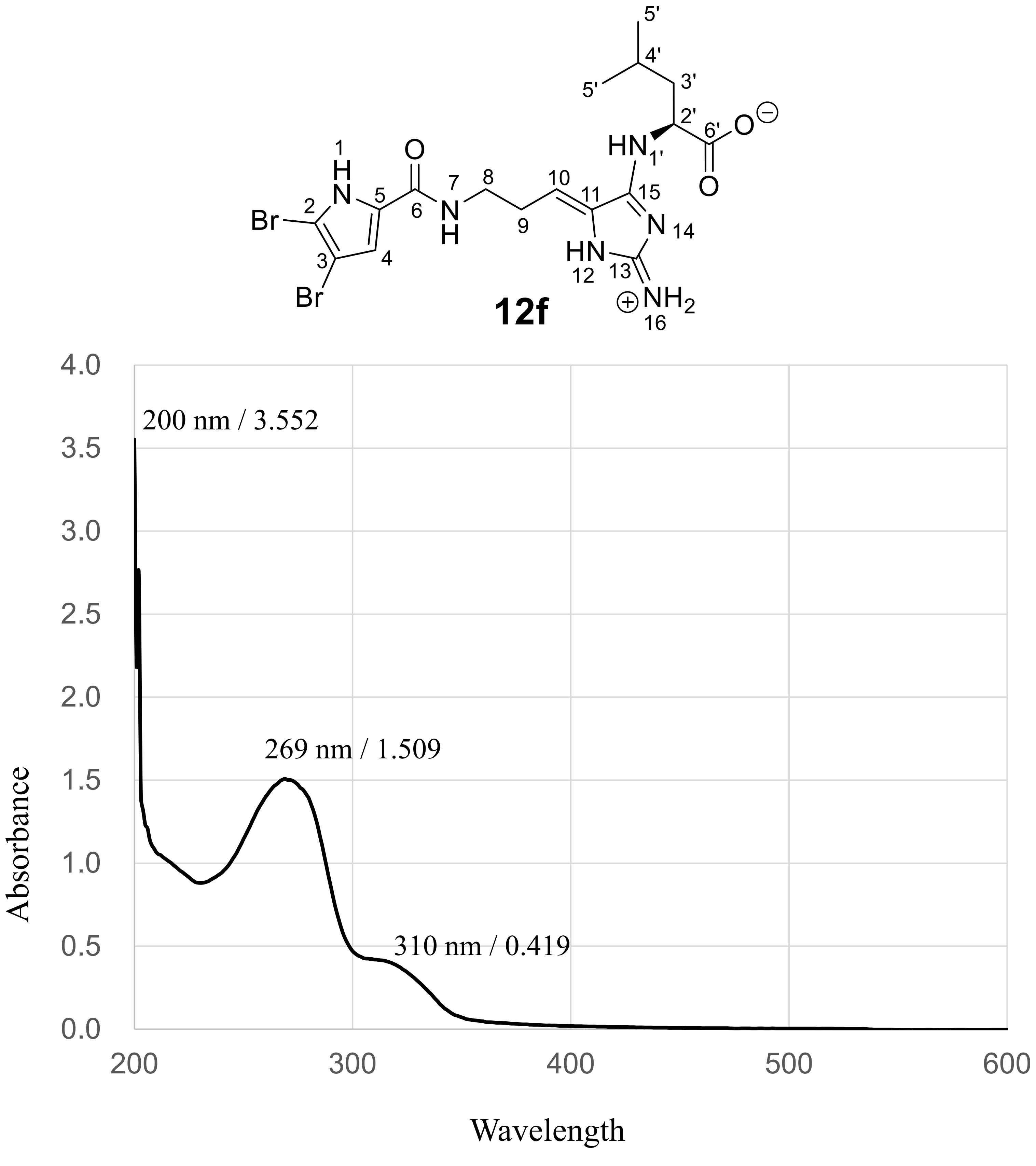

Figure S107. UV absorption spectrum of **12f** (MeOH). c = 9.65 x 10^{-5} (M)

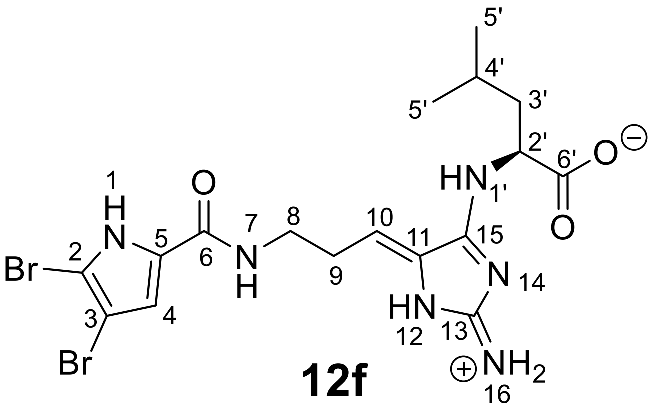

12f

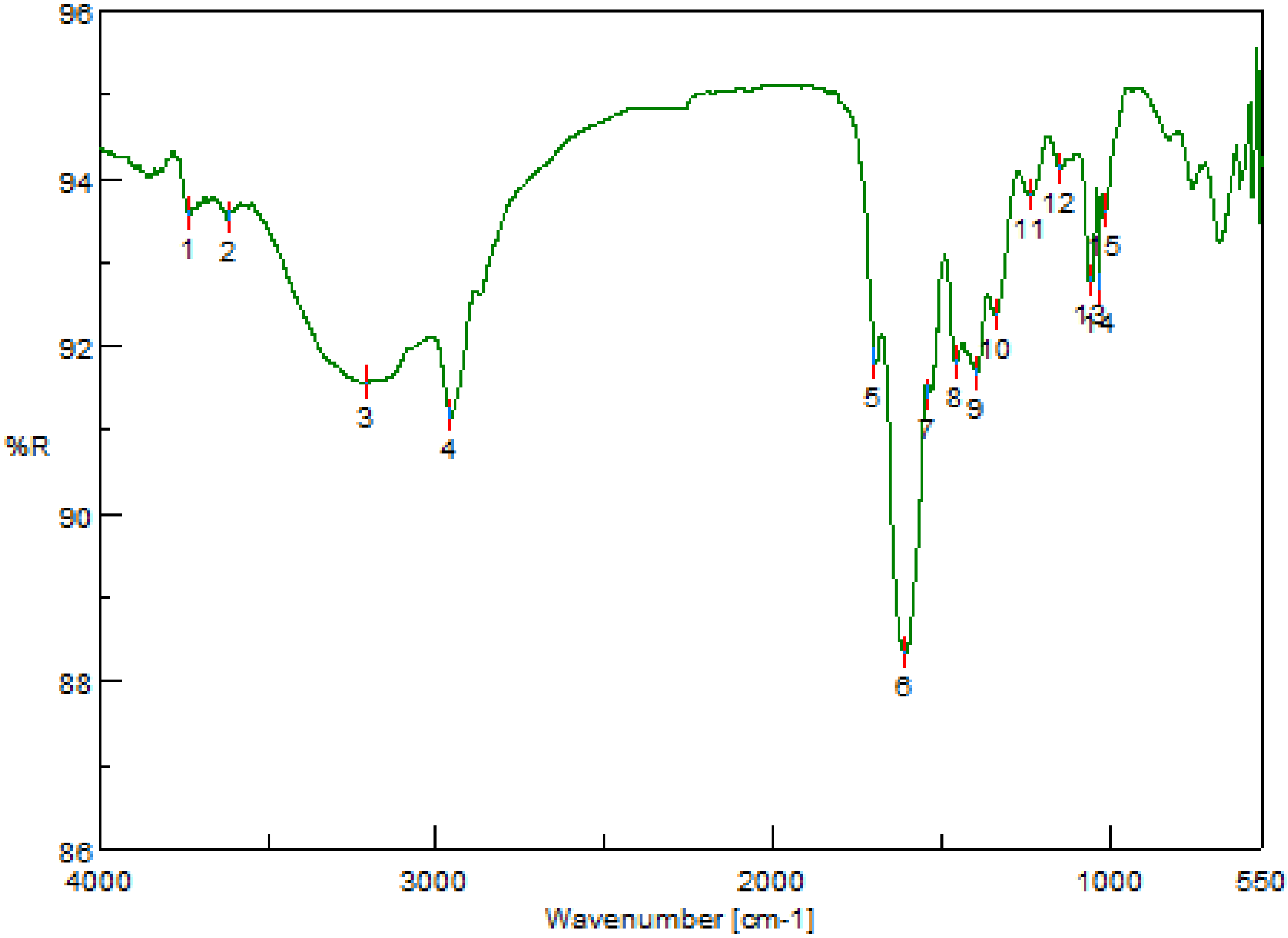

No.	Wavenumber	Strength	No.	Wavenumber	Strength
1	3731.6	93.5722	9	1395.3	91.6616
2	3619.7	93.5151	10	1339.3	92.3609
3	3207.0	91.5652	11	1235.2	93.7960
4	2957.3	91.1609	12	1146.5	94.1044
5	1699.9	91.7793	13	1052.9	92.7710
6	1606.4	88.3360	14	1032.7	92.6720
7	1545.7	91.4020	15	1013.4	93.5947
8	1456.0	91.8049			

Figure S108. IR spectrum of **12f** (ATR).

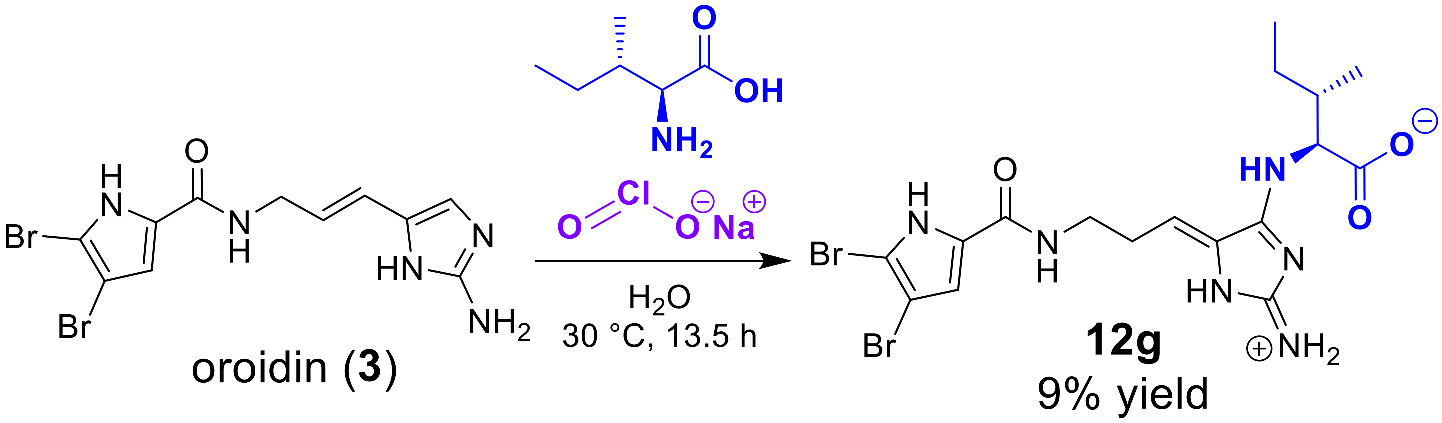

Scheme S7. Synthesis of **12g**.

Five batches of oroidin (**3**) (HCOOH salt, 3.0 mg, 0.0069 mmol each; 15 mg, 0.034 mmol in total) were placed in 20 mL round-bottomed flasks, and H_2O (4.0 mL) was added to each flask with stirring. L-Isoleucine (0.50 g, 3.81 mmol, 552 equiv.) was then added to the mixtures, followed by addition of $NaClO_2$ (120 mg, 1.33 mmol, 193 equiv.). The flasks were sealed with septa caps, and the reaction mixtures were stirred at 30 °C for 13.5 h. After completion, the mixtures were combined and filtered through a small pad of Celite, rinsing the flasks and filter cake with H_2O. The filtrate was directly purified by ODS silica gel column chromatography (MeOH/H_2O, 30:70 to 70:30, v/v). The eluate was concentrated under reduced pressure, and the crude material was filtered through a Cosmospin filter H (0.45 μm). Further purification was performed by RP-HPLC (InertSustain AQ-C18, 5 μm, 10 mm i.d. × 250 mm; GL Science) using gradient elution (0–4 min, MeOH/H_2O/HCOOH = 40:60:0.1 to 60:40:0.1, v/v; 4 min–, 60:40:0.1) at a flow rate of 2.0 mL/min. Pure **12g** was obtained at 23–27 min (1.61 mg, 0.00311 mmol, 9% yield) as an off-white to slightly yellow solid.

12g:

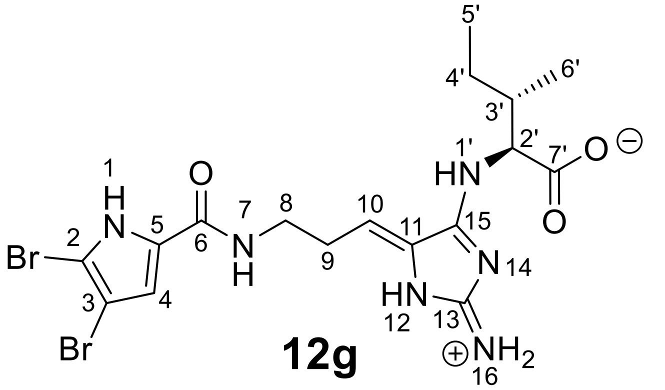

12g

$\mathbf{R_f}$ = 0.66 ($CHCl_3$/MeOH/28%NH_3 aq. = 60:40:2, v/v/v; UV).

$\mathbf{[\alpha]_D^{25}}$: +5.4 (c = 7.75 x 10^{-4}, MeOH).

UV/vis λ_{max} (MeOH) nm (log ε): 310 (3.97), 272 (4.43), 200 (4.65).

^{1}H NMR (600 MHz, CD_3OD containing 0.1% TFA): δ 6.83 (s, C4-H, 1H), 6.78 (s, C4-H, 1H), 6.45 (t, J = 8.1 Hz, C10-H, 1H), 4.61 (d, J = 6.0 Hz, C2'-H, 1H), 3.48 (m, C8-H, 2H), 2.62 (q, J = 7.0 Hz, C9-H, 2H), 2.03 (m, C3'-H, 1H), 1.57 (m, C4'-H, 1H), 1.31 (m, C4'-H, 1H), 1.00 (d, J = 6.6 Hz, C6'-H, 3H), 0.95 (t, J = 7.2 Hz, C5'-H, 3H).

^{1}H NMR (600 MHz, DMSO-d_6 containing 0.1% TFA): δ 12.72 (d, J = 2.4 Hz, N1-H, 1H), 11.38 (s, N12-H, 1H), 9.56 (d, J = 7.8 Hz, N1'-H, 1H), 9.21 (s, N16-H, 1H), 8.51 (s, N16-H, 1H), 8.29 (t, J = 5.4 Hz, N7-H, 1H), 6.92 (d, J = 2.4 Hz, C4-H, 1H), 6.60 (t, J = 7.5 Hz, C10-H, 1H), 4.37 (t, J = 7.5 Hz, C2'-H, 1H), 3.37 (q, J = 6.4 Hz, C8-H, 2H), 2.57 (q, J = 7.2 Hz, C9-H, 2H), 1.95 (m, C3'-H, 1H), 1.47 (m, C4'-H, 1H), 1.26 (m, C4'-H, 1H), 0.93 (d, J = 6.6 Hz, C6'-H, 3H), 0.86 (t, J = 7.5 Hz, C5'-H, 3H).

^{13}C NMR (151 MHz, CD_3OD containing 0.1% TFA): δ 174.1 (C7'), 170.6 (C15), 168.7 (C13), 162.8 (C6), 135.5 (C11), 129.4 (C5), 118.0 (C10), 116.0 (C4), 115.0 (C4), 107.2 (C2), 100.8 (C3), 63.0 (C2'), 40.0 (C8), 39.0 (C3'), 30.2 (C9), 27.4 (C4'), 16.7 (C6'), 12.4 (C5').

HRMS (**ESI**): (*m/z*) calcd for $C_{17}H_{23}{}^{79}Br_2N_6O_3^+$ $[M+H]^+$: 517.0193, found 517.0183.

IR ν_{max}: 3175 (br), 2889 (br), 2778 (s), 1692 (s), 1623 (s), 1529 (m), 1438 (m), 1328 (w), 1204 (s), 1144 (m).

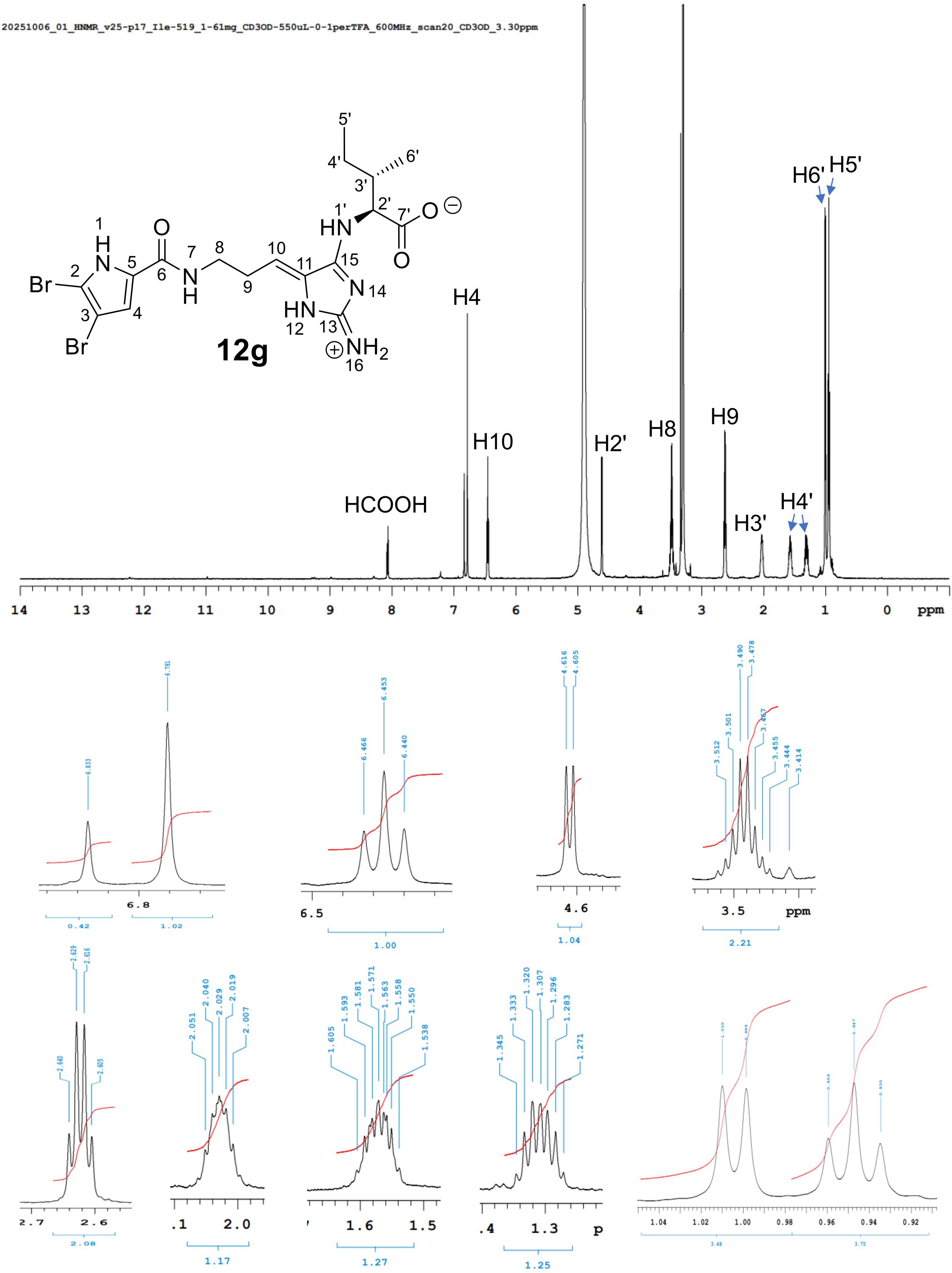

Figure S109. ^{1}H NMR spectrum of **12g** (1.61 mg) (600 MHz, CD_3OD: 550 µL - 0.1% TFA).

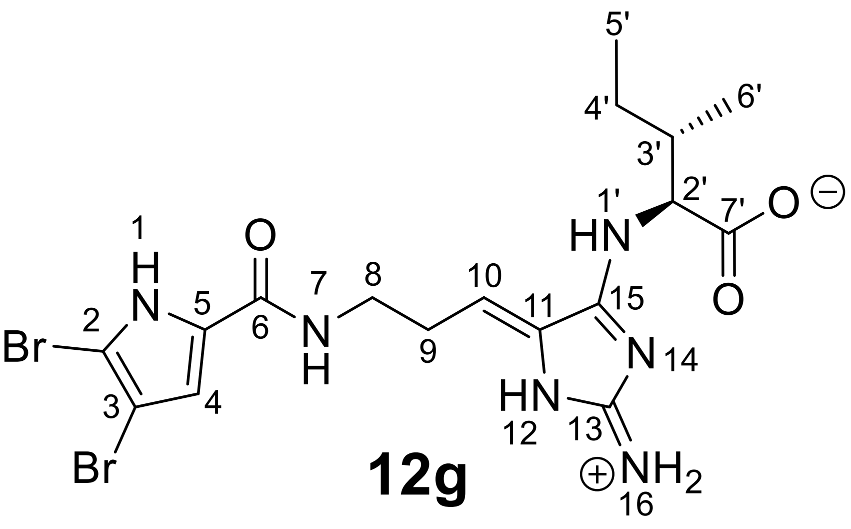

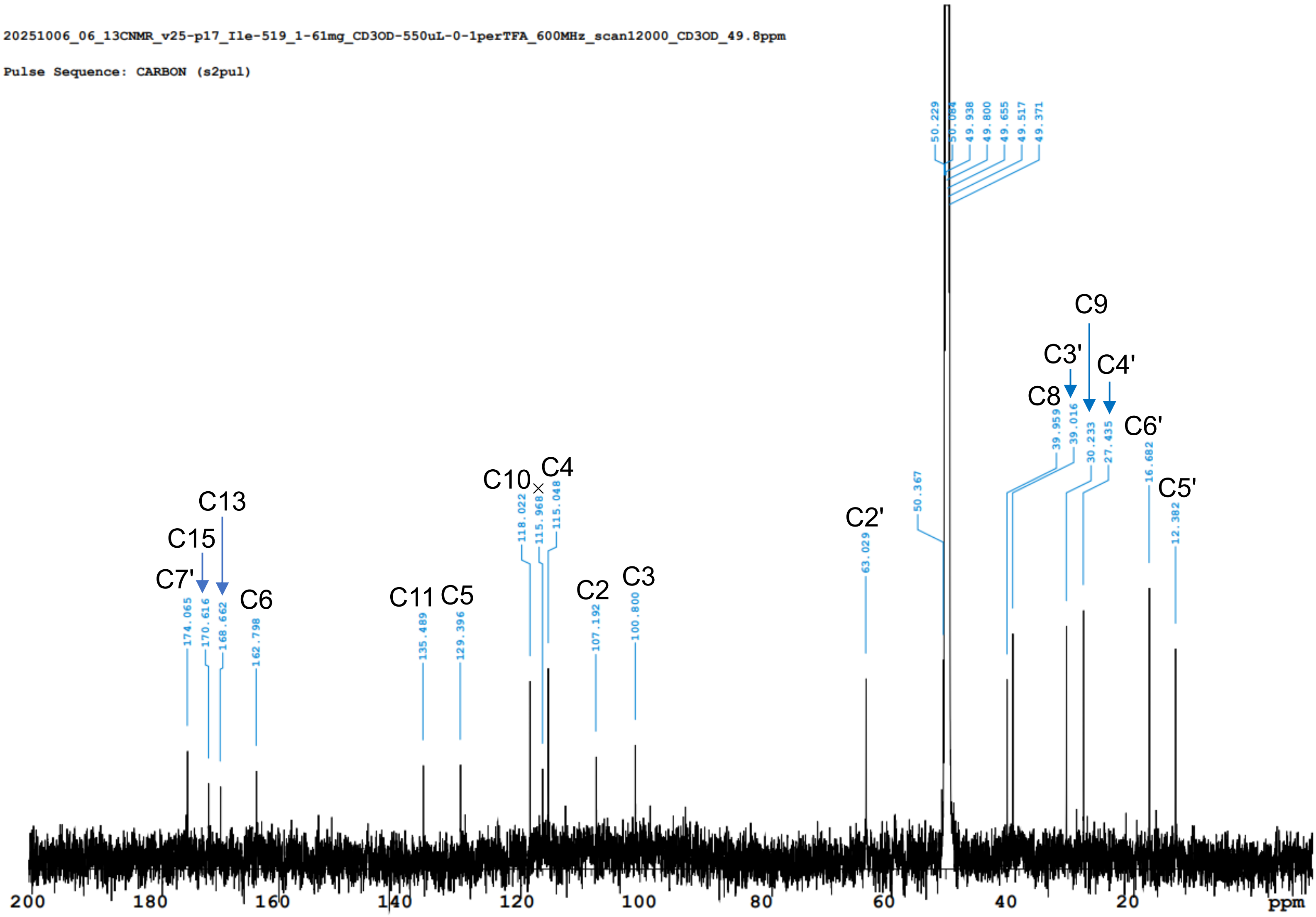

Figure S110. ^{13}C NMR spectrum of **12g** (1.61 mg) (151 MHz, CD_3OD: 550 µL - 0.1% TFA).

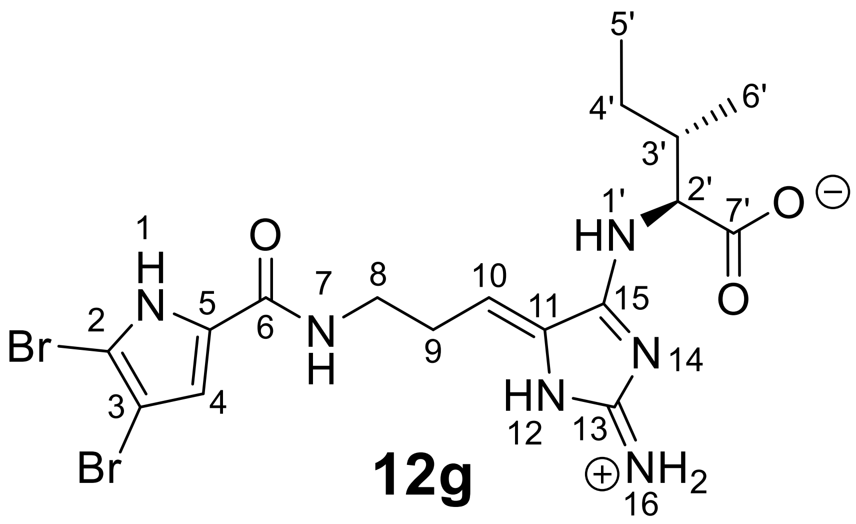

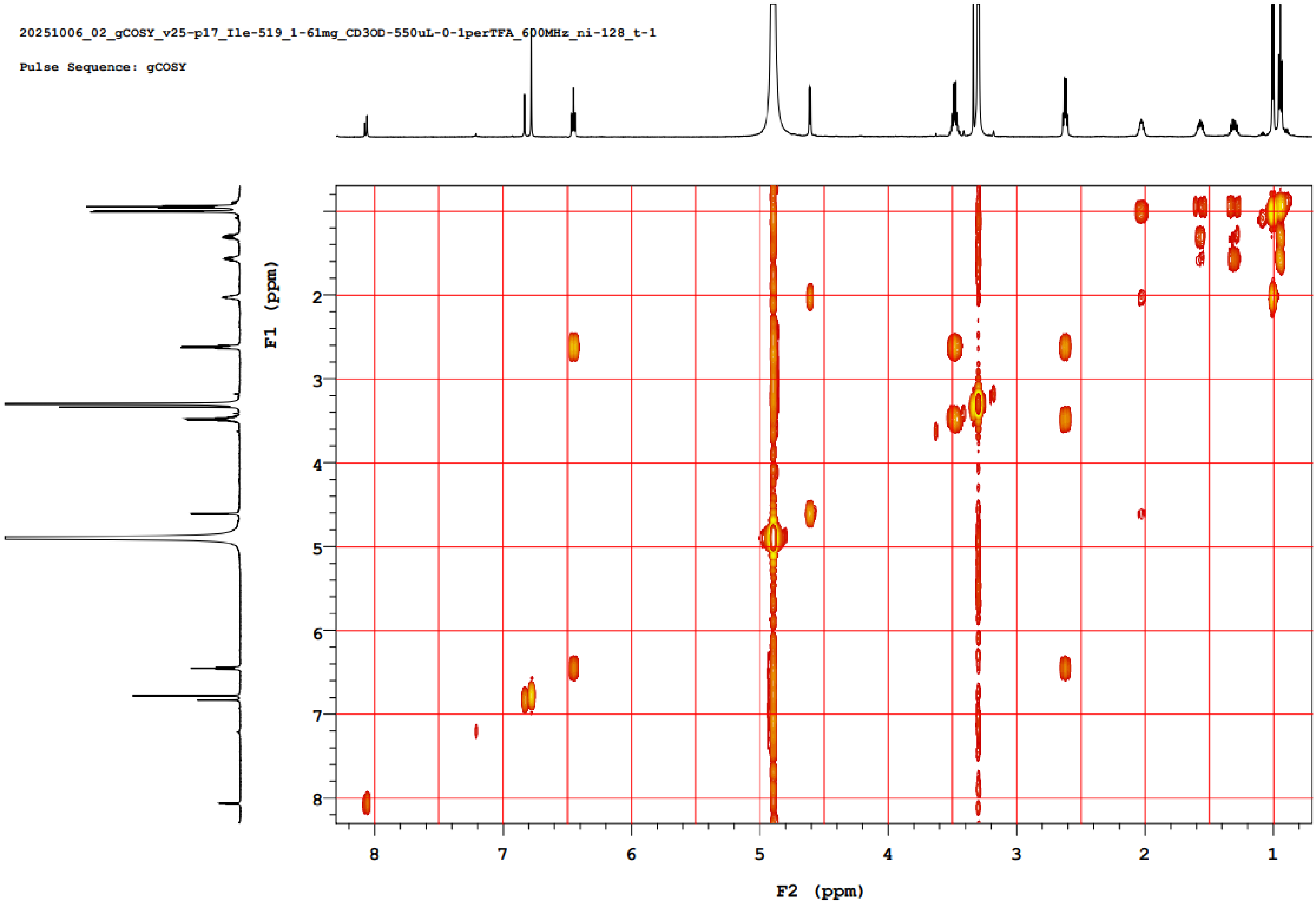

Figure S111. COSY spectrum of **12g** (1.61 mg) (600 MHz, CD_3OD: 550 µL - 0.1% TFA).

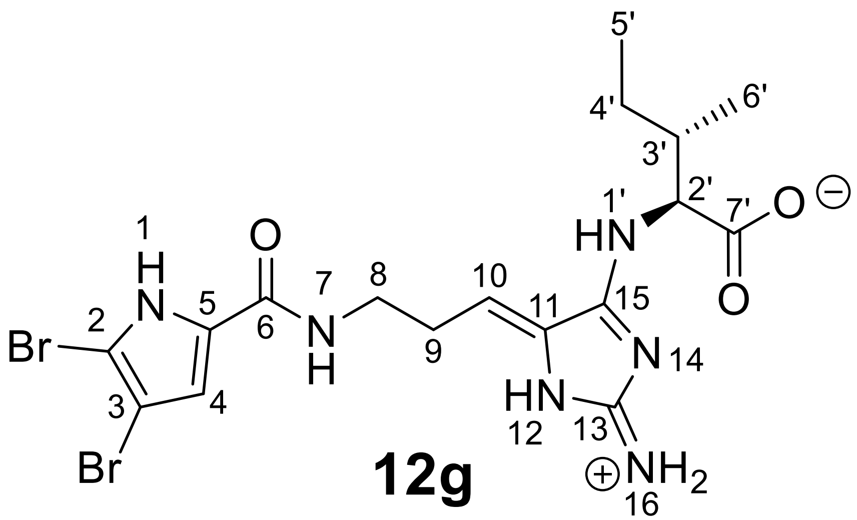

12g

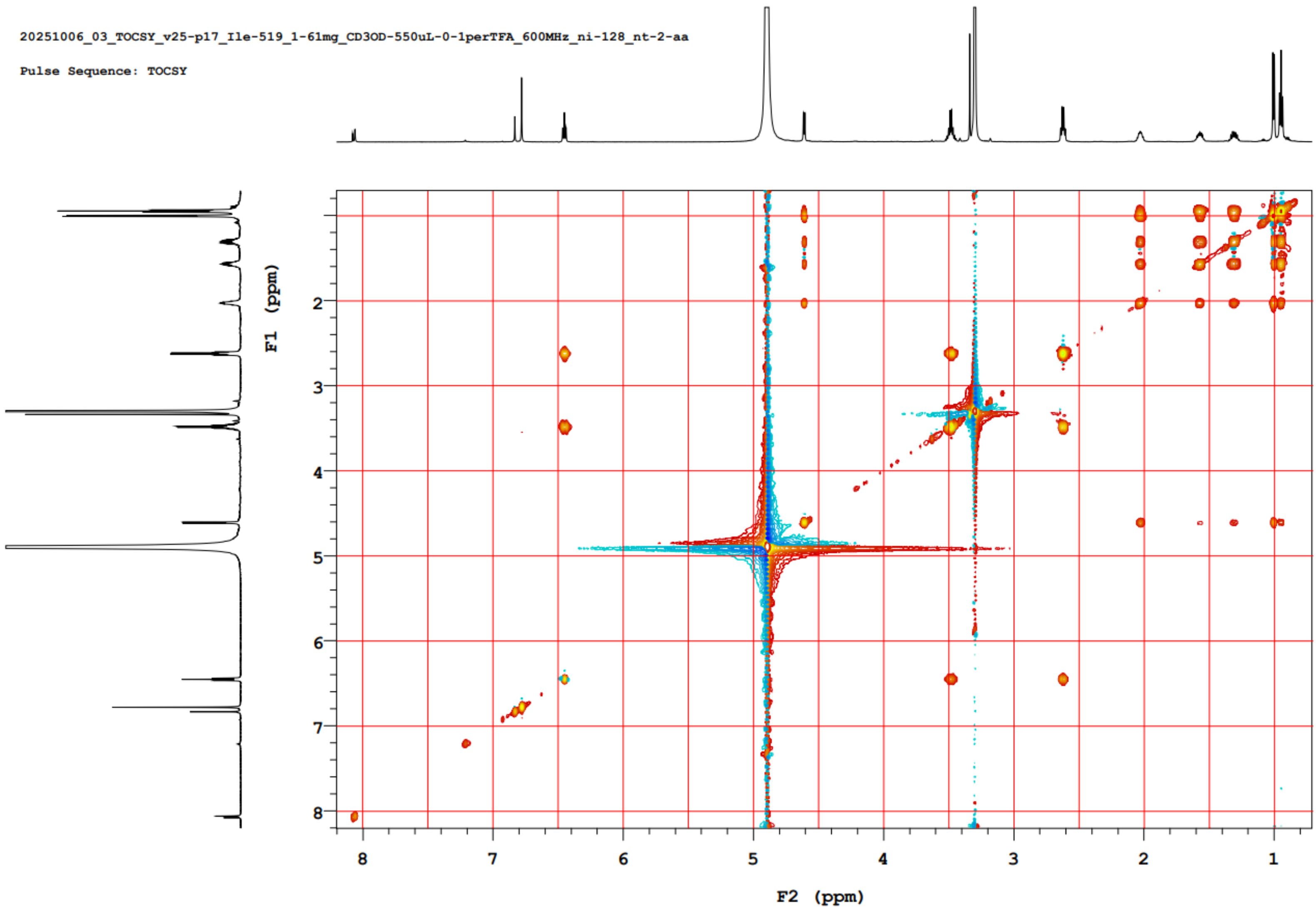

Figure S112. TOCSY spectrum of **12g** (1.61 mg) (600 MHz, CD_3OD: 550 µL - 0.1% TFA).

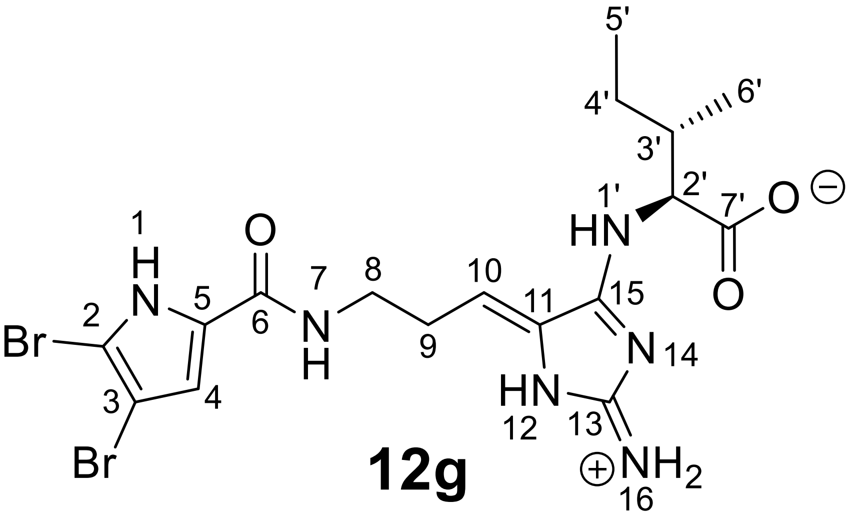

12g

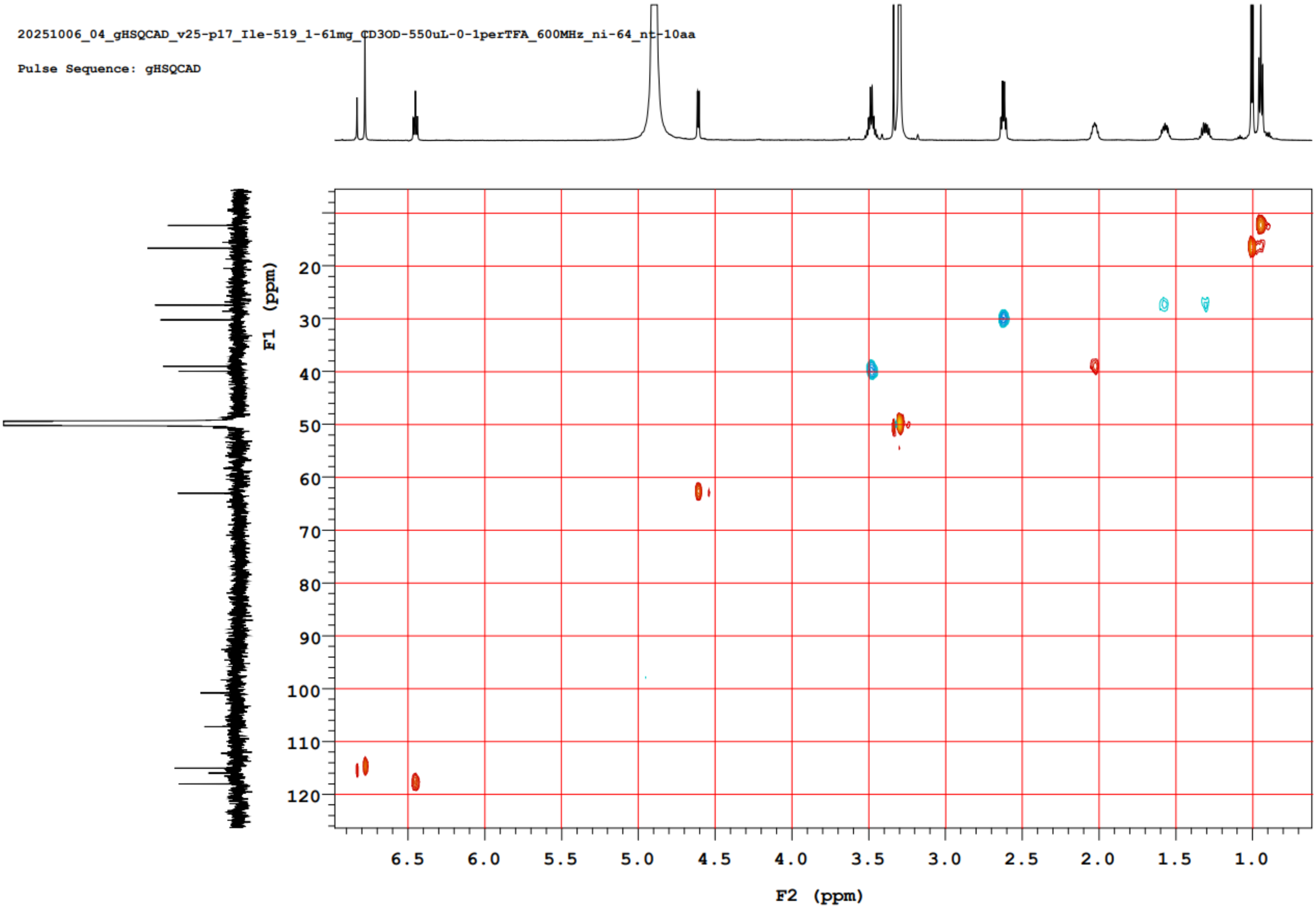

Figure S113. ^{1}H-^{13}C HSQC spectrum of **12g** (1.61 mg) (600 MHz/151 MHz, CD_3OD: 550 µL - 0.1% TFA).

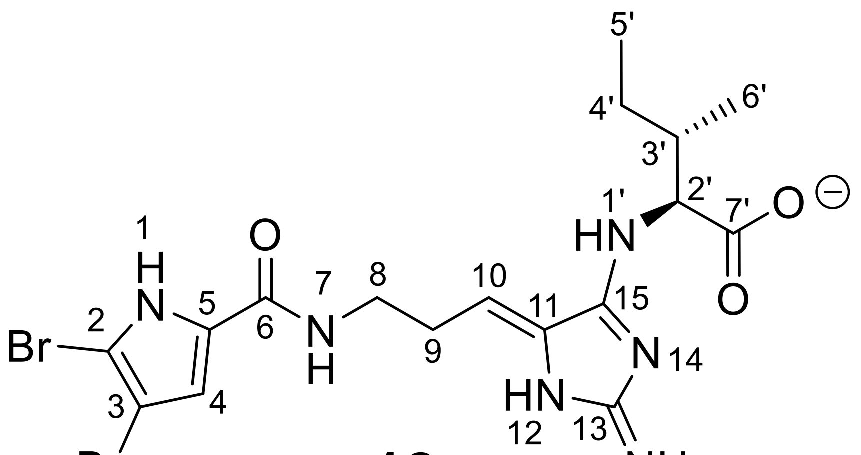

12g

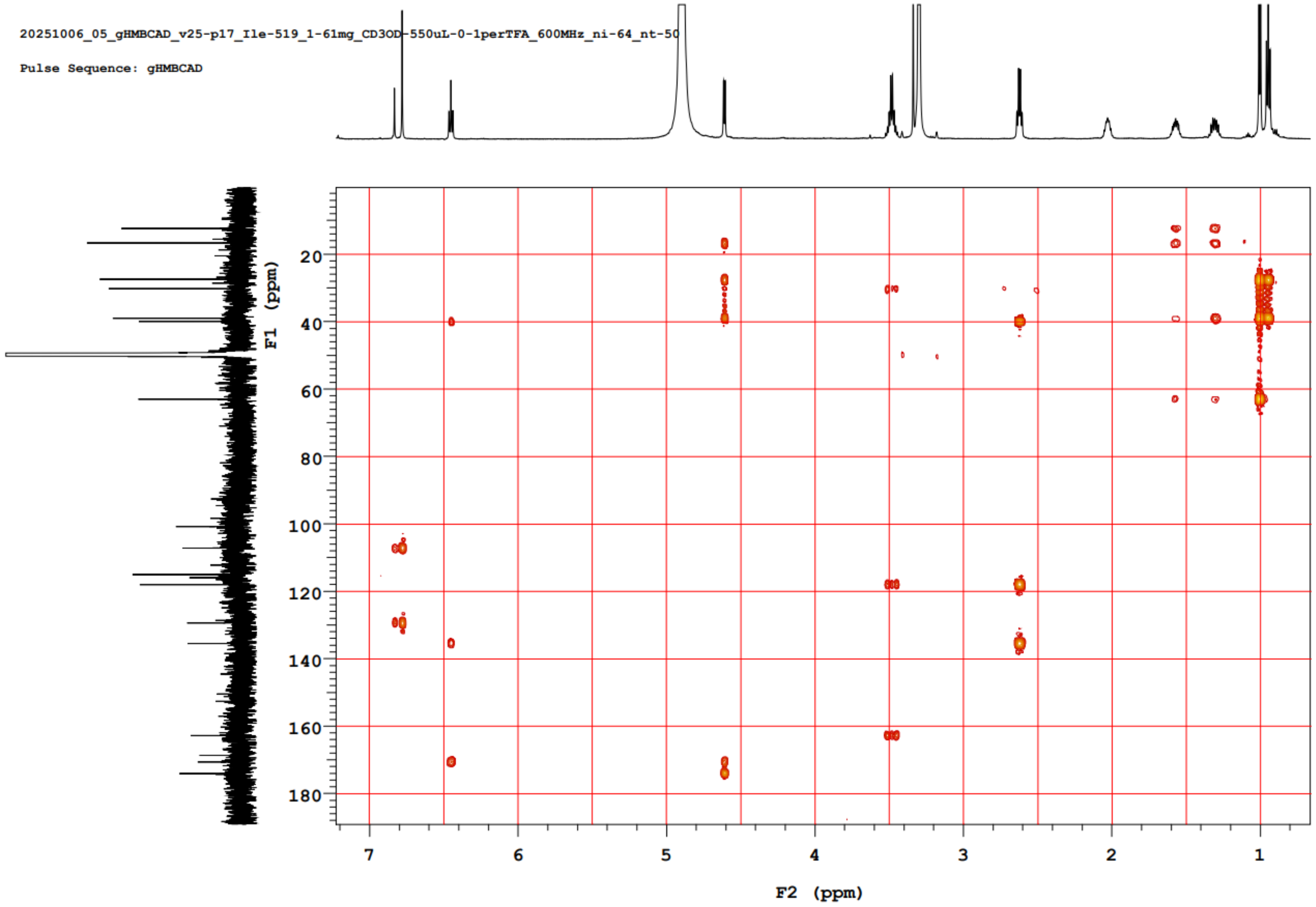

Figure S114. ^{1}H-^{13}C HMBC spectrum of **12g** (1.61 mg) (600 MHz/151 MHz, CD_3OD: 550 µL - 0.1% TFA).

20251025_01_HNMR_v25-p17_Ile-519_1-55mg_DMSO-d6-500uL-0-1perTFA_600MHz_scan20_DMSO-d6_2.50ppm

Pulse Sequence: PROTON (s2pul)

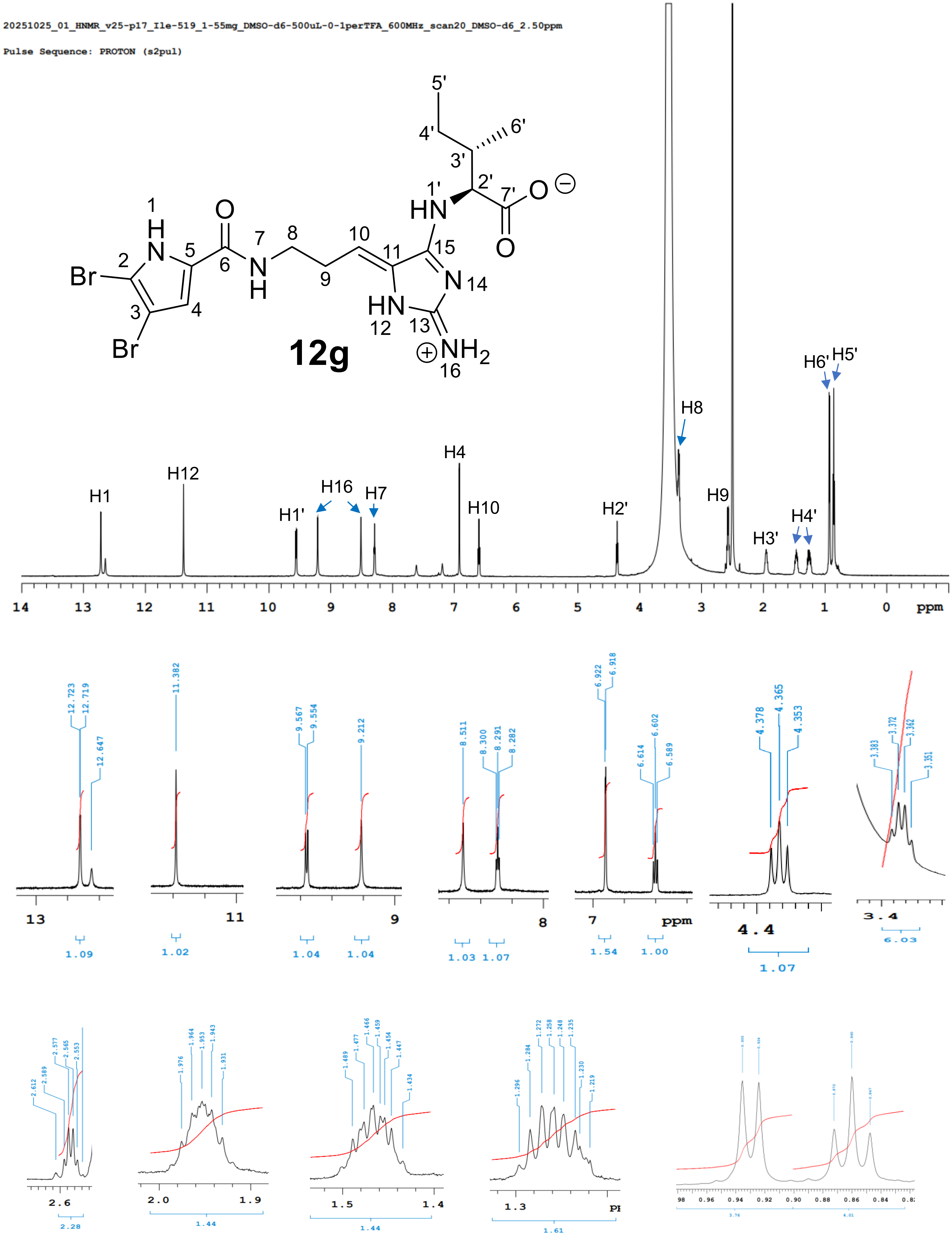

Figure S115. ^{1}H NMR spectrum of **12g** (1.55 mg) (600 MHz, DMSO-d_6: 500 μL - 0.1% TFA).

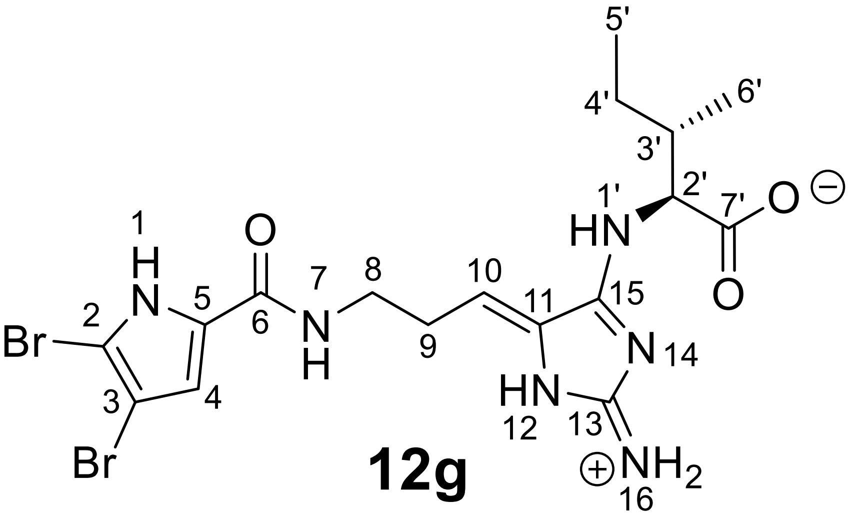

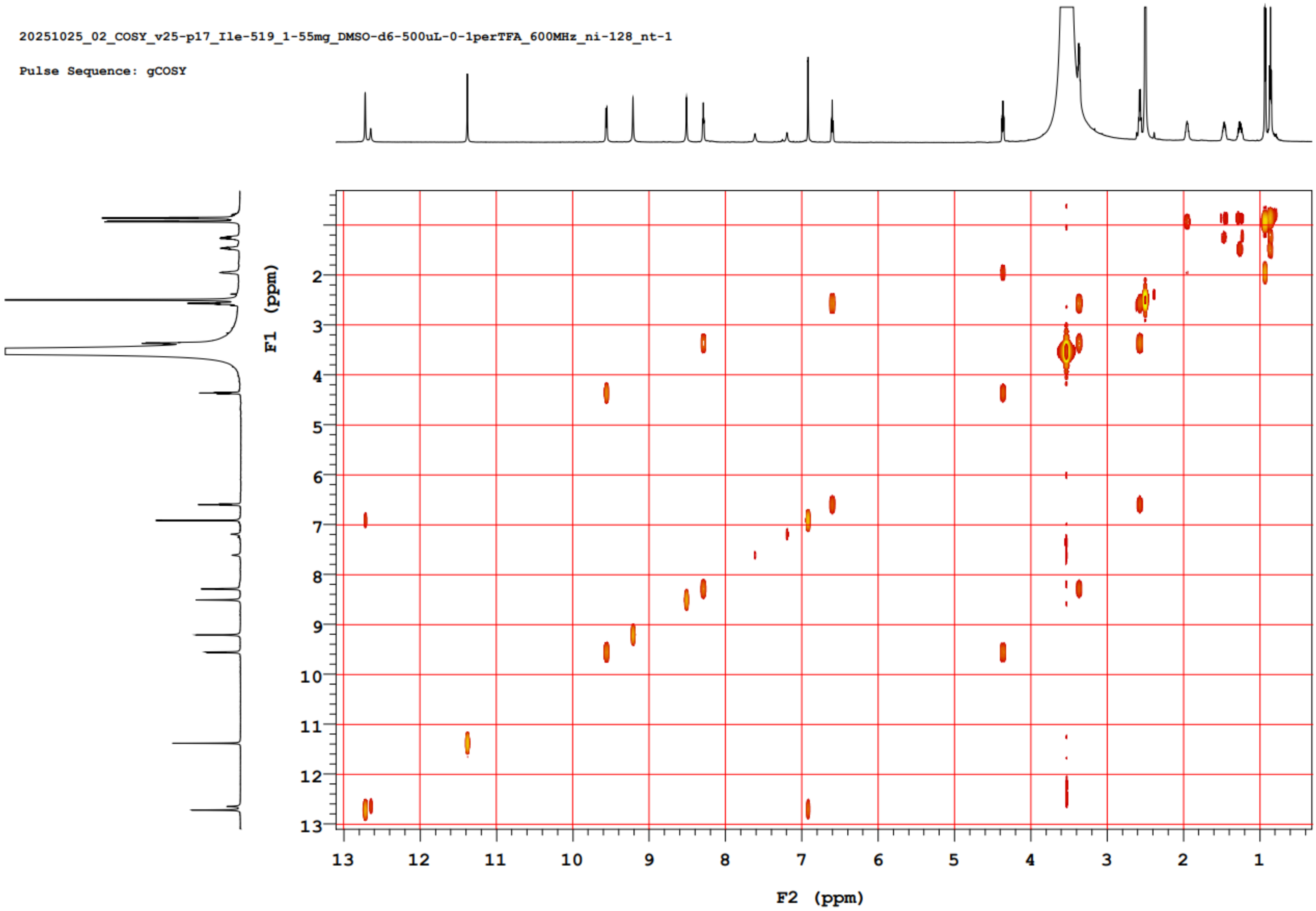

Figure S116. COSY spectrum of **12g** (1.55 mg) (600 MHz, DMSO-d_6: 500 µL - 0.1% TFA).

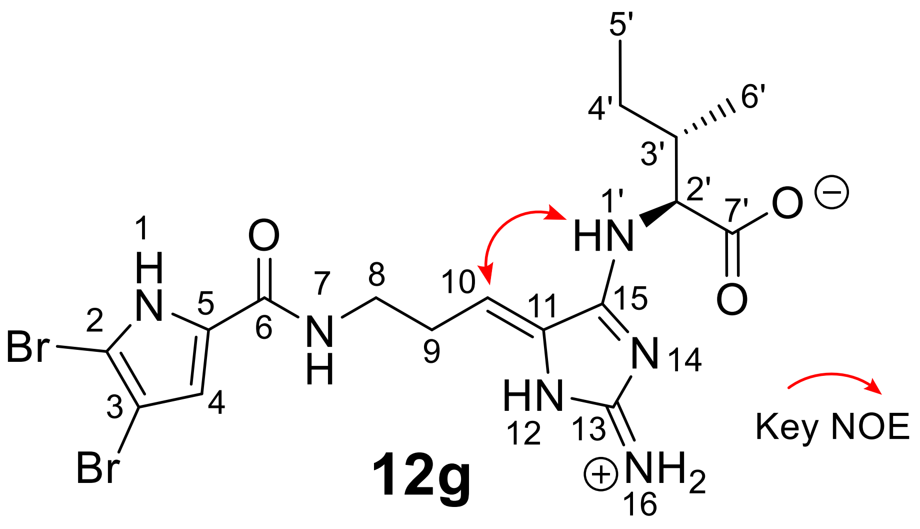

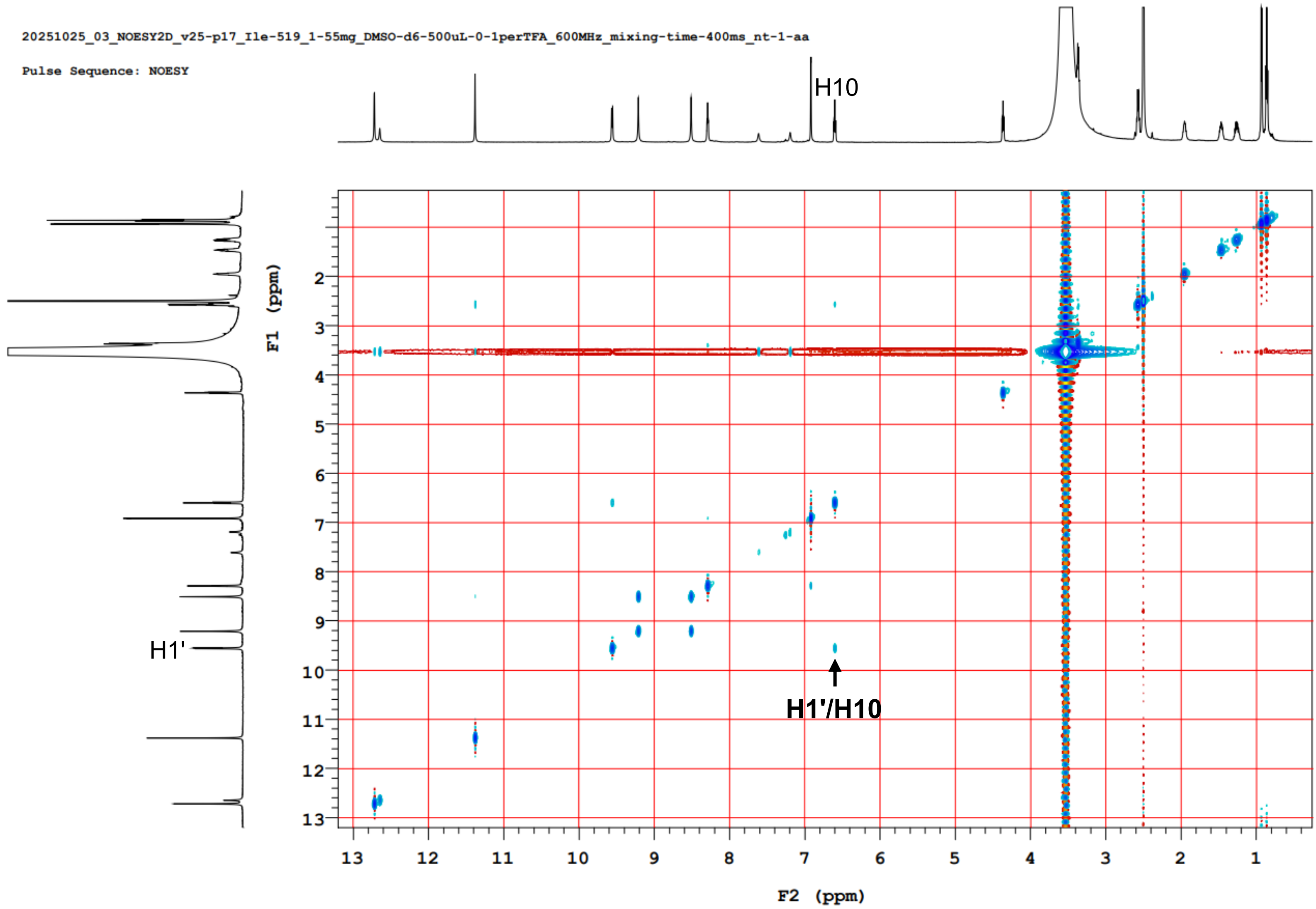

Figure S117. NOESY spectrum of **12g** (1.55 mg) (600 MHz, DMSO-d_6: 500 µL - 0.1% TFA).

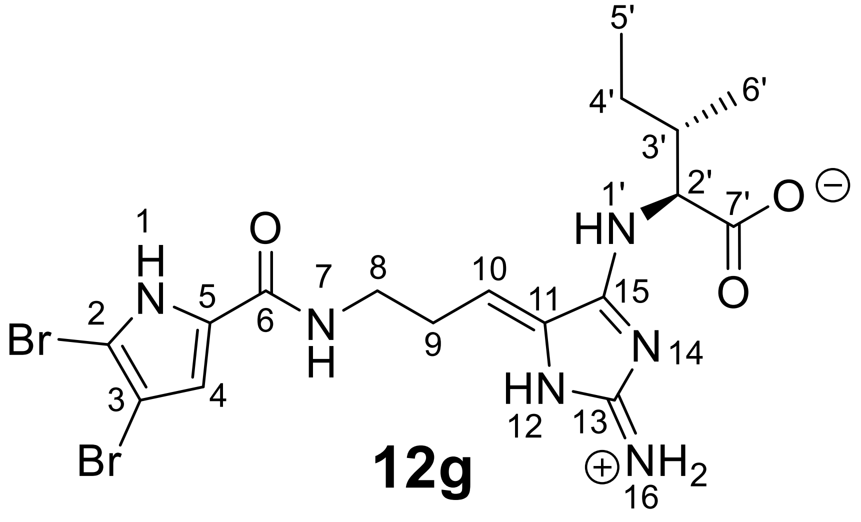

12g

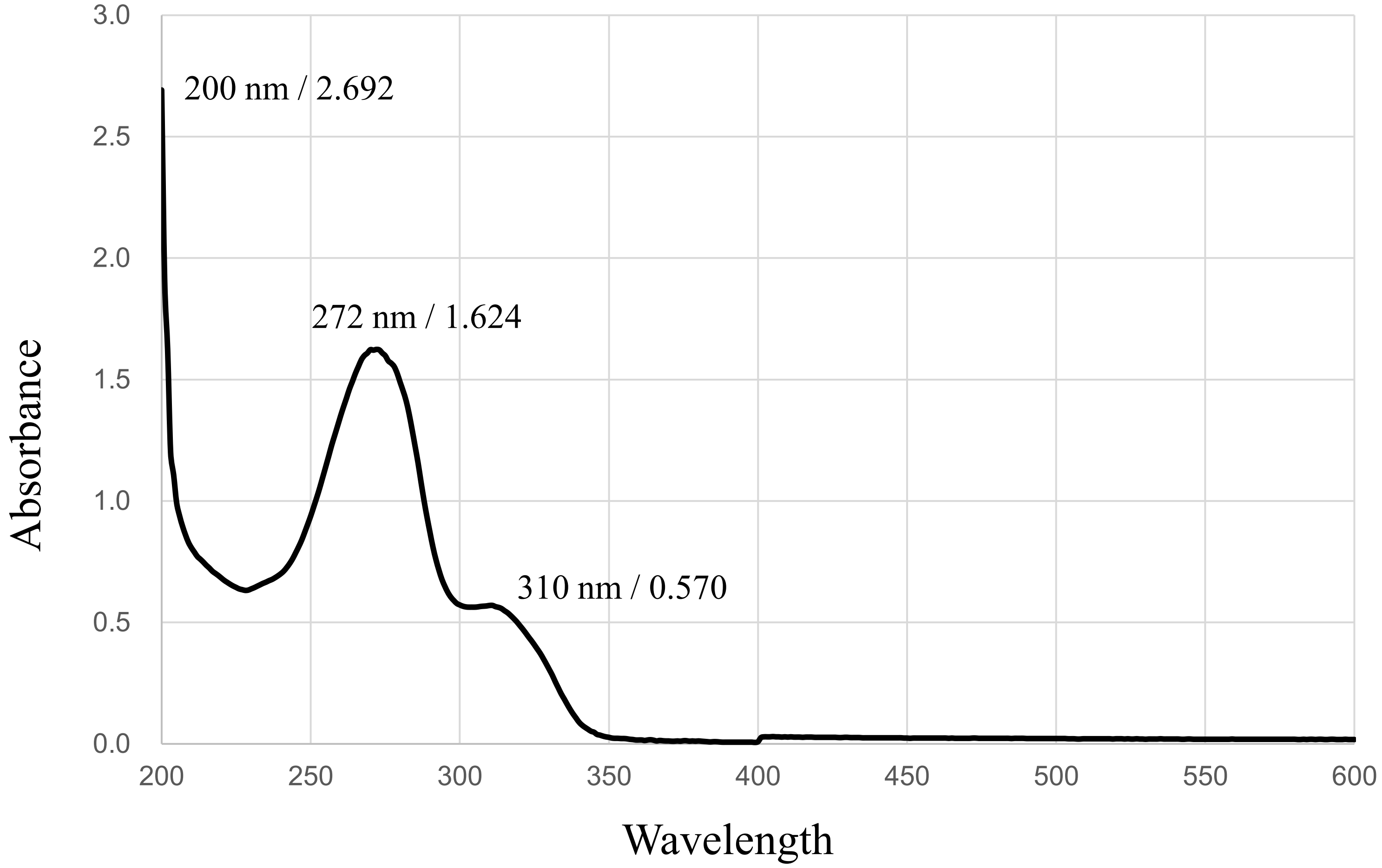

Figure S118. UV absorption spectrum of **12g** (MeOH). c = 6.05 x 10^{-5} (M)

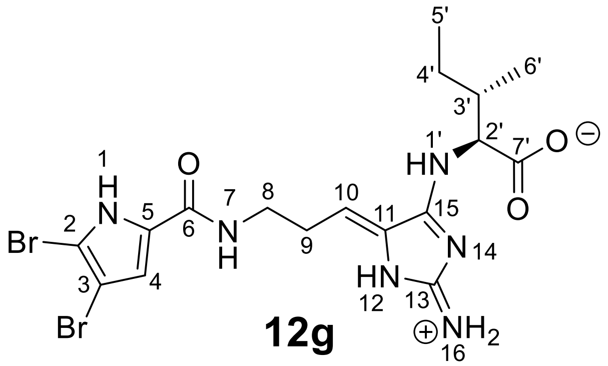

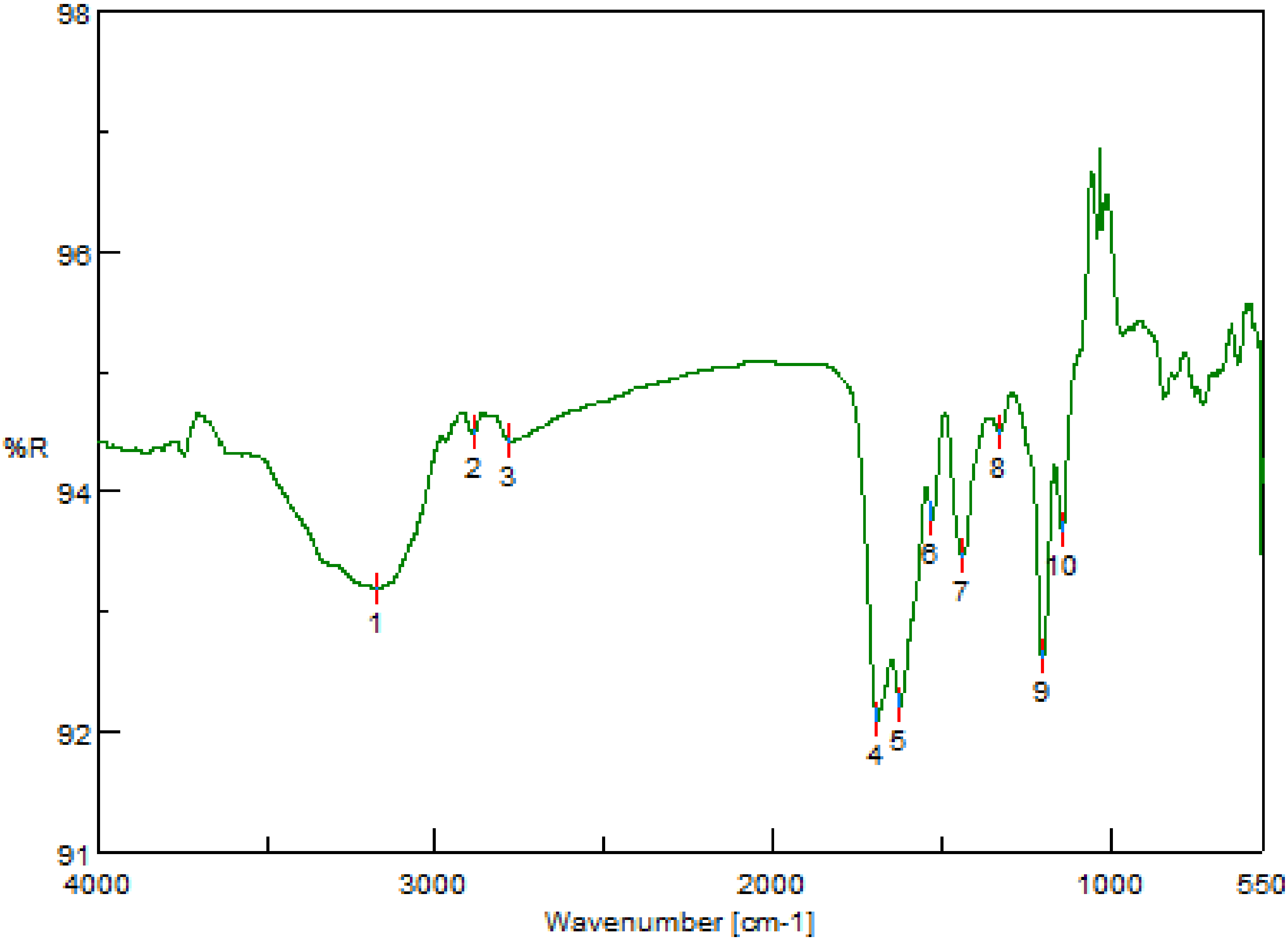

No.	Wavenumber	Strength
1	3175.2	93.1819
2	2888.8	94.4801
3	2778.0	94.4114
4	1692.2	92.0956
5	1622.8	92.2177
6	1529.3	93.7655
7	1437.7	93.4591
8	1327.8	94.4852
9	1204.3	92.6151
10	1143.6	93.6715

Figure S119. IR spectrum of **12g** (ATR).

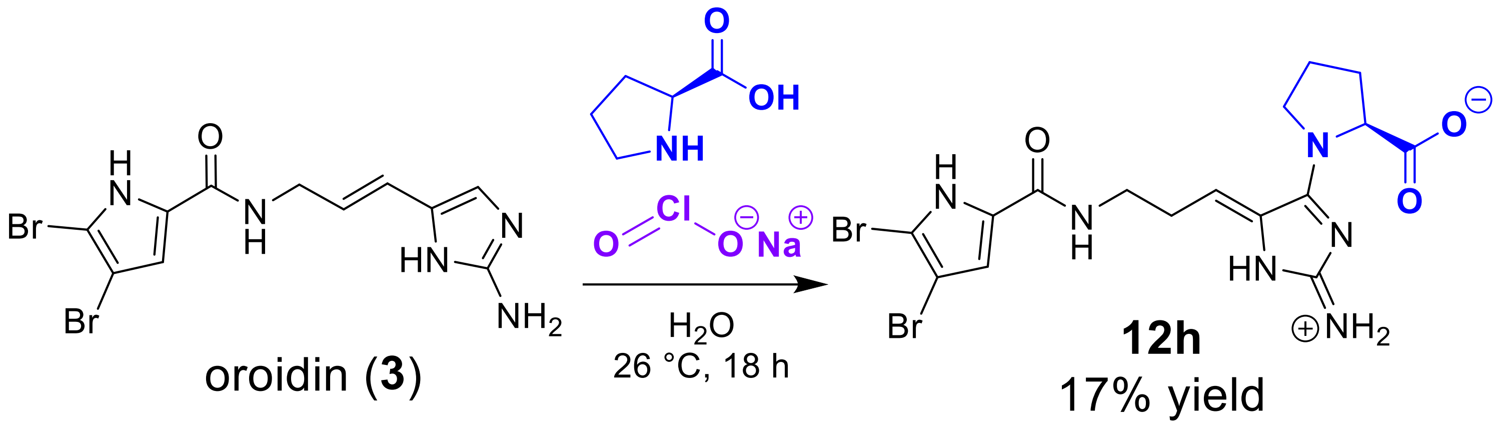

Scheme S8. Synthesis of **12h**.

Six batches of oroidin (**3**) (HCOOH salt, 3.0 mg, 0.0069 mmol each; 18 mg, 0.041 mmol in total) were placed in 20 mL round-bottomed flasks, and H_2O (4.0 mL) was added to each flask with stirring. L-Proline (1.15 g, 10 mmol, 1449 equiv.) was then added to the mixtures, followed by addition of $NaClO_2$ (120 mg, 1.33 mmol, 193 equiv.). The flasks were sealed with septa caps, and the reaction mixtures were stirred at 26 °C for 18 h. After completion, the mixtures were combined and filtered through a small pad of Celite, rinsing the flasks and filter cake with H_2O. The filtrate was directly purified by ODS silica gel column chromatography (MeOH/H_2O, 30:70 to 55:45, v/v). The eluate was concentrated under reduced pressure, and the crude material was filtered through a Cosmospin filter H (0.45 μm). Further purification was performed by RP-HPLC (InertSustain AQ-C18, 5 μm, 10 mm i.d. × 250 mm; GL Science) using gradient elution (0–4 min, MeOH/H_2O = 3:97 to 55:45, v/v; 4 min-, 55:45) at a flow rate of 2.0 mL/min. Semi-pure **12h** was obtained at 27-37 min and was further purified by RP-HPLC (InertSustain AQ-C18, 5 μm, 10 mm i.d. × 250 mm; GL Science) again using gradient elution (0–4 min, MeOH/H_2O/HCOOH = 3:97:0.1 to 55:45:0.1, v/v; 4 min-, 55:45:0.1) at a flow rate of 2.0 mL/min. Pure **12h** was obtained at 19-26 min (3.43 mg, 0.0068 mmol, 17% yield) as an off white solid.

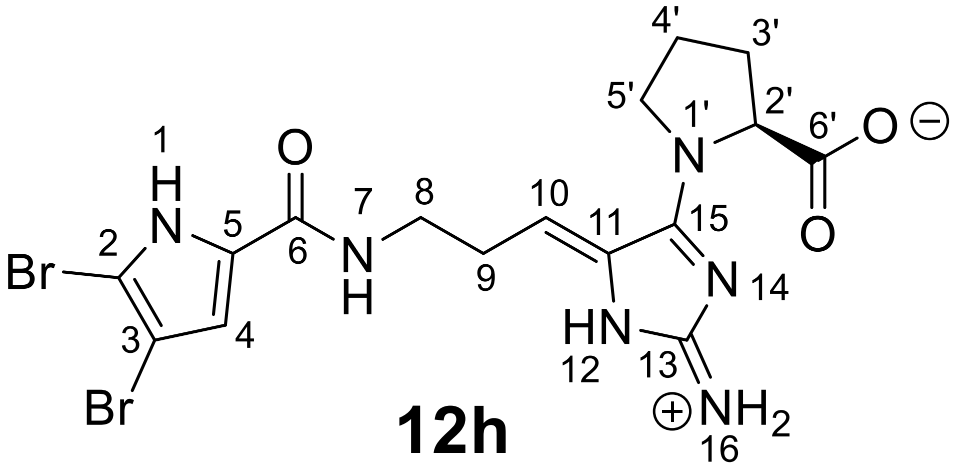

12h

12h:

$\mathbf{R_f}$ = 0.18 ($CHCl_3$/MeOH/28% NH_3 aq. = 60:40:2, v/v/v; UV).

$\mathbf{[\alpha]_D^{25}}$: −9.6 (c = 1.69 x 10^{-3}, MeOH).

UV/vis λ_{max} (MeOH) nm (log ε): 311 (4.23), 276 (4.68), 200 (4.75).

^{1}H NMR (600 MHz, CD_3OD containing 0.4% TFA): *δ* 6.77 (s, C4-H, 1H), 6.26 (t, *J* = 7.2 Hz, C10-H, 1H), 4.82 (dd, *J* = 9.0, 2.4 Hz, C2'-H, 1H), 3.91 (m, C5'-H, 1H), 3.80 (m, C5'-H, 1H), 3.54 (m, C8-H, 2H), 2.67 (q, *J* = 6.8 Hz, C9-H, 2H), 2.36 (m, C3'-H, 1H), 2.20 (m, C3'-H, 1H), 2.20 (m, C4'-H, 2H).

^{13}C NMR (151 MHz, CD_3OD containing 0.4% TFA): *δ* 174.6 (C6'), 167.1 (C15), 167.1 (C13), 162.9 (C6), 136.1 (C11), 129.4 (C5), 124.0 (C10), 115.0 (C4), 107.2 (C2), 100.8 (C3), 66.2 (C2'), 52.0 (C5'), 39.8 (C8), 31.4 (C9), 30.6 (C3'), 26.6 (C4').

HRMS (**ESI**): (*m/z*) calcd for $C_{16}H_{19}{}^{79}Br_2N_6O_3^+$ $[M+H]^+$: 500.9880, found 500.9871.

IR ν_{max}: 3165 (br), 1691 (s), 1614 (s), 1529 (w), 1398 (w), 1329 (w), 1198 (s), 1144 (m).

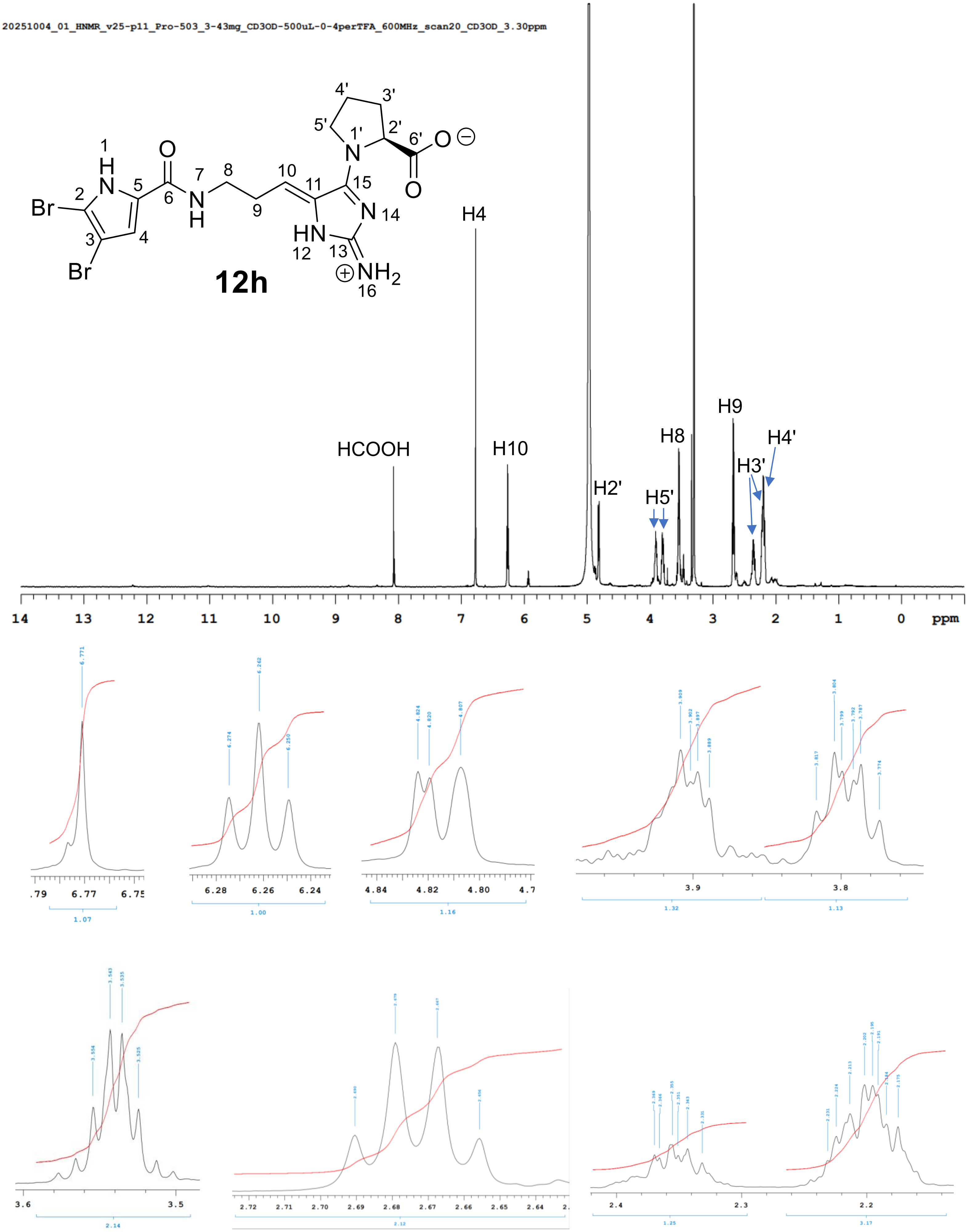

Figure S120. ^{1}H NMR spectrum of **12h** (3.43 mg) (600 MHz, CD_3OD: 500 µL - 0.4% TFA).

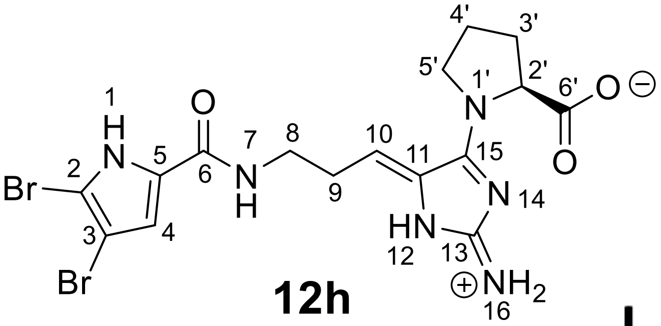

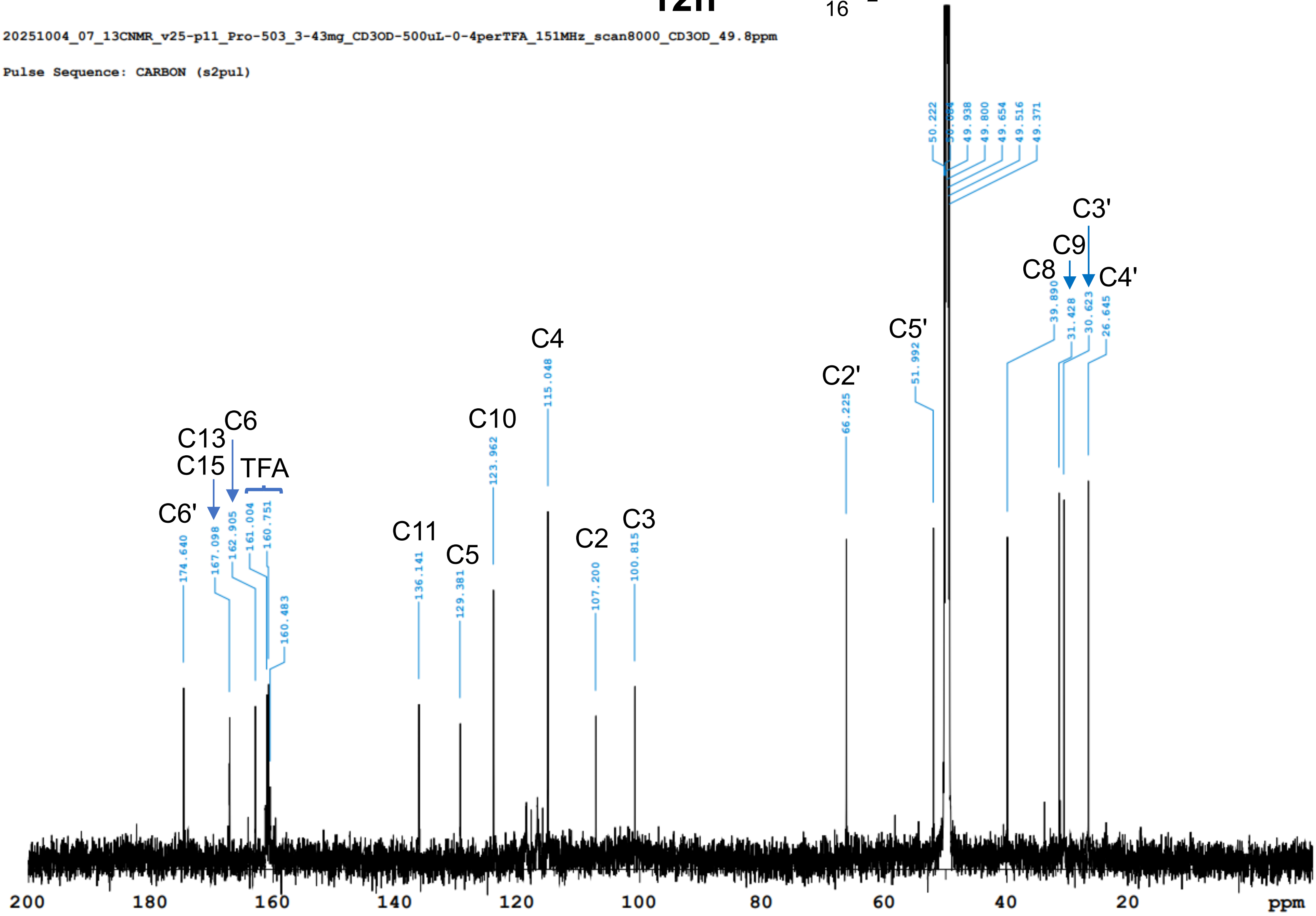

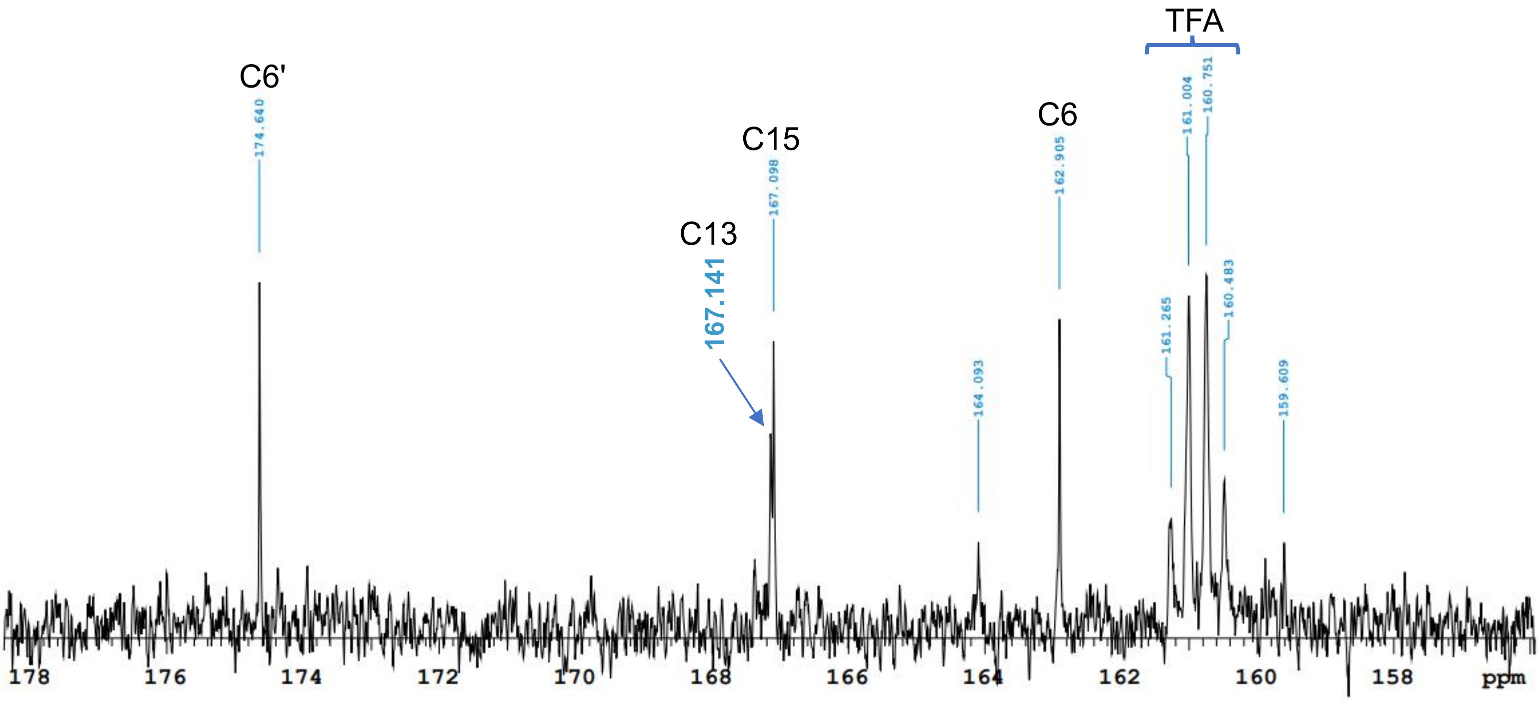

Figure S121. ^{13}C NMR spectrum of **12h** (3.43 mg) (151 MHz, CD_3OD: 500 µL - 0.4% TFA).

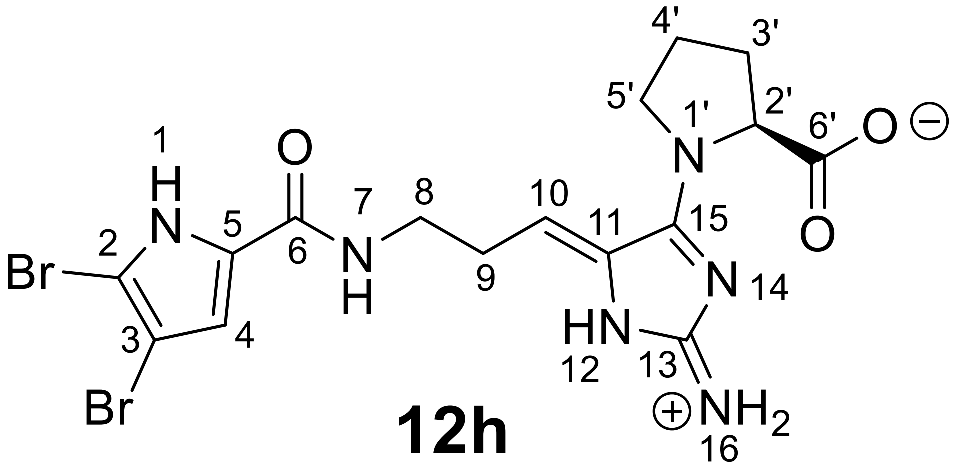

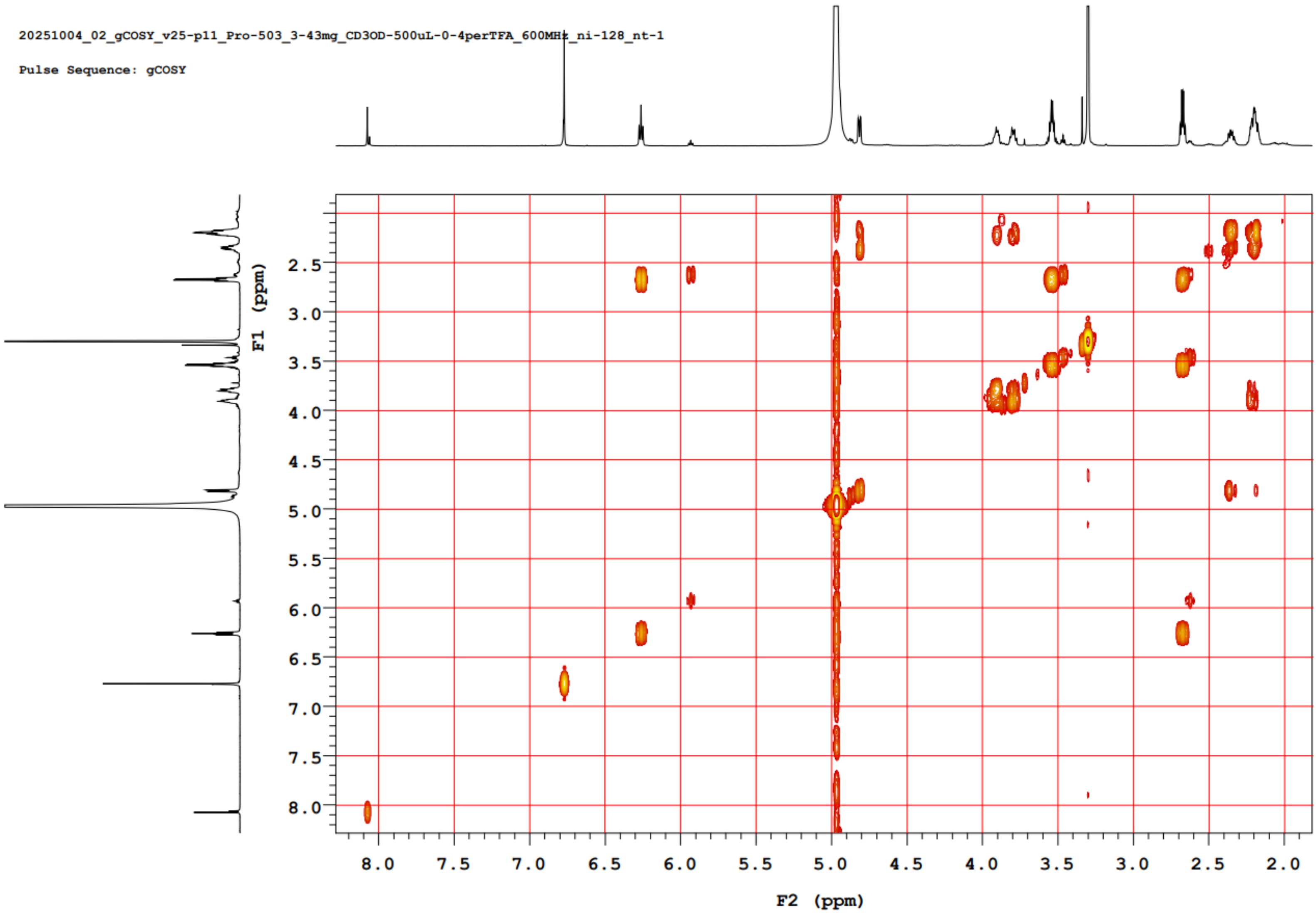

Figure S122. COSY spectrum of **12h** (3.43 mg) (600 MHz, CD_3OD: 500 µL - 0.4% TFA).

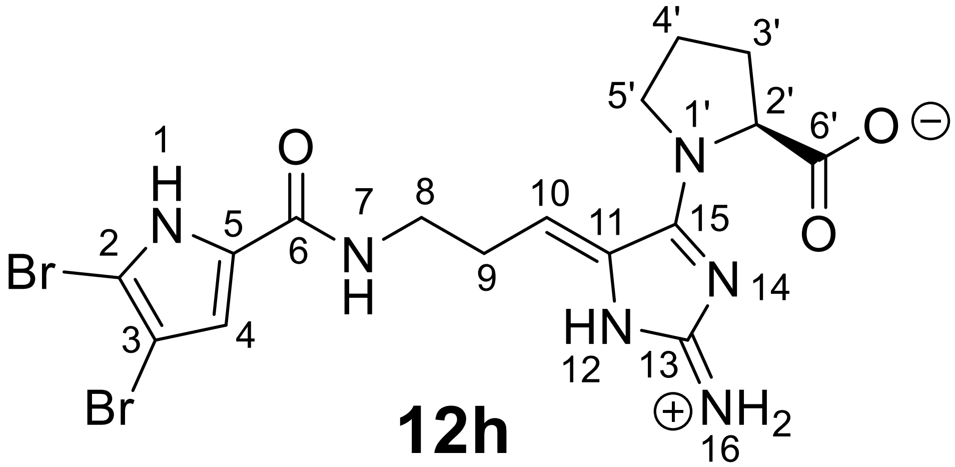

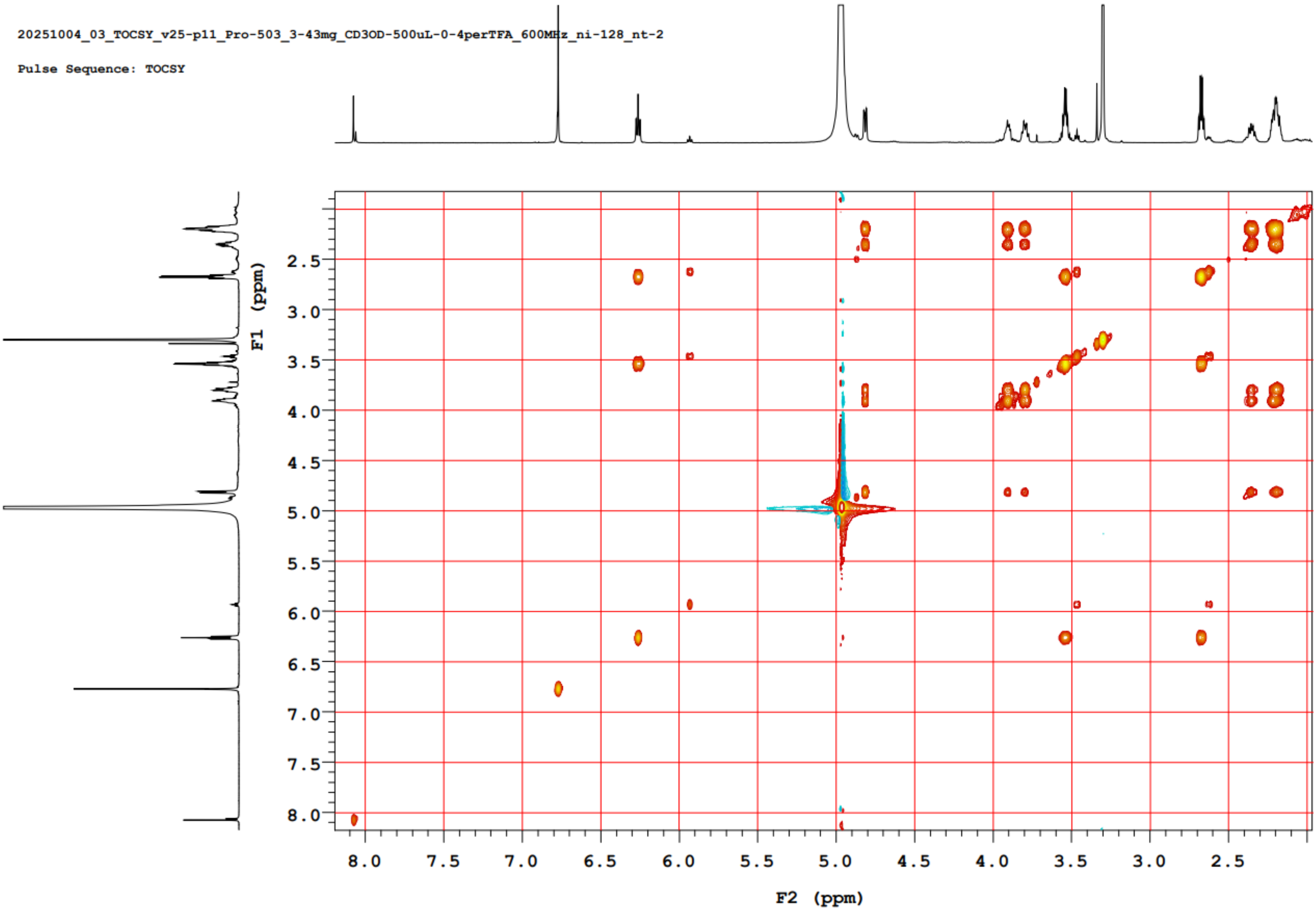

Figure S123. TOCSY spectrum of **12h** (3.43 mg) (600 MHz, CD_3OD: 500 µL - 0.4% TFA).

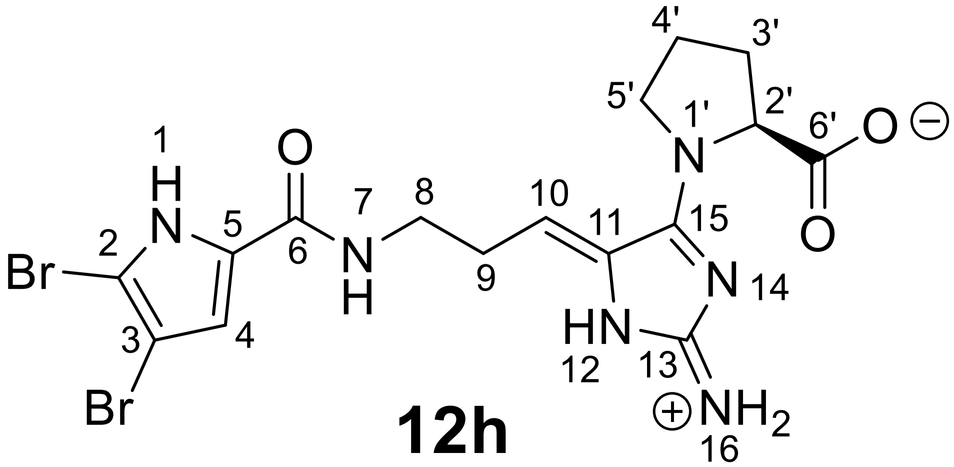

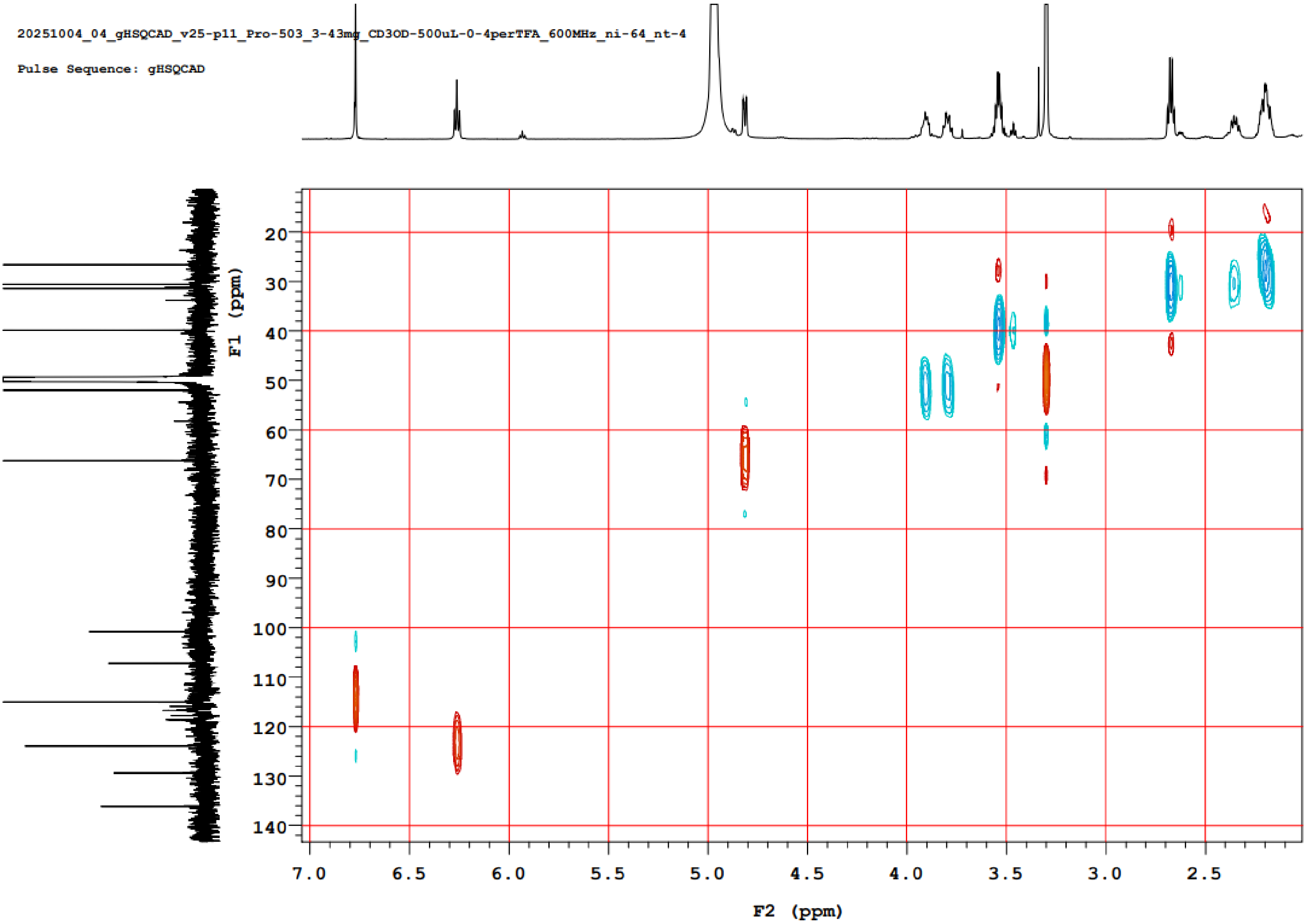

Figure S124. ^{1}H-^{13}C HSQC spectrum of **12h** (3.43 mg) (600 MHz, CD_3OD: 500 µL - 0.4% TFA).

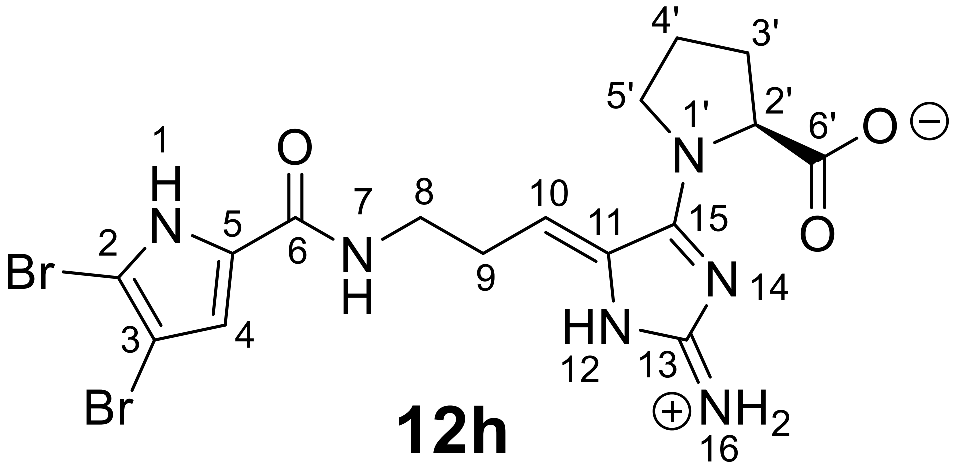

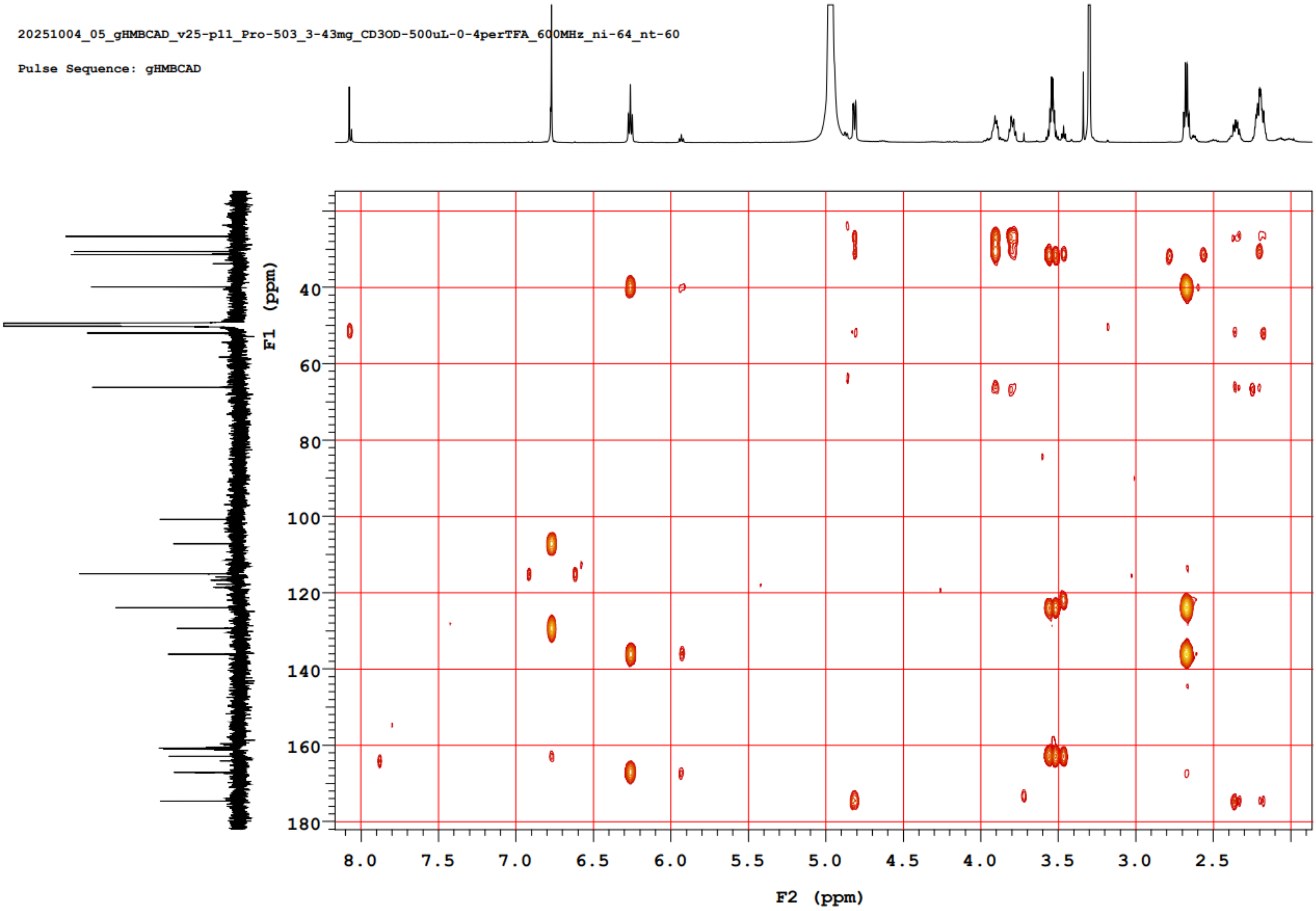

Figure S125. ^{1}H-^{13}C HMBC spectrum of **12h** (3.43 mg) (600 MHz, CD_3OD: 500 μL - 0.4% TFA).

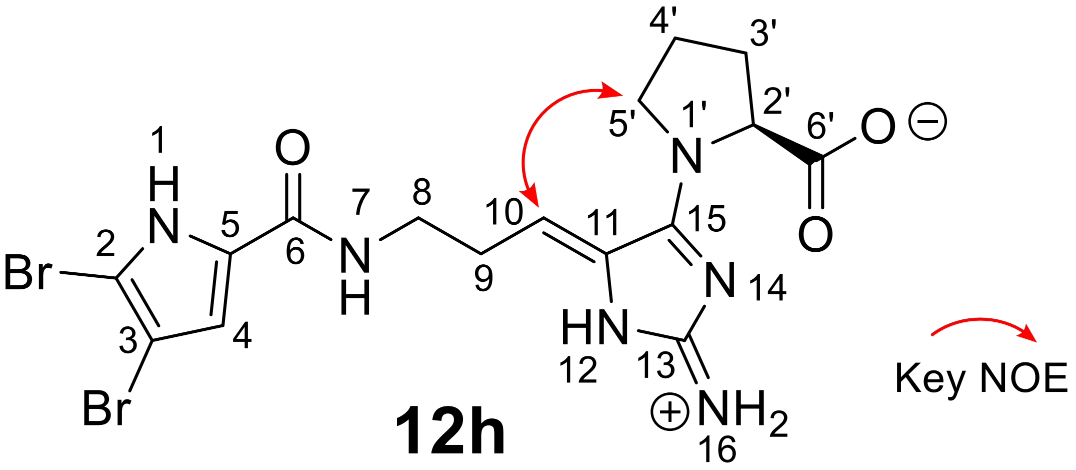

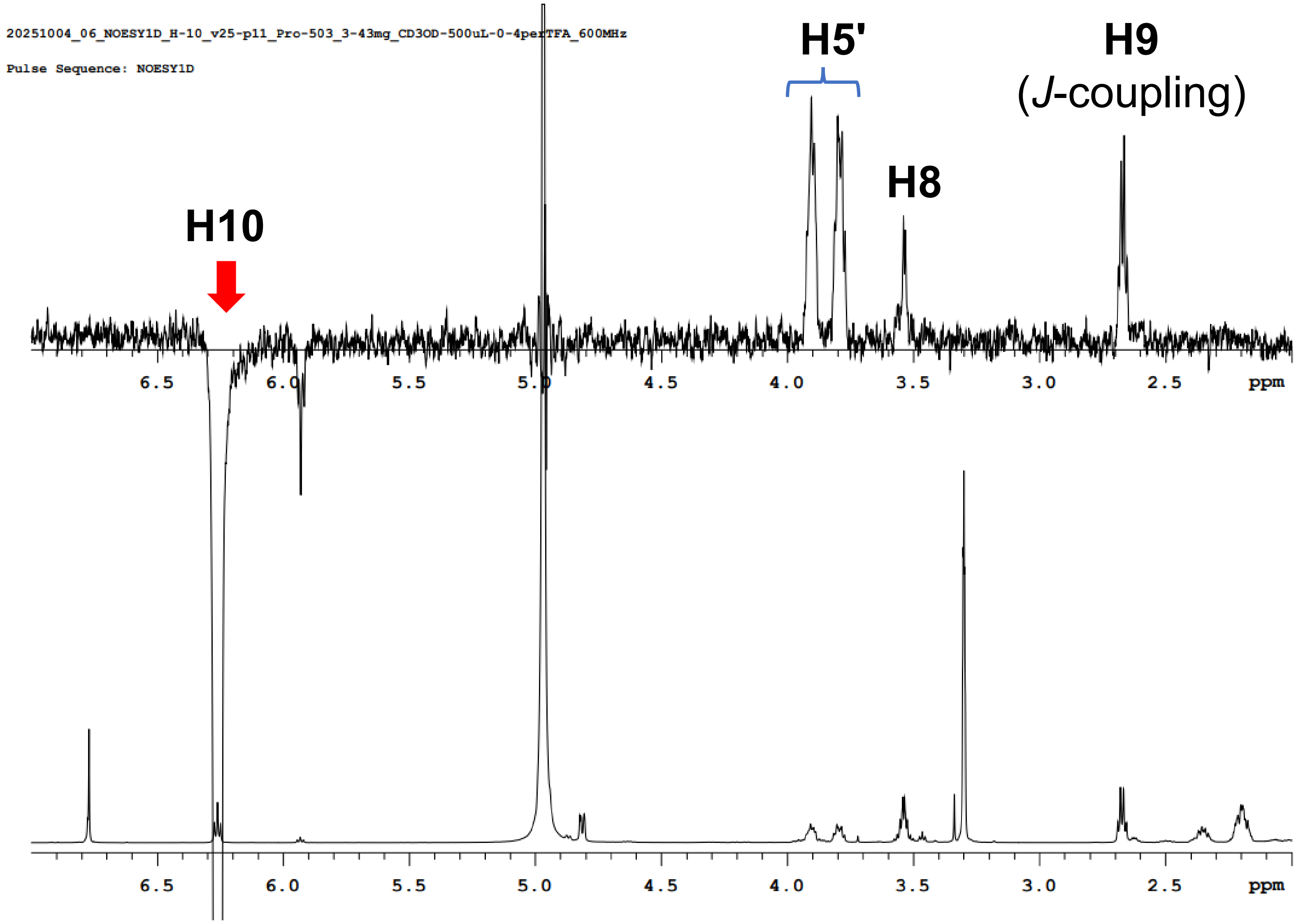

Figure S126. NOESY-1D spectrum of **12h** (3.43 mg); irradiation at 6.26 ppm (600 MHz, CD_3OD: 500 µL - 0.4% TFA).

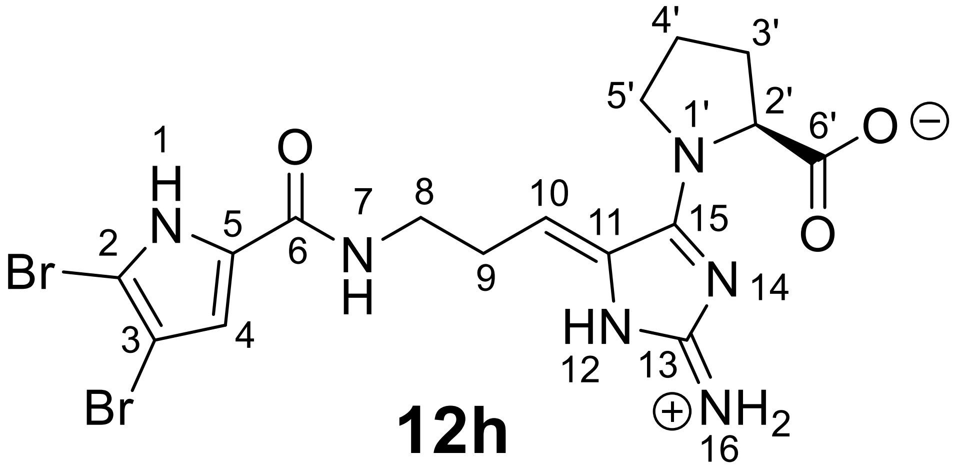

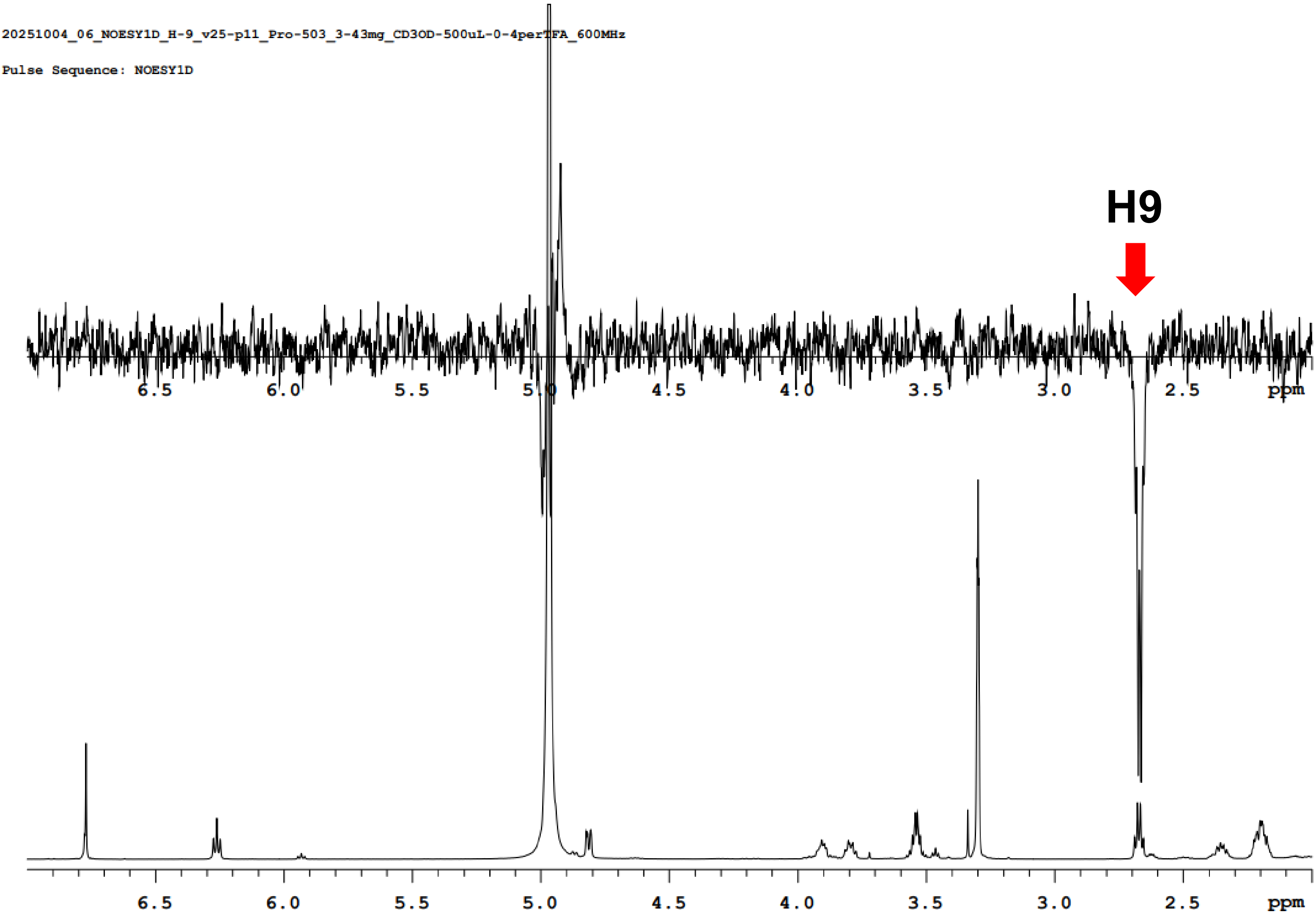

Figure S127. NOESY-1D spectrum of **12h** (3.43 mg); irradiation at 2.67 ppm (600 MHz, CD_3OD: 500 μL - 0.4% TFA).

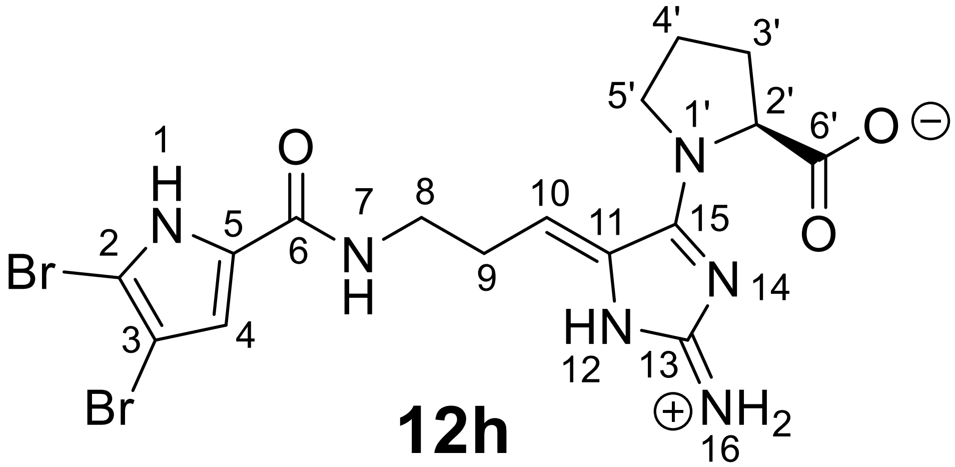

12h

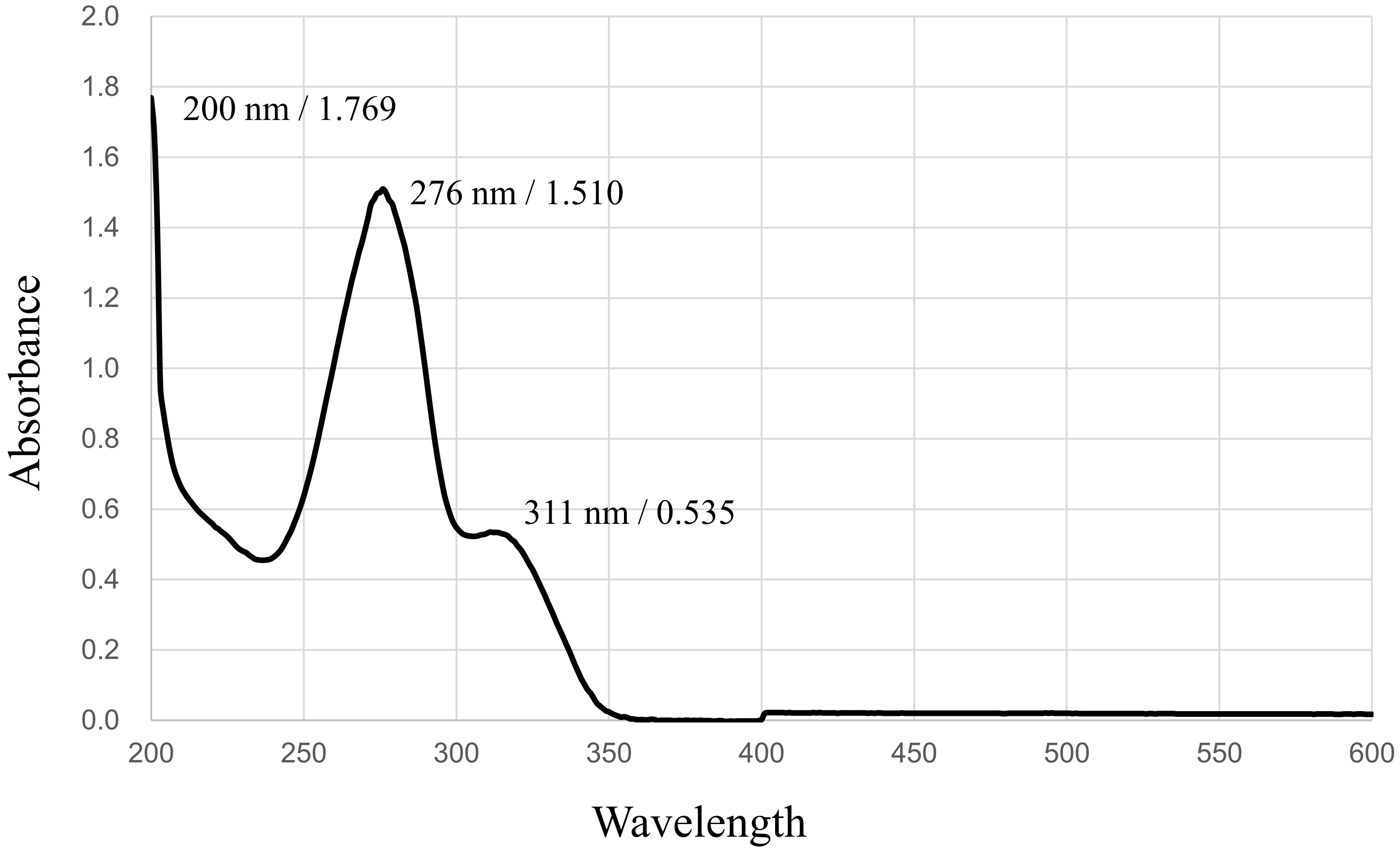

Figure S128. UV absorption spectrum of **12h** (MeOH). c = 3.12 x 10^{-5} (M)

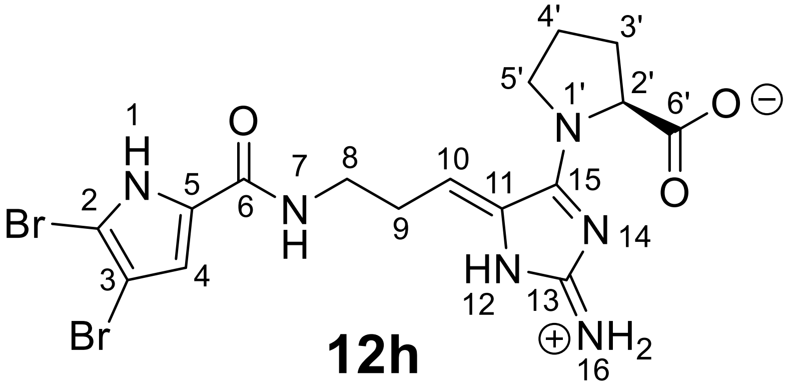

12h

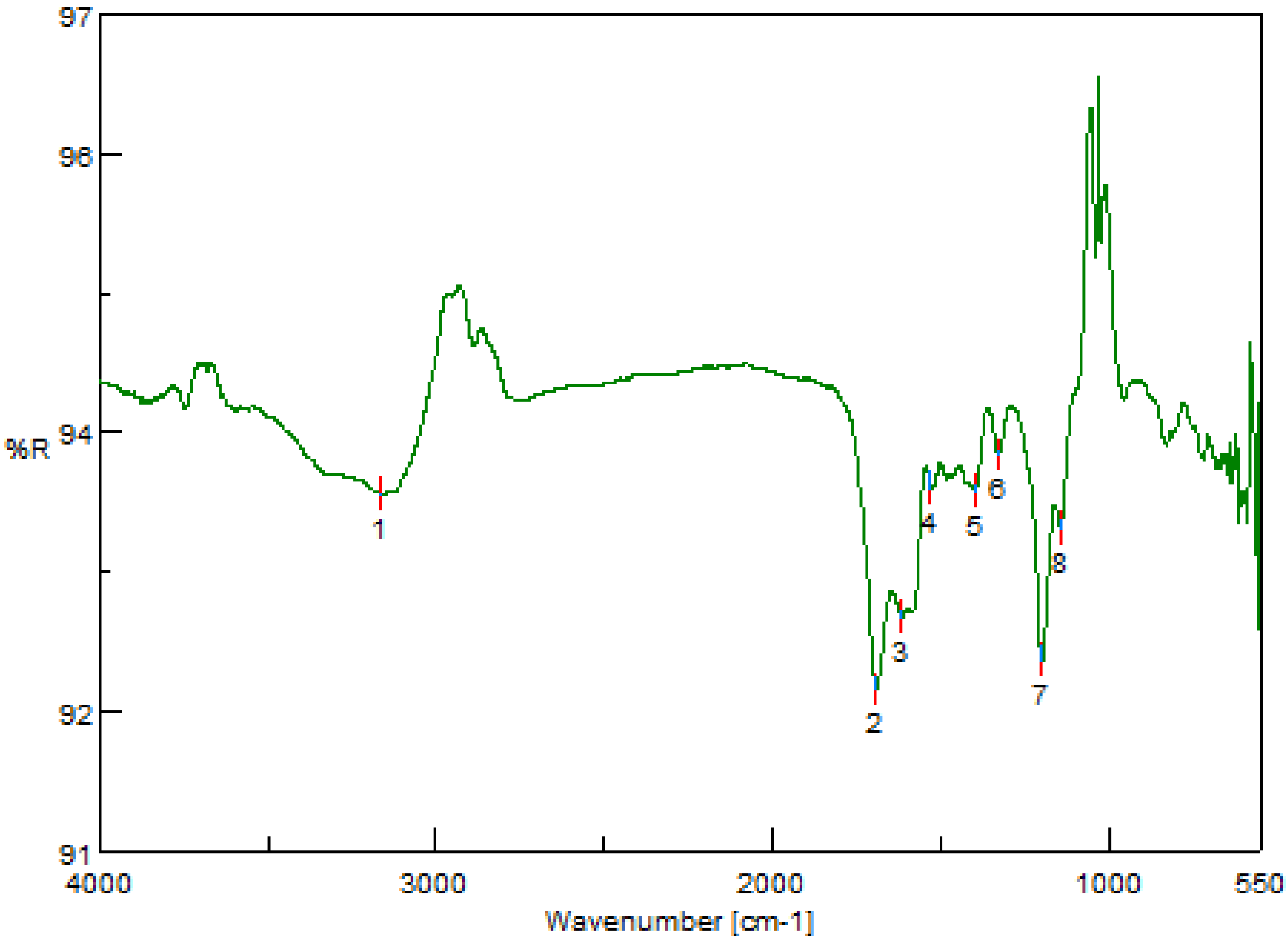

No.	Wavenumber	Strength
1	3164.6	93.556
2	1691.3	92.155
3	1614.1	92.68
4	1529.3	93.5966
5	1398.1	93.581
6	1328.7	93.8337
7	1197.6	92.3669
8	1143.6	93.3068

Figure S129. IR spectrum of **12h** (ATR).

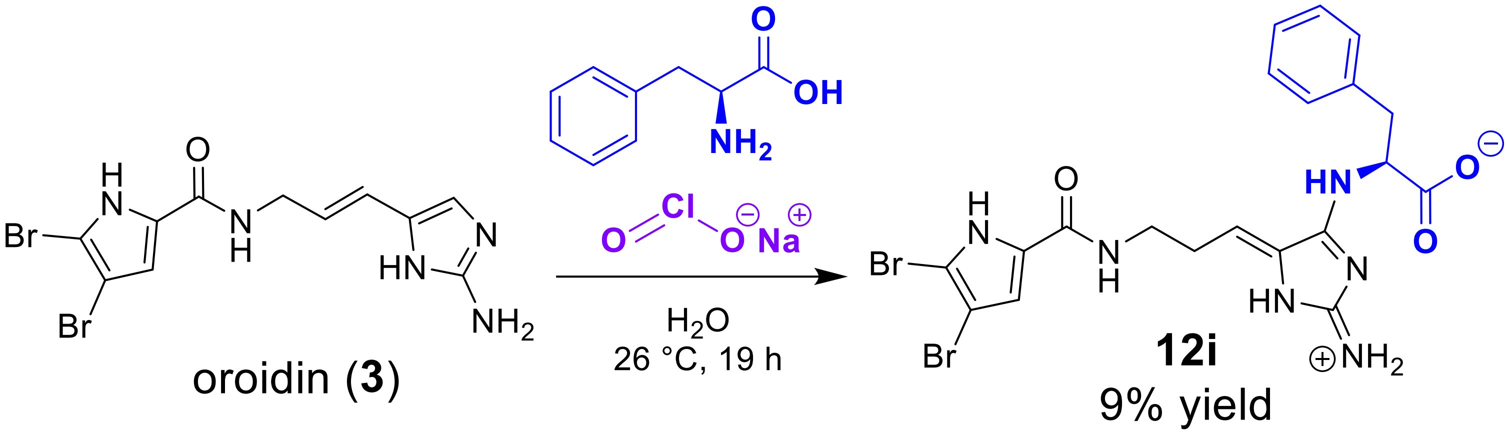

Scheme S9. Synthesis of **12i**.

Five batches of oroidin (**3**) (HCOOH salt, 3.0 mg, 0.0069 mmol each; 15 mg, 0.034 mmol in total) were placed in 20 mL round-bottomed flasks, and H_2O (4.0 mL) was added to each flask with stirring. L-Phenylalanine (0.99 g, 6.0 mmol, 870 equiv.) was then added to the mixtures, followed by addition of $NaClO_2$ (120 mg, 1.33 mmol, 193 equiv.). The flasks were sealed with septa caps, and the reaction mixtures were stirred at 26 °C for 19 h. After completion, the mixtures were combined and filtered through a small pad of Celite, rinsing the flasks and filter cake with H_2O. The filtrate was directly purified by ODS silica gel column chromatography (MeOH/H_2O, 50:50 to 70:30, v/v). The eluate was concentrated under reduced pressure, and the crude material was filtered through a Cosmospin filter H (0.45 µm). Further purification was performed by RP-HPLC (InertSustain AQ-C18, 5 µm, 10 mm i.d. × 250 mm; GL Science) using gradient elution (0–4 min, MeOH/H_2O/HCOOH = 3:97:0.1 to 60:40:0.1, v/v; 4 min–, 60:40:0.1) at a flow rate of 2.0 mL/min. Pure **12i** was obtained at 26-31 min (1.77 mg, 0.0032 mmol, 9% yield) as an off white solid.

12i:

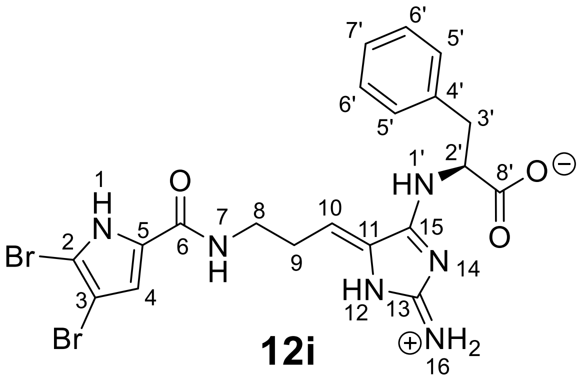

12i

$\mathbf{R_f}$ = 0.26 ($CHCl_3$/MeOH/28%NH_3 aq. = 60:40:2, v/v/v; UV).

$\mathbf{[\alpha]_D^{25}}$: −18.7 (c = 8.5 x 10^{-4}, MeOH).

UV/vis λ_{max} (MeOH) nm (log ε): 310 (3.79), 270 (4.27), 200 (4.33).

^{1}H NMR (600 MHz, CD_3OD containing 0.1% TFA): δ 7.21 (m, C5'-H, C6'-H, C7'-H, 5H), 6.79 (s, C4-H, 1H), 6.24 (t, J = 7.8 Hz, C10-H, 1H), 4.94 (m, C2'-H, 1H), 3.46 (m, C8-H, 2H), 3.38 (dd, J = 14.4, 4.2 Hz, C3'-H, 1H), 3.08 (dd, J = 13.8, 10.2 Hz, C3'-H, 1H), 2.59 (q, J = 7.2 Hz, C9-H, 2H).

^{1}H NMR (600 MHz, DMSO-d_6 containing 0.1% TFA): δ 12.73 (d, J = 1.8 Hz, N1-H, 1H), 11.35 (s, N12-H, 1H), 9.87 (d J = 8.4 Hz, N1'-H, 1H), 9.21 (s, N16-H, 1H), 8.48 (s, N16-H, 1H), 8.30 (t, J = 5.4 Hz, N7-H, 1H), 7.25 (m, C5'-H, C6'-H, C7'-H, 5H), 6.94 (d, J = 2.4 Hz, C4-H, 1H), 6.40 (t, J = 7.5 Hz, C10-H, 1H), 4.66 (m, C2'-H, 1H), 3.36 (m, C8-H, 2H), 3.25 (dd, J = 14.4, 4.2 Hz, C3'-H, 1H), 3.08 (dd, J = 13.8, 10.2 Hz, C3'-H, 1H), 2.55 (q, J = 7.0 Hz, C9-H, 2H).

^{13}C NMR (151 MHz, CD_3OD containing 0.1% TFA): δ 173.9 (C8'), 170.4 (C15), 168.6 (C13), 162.8 (C6), 138.7 (C4'), 135.4 (C11), 130.9 (C5'), 130.4 (C6'), 129.4 (C5), 128.8 (C7'), 117.6 (C10), 115.1 (C4), 107.2 (C2), 100.8 (C3), 59.6 (C2'), 39.9 (C8), 38.9 (C3'), 30.1 (C9).

HRMS (**ESI**): (m/z) calcd for $C_{20}H_{21}{}^{79}Br_2N_6O_3^+$ $[M+H]^+$: 551.0036, found 551.0034.

IR ν_{max}: 3223 (br), 2892 (w), 2769 (w), 1694 (s), 1623 (s), 1527 (m), 1450 (m), 1327 (w), 1200 (m).

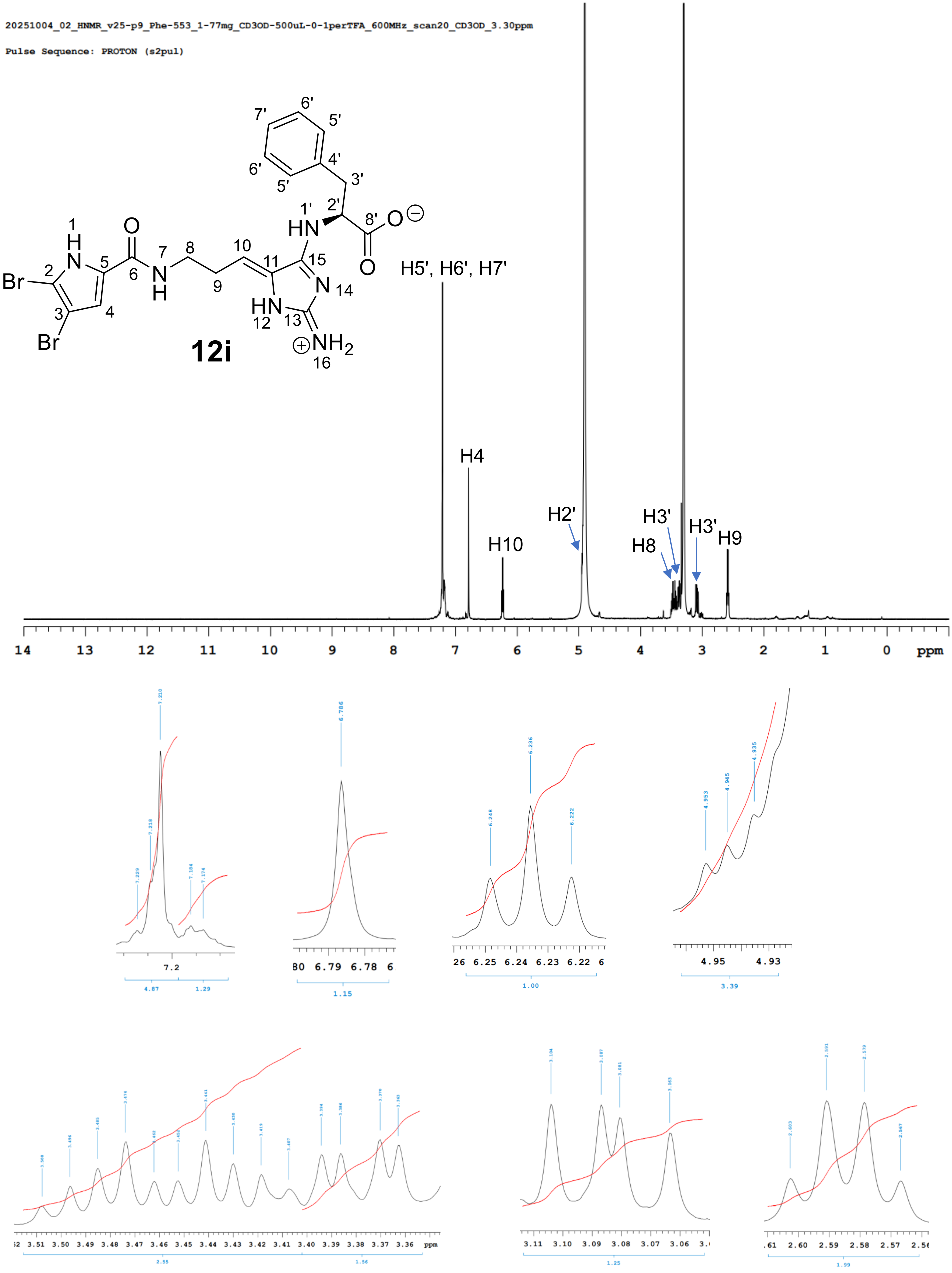

Figure S130. ^{1}H NMR spectrum of **12i** (1.77 mg) (600 MHz, CD_3OD: 500 µL - 0.1% TFA).

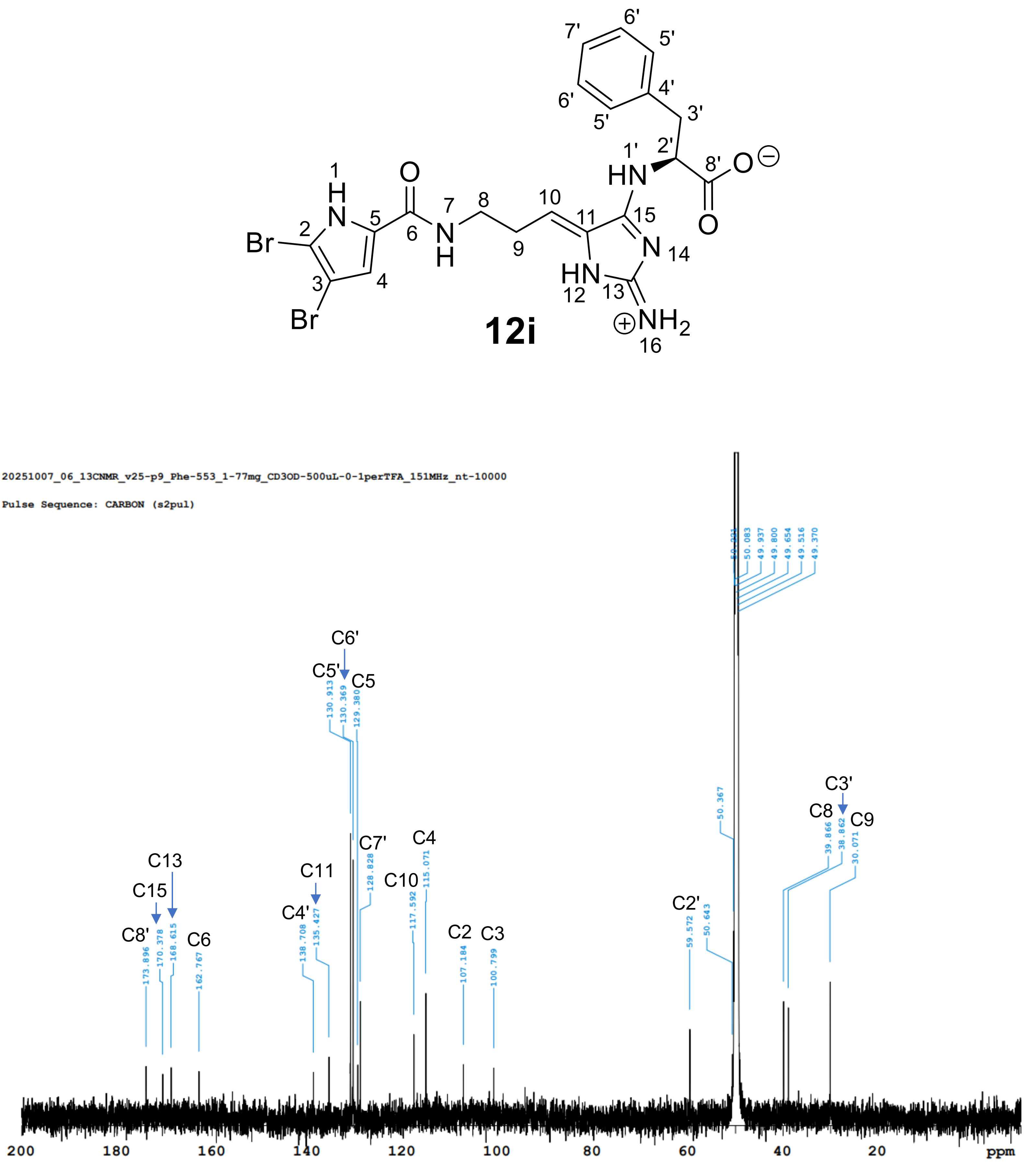

Figure S131. ^{13}C NMR spectrum of **12i** (1.77 mg) (151 MHz, CD_3OD: 500 µL - 0.1% TFA).

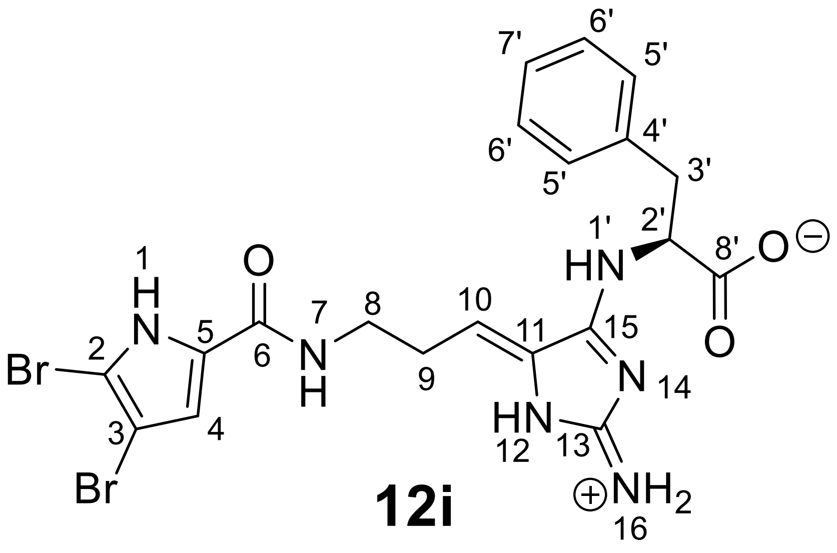

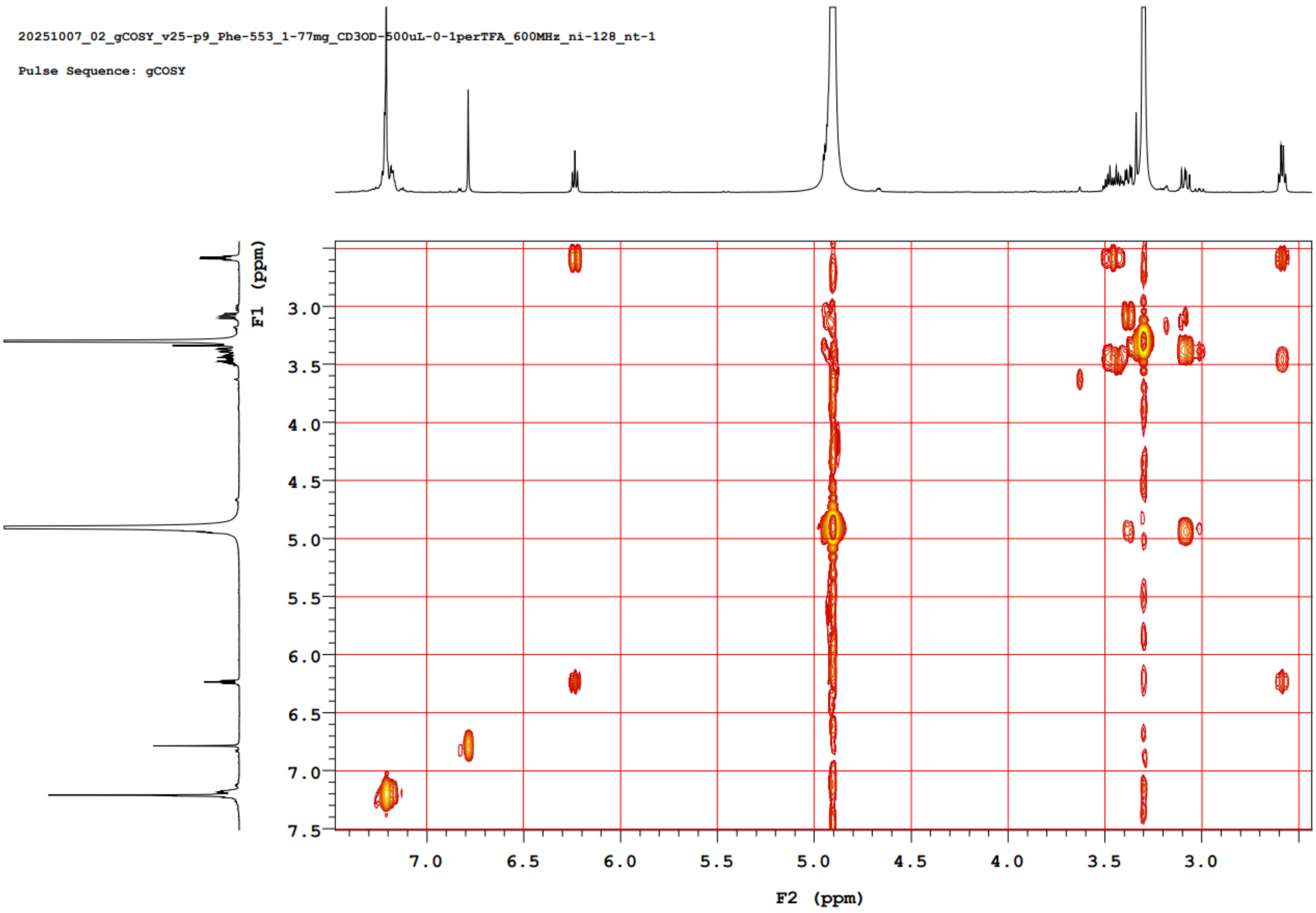

Figure S132. COSY spectrum of **12i** (1.77 mg) (600 MHz, CD_3OD: 500 µL - 0.1% TFA).

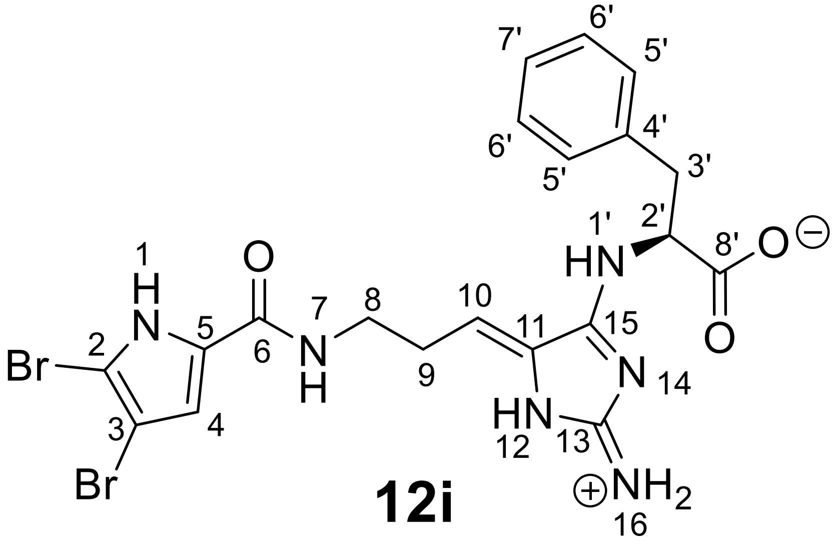

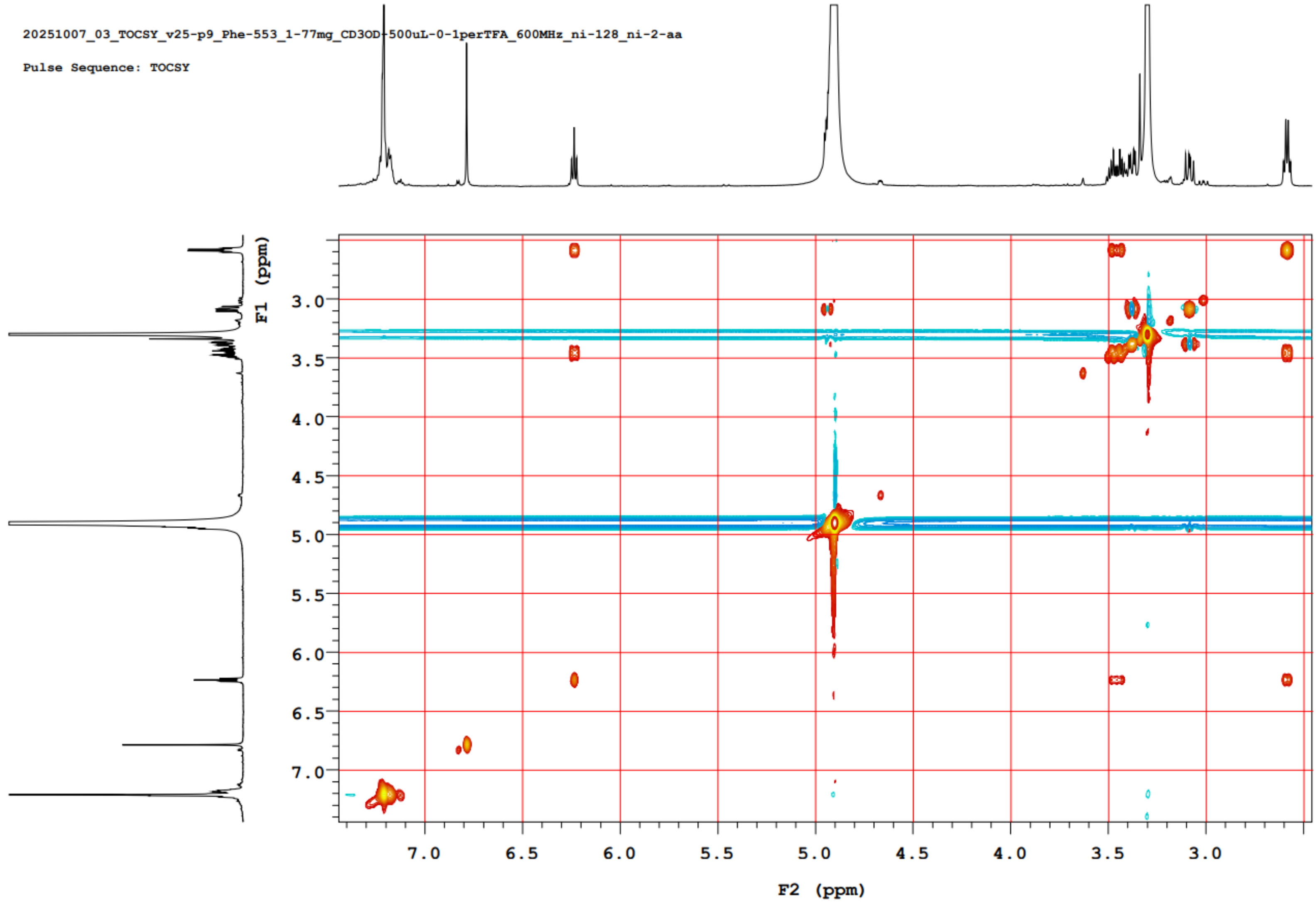

Figure S133. TOCSY spectrum of **12i** (1.77 mg) (600 MHz, CD_3OD: 500 µL - 0.1% TFA).

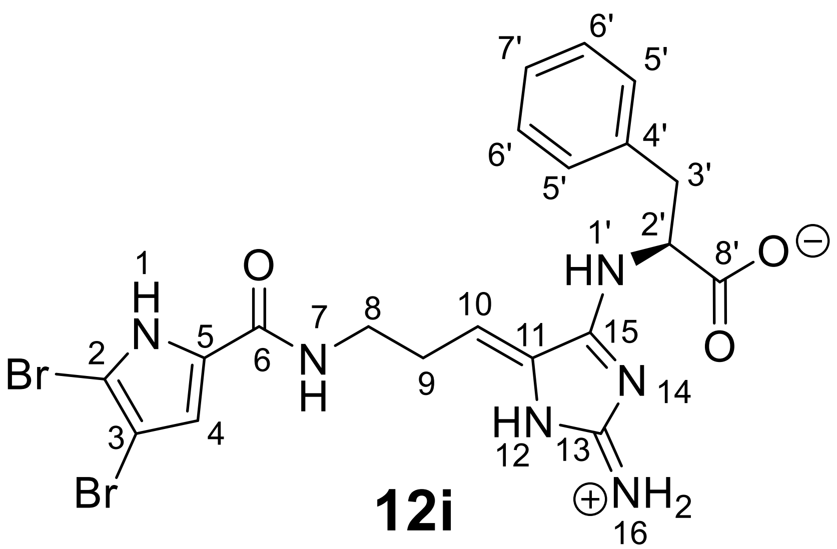

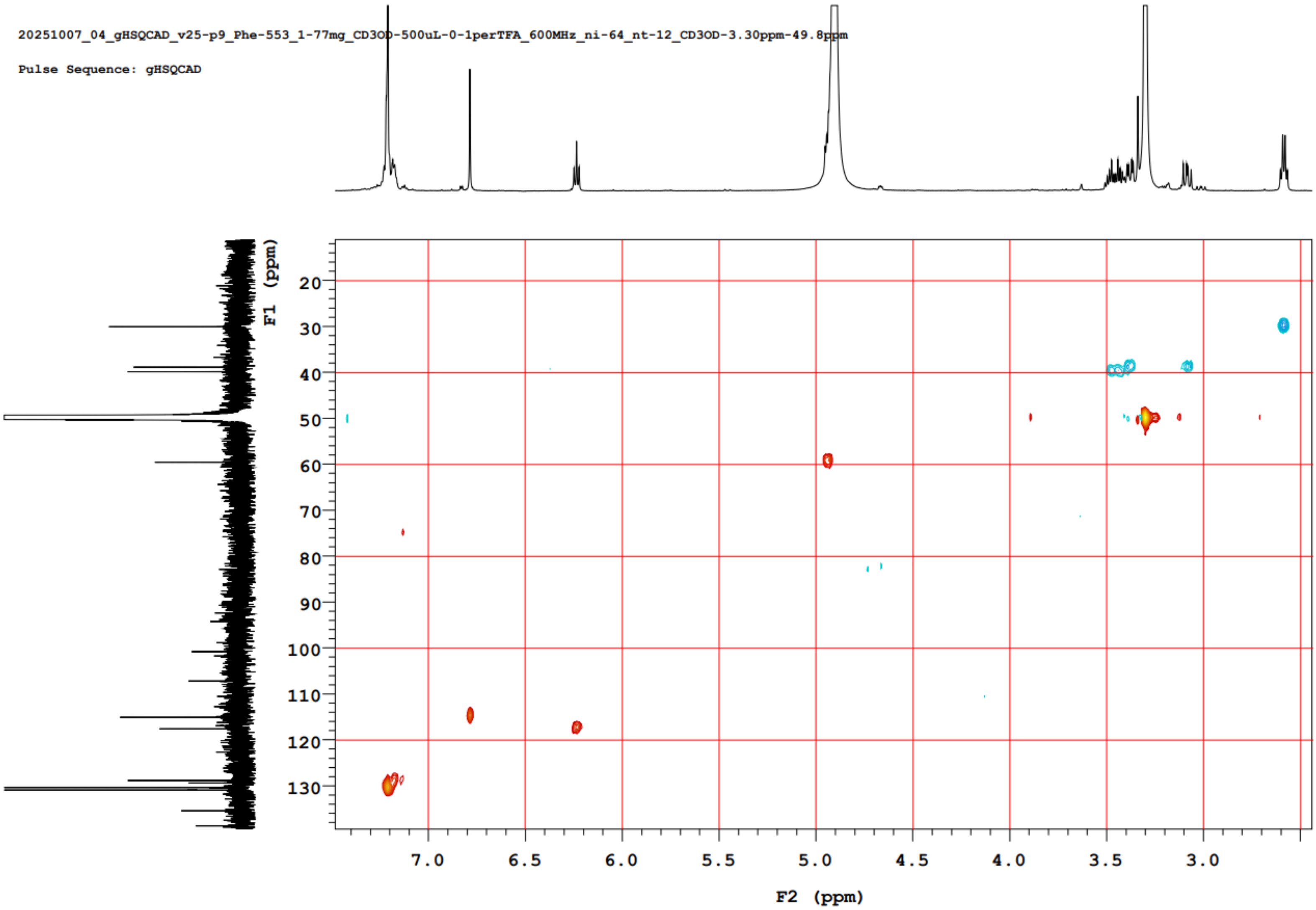

Figure S134. ^{1}H-^{13}C HSQC spectrum of **12i** (1.77 mg) (600 MHz/151 MHz, CD_3OD: 500 µL - 0.1% TFA).

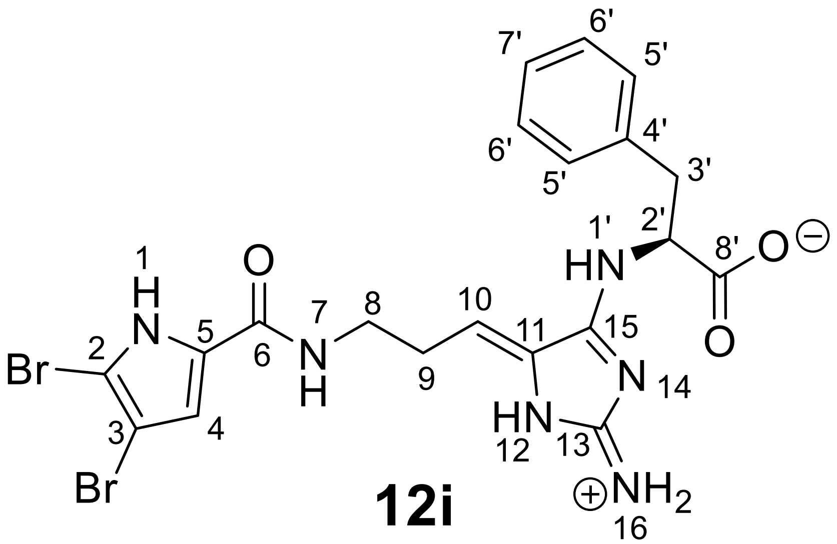

12i

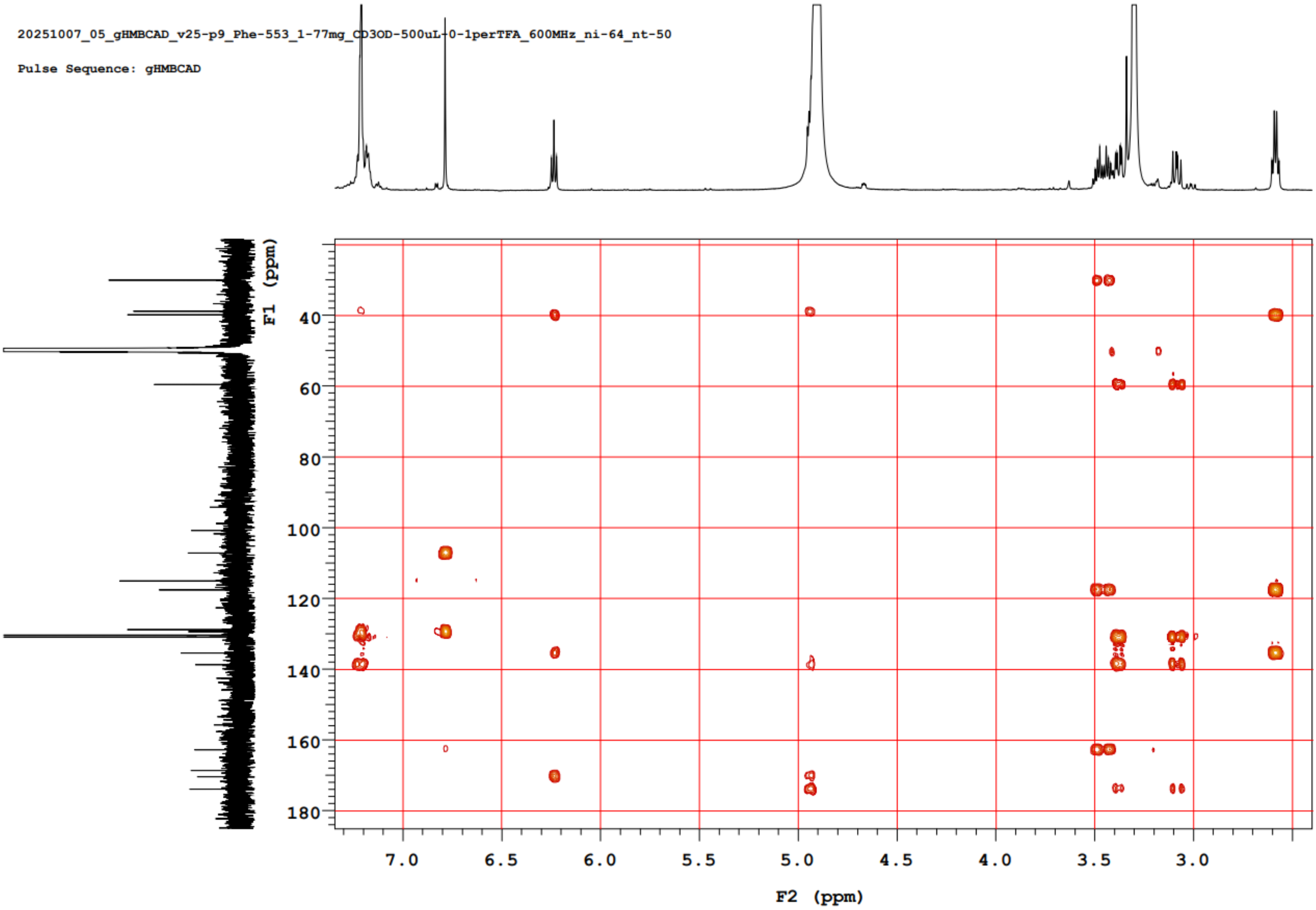

Figure S135. ^{1}H-^{13}C HMBC spectrum of **12i** (1.77 mg) (600 MHz/151 MHz, CD_3OD: 500 µL - 0.1% TFA).

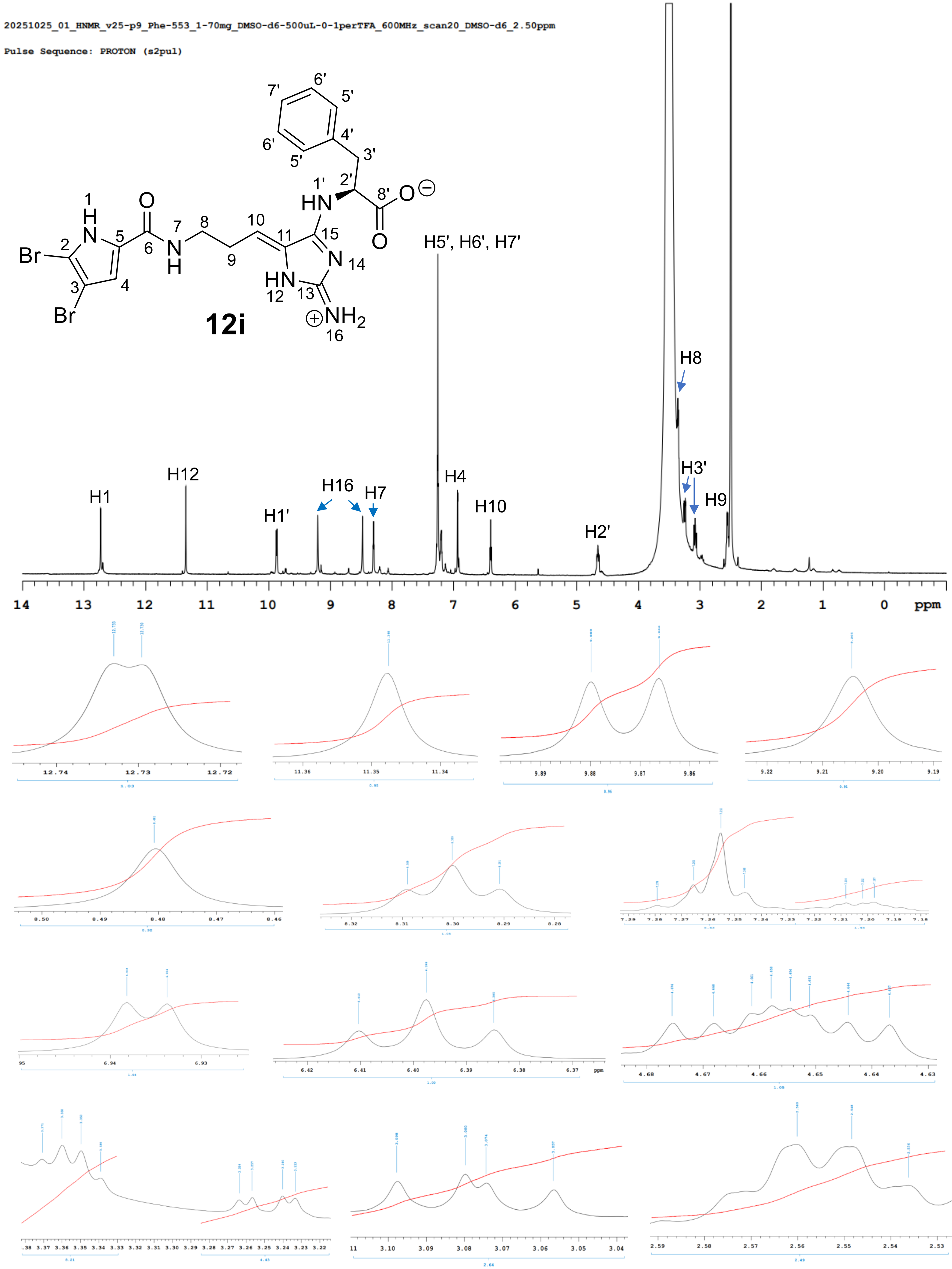

Figure S136. ^{1}H NMR spectrum of **12i** (1.70 mg) (600 MHz, DMSO-d_6: 500 µL - 0.1% TFA).

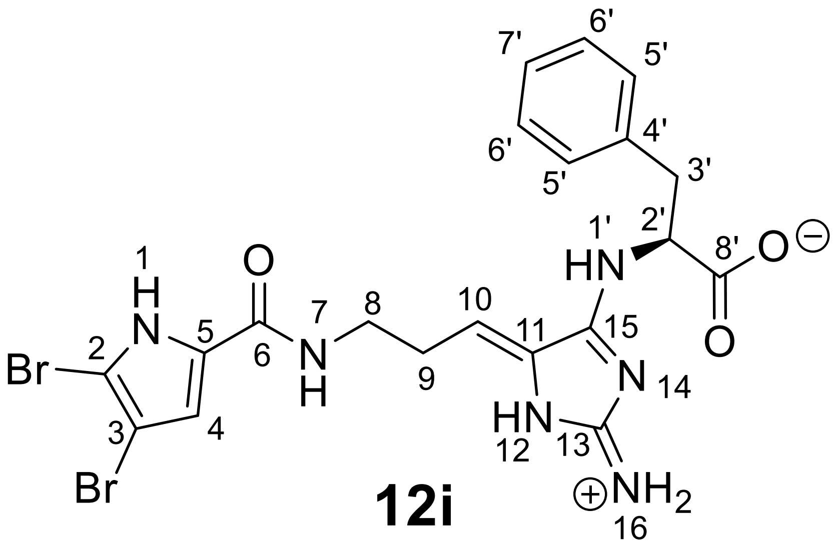

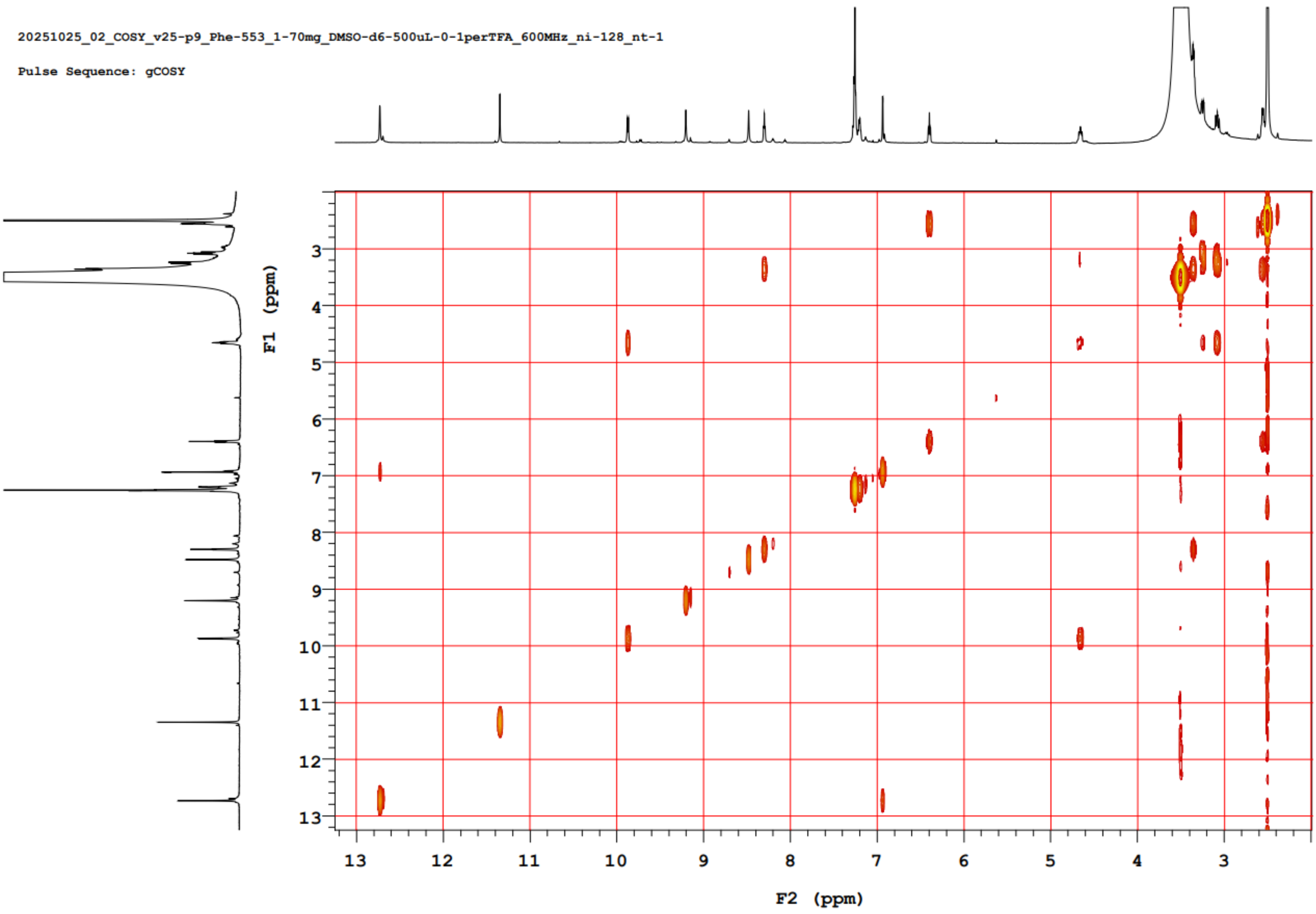

Figure S137. COSY spectrum of **12i** (1.70 mg) (600 MHz, DMSO-d_6: 500 µL - 0.1% TFA).

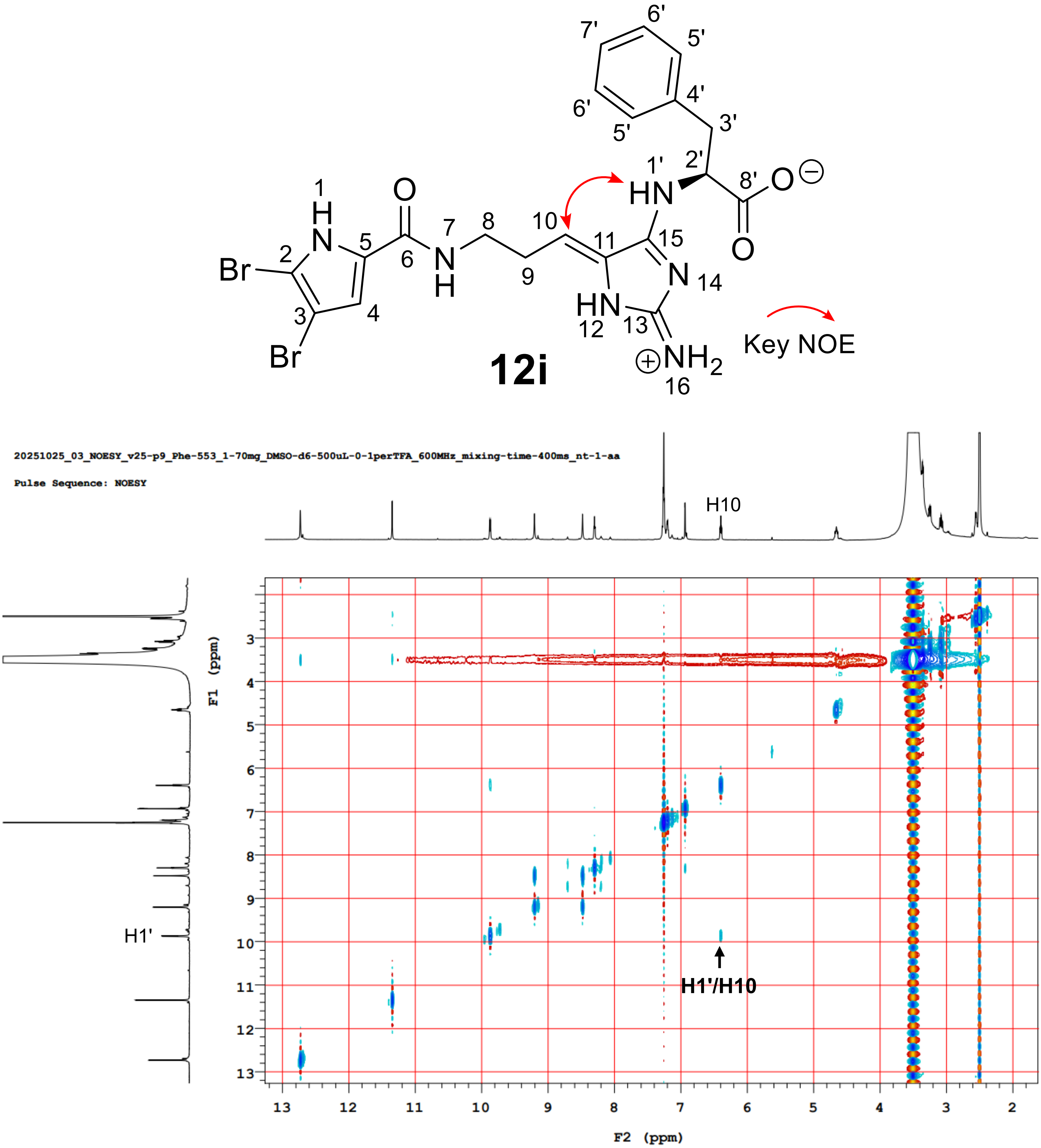

Figure S138. NOESY spectrum of **12i** (1.70 mg) (600 MHz, DMSO-d_6: 500 µL - 0.1% TFA).

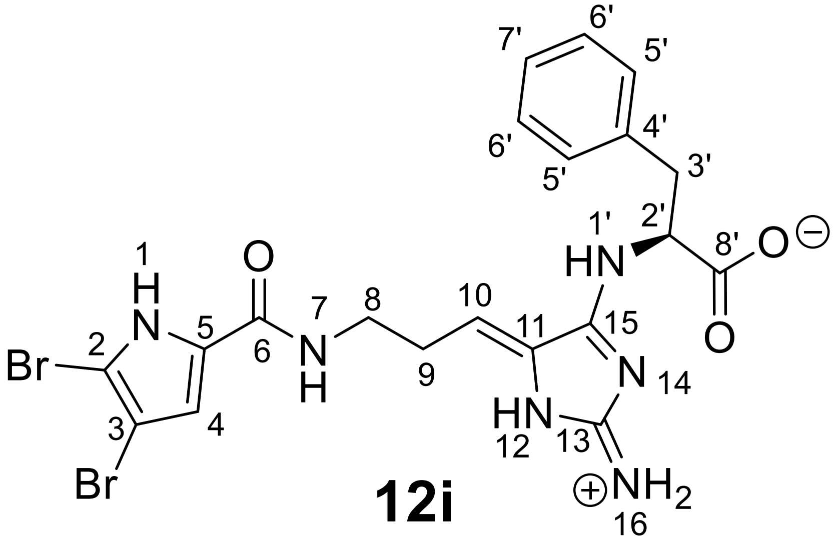

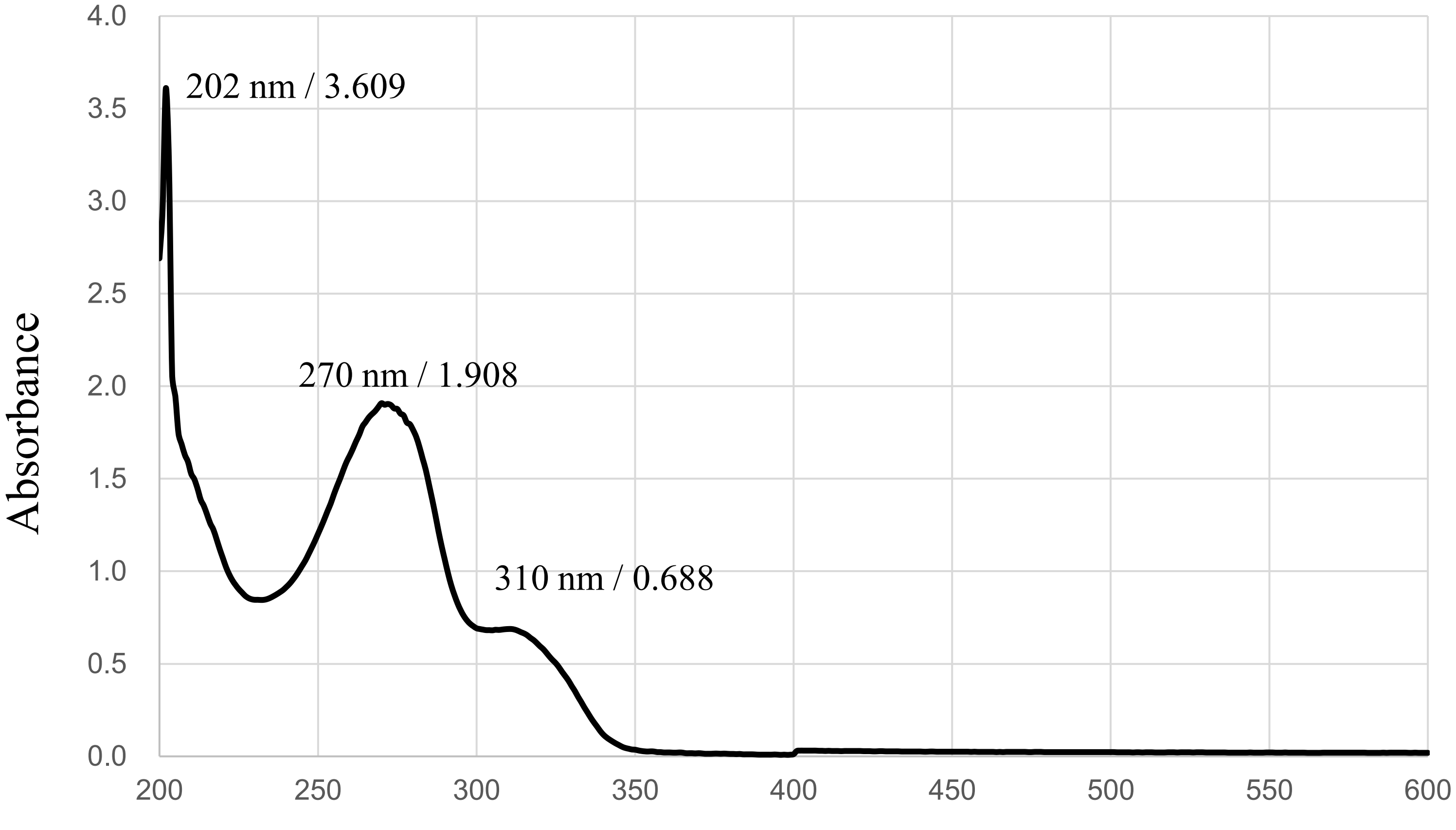

Figure S139. UV absorption spectrum of **12i** (MeOH). c = 1.13 x 10^{-4} (M)

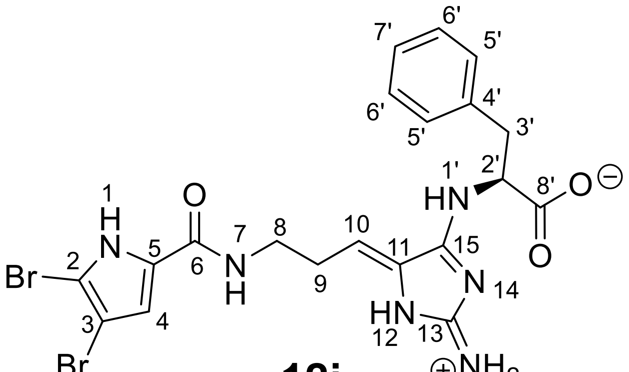

12i

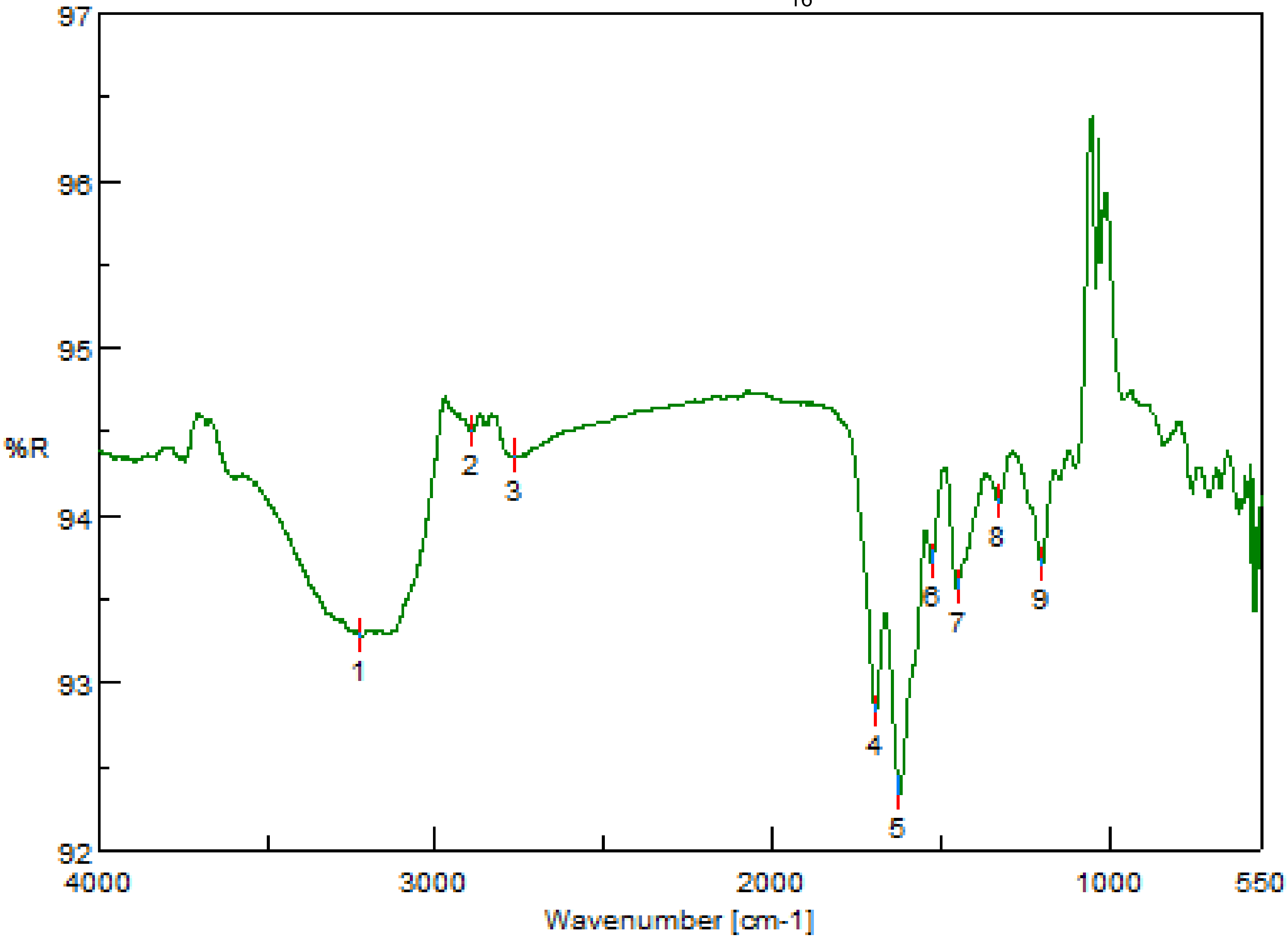

No.	Wavenumber	Strength
1	3222.5	93.2762
2	2891.7	94.4995
3	2769.3	94.3541
4	1694.2	92.8265
5	1622.8	92.3386
6	1527.4	93.721
7	1450.2	93.5679
8	1326.8	94.0787
9	1199.5	93.705

Figure S140. IR spectrum of **12i** (ATR).

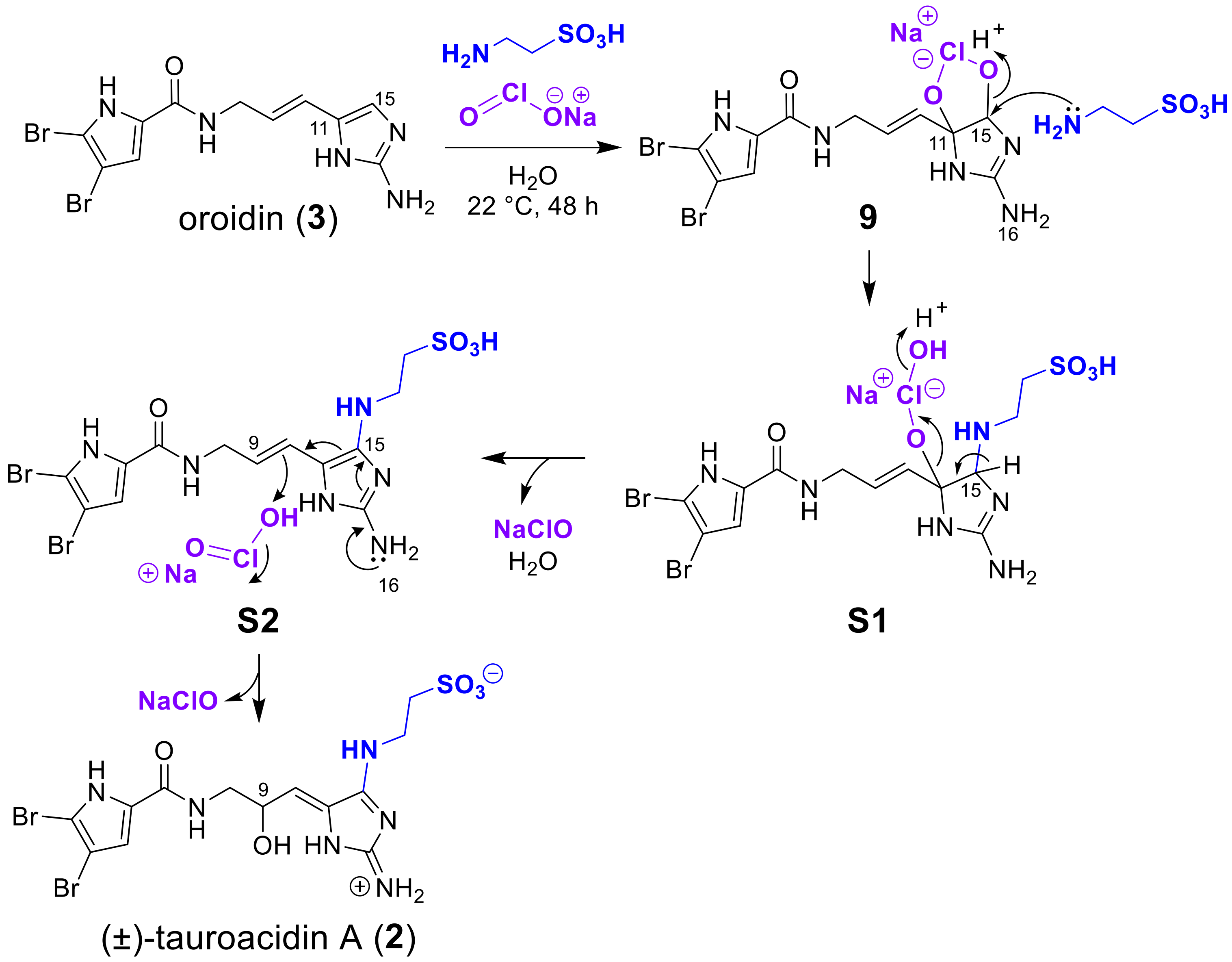

Scheme S10. Possible mechanism for the formation of (±)-tauroacidin A (**2**) from oroidin (**3**) with $NaClO_2$ and taurine.

Table S4. Amino acids that could not be introduced into oroidin (**3**).

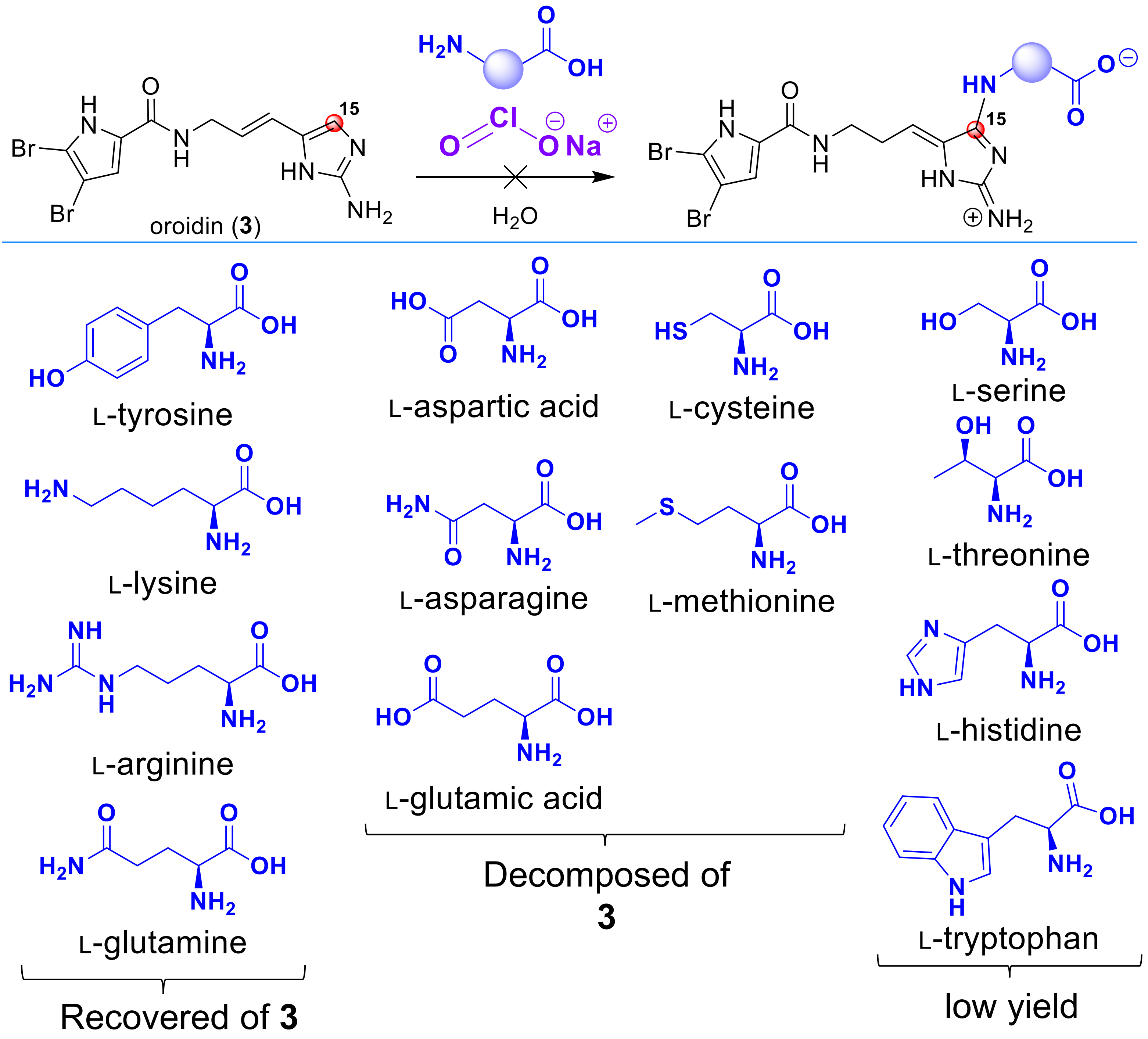

L-Tyrosine (Tyr), L-lysine (Lys), L-arginine (Arg), and L-glutamine (Gln) led only to the recovery of oroidin (**3**). L-Aspartic acid (Asp), L-asparagine (Asn), L-glutamic acid (Glu), L-cysteine (Cys), and L-methionine (Met) caused decomposition of oroidin (**3**), yielding no desired products. L-Serine (Ser), L-threonine (Thr), L-histidine (His) and L-tryptophan (Trp) gave mass spectrometric peaks suggestive of product formation; however, the yields were extremely low or poorly reproducible, and thus they were omitted from Table 1. Other nucleophiles such as glycylglycine and glucosamine resulted solely in decomposition of oroidin (**3**).

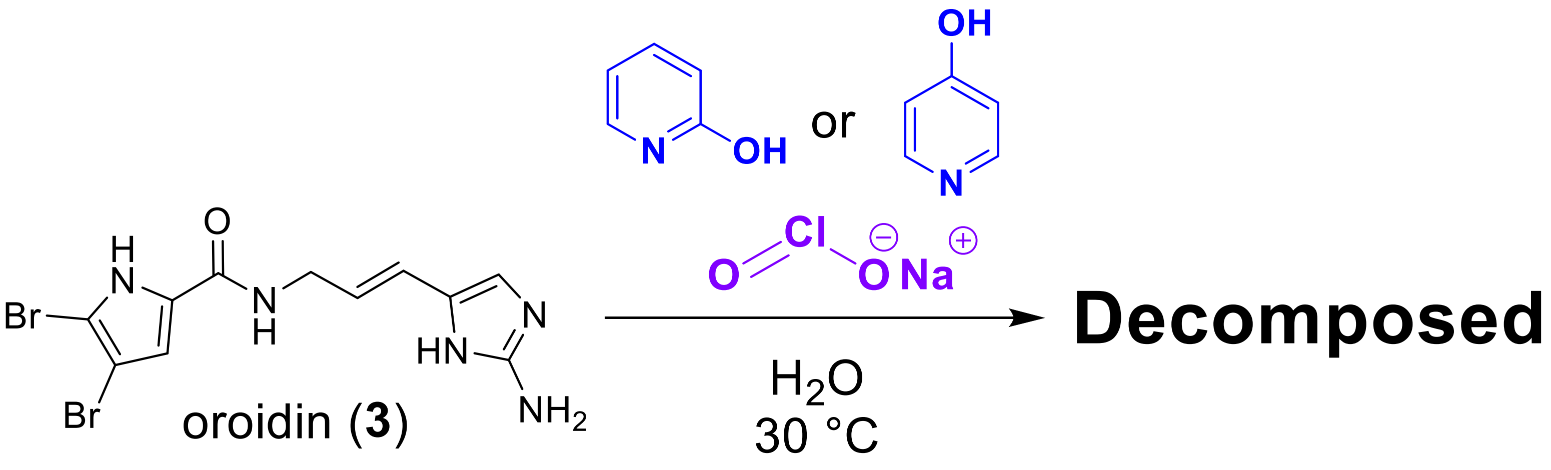

Scheme S11. Reaction of oroidin (**3**) with 2-hydroxypyridine or 4-hydroxypyridine using sodium chlorite.

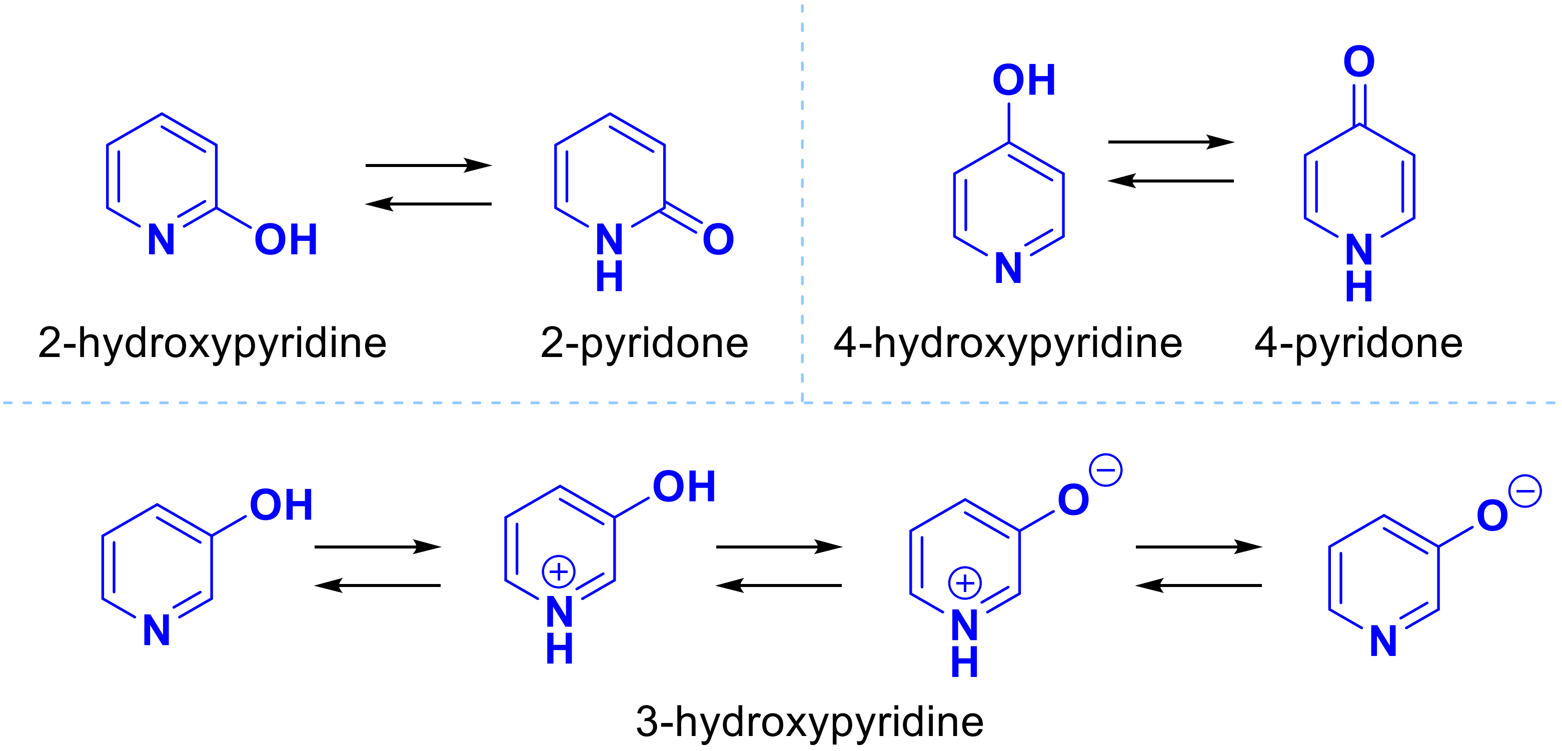

Scheme S12. Isomerization of hydroxypyridine isomers.[28,29]

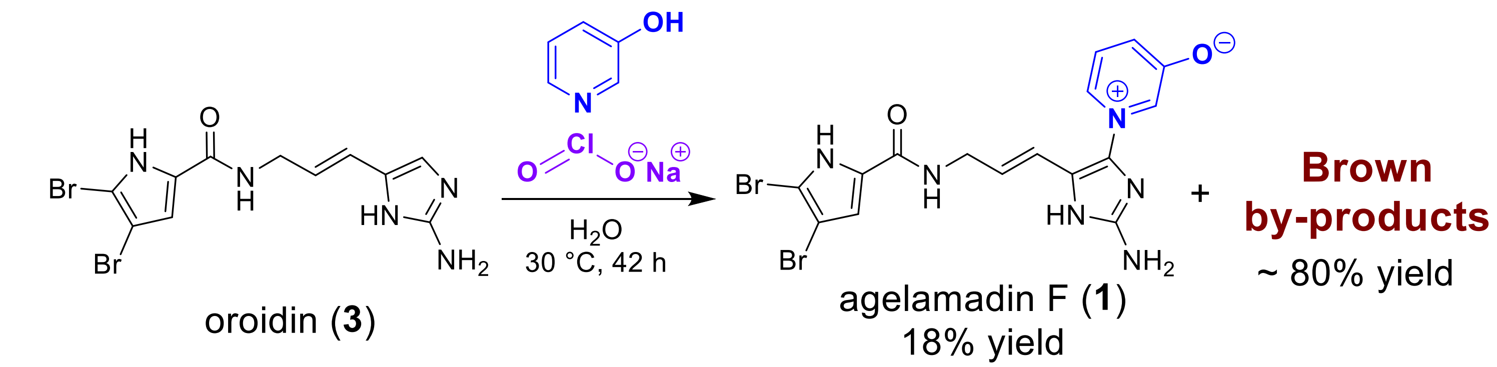

Scheme S13. Product and by-products during the synthesis of agelamadin F (**1**).

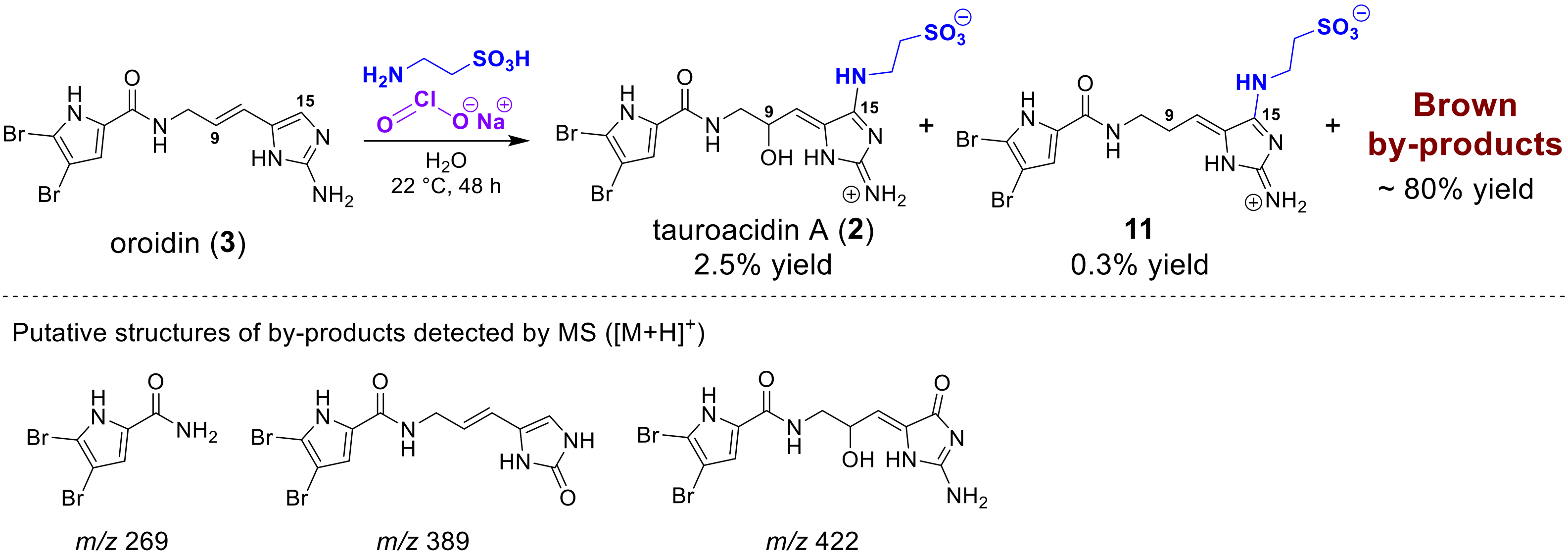

Scheme S14. Product and putative by-products during the synthesis of tauroacidin A (**2**) based on MS spectra.

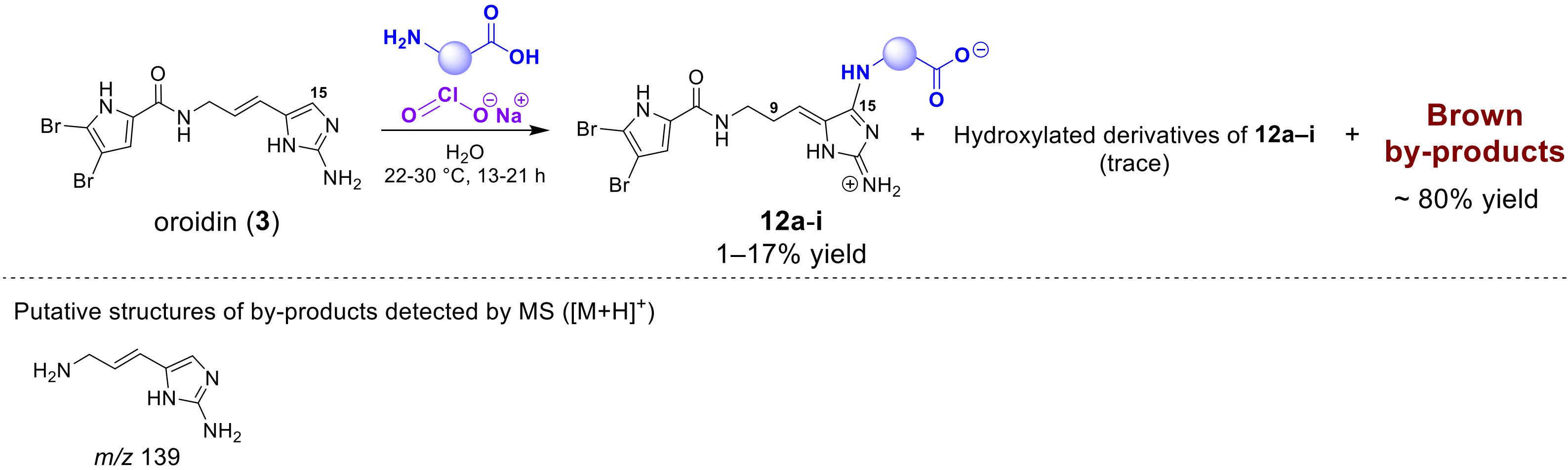

Scheme S15. Product and putative by-products during the synthesis of compounds **12a–i** based on MS spectra.
